# Tetrahydropyrazolopyridinones as a Novel Class of Potent and Highly Selective LIMK Inhibitors

**DOI:** 10.1021/acs.jmedchem.5c00974

**Published:** 2025-08-06

**Authors:** Alex G. Baldwin, David W. Foley, D. Heulyn Jones, Hyunah Lee, Ross Collins, Ben Wahab, Josephine H. Pedder, Loren Waters, Marie Paine, Lauramariú Schino, Gui Jie Feng, Benson M. Kariuki, Jonathan M. Elkins, John R. Atack, Simon E. Ward

**Affiliations:** † Medicines Discovery Institute, School of Biosciences, Cardiff University, Main Building, Park Place, Cardiff CF10 3AT, United Kingdom; ‡ Centre for Medicines Discovery, University of Oxford, Roosevelt Drive, Oxford OX3 7FZ, United Kingdom; § School of Chemistry, Cardiff University, Main Building, Park Place, Cardiff CF10 3AT, United Kingdom

## Abstract

LIMKs are serine/threonine and tyrosine kinases that play critical roles in regulating actin filament turnover, affecting key cellular processes such as cytoskeletal remodeling, proliferation and migration. Aberrant LIMK overactivation has been implicated in several diseases, including cancers and neurodegenerative disorders. Understanding the precise molecular mechanisms by which LIMKs modulate actin cytoskeletal dynamics necessitates highly potent and selective LIMK pharmacological inhibitors. We report the discovery of a novel class of allosteric dual-LIMK1/2 inhibitors based on the tetrahydropyrazolopyridinone scaffold. Using structure-based drug design, we identified MDI-117740 (**69**) as a highly potent dual-LIMK1/2 inhibitor with significantly improved DMPK properties compared to prior inhibitors, suitable for *in vivo* evaluation. Importantly, **69** has very low kinome promiscuity, including former off-target RIPK1, representing the most selective LIMK inhibitor reported to date. Such a chemical probe will enable researchers to selectively dissect LIMK activation under physiological or disease conditions and spur translation of new therapeutics targeting LIMK pathologies.

## Graphical abstract

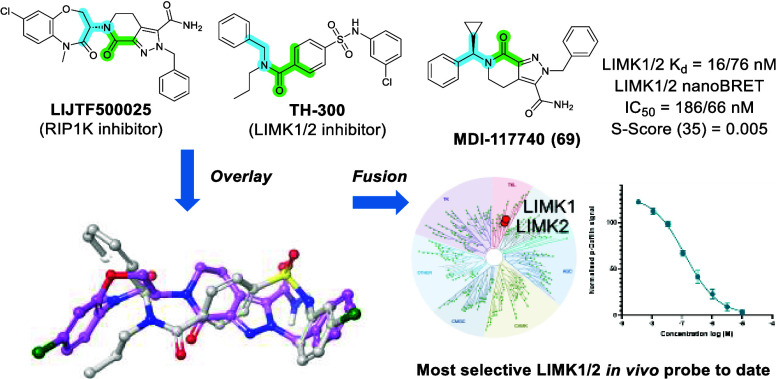

## Introduction

The LIM domain kinases (LIMKs) are dual specificity, serine/threonine and tyrosine kinases that belong to the tyrosine-kinase family (TKL). They are a family of proteins comprised of two highly conserved members, LIMK1 and LIMK2, sharing 50% overall sequence similarity with 70% identity in the kinase domain. LIMKs have a unique structure of signaling domains composed of two N-terminal LIM domains and a PDZ domain, which are important in regulating kinase activity, a proline/serine-rich region and a C-terminal kinase domain.^1–3^ LIMK1/2 are a convergence point for at least four different signaling pathways: (i) the Rac-PAK (p21-activated kinase), (ii) Rho-ROCK (Rho kinase), (iii) Cdc42-MRCKα (myotonic dystrophy kinase-related Cdc42-binding kinase), and (iv) CaMKIV (Ca^2+^/calmodulin-dependent protein kinase) cascades. These upstream pathways all activate LIMKs by phosphorylating a conserved threonine residue, Thr508 in LIMK1 and Thr505 on LIMK2, on the activation loop.^4–8^ LIMK activation leads to phosphorylation at Ser3 of its substrates cofilin 1, cofilin 2, and destrin (actin depolymerizing factor) family of proteins, commonly referred to as “cofilin”.^1,9^ This phosphorylation event inactivates cofilin, causing actin polymerization and stabilization. Therefore, LIMKs are key regulators of actin stability and control cytoskeletal dynamics responsible for cell motility, proliferation and migration, in addition to synapse stability. There is also evidence that LIMKs regulate microtubule stability independently of their central role in modulating actin filament dynamics through interaction of its PDZ domain with tubulin.^10^

Inappropriate activation or overexpression of LIMK1 and LIMK2 has been associated with increased cellular invasion and tumor growth in several cancers, including breast,^11–13^ gastric,^14^ prostrate^15,16^ and colorectal cancers.^17^ Fragile X Syndrome (FXS), the most common hereditary cause of intellectual disability and autism spectrum disorder (ASD), is caused by gene silencing of the fragile X mental retardation 1 protein (FMRP).^18^ This leads to LIMK1 activation due to increased levels of full length bone morphogenetic protein type II receptor (BMPR2) that directly binds LIMK1 ^19^ as well as increased activation through the Rac1-PAK1 pathway.^20,21^ Such inappropriate LIMK1 activity drives abnormal synaptic and dendritic spine morphology in the well-established *Fmr1* KO mouse model^20^ and Drosophila model of FXS,^22^ which can also be observed in FXS individuals.^19,23,24^ LIMK dysregulation has also been implicated in other CNS disorders, particularly Alzheimer’s disease,^25^ amyotrophic lateral sclerosis^26^ and schizophrenia.^27,28^

Given the pivotal role of LIMKs in disease and their therapeutic potential, there has been a growing number of reported LIMK inhibitors.^9,29^ The most characterized inhibitor is LIMKi3 (**1**, BMS-5, Figure 1), a highly potent, type I dual LIMK1/2 inhibitor originally developed by Bristol-Myers Squibb.^30^ **1** inhibits cell proliferation and motility in several cancer cell lines,^15,31,32^ reverses abnormal dendritic spine morphology and normalizes anxiety-related behavior in the *Fmr1* KO mouse model.^19,22^ Nevertheless, it has promiscuous kinome selectivity^33^ and has not been progressed further. FRAX486 (**2**, Figure 1) is a highly potent, CNS-penetrant group I PAK inhibitor that restores abnormal synaptic morphology and rescues seizures and behavioral abnormalities in *Fmr1* KO mice^20,34^ that was progressed as a clinical candidate for FXS. However, we have shown that **2**is unselective and strongly inhibits LIMK1/2,^29^ therefore its efficacy in FXS models is likely to be attributed to dual PAK1/LIMK inhibition. The pyrrolopyrimidines LX7101 (**3**), developed by Lexicon Pharmaceuticals, and SR7826 (**4**, Figure 1) have also been disclosed as potent type I LIMK inhibitors.^35–37^ **3**is a dual LIMK/ROCK inhibitor that advanced to phase 1/2a clinical trials for primary open-angle glaucoma or ocular hypertension.^38^ **4**, an analogue devoid of ROCK inhibition, protects against Aβ-induced hippocampal thin spine loss, dendritic spine degeneration^25^ and rescues impaired hippocampal long-term potentiation in neonatal *Fmr1* KO mice.^39^ However, both **3** and **4** remain as unselective, type I LIMK1/2 inhibitors.^29,40^

**Figure 1:**
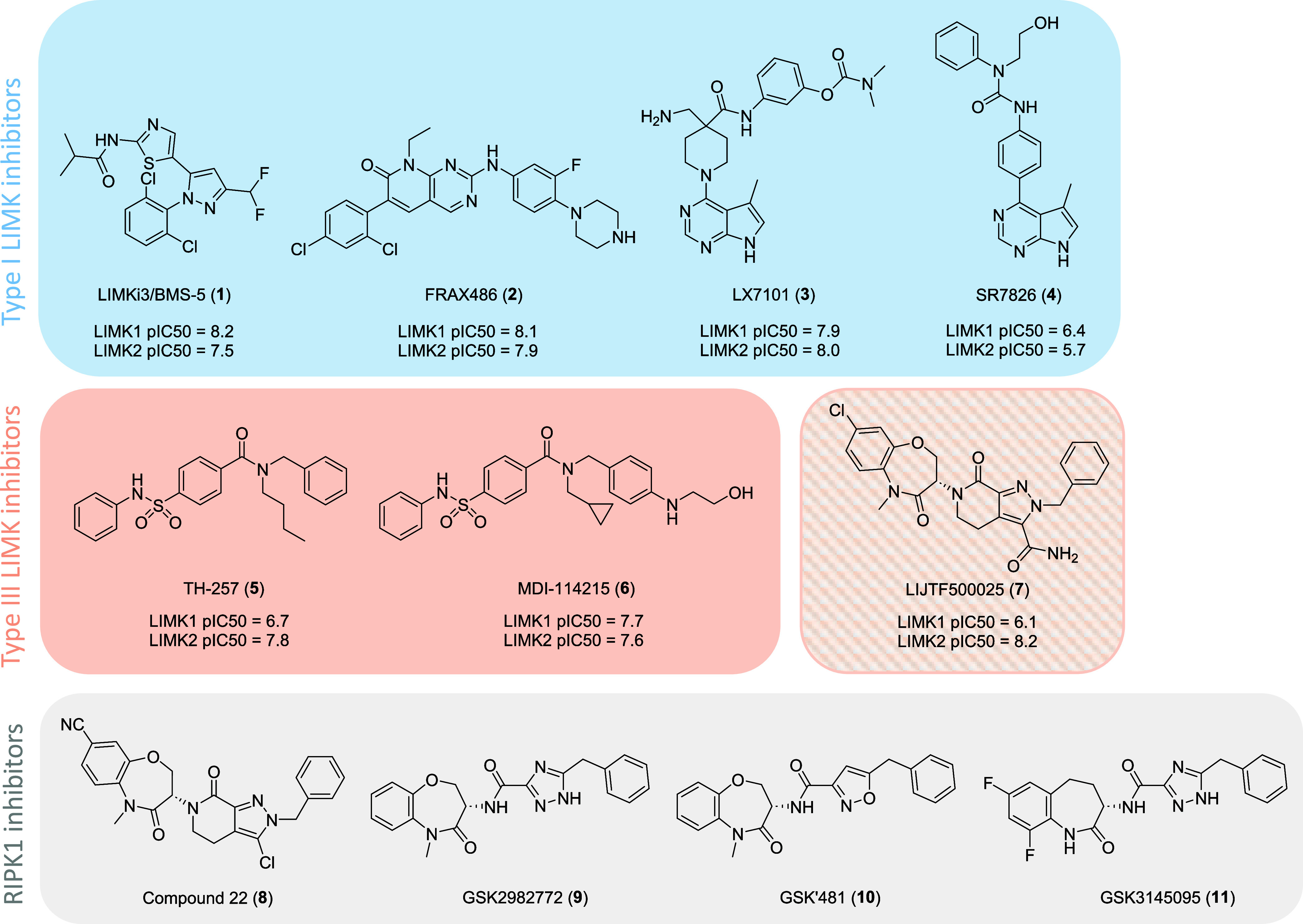
Chemical structures of reported LIMK1/2 inhibitors and structurally related RIPK1 inhibitors.

Consequently, there has been greater interest in developing allosteric type III LIMK1/2 inhibitors that are more selective and whose potencies are not appreciably different in PAK-phosphorylated LIMK variants.^29^ TH-257 (**5**, Figure 1) reported by Knapp et al.^33^ and closely related to a series first discovered by Lexicon Pharmaceuticals,^41^ is a highly selective, type III dual LIMK1/2 inhibitor that dose-dependently inhibits neurite outgrowth and reduces phospho-cofilin levels in human iPSC-derived cortical neurons from FXS patients.^39^ We recently disclosed the very potent dual LIMK1/2 inhibitor MDI-114215 (**6**), which significantly reduces phospho-cofilin in hippocampal brain slices and rescues impaired long-term potentiation in the *Fmr1* KO mouse model. Unfortunately, compounds **5** and **6** could not be progressed further due to their suboptimal DMPK properties and poor CNS penetration, respectively.^29^ ,^33^ ,^39^ Therefore, alternative starting points to develop allosteric type III LIMK1/2 inhibitors with improved *in vitro* DMPK properties suitable for *in vivo* evaluation are highly desirable.

The X-ray crystal structure of the LIMK1 kinase domain bound to LIJTF500025 (**7**, Figure 1) was recently published (PDB: 7ATU).^42^ **7** was originally developed as a potent receptor interacting protein 1 (RIP1) kinase inhibitor by Takeda Pharmaceuticals as part of a CNS-penetrant, orally available RIPK1 inhibitor program leading to compound 22 (**8**, Figure 1).^43^ This series features a tetrahydro-6*H*-pyrazolo­[3,4-*c*]­pyridin-7-one core designed from a merging strategy between an identified HTS hit and the previously reported RIPK1 inhibitor GSK2982772 (**9**, Figure 1).^44^ **9** is a clinical candidate that has recently completed phase II clinical trials for psoriasis, ulcerative colitis and rheumatoid arthritis^45–47^ that constitutes an advanced RIPK1 inhibitor series developed by GlaxoSmithKline, exemplified by GSK′481 (**10**)^48^ and GSK3145095 (**11**, Figure 1).^49^

**7** is a type III, allosteric dual LIMK1/2 and RIPK1 inhibitor whose binding to LIMK1 causes distortion of the P-loop and outward displacement of the αC helix, whereby the DFG motif adopts the DFG-out conformation.^42^ The central tetrahydropyrazolopyridinone core is supported in place with interactions of the carbonyl (and potentially pyrazolo N) with the catalytic D478 of the DFG loop. The ligand also makes an important diatomic interaction of the primary amide with M388/L397 and an edge π-cation interaction of the benzyl ring with H458 (Figure 2A). The narrow lipophilic back pocket is filled by the benzoxazepin-4-one motif which positions the haloaromatic into this hydrophobic region, encompassed by F350, A353, V366, and F479 (Figure 2B). Interestingly, comparable interactions were observed in the X-ray structure of TH-300 (**30**, Figure 3C), a close analogue of **5**, in complex with LIMK2 (PDB: 5NXD) and the LIMK1 homology model of **6**.^39^ Given these similarities, we wished to develop a hybrid series between the RIPK1 inhibitor **7** and our potent dual LIMK1/2 inhibitor **6** that aimed to further improve the suboptimal *in vitro* DMPK properties of 6 while avoiding any related RIPK1 activity.

**Figure 2:**
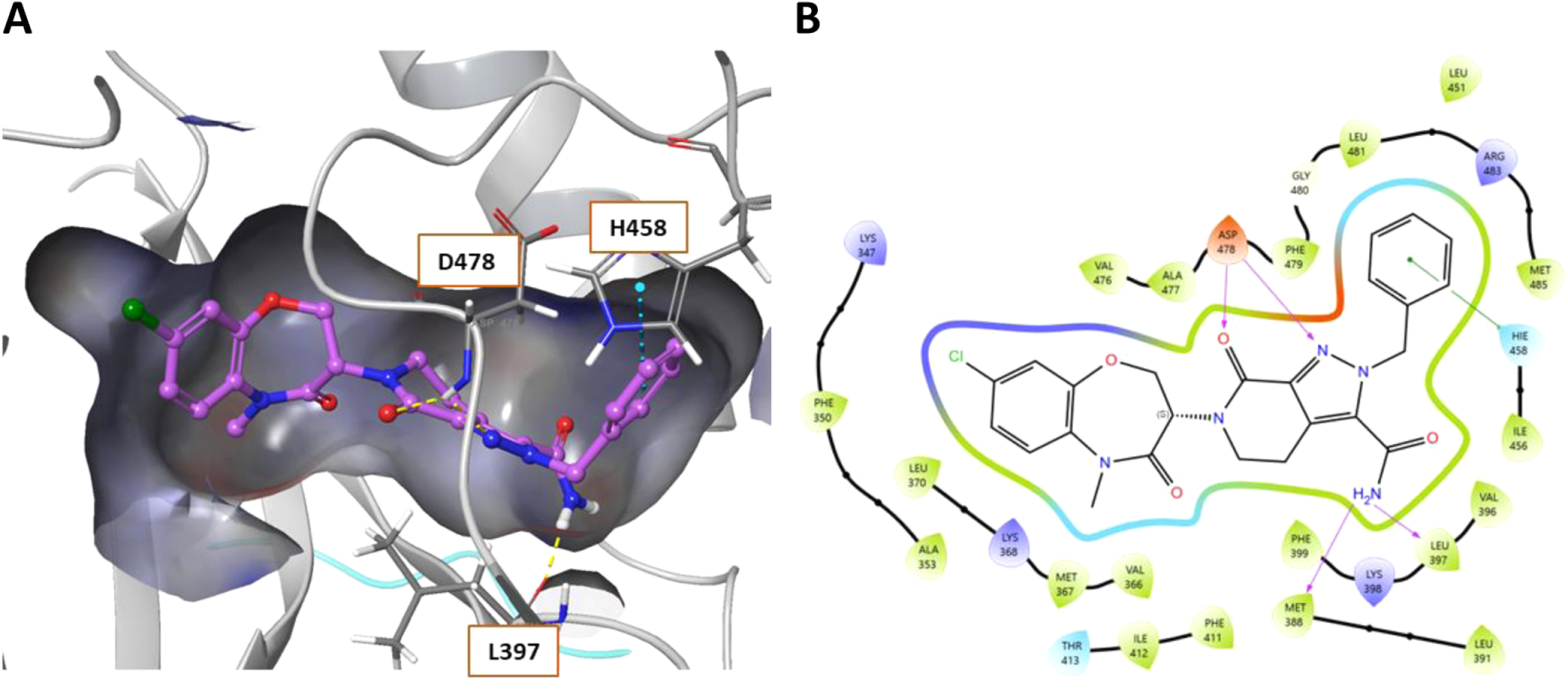
(A) Co-crystal structure of allosteric inhibitor **7** (LIJTF500025) bound to LIMK1 (PDB: 7ATU). (B) Ligand interaction diagram of **7**. Shading represents the following: hydrophobic region (green), charged interaction (positive, blue; negative, red), polar (teal).

Here we report this merging strategy and successive SAR campaigns that led to the discovery of a novel class of highly potent, selective LIMK1/2 inhibitors devoid of RIPK1 binding. The most advanced molecule from this series, MDI-117740 (**69**), has similar LIMK1/2 binding and cellular potency and target engagement but significantly improved *in vitro* DMPK compared to our previously reported lead compound **6**. Notably, **69** is the most selective LIMK inhibitor known to date. We also characterized structure-permeability relationships within this new series of allosteric inhibitors.

## Results and Discussion

To identify the chemical features necessary for potent dual LIMK1/2 activity, structurally related RIPK1 inhibitors **8**-**11** and an initial set of analogues of **7** were screened using our previously reported LIMK1/2 RapidFire mass spectrometry assay.^29^ First, we attempted to access key intermediate 5-cyano-1*H*-pyrazole-3-carboxylate **12** (see Scheme S1) to enable a regioselective synthesis of amide analogues of **7** (Series A, Table 1) via nitrile hydrolysis and amide coupling. 5-Bromo-1*H*-pyrazole-3-carboxylate **14** was synthesized from ethyl 1-benzyl-5-hydroxy-1*H*-pyrazole-3-carboxylate **13** using POBr_3_ and DMF prior to Wittig reaction with (methoxymethyl)­triphenylphosphonium chloride, HCl-mediated deprotection, reductive amination and ester hydrolysis/amide coupling (Scheme S1). Despite our more convergent synthetic route, late-stage Pd-catalyzed cyanation of **19** using Zn­(CN)_2_ ^50^ failed to afford the nitrile derivative **12**. As an alternative, derivatives **20**–**26** were synthesized similar to previously described by Yoshikawa et al.^43^ using dimethyl 1-benzyl-4-(2-oxoethyl)-1*H*-pyrazole-3,5-dicarboxylate **27** and substituted benzoxazepine-4-one starting materials (Scheme S2). Typically, the reductive aminated intermediates **28a**–**b** spontaneously cyclized overnight to afford a mixture of regioisomers. After separation by flash column chromatography and amide coupling, this led to two distinct series: 1-benzylpyrazolo-5-carboxamides (Series A) and 1-benzylpyrazolo-3-carboxamides (Series B, Table 1). Ring-opened analogue **29** was synthesized from **50** (see Scheme 2).

**Table 1:**
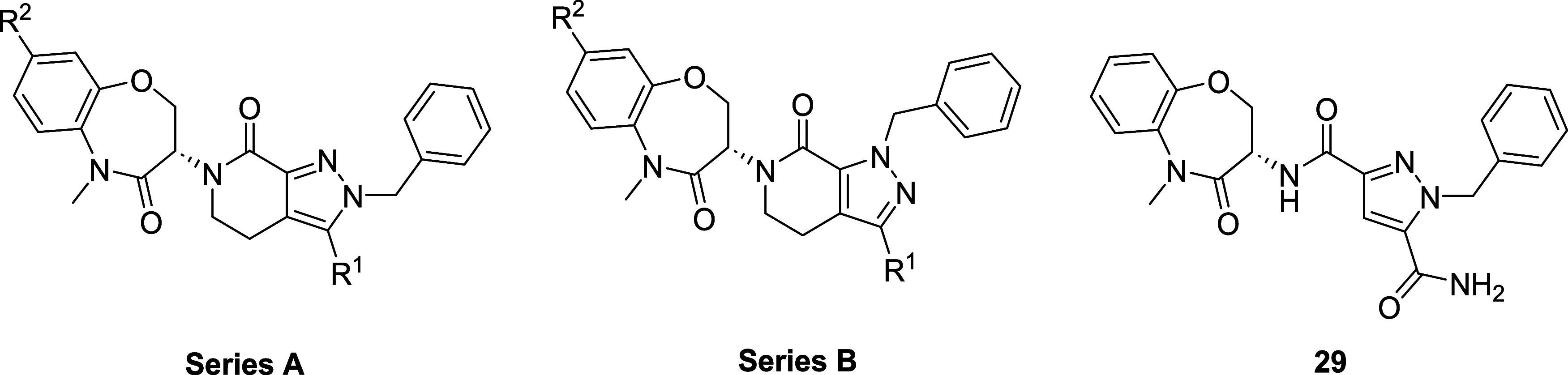
Evaluation of Structurally Related RIPK1 Inhibitors 8–11 and Analogues 19–26 and 29 against LIMK1 and LIMK2 to Explore Early Structure-Activity Relationship (SAR) of LIJTF500025 (7)

				enzymatic pIC_50_ ^a^	
compound	R^1^	R^2^	series	LIMK1	LIMK2
**7**	CONH_2_	Cl	A	7.17^b^	7.82^b^
**8**	Cl	CN	A	<5	5.22^b^
**9**	-			5.04^b^	5.03^b^
**10**	-			5.39^b^	<5
**11**	-			5.13^b^	5.12^b^
**19**	Br	Cl	A	5.29 ± 0.57^c^	5.91 ± 0.35^c^
**20**	CONH_2_	Cl	B	5.24^b^	5.53^b^
**21**	CONH_2_	H	A	6.98 ± 0.03^c^	7.95 ± 0.09^c^
**22**	CONH_2_	H	B	<5	<5
**23**	CONHMe	H	A	5.76^b^	5.82^b^
**24**	CONHMe	H	B	<5	<5
**25**	CONMe_2_	H	A	<5	<5
**26**	CONMe_2_	H	B	<5	<5
**29**	-			<5	5.16^b^

a The phosphorylation of cofilin was assessed by RapidFire mass spectrometry following an enzymatic assay.

b *n* = 1.

c Mean of two independent experiments.

RIPK1 inhibitors **8–11** were not potent LIMK1 or LIMK2 inhibitors (Table 1). Given bromo analogue **19** also demonstrated poor potency, these data strongly suggest the C5-substituted amide moiety is indispensable for dual LIMK1/2 inhibitory activity. Indeed, mono- or dimethylation of the primary amide (**23** and **25**, respectively) led to significant weaker inhibitors (Table 1), consistent with its known HBD interactions with LIMK1 (Figure 2). Interestingly, comparison between the 1-benzylpyrazolo-5-carboxamide and 1-benzylpyrazolo-3-carboxamide regioisomers showed a clear preference for the 5-regioisomer, with the 3-regioisomer of **7** (**20**) displaying at least 100-fold potency drop against LIMK1 and LIMK2. Breaking the annulated 5,6-fused ring similarly led to weak inhibitory activity (**29**, Table 1), as did sulfonamide and secondary amine replacements of the amide in compound **29** (data not shown). Therefore, these initial SAR data indicate the tetrahydropyrazolo­[3,4-*c*]­pyridine-5-carboxamide moiety is a privileged scaffold to develop potent LIMK1/2 inhibitors. As the des-Cl analogue of **7** is also a highly potent RIPK1 inhibitor (compound 20, p*K_i_* = 8.60 ^43^) and expectedly shows high RIPK1 binding in a kinome selectivity panel (27% of control @1 μM, data not shown), we sought to eliminate off-target RIPK1 activity through a hybridization approach with highly selective, allosteric LIMK inhibitors.

Using the X-ray crystal structures of LIMK2 bound to the allosteric inhibitor TH-300 (**30**, PDB: 5NXD), **7** was overlaid in order to identify possible sites for hybrid generation. Although there is variance between the benzoxazepin-4-one and *N*-benzylpropyl motifs, the spatial and electrostatic fields between the 2-benzyl tetrahydropyrazolopyridine-3-carboxamide of **7** and *N*-phenylsulfamoylbenzamide of **30** share high similarity (Figure 3A). In particular, the electrostatic potentials are shared in two loci which demonstrate the common positioning of (i) the cyclic amide carbonyl of **7** and branched amide carbonyl in **30**, and (ii) the primary amide carbonyl of **7** and sulfonamide O in **30** (Figure 3B). Two instances where atoms from both series overlaid well were identified:

(1) Carbonyl carbon of **7** with the quaternary carbon of **30** (highlighted in yellow).
(2) sp^3^ Hybridized carbon of **7** with the benzyl carbon of **30** (highlighted in red, Figure 3C).

**Figure 3:**
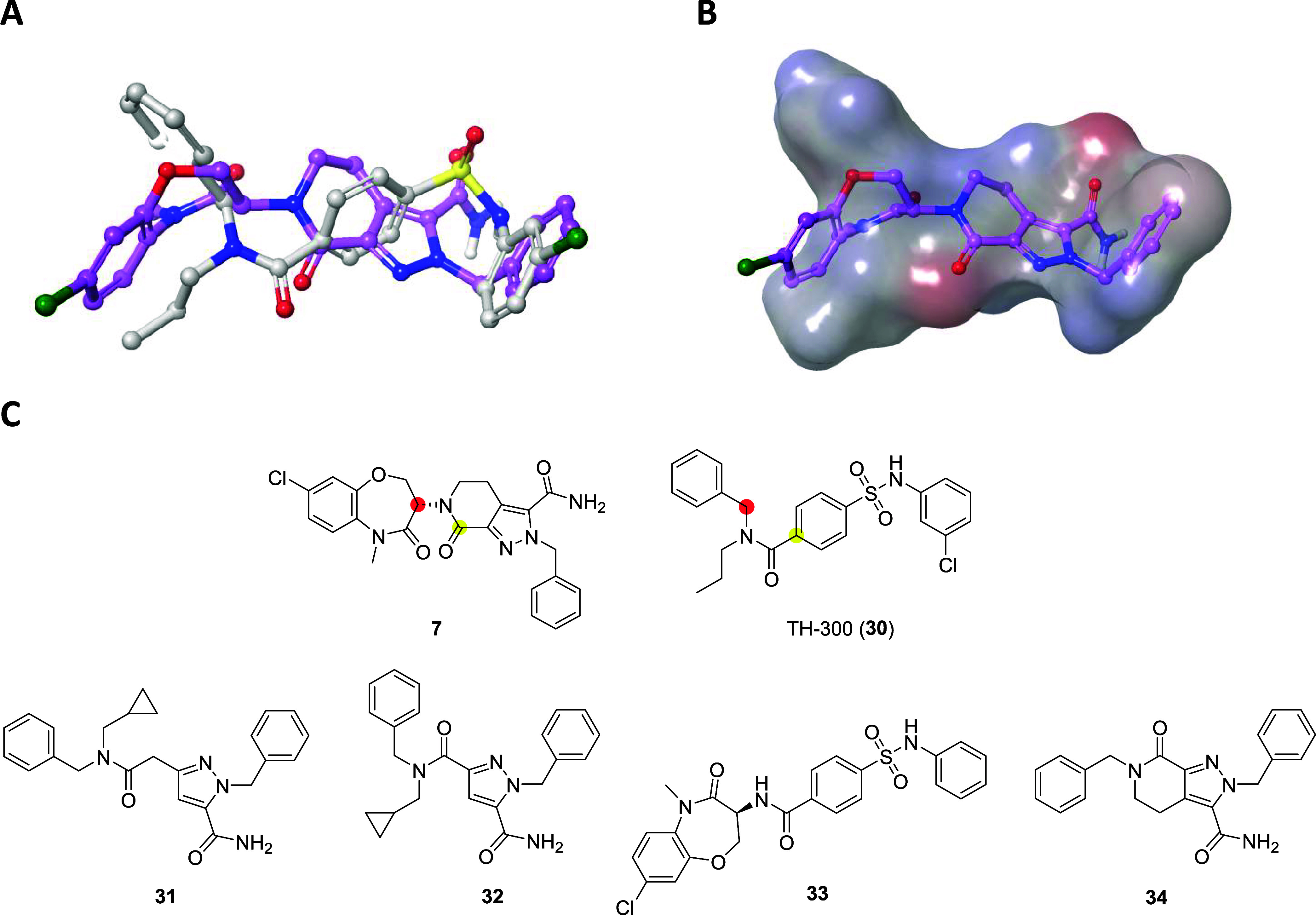
Overlay of RIPK1 inhibitor **7** (LIJTF500025) and **30** (TH-300) and design of novel hybrids **31**-**34**. (A) Binding mode images of **7** (from LIMK1, PDB: 7ATU, magenta) and **30** (from LIMK2, PDB: 5NXD, white). (B) The shape and electrostatic field of **30** (LIMK2, *loc cit*.) with **7** (LIMK1, *loc cit*., magenta) overlaid. (C) Chemical structures of **7** and **30** highlighting key positions for hybrid generation (above) and designed hybrid molecules (below).

For the yellow highlighted position, two hybrids were designed whereby the tetrahydropyrazolopyridone ring was opened and the tertiary amide was either extended by one CH_2_ unit (hybrid **31**) or directly attached (hybrid **32**). In both instances, we targeted synthesis of the methylene cyclopropyl-containing amide as we previously observed a consistent 10-fold LIMK1/2 potency improvement compared to the propyl substituent.^39^ A further two hybrids were proposed based on the second overlaid position highlighted in red; hybrid **33** that replaces the benzyl substituent of **30** with the benzoxazepin-4-one of **7**, and the reversed hybrid **34**, where the tertiary carbon of **7** was replaced with the benzyl group of **30**.

The synthesis of hybrid **31** proved challenging, with first attempts focused on installing the primary amide prior to formation of the tertiary amide bond (Scheme 1). Benzylation of 3,5-dibromopyrazole **35** proceeded cleanly, followed by a reported selective lithiation-quenching with carbon dioxide,^51^ which gave single regioisomer **37**. Generation of the primary amide **38** via the acid chloride followed by a Suzuki reaction with (*E*)-(2-ethoxyvinyl)­boronic acid pinacol ester gave **39**, from which a single crystal was grown to confirm the regioselectivity of the lithiation step (Scheme 1). Unfortunately, acidic deprotection to give the aldehyde **40** was unsuccessful due to intermolecular attack of the primary amide on the aldehyde. Several different strategies to install the CH_2_CO_2_H via bromo intermediate **38** also failed. A change in strategy to unveil the primary amide at the end of the synthesis proved fruitful. Ester formation followed by Suzuki reaction gave intermediate **42** in satisfactory yield. This time, acidic deprotection to give the aldehyde was successful and following oxidation (**44**), *N*-benzyl-1-cyclopropylmethanamine was introduced via amide coupling to afford key intermediate **45**. Finally, ester hydrolysis (**46**) and primary amide formation gave the target compound **31**.

**Scheme 1:**
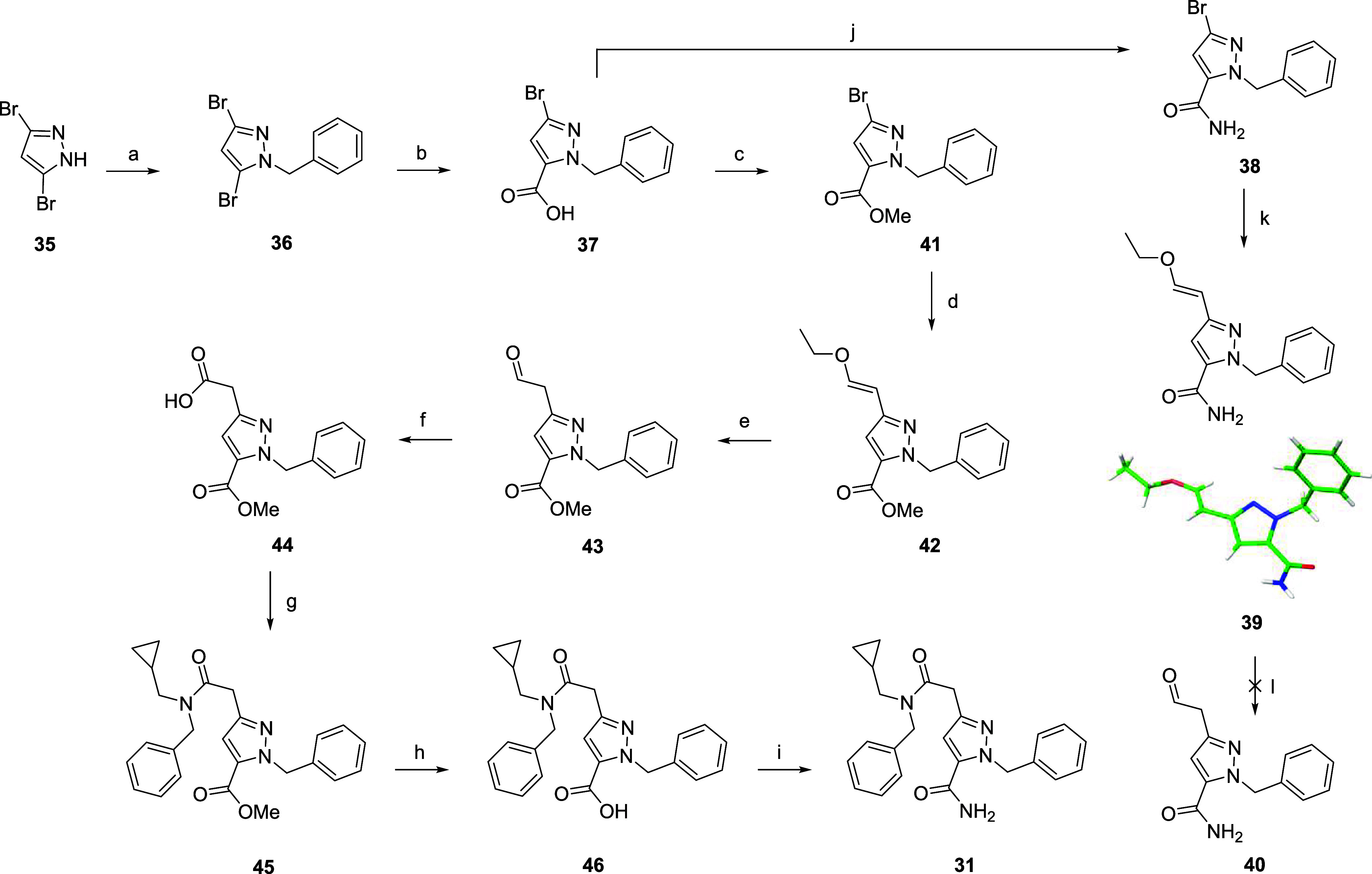
Synthesis of Hybrid 31^a^

a Reagents and conditions: (a) (i) K_2_CO_3_ (1.3 equiv), DMF, 0 °C, (ii) BnBr (1.0 equiv), 0 °C → rt, 40 min, 96%; (b) (i) iPrMgCl·LiCl (1.2 equiv), THF, −78 °C, 30 min, (ii) CO_2_, 20 min, 94%; (c) MeOH, SOCl_2_ (3.0 equiv), rt, 18 h → 50 °C, 18 h, 98%; (d) (*E*)-(2-ethoxyvinyl)­boronic acid pinacol ester (1.3 equiv), Pd­(dppf)_2_Cl_2_ (0.1 equiv), Cs_2_CO_3_ (3.0 equiv), monoglyme/H_2_O, 80 °C, 18 h, 39%; (e) 4 M HCl in 1:1 dioxane/DCM (8.0 equiv), rt, 1 h, quantitative; (f) oxone (1.0 equiv), DMF, rt, 1 h, 61%; (g) *N*-benzyl-1-cyclopropylmethanamine (1.0 equiv), HOBt (1.1 equiv), EDC·HCl (1.3 equiv), Et_3_N (1.5 equiv), DCM, rt, 18 h, 22%; (h) LiOH·H_2_O (1.3 equiv), 1:1:1 MeOH/THF/H_2_O, rt, 2 h, 98%; (i) PyBOP (1.4 equiv), HOBt (1.4 equiv), DIPEA (3.9 equiv), NH_4_Cl (1.9 equiv), DMF, rt, 1 h, 48%; (j) (i) SOCl_2_ (19.6 equiv), 65 °C, 2 h, (ii) NH_4_OH (5.9 equiv), DCM, rt, 5 min, 86% (over two steps); (k) (*E*)-(2-ethoxyvinyl)­boronic acid pinacol ester (1.7 equiv), Pd­(dppf)_2_Cl_2_ (1.0 equiv), Cs_2_CO_3_ (1.2 equiv), monoglyme/H_2_O, 90 °C, 18 h, 89%; (l) 4 M HCl in 1:1 dioxane (5.0 equiv)/DCM, rt, 1 h.

Hybrid **32** was synthesized according to Scheme 2. Benzylation of bis ester **47**, followed by a partially selective monoester hydrolysis/primary amide formation sequence as previously reported^52^ gave intermediate **49**, initially as a mixture of regioisomers (4:1 ratio). The major regioisomer was isolated and a single crystal grown for X-ray analysis, confirming the desired regiochemistry (Scheme 2). Subsequent ester hydrolysis to form **50** and primary amide formation yielded the target compound **32**.

**Scheme 2:**
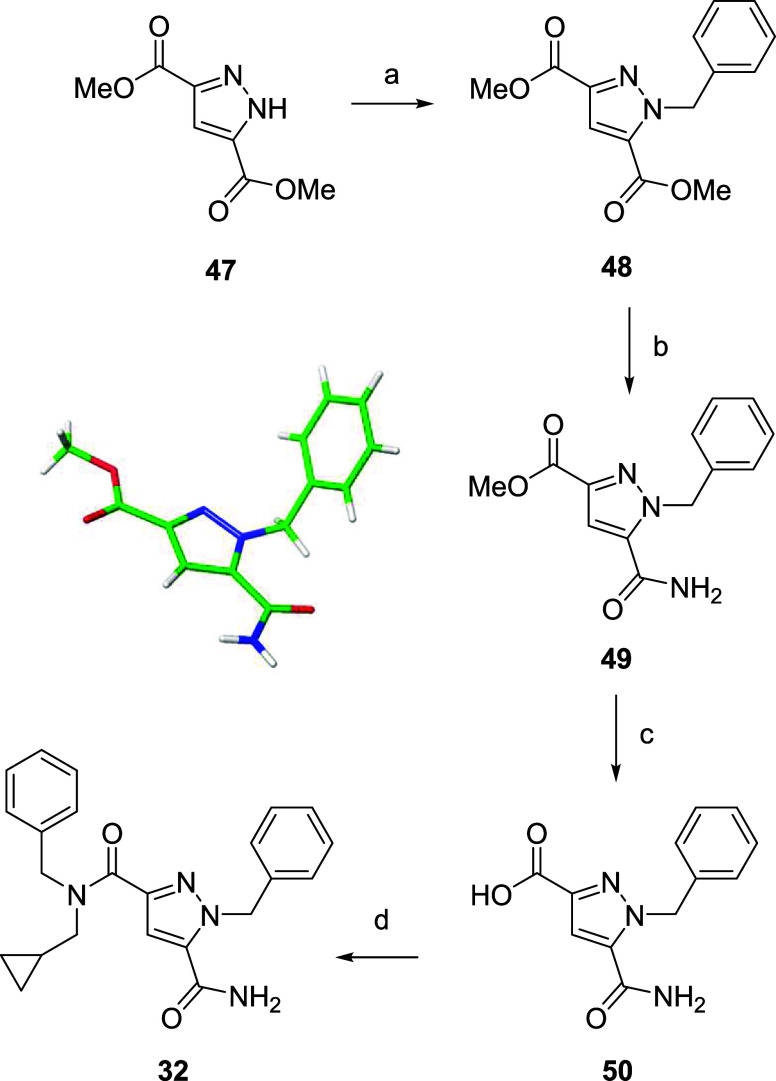
Synthesis of Hybrid 32^a^

a Reagents and conditions: (a) (i) K_2_CO_3_ (1.3 equiv), DMF, 0 °C, (ii) BnBr (1.0 equiv), 0 °C → rt, 1 h, 93%; (b) (i) LiOH·H_2_O (1.0 equiv), 1:1:1 MeOH/THF/H_2_O, 0 °C, 2 h, (ii) SOCl_2_ (21.0 equiv), 65 °C, 2 h, (iii) DCM, NH_4_OH (4.0 equiv), rt, 5 min, 63%; (c) LiOH·H_2_O (3 equiv), 1:1:1 MeOH/THF/H_2_O, rt, 30 min, 80%; (d) (i) SOCl_2_ (34.3 equiv), 65 °C, 2 h, (ii) DCM, Et_3_N (2.5 equiv), *N*-benzyl-1-cyclopropylmethanamine (1.0 equiv), rt, 5 min, 21%.

Hybrid **33** was easily accessible through a one-step amide coupling from the key amine intermediate **51** (Scheme 3), synthesized as previously described.^29^ The final hybrid **34** was synthesized as shown in Scheme 4. Iodination in the presence of ceric ammonium nitrate followed by benzylation gave intermediate **53** in high yield. Suzuki reaction and successful enol ether deprotection gave the key aldehyde intermediate **27** in good yield. Reductive cyclization using 2-methylpyridine-2-borane complex proceeded cleanly, giving the expected two regioisomers **55** and **56** arising from cyclization onto either methyl ester. The mixture of regioisomers were hydrolyzed and amide coupled to afford primary amides **34** and **57** that were isolated in 17% and 9% yields, respectively. Initially, we relied upon the presence or absence of unique HMBC cross-peaks between the benzyl CH_2_, amide NH_2_ and lactam benzyl CH_2_ for confirmation of the regiochemistry. However, we subsequently noted consistent patterns in the ^1^H NMR, specifically differences in the peak position and splitting of the primary amide protons between the two regioisomers (Figure S4). Therefore, regiochemical assignment was possible in the absence of HMBC experiments.

**Scheme 3:**
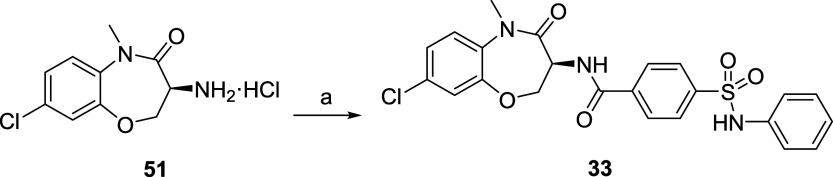
Synthesis of Hybrid 33^a^

a Reagents and conditions: (a) 4-(phenylsulfamoyl)­benzoic acid (1.0 equiv), HOBt (1.1 equiv), EDC·HCl (1.2 equiv), DIPEA (2.6 equiv), DCM, rt, 5 min, 94%.

**Scheme 4:**
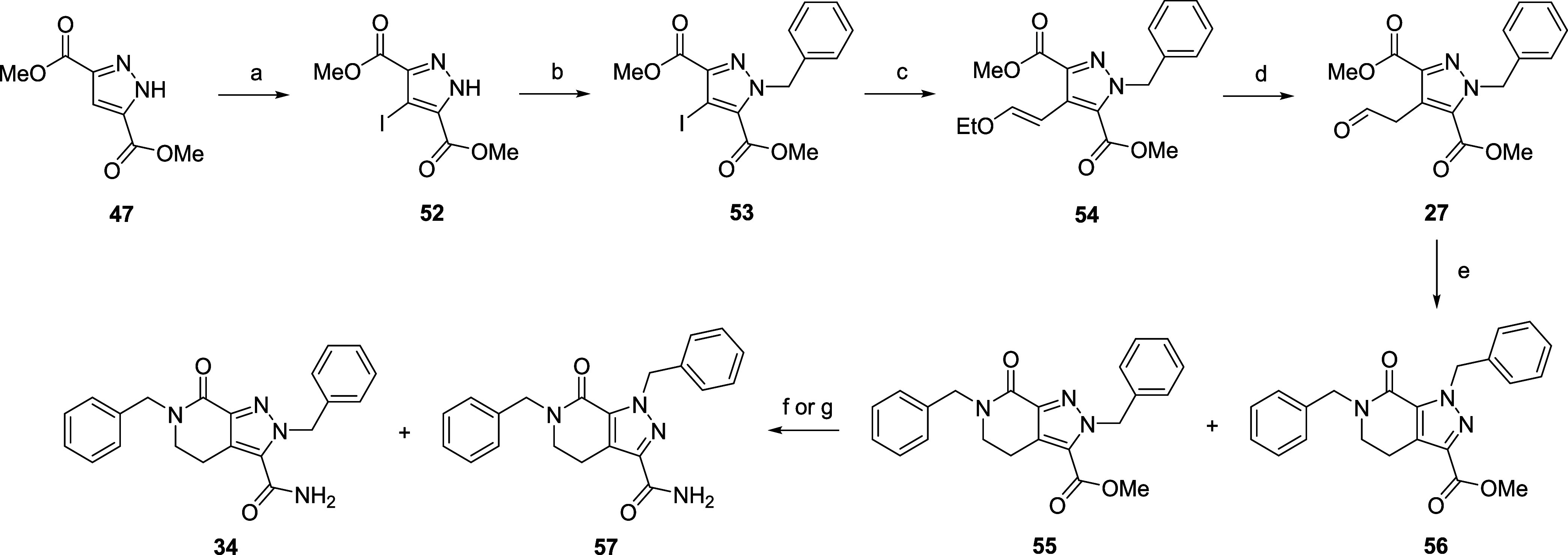
Synthesis of Hybrid 34 and Regioisomer 57^a^

a Reagents and conditions: (a) I_2_ (1.3 equiv), CAN (1.1 equiv), MeCN, rt, 48 h, 89%; (b) (i) K_2_CO_3_ (1.3 equiv), DMF, 0 °C, (ii) BnBr (1.0 equiv), 0 °C → rt, 1 h, 90%; (c) (*E*)-(2-ethoxyvinyl)­boronic acid pinacol ester (1.7 equiv), Pd­(dppf)_2_Cl_2_ (1.0 equiv), Cs_2_CO_3_ (2.1 equiv), monoglyme/water, 90 °C, 48 h, 77%; (d) 6 M HCl in 1:1 dioxane/THF (18.1 equiv), 0 °C → rt, 1 h, 66%; (e) benzylamine·HCl (1.3 equiv), 2-methylpyridine·BH_3_ complex (1.5 equiv), AcOH (0.3 equiv), MeOH, 0 °C, 3 h, 42% (**55**), 22% (**56**); (f) (i) LiOH·H_2_O (1.2 equiv), 1:1:1 MeOH/THF/H_2_O, rt, 18 h, (ii) HOBt (1.1 equiv), EDC·HCl (1.3 equiv), DIPEA (2.4 equiv), NH_4_Cl (3 equiv), DCM, rt, 18 h, 9% (for **34**); (g) 7 M NH_3_ in MeOH (87.5 equiv), 70 °C, 18 h, sealed tube, 17% (for **57**).

All four hybrid targets **31**-**34** and regioisomer **57** were subsequently tested in a range of LIMK1/2 *in vitro* assays (Table 2). LIMK1/2 binding dissociation constants (*K*_d_) were measured using the *K*_d_ELECT platform (provided by Eurofins/DiscoverX) and inhibitory activity assessed using our previously reported RapidFire mass spectrometry assay.^29^ Cellular target engagement and LIMK selectivity was measured using LIMK1 and LIMK2 NanoBRET assays in HEK293 cells, while cellular proof-of-mechanism was assessed by measuring p-cofilin levels in SH-SY5Y cells using the AlphaLISA platform. Since cofilin is a substrate of both LIMK1 and LIMK2, our assay is unable to assess selectivity against these enzymes in contrast to the NanoBRET assay. Hybrids **31** and **32** did not show any inhibition across the various assays and **33** showed only weak inhibition in the LIMK1/2 RapidFire assay (Table 2). We were excited to observe, however, that 1-benzylpyrazolo-5-carboxamide **34** showed potent LIMK1/2 binding and good inhibitory activity in both RapidFire and cellular assays, showing a pIC_50_ value of 6.20 in the AlphaLISA *p*-cofilin assay. On the other hand, its 1-benzylpyrazolo-3-carboxamide regioisomer **57** was inactive in all *in vitro* assays (Table 2). This result was corroborated when we assessed all 1-benzylpyrazolo-3-carboxamides isomers that were isolated during the course of synthesizing the active 5-carboxamides (data not shown).

**Table 2:** Evaluation against LIMK1 and LIMK2 in Binding, Enzymatic and Cellular Target Engagement Assays Values to Explore Structure–Activity Relationship (SAR) of Hybrids 31-34 and Regioisomer 57^a^

	binding affinity (p*K*_d_)^b^		enzymatic pIC_50_		nanoBRET pIC_50_		
compound	LIMK1	LIMK2	LIMK1	LIMK2	LIMK1	LIMK2	alphaLISA pIC_50_
**7**	7.21^c^	7.06^c^	7.17^c^	7.82^c^	6.74 ± 0.15	7.02 ± 0.07	7.06 ± 0.04
**21**	7.73 ± 0.04^d^	7.25 ± 0.04^d^	6.98 ± 0.03^d^	7.95 ± 0.09^d^	6.15^c^	6.29^c^	6.58 ± 0.04
**31**	nd	nd	nd	nd	<5	<5	<5
**32**	nd	nd	<5	<5	<5	<5	<5
**33**	nd	nd	5.01^c^	5.22^c^	<5	<5	<5
**34**	6.80 ± 0.11	7.29 ± 0.05	6.16^c^	7.03^c^	5.77 ± 0.13	6.04 ± 0.01	6.20 ± 0.43^d^
**57**	nd	nd	<5	<5	<5	<5	<5

a Data are reported as mean ± SEM of at least three independent experiments, unless otherwise stated.

b Screened at DiscoverX, Eurofins (San Diego).

c *n* = 1.

d Mean of two independent experiments. nd, not determined.

Based on novel analogue **34**, a series of benzylic substituted analogues and replacements **58**–**72** were synthesized in an effort to increase LIMK1 potency (Schemes 5 and 6). Again, we leveraged SAR knowledge gained from **6** where the cPr moiety increased LIMK1 inhibition by at least 10-fold^39^ and molecular docking studies revealed that the benzylic position was the most suitable position to incorporate this group. Intermediates **73a**–**i** were synthesized using a similar three-step reductive amination, ester hydrolysis and amide coupling route of a benzyl substituted or nonaromatic amine (Scheme 5), as previously discussed. However, we observed the majority of reductive aminated intermediates underwent spontaneous cyclization to form two regioisomers (in approximately 1:1 ratio) upon storage that negated the requirement for a conventional intramolecular amide coupling. Primary amide formation was accomplished by refluxing in methanolic NH_3_ to afford final compounds **58**–**66** (Scheme 5). Hydrolysis product **67** was also isolated as a side product from synthesis of **58**. As expected, sterically hindered amines leading to **68**–**71** and **79** failed to undergo ring cyclization spontaneously, and instead were prepared according to Scheme 6 through a two-step, one-pot amide coupling of dicarboxylic acid **78a**–**c** with the respective amine and NH_4_Cl. Compound **71** was afforded from reductive amination of elaborated amine **76**, itself synthesized through a two-step S_N_Ar and Grignard addition from **74** (Scheme 6). A putative hinge-binding hybrid **72** was also synthesized through Boc deprotection of intermediate **79** and subsequent amide coupling of **80** (Scheme 6), as we noted significant LIMK1 potency improvements when hybridized with our MDI-114215 series (data not shown) in line with previous reports with the Type II kinase inhibitor TH-470.^29,33^

**Scheme 5:**
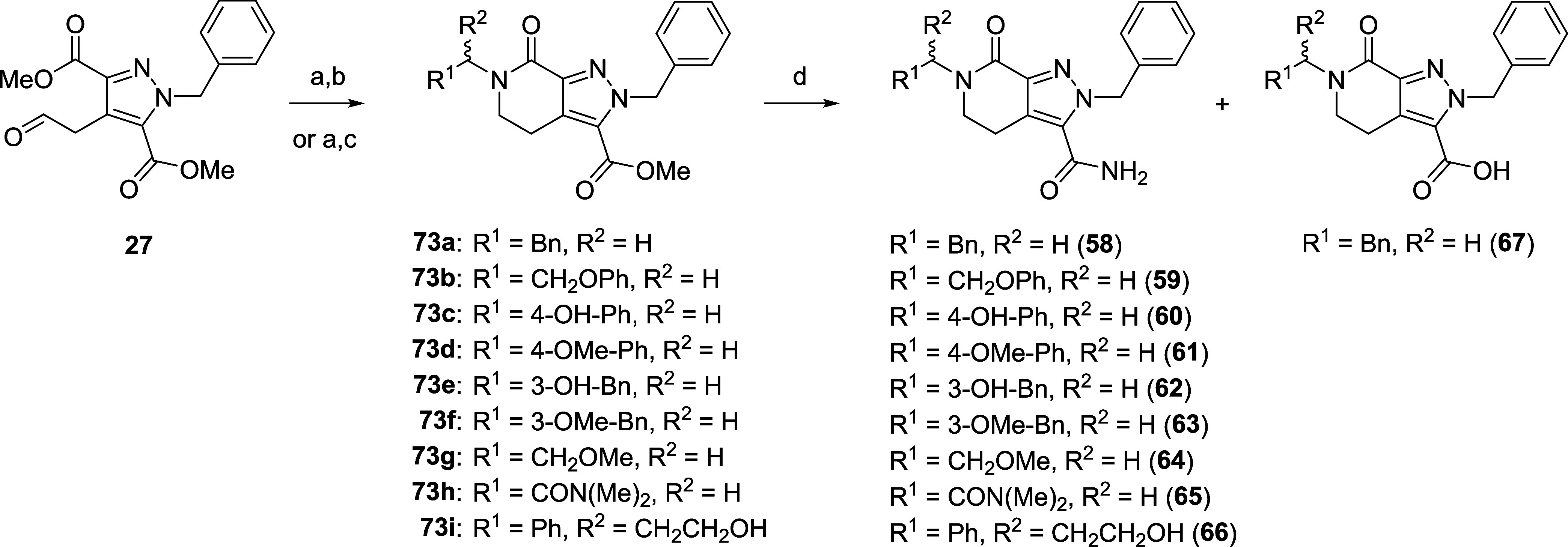
Synthesis of Substituted Tetrahydropyridinone Analogues **58–67** ^a^

a Reagents and conditions: (a) R^1^R ^2^NH_2_·HCl (1.3–1.5 equiv), Pic-BH_3_ (0.9 equiv), AcOH (0.7–2.1 equiv), MeOH, 0 °C, 3 h; 19–61%; (b) R^1^R^2^NH_2_ (1.0–1.5 equiv), STAB (2.5 equiv), AcOH (0.8–2.4 equiv), DCE, rt, 18 h; 13–51% (for **73g** and **73h**); (c) 2 M AlMe_3_ in toluene (3.4 equiv), 100 °C, 3 h, 53% (for **73i**); (d) 7 M NH_3_ in MeOH (32.4–148.9 equiv), 70 °C, 18 h, sealed tube, 6–21%.

**Scheme 6:**
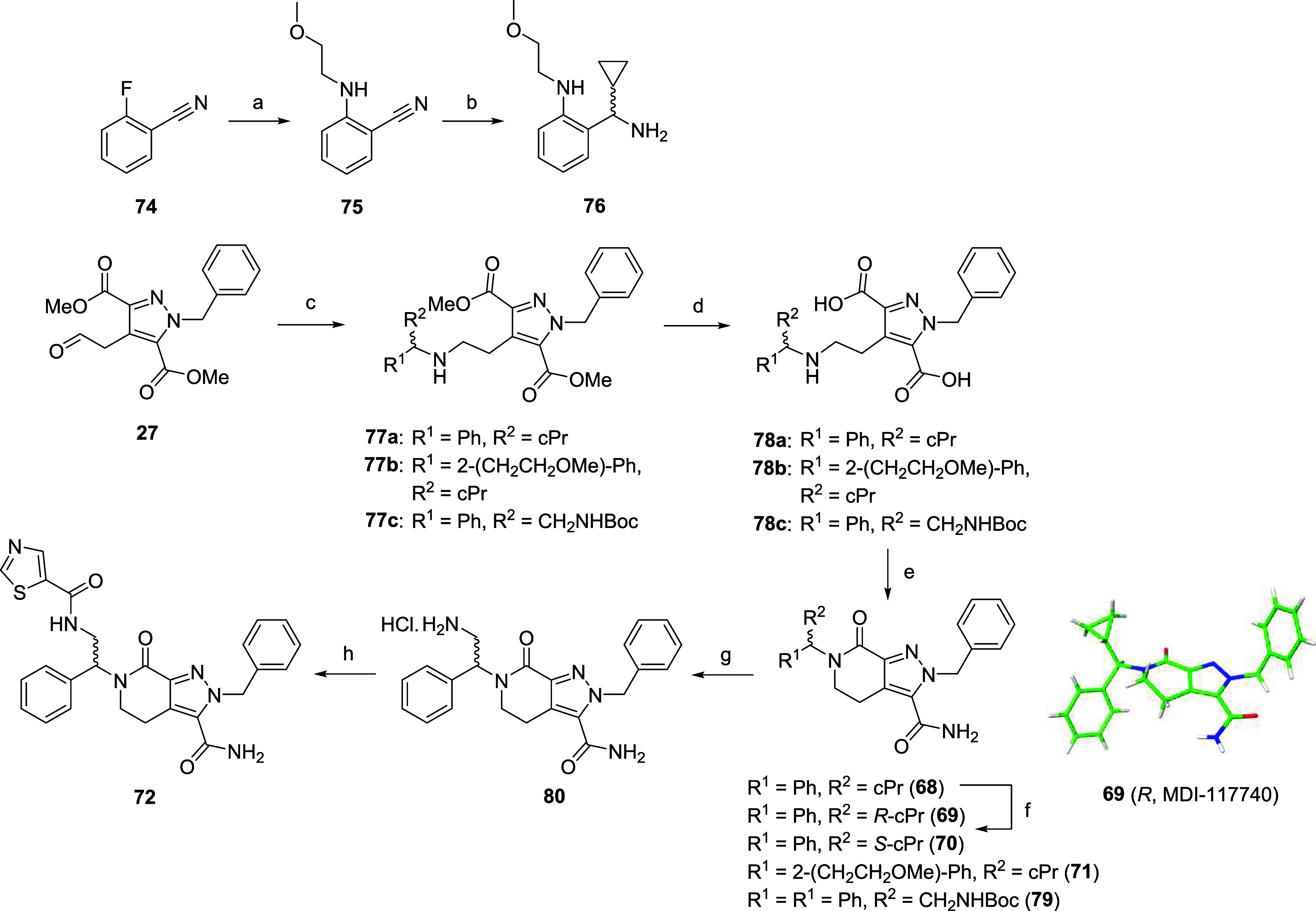
Synthesis of Substituted Tetrahydropyridinone Analogues 68–72^a^

a Reagents and conditions: (a) 2-methoxyethylamine (2.0 equiv), MeCN, 100 °C, 18 h, sealed tube, 34%; (b) cPrMgBr (3.0 equiv), THF, 100 °C, 15 min then NaBH_4_ (5.0 equiv), MeOH, 0 °C, 30 min, 47%; (c) R^1^R^2^NH_2_·HCl (1.0 equiv), Pic-BH_3_ (1.0–1.5 equiv), AcOH (1.1–1.9 equiv), MeOH, 0 °C, 3 h, 22–45%; (d) LiOH·H_2_O (3.0–5.0 equiv), THF/MeOH/H_2_O, rt, 18 h, 78–91%; (e) NH_4_Cl (5.0 equiv), HOBt (1.1 equiv), EDC·HCl (1.3 equiv), DIPEA (2.4 equiv), DCM, rt, 18 h, 5–48%; (f) chiral separation; (g) **79**, 4 M HCl in 1,4-dioxane (33.3 equiv), DCM, rt, 2 h, quantitative; (h) thiazole-5-carboxylic acid (1.0 equiv), HOBt (1.1 equiv), EDC·HCl (1.3 equiv), DIPEA (2.4 equiv), DCM, rt, 18 h, 42%.

The synthesized analogues **58–72** were screened for binding dissociation constants (*K*_d_). Although not a measure of inhibition, we previously observed that apparent *K*_d_ and RapidFire pIC_50_ values were comparable across these *in vitro* assays (see Table 2 and previously described^39^). The LIMK1/2 *K*_d_ binding data are shown in Table 3. The benzyl ring was found to be largely sensitive to substitutions with the most tolerated analogue **60** leading to an approximate 5-fold drop in LIMK1 potency with LIMK2 potency unchanged. Further attempts to extend the benzyl ring position (**62** and **63**) or replace with nonaromatic groups resulted in even greater loss of LIMK1/2 potency (Table 3). Based on a similar binding mode of **7** with LIMK1 compared to the TH-300 (**30**)-LIMK2 structure, we hypothesize that the benzyl ring forms a key π-cation interaction with the protonated K368, similar to other allosteric LIMK inhibitors. We and others have demonstrated the importance of benzyl positioning and aromaticity to maintain this interaction.^33,39,41^ Turning attention to the benzylic position, pleasingly our rationale to incorporate a racemic cPr group increased potency (**68**) and chiral resolution led to a significant potency gain against LIMK1 for the *R*-enantiomer (**69**, Table 3), whose absolute configuration was confirmed by X-ray crystallography (Scheme 6). Various alternative substituents previously reported that project past the gatekeeper residue T316 toward the hinge region did not lead to any noticeable potency gain (such as **66**), including the hinge-binding hybrid **72**.^39^ Attempts to merge compound **69** with **6** conferred nonbinding hybrids such as **71** (Table 3), which was consistent with our benzyl SAR. Therefore, through our SAR assessment, we identified **69** as a novel, highly potent dual LIMK1/2 binder that could be suitable as a tool inhibitor to explore LIMK biology.

**Table 3:**
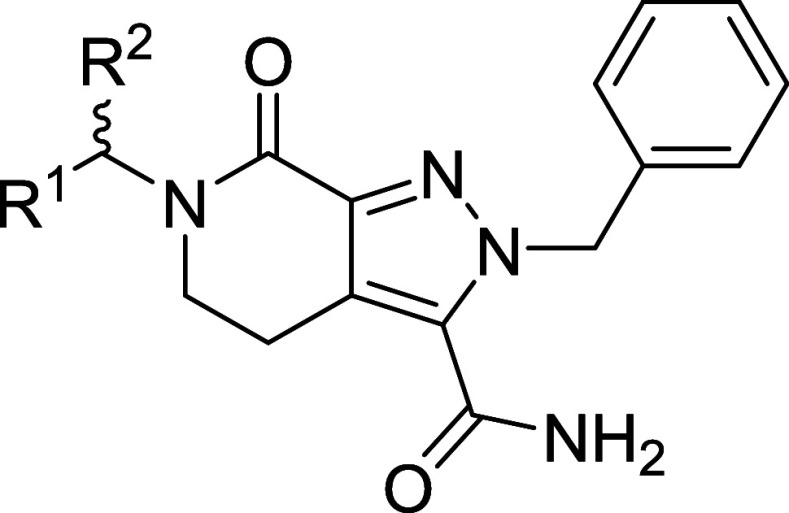
LIMK1 and LIMK2 *K*_d_ Values to Explore Structure–Activity Relationship (SAR) of Substituted Tetrahydropyridinone Analogues 58–72

			binding affinity (p*K*_d_)^a^	
compound	R^1^	R^2^	LIMK1	LIMK2
**34**	Ph	H	6.80 ± 0.11	7.29 ± 0.05
**58**	Bn	H	5.31 ± 0.01	6.29 ± 0.03
**59**	PhOCH_2_	H	5.87 ± 0.09	7.02 ± 0.00
**60**	4-OH-Ph	H	6.08 ± 0.09	7.28 ± 0.05
**61**	4-OMe-Ph	H	5.95 ± 0.23	6.69 ± 0.21
**62**	3-OH-Bn	H	5.31 ± 0.04	5.92 ± 0.08
**63**	3-OMe-Bn	H	<5	6.24 ± 0.23
**64**	CH_2_OMe	H	5.13 ± 0.14	5.90 ± 0.06
**65**	CON(Me)_2_	H	<5	5.28 ± 0.03
**66** ^b^	Ph	CH_2_CH_2_OH	6.73 ± 0.01	6.65 ± 0.07
**67**	see Scheme 5		<5	<5
**68** ^b^	Ph	cPr	7.37 ± 0.11	6.87 ± 0.02
**69**	Ph	*R*-cPr	7.80 ± 0.20	7.12 ± 0.00
**70**	Ph	*S*-cPr	6.87 ± 0.05	6.73 ± 0.12
**71** ^b^	2-((CH_2_)_2_OMe)-Ph	cPr	<5	<5
**72**	see Scheme 6		6.27 ± 0.05	5.92 ± 0.08

a Data are reported as mean ± SEM of at least two independent experiments.

b Racemic.

Modeling of the binding mode of MDI-117740 (**69**) in LIMK1 (PDB: 7ATU) shows good alignment with LIJTF500025 (**7**, Figure 4), maintaining the key hydrogen bonding interactions with H458, D478 and L397 as described for **7** (Figure 5A). However, a difference in binding mode of the benzyl cyclopropylmethylene moiety is predicted which projects the amide substituents of **7** and **69** into different vectors (as shown by magenta and green arrows, respectively, Figure 4). The docked structure of **69** suggests that the *R*-cyclopropyl group pushes into a small hydrophobic pocket enclosed by F479, the alkyl chain of K368, V366 and L367, which occupies the same hydrophobic niche of the *N*-Me group in **7**. Meanwhile, the phenyl ring is presented toward a hydrophobic tunnel comprising L370, L481, F411, and the alkyl chain of K368 (Figure 5). This hydrophobic tunnel is the same as that exploited by MDI-114215 (**6**) with an ethanolamine-substituted aromatic pendant.^39^

**Figure 4:**
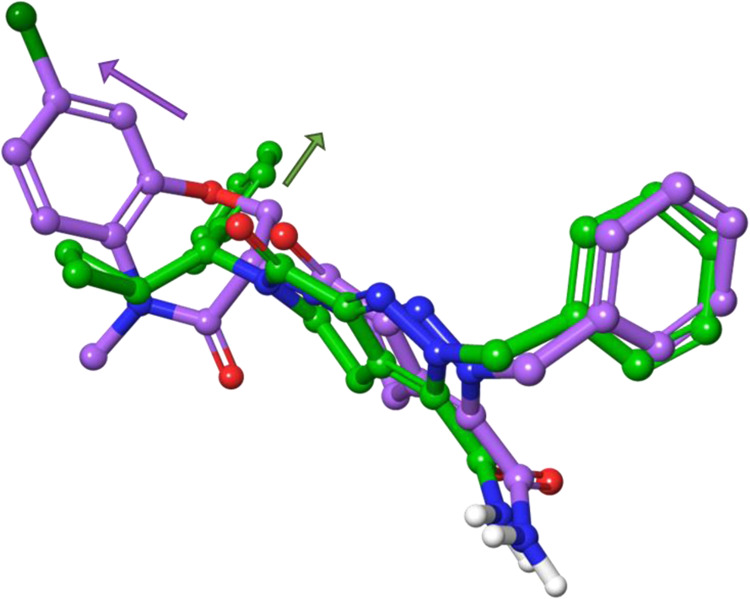
Overlay of modeled **69** (MDI-117740, green) superimposed with **7** (LIJTF500025, magenta) in LIMK1 (PDB: 7ATU, magenta). Ligands were extracted from LIMK1 for clarity.

**Figure 5:**
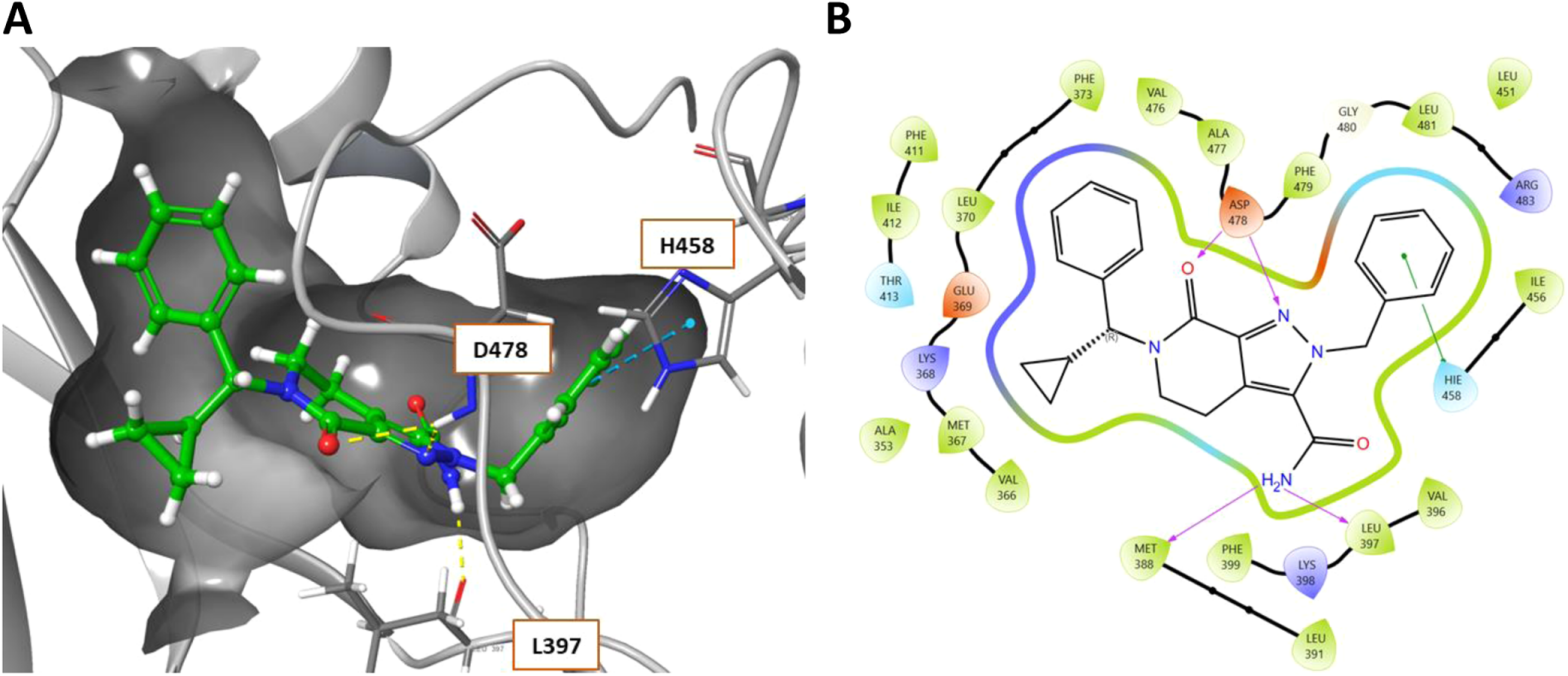
Novel LIMK1 binding mode revealed with **69** (MDI-117740). (A) **69** docked into the **7**-LIMK1 cocrystal structure (PDB: 7ATU). The stereo configuration of the tetrahydropyrazolopyridinone core positions the benzyl cyclopropylmethylene moiety in a different subpocket compared to **7** (LIJTF500025), as detailed in Figure 4. (B) Ligand interaction diagram of **69**. Shading represents the following: hydrophobic area (green), charged interaction (positive, blue; negative, red), polar (teal).

Using novel compound **34** as a template, we also further explored SAR around the primary amide through synthesis of various amide replacements (**81**–**91**), substituted amides (**82**–**88**) and amide isosteres (**89**–**90**, Scheme 7). The cocrystal structure of **7** bound to LIMK1 (PDB: 7ATU) shows a bidentate H-bond interaction between the primary amide and L397/M388 (Figure 2) that we speculated was similarly important for LIMK1 potency on our new scaffold. To confirm this, a small set of secondary and tertiary amides **82**–**88** were synthesized through methyl ester **55** or carboxylic acid **81** that was amenable to various amide coupling conditions (Scheme 7). 1,3,4-Oxadiazole **89** was synthesized from hydrazide **87** and triethylorthoformate under reflux, while partially reduced oxazole **90** was generated from intramolecular cyclization of compound **88** under Mitsunobu conditions (Scheme 7). Attempts to oxidize the semireduced oxazole under various oxidation conditions (*e*.*g*., DDQ) unfortunately failed to furnish the desired oxazole product. Nitrile analogue **91** was synthesized by dehydrating primary amide **34** under the action of TFAA (Scheme 7).

**Scheme 7:**
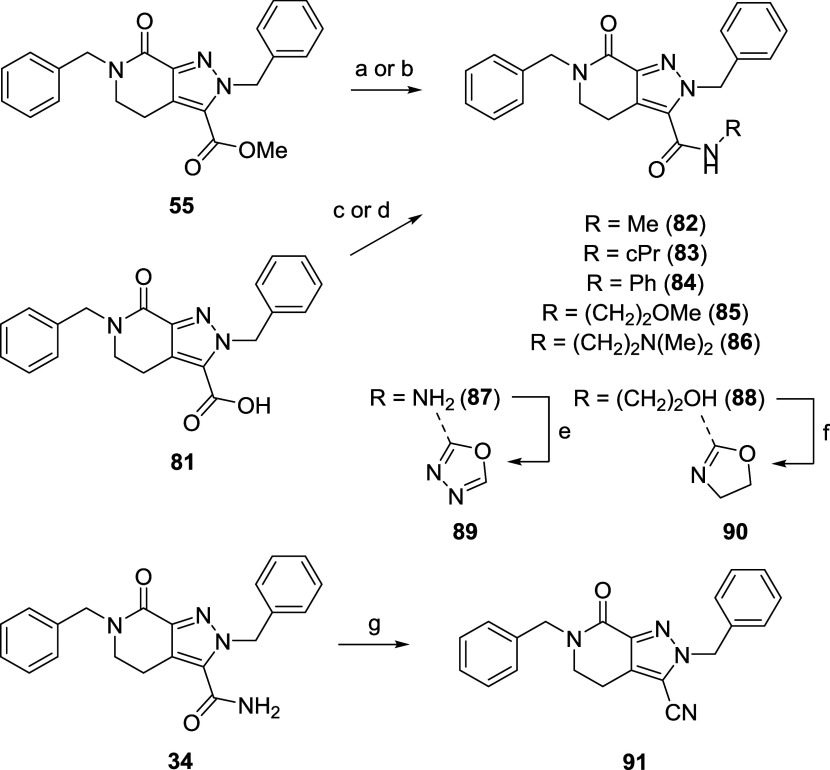
Synthesis of Substituted Amides 82–88, Amide Isosteres 89–90 and Nitrile Analogue 91^a^

a Reagents and conditions: (a) NH_2_NH_2_ (23.6 equiv), EtOH, 90 °C, 6 h, quantitative (when R = NH_2_); (b) NH_2_(CH_2_)_2_OH, DIPEA, MeOH, 130 °C, 16 h, 86% (when R = (CH_2_)_2_OH); (c) RNH_2_ (1.5 equiv), T3P (3.0 equiv), DIPEA (2.6 equiv), THF, rt, 18 h, 5–35%; (d) RNH_2_ (1.3–1.5 equiv), HOBt (1.1 equiv), EDC·HCl (1.3 equiv), DIPEA (2.5 equiv), DCM, rt, 18 h, 15–18%; (e) HC­(OEt)_3_ (37.6 equiv), 100 °C, 18 h, 7%; (f) DEAD (1.5 equiv), PPh_3_ (1.5 equiv), toluene, rt, 18 h, 4%; (g) TFAA (5.3 equiv), Et_3_N (7.5 equiv), DCM, rt, 18 h, 40%.

The synthesized substituted amides and primary amide replacements were then screened against LIMK1 and LIMK2 in the *K*_d_ELECT assay. The results unequivocally demonstrated that the primary amide was indispensable for LIMK1/2 binding (Table 4). The majority of compounds were found to be inactive with only weak binding affinities recorded for nitrile analogue **91** (Table 4). The strong requirement for an unsubstituted primary amide is consistent with both (i) **7**-LIMK1 structure which forms key H-bond interactions with the protein through each amidic NH, and (ii) overlay of **7** with the TH-300 (**30**)-LIMK2 structure (PDB: 5NXD) that superposes the primary amide with the sulfonamide (Figure 3A), a group which also could not be substituted or replaced.^39^ Indeed, similar to the primary amide-sulfonamide comparison, the benzyl ring of **7** superimposes very well with the aniline ring of **30** (Figure 3A), which we and others showed does not tolerate any modification.^39,41^ Therefore, we regard this region where type III allosteric inhibitors **6** and **7** bind deep into the protein pocket as inflexible and their unique space filling properties and H-bond interaction network as pivotal to LIMK1/2 binding or inhibition. As a result, we decided against further exploring modifications to the benzyl ring.

**Table 4:**
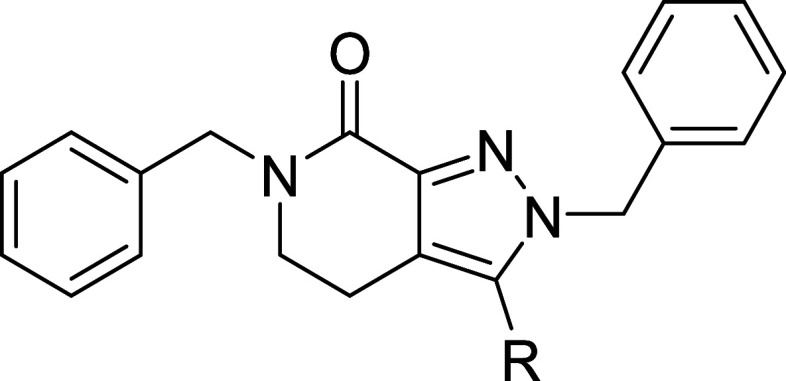

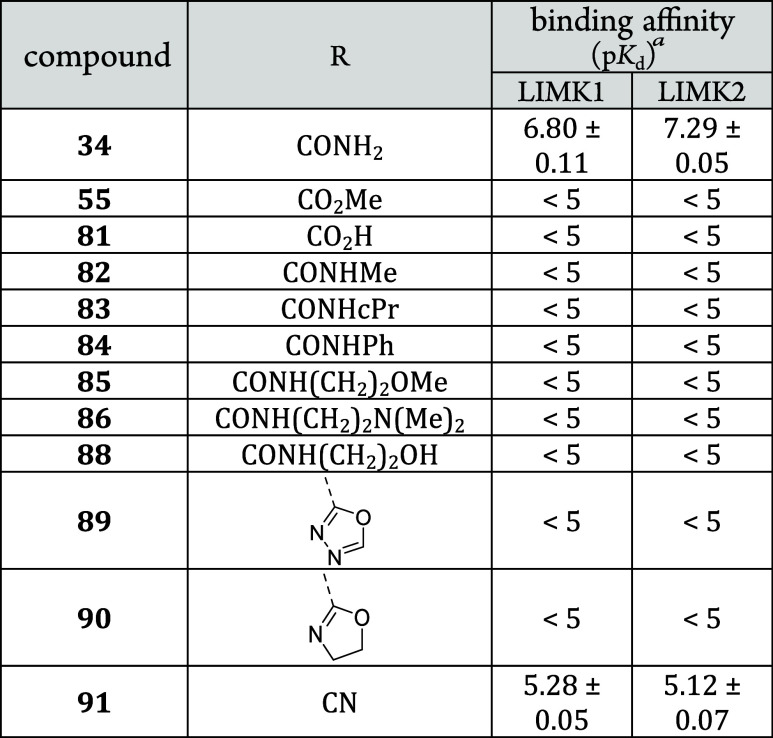
LIMK1 and LIMK2 *K*_d_ Values to Explore Structure–Activity Relationship (SAR) of the Primary Amide of Lead Compound 34

a Data are reported as mean ± SEM of at least two independent experiments.

The most optimized molecule **69** was further evaluated for its suitability as an *in vitro* and *in vivo* tool by assessing cellular activity and DMPK properties (Table 5). **69** displayed excellent cellular target engagement and potently inhibits p-cofilin levels in SH-SY5Y cells. The *in vitro* DMPK properties of **69** were also significantly improved (Table 5) compared to **6** (aqueous solubility = 11 μM, rat/human microsomal CL_int_ = 289/98 μL/min/mg), a type III allosteric dual LIMK1/2 inhibitor that we recently disclosed where optimizing drug clearance was challenging.^39^ Given its promising *in vitro* DMPK profile, we then assessed ADME properties of **69** through *in vivo* PK evaluation. The low *in vitro* microsomal clearance translated to low *in vivo* CL_int_ in male Sprague–Dawley rats and, again, was improved compared to **6** (CL_int_ = 33 mL/min/kg). This in turn led to enhanced drug exposure for **69** (654 nM), achieving >40-fold and >8-fold greater total drug concentration than LIMK1 and LIMK2 *K*_d_, respectively. Other key parameters (*V*_D_, *T*_1/2_) were also acceptable after i.v. dosing (Table 5). Unfortunately, oral bioavailability of **69** was very low with maximal drug concentrations (*C*_max_) in plasma reaching approximately 27 ng/mL after p.o. dosing (at 3 mg/kg) and no evidence of good CNS penetration was observed after i.p. dosing (at 10 mg/kg, Table 5). The limited CNS penetration of **69**, similar to other type III allosteric LIMK inhibitors, represents a limitation for studies exploring the role of LIMKs in neuronal function or synaptic regulation. Therefore, we suspected that **69** was a drug efflux substrate given the effect of different routes of administration on the *in vivo* PK profile.

**Table 5:** *In Vitro* and *In Vivo* DMPK Properties of **69**

	69 (MDI-117740)
nanoBRET LIMK1/2 pIC_50_ ^a^	6.73 ± 0.09/7.18 ± 0.18
alphaLISA *p*-cofilin pIC_50_ ^a^	6.89 ± 0.02
aq. solubility (μM, pH 7.0)^b^	85
R/H^c^ microsomal CL (μL/min/mg)	96/48
i.v. dose (mg/kg)	0.2
AUC_inf_ (h·ng/mL)	262
CL_int_ (mL/min/kg)	13
*V*_D_ (L/kg)	0.5
*T*_1/2_ (h)	0.6
*C*_max_ (ng/mL)^d^	26.7
*T*_max_ (h)^d^	0.4
F%^e^	2
B/P ratio^f^	0.02

a Data are reported as mean ± SEM of at least three independent experiments.

b Average of at least two separate experiments.

c Rat and human microsomes.

d Determined after p.o. dosing at 3 mg/kg.

e Determined by comparison with 3 mg/kg dosing p.o.

f Determined internally after i.p. dosing at 10 mg/kg.

We carried out a structure-permeability relationship study to prove **69** was a drug efflux substrate and identify the chemical motifs responsible for poor permeability. Compounds were assessed in the Caco-2 permeability assay as it is widely used as a model of the intestinal epithelial barrier and thus predicts human intestinal permeability. The results are presented in Table 6. In summary, we confirmed that **69** was a drug efflux substrate (ER = 5.5). Structure-permeability relationships also clearly showed that 1-benzylpyrazolo-5-carboxamides were exclusively subject to high efflux (ER > 2), whereas 1-benzylpyrazolo-3-carboxamides (*e*.*g*. **57**), primary amide isosteres/replacements (*e*.*g*., **89**) or ring-opened analogue **32** demonstrated low ER (Table 6). Thus, the privileged 1-benzylpyrazolo-5-carboxamide scaffold necessary for potent dual LIMK1/2 inhibition also leads to high drug efflux precluding oral dosing (and most likely low CNS penetration) that could not be separated, presenting significant challenges in developing a highly potent, selective, CNS-penetrant series with good oral bioavailability.

**Table 6:** Caco-2 Permeabilities and Efflux Ratios of Selected Compounds

	caco-2 permeability^a^		
compound	*P*_app(A–B)_ (×10^–6^ cm/s)	ER^b^	mean % recovery (A–B/B–A)
antipyrine^c^	44.1 ± 1.59	0.95	86.6/92.7
atenolol^d^	0.28 ± 0.02	1.94	87.8/93.8
talinolol^e^	0.31 ± 0.07	37.9	79.1/92.0
estrone 3-sulfate^f^	0.45 ± 0.06	52.3	61.4/84.4
**7**	5.11 ± 0.51	13.9	85.9/74.2
**19** ^g^	6.84 ± 0.16	1.20	36.5/25.9
**21**	3.06 ± 0.08	14.6	91.2/85.3
**32** ^h^	18.8 ± 1.89	2.00	40.5/66.2
**34**	7.00 ± 0.74	6.79	85.4/85.6
**55**	40.6 ± 0.95	0.85	72.3/63.2
**57**	28.3 ± 1.22	0.96	63.2/66.5
**59**	10.0 ± 0.52	5.54	82.9/81.5
**64**	1.93 ± 0.30	11.0	87.1/96.1
**66** ^i^	0.38 ± 0.17	86.9	84.2/84.3
**68**	8.29 ± 0.44	6.32	77.2/65.2
**69**	10.1 ± 0.07	5.52	81.5/82.2
**82**	12.8 ± 0.53	4.29	76.1/85.7
**89**	44.5 ± 1.86	1.09	84.4/85.6
**90**	38.5 ± 2.84	1.04	69.8/71.0
**91**	35.0 ± 0.22	0.97	72.3/64.3

a Data are reported as mean ± SD of at least two independent experiments.

b Efflux is calculated as *P*_app(A–B)_/*P*_app(B–A)_.

c Human absorption = 97%.

d Human absorption = 50%.

e Known P-gp substrate.

f Known BCRP substrate.

g High *C*_0_ value A–B direction and low recovery B–A direction.

h Low *C*_0_ value B–A direction was observed.

i Compound concentration from basolateral compartment below limit of quantification.

As exquisite kinome selectivity is an essential requirement for developing a LIMK selective tool compound, we assessed the wider kinome selectivity of **69** using the Eurofins/DiscoverX scanMAX panel of 468 kinases (which employs the KINOMEscan Technology, further details at https://www.eurofinsdiscovery.com/solution/kinomescan-technology, full kinome screening data are reported in Table S1). We chose to screen at a single concentration of 300 nM as approximately 10-fold LIMK1 *K*_d_ (Table 3) and a selectivity score, S(35)-score, was calculated by dividing the number of kinases that bind by >35% with the total number of kinases tested in the panel. Smaller scores indicate higher selectivity and was used to facilitate comparison with reported literature LIMK inhibitors. The selectivity screen revealed an S(35)-score of 0.005 (Figure 6), making **69** the most selective dual LIMK1/2 inhibitor reported to date.^29,33^ Importantly, no binding to RIPK1 was observed, a known off-target of **7**.^42,43^ In addition, **69** does not bind PAK1–2/4, ROCK1/2, MRCKα or CAMK4 that are known to activate LIMK1/2 through phosphorylation.^4,6,8,53^ Such a selective LIMK inhibitor is critical to prevent assay misinterpretation for researchers wanting to investigate LIMKs in health and disease, similar to previous observations with the now known dual LIMK/PAK inhibitor FRAX486.^29,34^

**Figure 6:**
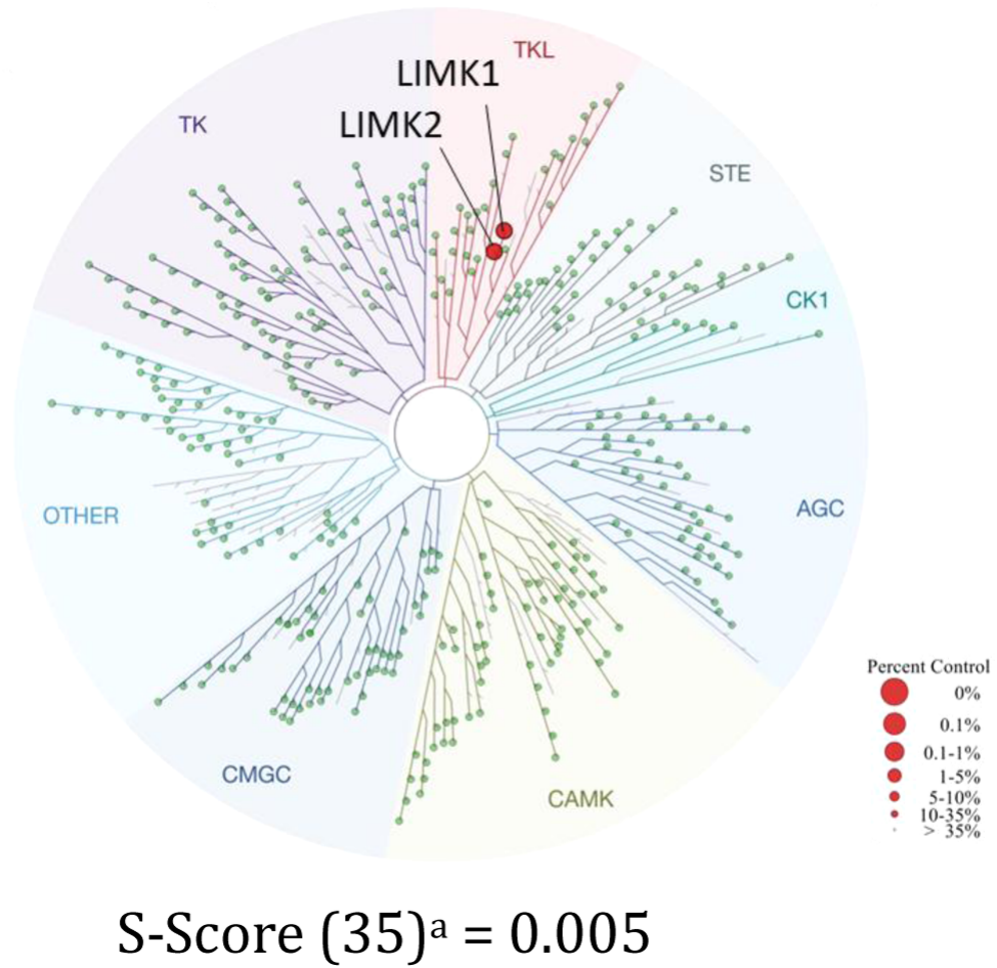
Kinome selectivity profiling of MDI-117740 (**69**). Selectivity profile using the scanMAX kinome wide selectivity assay (Eurofins), screened at 300 nM. Data are illustrated using the TREE_spot_ interaction map (DiscoverX). ^a^Number of nonmutant kinases that **69** binds to with %Ctrl <35 divided by the total number of distinct kinases tested.

To assess the functional impact of **69** on cell motility, an *in vitro* wound healing assay was performed in MDA-MB-231 cells for 48 h. A scratch was induced to confluent monolayers and cells were treated with **69**, FRAX486 (**2**) or a structurally related negative control (**57**, enzymatic LIMK1/2 pIC_50_ < 5, Table 2) at three separate concentrations (0.25 μM, 1 μM, and 3 μM) or untreated control. **2** was selected as the positive control as it was previously shown to significantly inhibit TNBC MDA-MB-231 cell migration in wound healing assays.^54^ Wound closure was measured over 48 h and the percentage wound closure was quantified relative to the initial wound area. Treatment with compound **69** significantly inhibited wound closure compared to untreated control at 0.25 μM and 1 μM concentrations (*p* = 0.049 and *p* = 0.019, respectively) and had a more pronounced effect observed at 3 μM (*p* = 0.006, Figure 7A). LIMK inhibitor **2** also induced significant effects on cell migration (0.25 μM *p* = 0.015, 1 μM *p* = 0.002 and 3 μM *p* = 0.001, Figure 7B), consistent with previous literature.^54^ No significant effect was observed for **57** (*p* = 0.46, Figure 7A/B). It should be noted that cells treated at higher concentrations of compound **2** appear more rounded which may indicate mitotic arrest, a phenotype not observed upon treatment with compounds **57** or **69**. Given that **2** has broader kinome promiscuity than **69**,^29^ this suggests that **2** might act on additional targets involved in cell division beyond LIMK1 or LIMK2. Further cell cycle analysis is warranted to confirm these observations. Taken together, these data strongly suggest that **69** is a potent and highly selective dual-LIMK1/2 inhibitor that demonstrates cellular target engagement, potent inhibition of cofilin phosphorylation, and positive LIMK-dependent downstream phenotypic effects on cell migration.

**Figure 7:**
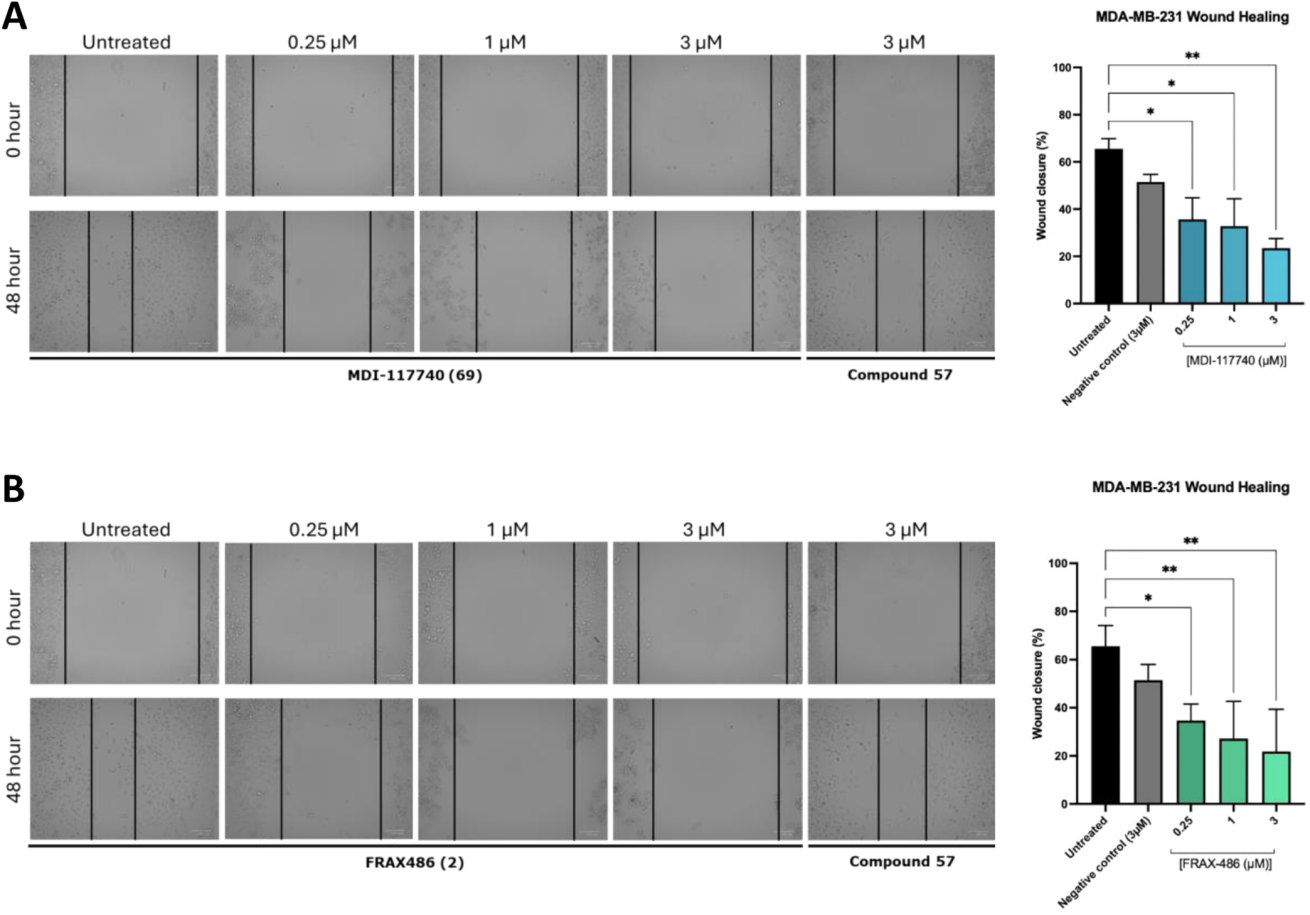
Effect of MDI-117740 (**69**) and FRAX486 (**2**) on cell migration in a wound healing assay in MDA-MB-231 cells. Confluent monolayers were scratched and treated with either compound **69** (A) or **2** (B) at 0.25, 1, or 3 μM. Wound closure was assessed after 48 h. Representative images and quantification of wound area are shown. Scale bar: 100 μm. Compound **69** inhibited wound closure at all tested concentrations in this experiment. Data are from four independent experiments.

## Conclusions

In summary, we have discovered and characterized tetrahydropyrazolopyridinones as a novel class of LIMK1/2 inhibitors. Structure-based drug design using the Takeda RIPK1 inhibitor **7**-LIMK1 structure, together with our prior understanding of similar type III allosteric inhibitors, led to discovery of MDI-117740 (**69**) as a potent LIMK1/2 inhibitor that demonstrates good *in vivo* PK properties suitable as both an *in vitro* and *in vivo* probe when dosed intravenously. A structure-permeability relationships study confirmed that the indispensable 1-benzylpyrazolo-5-carboxamide scaffold is responsible for low oral bioavailability in this series due to high drug efflux. Importantly, we show that **69** is the most selective LIMK1/2 inhibitor currently available in the literature, eliminating any pre-existing RIPK1 activity associated with **7**. The highly selective and potent dual LIMK1/2 inhibitor **69** described herein will facilitate understanding of the mechanistic roles of LIMK in disease and their application toward therapies targeting LIMK pathologies.

## Experimental Section

### General Methods

All commercial materials were used as received without further purification. Identity and purity checks were carried out prior to use in biological experiments using ^1^H NMR spectroscopy and UPLC-MS analysis on the instruments as described below that are presented in the Supporting Information for reference. All final compounds were ≥95% purity as determined by ^1^H NMR and/or UPLC-MS analyses.

### Materials

LIJTF500125 (**7**), (3*S*)-3-amino-8-chloro-5-methyl-2,3-dihydro-1,5-benzoxazepin-4-one (as the hydrochloride or trifluoroacetate salt) and 4-(phenylsulfamoyl)­benzoic acid were prepared as previously described.^29^ *N*-benzyl-1-cyclopropylmethanamine was synthesized as previously described.^39^

### Synthetic Procedures and Compound Characterization

An example stacked ^1^H NMR spectrum permitting regiochemical assignment of 1-benzylpyrazolo-carboxamides in the absence of HMBC experiments can be found in Figure S4. ^1^H and ^13^C NMR spectra were recorded on a Bruker Avance III HD 500 or 400 MHz equipped with a Prodigy cryoprobe. Chemical shifts (δ) are defined in parts per million (ppm). ^1^H NMR spectra were referenced to tetramethylsilane (TMS, δ = 0.00 ppm) or residual undeuterated solvent (CDCl_3_, δ = 7.26 ppm; MeOH-*d*_4_, δ = 3.31 ppm; DMSO-*d*_6_, δ = 2.50 ppm). ^13^C NMR spectra were referenced to undeuterated solvent (CDCl_3_, δ = 77.16 ppm; DMSO-*d*_6_, δ = 39.52 ppm). Multiplicities are abbreviated as follows: s, singlet; d, doublet; t, triplet; q, quartet; dd, doublet of doublets; dt, doublet of triplets; m, multiplet; br, broad, or combinations thereof. Coupling constants were measured in Hertz (Hz). Liquid chromatography–mass spectrometry (LCMS) was primarily carried out on a Waters Acquity Hclass plus UPLC coupled to a Waters Acquity HPLC PDA detector and a Waters Acquity QDa API-ES mass detector. Samples were eluted through a BEH C_18_ 2.1 mm × 50 mm, 1.7 μm column using H_2_O and MeCN acidified by 0.1% formic acid. The gradient runs H_2_O/MeCN/formic acid at 90:10:0.1–10:90:0.1 for 3 min at 1.5 mL/min and detected at 254 nm. LCMS for key intermediates were also carried out on either a: (i) Waters HPLC equipped with XSelect CSH C_18_ 3.0 mm × 50 mm × 2.5 μm column with samples eluting using a gradient of 95:5–1:49 2.5 mM (NH_4_)­HCO_3_/MeCN for 5 min at 1 mL/min, or (ii) Waters HPLC equipped with XBridge CSH C_18_ 3.0 mm × 50 mm × 2.5 μm with sample eluting using a gradient of 95:5–1:49 H_2_O/MeCN acidified by 0.05% formic acid for 4 min at 1.2 mL/min, or (iii) LCMS EW21065–214-P1B1, or (iv) Shimadzu LC-20AB & MS 2020 with HALO C_18_ 3 mm × 30 mm, 5 uM using 0.04% TFA in water and 0.02% TFA in MeCN at a flow rate of 2.0 mL/min. Accurate mass determination was performed on a Waters Synapt G2 SI with ESI probe. Molecular ion peaks are defined as mass/charge (*m*/*z*) ratios. Melting point analysis was carried out using an Electrothermal IA9100 digital melting point apparatus. Analytical thin-layer chromatography (TLC) was performed using VWR silica gel 60 on aluminum plates coated with F_254_ indicator. All spots were visualized with ultraviolet light using a UVP C-10 Chromato-Vue cabinet or stained using KMnO_4_. Normal-phase purifications were completed using a Teledyne ISCO CombiFlash NEXTGEN 300+ using silica gel with particle size 40–63 μm; reverse-phase purifications were completed using a Teledyne ACCQPrep system equipped with a 20 mm × 150 mm C_18_ column, or Waters system equipped with a 30 mm × 250 mm C_18_ column, and eluted with a 10–100% MeOH/H_2_O, 10–100% MeCN/0.1% formic acid in H_2_O, or MeCN/5 mM (NH_4_)­HCO_3_ gradient. Evaporation of solvents was conducted on a Buchi Rotavapor R-300.

#### General Procedure A–Reductive Amination

Unless otherwise stated, a suspension of a primary or secondary amine (1.1–1.5 equiv) and dimethyl 1-benzyl-4-(2-oxoethyl)-1*H*-pyrazole-3,5-dicarboxylate (**27**, 1 equiv) in MeOH (5–10 mL) was added acetic acid (0.1–0.3 mL, catalytic). The reaction mixture was cooled to 0 °C and stirred for 5 min until a solution resulted. 2-Methylpyridine borane complex (0.9–1.5 equiv) was then added and the resultant solution stirred at 0 °C for 3 h, allowing to warm to room temperature. Saturated NaHCO_3_ (5–10 mL) was added and the mixture extracted with EtOAc (2 × 10–20 mL). The organic layers were combined, dried over NaSO_4_, filtered and concentrated under reduced pressure. The crude mixture was purified by flash column chromatography and fractions containing product were combined and concentrated under reduced pressure to afford the reductive aminated product.

#### General Procedure B–Ester Hydrolysis

Unless otherwise stated, a solution of a methyl 7-oxo-4,5,6,7-tetrahydropyrazolo­[3,4-*c*]­pyridine carboxylate in either THF/water (1:1) or MeOH/THF/water (1:1:1) was added LiOH·H_2_O (3–5 equiv). The reaction mixture was stirred at room temperature overnight. The reaction mixture was concentrated under reduced pressure. One M HCl (∼5 mL) was added and a solid immediately precipitated. The precipitate was filtered, washed with water (10 mL) and dried thoroughly to afford the 7-oxo-4,5,6,7-tetrahydropyrazolo­[3,4-*c*]­pyridine carboxylic acid.

#### General Procedure C–Amide Coupling

Unless otherwise stated, DIPEA (1.5–2.4 equiv) was added to a solution of a 7-oxo-4,5,6,7-tetrahydropyrazolo­[3,4-*c*]­pyridine carboxylic acid (1 equiv), HOBt hydrate (1.1 equiv), EDC·HCl (1.2–1.3 equiv) in DCM (2–8 mL). The reaction mixture was stirred at room temperature for 30 min. A (un)­substituted amine (1–5 equiv) was then added. The reaction mixture was further stirred at room temperature overnight. Saturated NaHCO_3_ (10 mL) was added and the reaction mixture stirred for 15 min at room temperature. The organic phase was separated using a phase separator and the filtrate concentrated under reduced pressure with silica. The crude mixture was purified by flash column chromatography and fractions containing product were combined and concentrated under reduced pressure to afford the substituted 7-oxo-4,5,6,7-tetrahydropyrazolo­[3,4-*c*]­pyridine carboxamide.

#### General Procedure D–Primary Amide Synthesis

Unless otherwise stated, a methyl 7-oxo-4,5,6,7-tetrahydro-2*H*-pyrazolo­[3,4-*c*]­pyridine carboxylate (1 equiv) was dissolved in 7 M NH_3_ in MeOH (5–15 mL). The reaction mixture heated to 70 °C and stirred overnight in a sealed tube. The reaction mixture was concentrated under reduced pressure. The crude mixture was purified by reverse-phase column chromatography and fractions containing product were combined and concentrated under reduced pressure to afford the primary 7-oxo-4,5,6,7-tetrahydro-2*H*-pyrazolo­[3,4-*c*]­pyridine carboxamide.

#### Ethyl 1-Benzyl-5-bromo-4-formyl-1*H*-pyrazole-3-carboxylate (**14**)

To the solution of ethyl 1-benzyl-5-hydroxy-1*H*-pyrazole-3-carboxylate (3.00 g, 12.1 mmol, 1.00 equiv) and POBr_3_ (30.8 g, 107 mmol, 8.83 equiv) in DCE (45 mL) was added DMF (1.66 g, 22.7 mmol, 1.87 equiv) at room temperature under N_2_. Upon completion of addition, the mixture was heated to 90 °C and stirred for 72 h. The reaction mixture was poured carefully onto ice water (500 mL), then stirred at room temperature for 30 min. The mixture was extracted with EtOAc (500 mL), and the organic layer washed with brine (500 mL), dried over Na_2_SO_4_, filtered and concentrated under reduced pressure to afford the crude product, which was purified by column chromatography (silica, 99:1 petrol/EtOAc to 9:1 petrol/EtOAc) to afford ethyl 1-benzyl-5-bromo-4-formyl-1*H*-pyrazole-3-carboxylate (**14**, 1.50 g, 4.44 mmol, 37% yield) as a yellow solid. ^1^H NMR (400 MHz, DMSO-*d*_6_) δ 10.24 (s, 1H), 7.48–7.15 (m, 5H), 5.57 (s, 2H), 4.36 (q, *J* = 7.1 Hz, 2H), 1.32 (t, *J* = 7.1 Hz, 3H). LCMS: EW21065–214-P1B1: Rt = 0.88 min; *m*/*z* 337.1 [M + H, ^79^Br]^+^.

#### Ethyl 1-Benzyl-5-bromo-4-(2-methoxyvinyl)-1*H*-pyrazole-3-carboxylate (**15**)

To a solution of (methoxymethyl)­triphenylphosphonium chloride (10.9 g, 32.0 mmol, 2.00 equiv) in THF (50 mL) was added *t*-BuOK (3.59 g, 31.9 mmol, 2.00 equiv) at 0 °C under N_2_. The reaction mixture was stirred at 0 °C for 5 min, before a solution of ethyl 1-benzyl-5-bromo-4-formyl-1*H*-pyrazole-3-carboxylate (5.40 g, 16.0 mmol, 1.00 equiv) in THF (100 mL) was added. The mixture was stirred at room temperature for 12 h. The reaction mixture was poured onto ice water (200 mL), then extracted with EtOAc (100 mL). The organic layer was washed with brine (100 mL), dried over Na_2_SO_4_, filtered and concentrated under reduced pressure to afford the crude product, which was purified by column chromatography (silica, 49:1 petrol/EtOAc to 9:1 petrol/EtOAc) to afford ethyl 1-benzyl-5-bromo-4-(2-methoxyvinyl)-1*H*-pyrazole-3-carboxylate (**15**, 5.02 g, 13.5 mmol, 61% yield) as a mixture of diastereomers (83% *trans* by coupling constant observed at 6.02 ppm compared to 6.16 ppm for minor *cis* isomer) as a light yellow solid. Major isomer (*trans*) reported: ^1^H NMR (500 MHz, CDCl_3_) δ 7.35–7.26 (m, 4H), 7.22–7.19 (m, 2H), 6.02 (d, *J* = 13.3 Hz, 1H), 5.47 (s, 2H), 4.42 (q, *J* = 7.1 Hz, 2H), 3.69 (s, 3H), 1.41 (t, *J* = 7.2 Hz, 3H). ACQUITY UPLC BEH C_18_ 1.7 μm: Rt = 1.85 min; *m*/*z* 387.0 [M + Na, ^79^Br]^+^, 389.0 [M + Na ^81^Br]^+^.

#### Ethyl 1-Benzyl-5-bromo-4-(2-oxoethyl)-1*H*-pyrazole-3-carboxylate (**16**)

Ethyl 1-benzyl-5-bromo-4-[(*E*)-2-methoxyvinyl]­pyrazole-3-carboxylate (131 mg, 0.34 mmol) was dissolved in 4 M HCl in 1,4-dioxane (1.2 mL). UPLC analysis after 5 min indicated reaction was complete. The mixture was taken to pH 9 by addition of a saturated aqueous solution of NaHCO_3_ (10 mL) and extracted with EtOAc (2 × 30 mL). The organic phase was dried over MgSO_4_, filtered and concentrated under reduced pressure to give ethyl 1-benzyl-5-bromo-4-(2-oxoethyl)­pyrazole-3-carboxylate (**16**, 106 mg, 0.287 mmol, 88% yield) as a colorless oil. ^1^H NMR (500 MHz, CDCl_3_) δ 9.67 (t, *J* = 1.4 Hz, 1H), 7.35–7.28 (m, 3H), 7.25–7.21 (m, 2H), 5.48 (s, 2H), 4.39 (q, *J* = 7.1 Hz, 2H), 3.80 (s, 2H), 1.37 (t, *J* = 7.1 Hz, 3H). ACQUITY UPLC BEH C_18_ 1.7 μm: Rt = 1.77 min; *m*/*z* 373.0 [M + Na, ^79^Br]^+^, 375.0 [M + Na, ^81^Br]^+^.

#### Ethyl 1-Benzyl-5-bromo-4-[2-[[(3*S*)-8-chloro-5-methyl-4-oxo-2,3-dihydro-1,5-benzoxazepin-3-yl]­amino]­ethyl]­pyrazole-3-carboxylate (**17**)

Synthesized according to General Procedure A using ethyl 1-benzyl-5-bromo-4-(2-oxoethyl)­pyrazole-3-carboxylate (100 mg, 0.27 mmol), [(3*S*)-8-chloro-5-methyl-4-oxo-2,3-dihydro-1,5-benzoxazepin-3-yl]­ammonium trifluoroacetate (97 mg, 0.27 mmol), acetic acid (0.50 mL, 8.73 mmol) and borane-2-methylpyridine complex (38 mg, 0.35 mmol). Purified by flash column chromatography (silica, 12 g, 1:0 petrol/EtOAc to 0:1 petrol/EtOAc containing 1% Et_3_N over 25 CV’s) to give ethyl 1-benzyl-5-bromo-4-[2-[[(3*S*)-8-chloro-5-methyl-4-oxo-2,3-dihydro-1,5-benzoxazepin-3-yl]­amino]­ethyl]­pyrazole-3-carboxylate (**17**, 134 mg, 0.22 mmol, 88% yield) as a colorless solid. ^1^H NMR (500 MHz, CDCl_3_) δ 7.26–7.17 (m, 3H), 7.13–7.08 (m, 3H), 7.08–7.01 (m, 2H), 5.35 (s, 2H), 4.32–4.26 (m, 3H), 4.03–3.98 (m, 1H), 3.48 (dd, *J* = 11.6, 7.3 Hz, 1H), 3.28 (s, 3H), 2.81–2.61 (m, 3H), 2.38 (ddd, *J* = 10.3, 8.4, 5.9 Hz, 1H), 1.32–1.27 (m, 3H). NH not observed. ACQUITY UPLC BEH C_18_ 1.7 μm: Rt = 1.67 min; *m*/*z* 563.1 [M + H, ^81^Br, ^35^Cl]^+^.

#### 1-Benzyl-5-bromo-4-[2-[[(3*S*)-8-chloro-5-methyl-4-oxo-2,3-dihydro-1,5-benzoxazepin-3-yl]­amino]­ethyl]­pyrazole-3-carboxylic Acid (**18**)

Synthesized according to General Procedure B using ethyl 1-benzyl-5-bromo-4-[2-[[(3*S*)-8-chloro-5-methyl-4-oxo-2,3-dihydro-1,5-benzoxazepin-3-yl]­amino]­ethyl]­pyrazole-3-carboxylate (134 mg, 0.24 mmol) and LiOH·H_2_O (60 mg, 1.43 mmol) to give 1-benzyl-5-bromo-4-[2-[[(3*S*)-8-chloro-5-methyl-4-oxo-2,3-dihydro-1,5-benzoxazepin-3-yl]­amino]­ethyl]­pyrazole-3-carboxylic acid (**18**, 127 mg, 0.22 mmol, quantitative yield) as a colorless solid. ACQUITY UPLC BEH C_18_ 1.7 μm: Rt = 1.57 min; *m*/*z* 535.0 [M + H, ^81^Br, ^35^Cl]^+^.

#### (*S*)-3-(2-Benzyl-3-bromo-7-oxo-2,4,5,7-tetrahydro-6*H*-pyrazolo­[3,4-*c*]­pyridin-6-yl)-8-chloro-5-methyl-2,3-dihydrobenzo­[*b*]­[1,4]­oxazepin-4­(5*H*)-one (**19**)

Synthesized according to General Procedure C using 1-benzyl-5-bromo-4-[2-[[(3*S*)-8-chloro-5-methyl-4-oxo-2,3-dihydro-1,5-benzoxazepin-3-yl]­amino]­ethyl]­pyrazole-3-carboxylic acid (127 mg, 0.24 mmol), HOBt hydrate (40 mg, 0.26 mmol), and EDC·HCl (55 mg, 0.29 mmol). Purified by flash column chromatography (silica, 12 g, 1:0 petrol/EtOAc to 0:1 petrol/EtOAc over 30 CV’s) to afford (*S*)-3-(2-benzyl-3-bromo-7-oxo-2,4,5,7-tetrahydro-6*H*-pyrazolo­[3,4-*c*]­pyridin-6-yl)-8-chloro-5-methyl-2,3-dihydrobenzo­[*b*]­[1,4]­oxazepin-4­(5*H*)-one (**19**, 84 mg, 0.16 mmol, 68% yield) as a colorless solid. ^1^H NMR (500 MHz, CDCl_3_) δ 7.34–7.27 (m, 3H), 7.27–7.22 (m, 3H), 7.19–7.15 (m, 2H), 5.90 (dd, *J* = 11.8, 8.1 Hz, 1H), 5.43 (s, 2H), 4.63 (dd, *J* = 11.8, 10.0 Hz, 1H), 4.41 (dd, *J* = 10.0, 8.1 Hz, 1H), 4.23 (dt, *J* = 12.0, 5.2 Hz, 1H), 3.55 (ddd, *J* = 12.0, 10.5, 4.4 Hz, 1H), 3.35 (s, 3H), 3.04 (ddd, *J* = 15.6, 10.4, 5.1 Hz, 1H), 2.64 (ddd, *J* = 15.6, 5.3, 4.4 Hz, 1H). ACQUITY UPLC BEH C_18_ 1.7 μm: Rt = 1.85 min; *m*/*z* 539.0 [M + Na, ^81^Br, ^35^Cl]^+^. ELSD/UV/^1^H NMR purity: 100/94/90%.

#### (*S*)-1-Benzyl-6-(8-chloro-5-methyl-4-oxo-2,3,4,5-tetrahydrobenzo­[*b*]­[1,4]­oxazepin-3-yl)-7-oxo-4,5,6,7-tetrahydro-1*H*-pyrazolo­[3,4-*c*]­pyridine-3-carboxamide (**20**)

Synthesized exactly as described for LIJTF500025 (**7**) in Collins et al.,^29^ Step 5 through separation of the other regioisomer. The semipurified product collected after flash column chromatography (silica, 24 g, 1:0 petrol/EtOAc to 0:1 petrol/EtOAc over 25 CV’s) was additionally purified by reverse-phase chromatography (9:1 H_2_O/MeOH to 0:1 H_2_O/MeOH over 25 min) to afford (*S*)-1-benzyl-6-(5-methyl-4-oxo-2,3,4,5-tetrahydrobenzo­[*b*]­[1,4]­oxazepin-3-yl)-7-oxo-4,5,6,7-tetrahydro-1*H*-pyrazolo­[3,4-*c*]­pyridine-3-carboxamide (**20**, 7 mg, 0.01 mmol, 9% yield) as a colorless solid. ^1^H NMR (500 MHz, DMSO-*d*_6_) δ 7.61 (s, 1H), 7.52 (d, *J* = 8.6 Hz, 1H), 7.42–7.37 (m, 3H), 7.35–7.22 (m, 3H), 7.19–7.15 (m, 2H), 5.67–5.58 (m, 2H), 5.48 (dd, *J* = 12.0, 7.7 Hz, 1H), 4.89 (dd, *J* = 12.0, 10.1 Hz, 1H), 4.41 (dd, *J* = 10.0, 7.7 Hz, 1H), 4.00 (ddd, *J* = 12.7, 7.4, 5.4 Hz, 1H), 3.69–3.60 (m, 1H), 3.28 (s, 3H), 3.11–2.93 (m, 2H). ACQUITY UPLC BEH C_18_ 1.7 μm: Rt = 1.77 min; *m*/*z* 480.1 [M + H]^+^. ELSD/UV/^1^H NMR purity: 100/89/97%.

#### Dimethyl 1-Benzyl-4-(2-oxoethyl)-1*H*-pyrazole-3,5-dicarboxylate (**27**)

A solution of dimethyl (*E*)-1-benzyl-4-(2-ethoxyvinyl)-1*H*-pyrazole-3,5-dicarboxylate (0.57 g, 1.66 mmol) in THF (5 mL) at 0 °C was added slowly to an ice-cold solution of hydrochloric acid (6M, 5 mL, 30.0 mmol). The reaction mixture was stirred at 0 °C for 1 h, allowing to warm to room temperature. The reaction mixture was poured onto saturated NaHCO_3_ solution (50 mL) and extracted with EtOAc (3 × 25 mL). The organic phases were combined, dried over MgSO_4_, filtered and concentrated under reduced pressure to afford dimethyl 1-benzyl-4-(2-oxoethyl)-1*H*-pyrazole-3,5-dicarboxylate (**27**, 0.49 g, 1.09 mmol, 66% yield, 70% purity) as a yellow oil. ^1^H NMR (400 MHz, CDCl_3_) δ 9.71 (s, 1H), 7.35–7.16 (m, 5H), 5.85 (s, 2H), 4.27 (s, 2H), 3.95 (s, 3H), 3.84 (s, 3H). ACQUITY UPLC BEH C_18_ 1.7 μm: Rt = 1.66 min; *m*/*z* 317.0 [M + H]^+^.

#### Dimethyl (*S*)-1-Benzyl-4-(2-((5-methyl-4-oxo-2,3,4,5-tetrahydrobenzo­[*b*]­[1,4]­oxazepin-3-yl)­amino)­ethyl)-1*H*-pyrazole-3,5-dicarboxylate (**28a**)

Synthesized according to General Procedure A using dimethyl 1-benzyl-4-(2-oxoethyl)-1*H*-pyrazole-3,5-dicarboxylate (235 mg, 0.74 mmol, **27**), (3*S*)-3-amino-5-methyl-2,3,4,5-tetrahydro-1,5-benzoxazepin-4-one hydrochloride (180 mg, 0.79 mmol, 1.1 equiv), acetic acid (0.3 mL) and 2-methylpyridine borane complex (119 mg, 1.11 mmol, 1.5 equiv). Purified by flash column chromatography (silica, 24 g, 7:3 petrol/EtOAc to 0:1 petrol/EtOAc) to afford dimethyl (*S*)-1-benzyl-4-(2-((5-methyl-4-oxo-2,3,4,5-tetrahydrobenzo­[*b*]­[1,4]­oxazepin-3-yl)­amino)­ethyl)-1*H*-pyrazole-3,5-dicarboxylate (**28a**, 220 mg, 0.42 mmol, 52% yield) as a colorless glass. ^1^H NMR (500 MHz, CDCl_3_) δ 7.35–7.25 (m, 3H), 7.24–7.19 (m, 3H), 7.19–7.14 (m, 3H), 5.79 (s, 2H), 4.38 (dd, *J* = 10.1, 7.4 Hz, 1H), 4.09 (dd, *J* = 11.6, 10.1 Hz, 1H), 3.91 (s, 3H), 3.81 (s, 3H), 3.59 (dd, *J* = 11.6, 7.4 Hz, 1H), 3.41 (s, 3H), 3.18–3.03 (m, 2H), 2.87 (ddd, *J* = 11.2, 9.2, 5.4 Hz, 1H), 2.47 (ddd, *J* = 11.2, 9.0, 6.5 Hz, 1H). NH not observed. ACQUITY UPLC BEH C_18_ 1.7 μm: Rt = 1.46 min; *m*/*z* 493.2 [M + H]^+^. ELSD/UV/^1^H NMR purity: 100/87/95%.

#### (*S*)-2-Benzyl-6-(5-methyl-4-oxo-2,3,4,5-tetrahydrobenzo­[*b*]­[1,4]­oxazepin-3-yl)-7-oxo-4,5,6,7-tetrahydro-2*H*-pyrazolo­[3,4-*c*]­pyridine-3-carboxamide (**21**)

Synthesized in a three-step telescope according to (i) General Procedure B using dimethyl (*S*)-1-benzyl-4-(2-((5-methyl-4-oxo-2,3,4,5-tetrahydrobenzo­[*b*]­[1,4]­oxazepin-3-yl)­amino)­ethyl)-1*H*-pyrazole-3,5-dicarboxylate (140 mg, 0.27 mmol) and LiOH·H_2_O (57 mg, 1.35 mmol, 5 equiv). Product taken forward as crude to the next step. (ii) General Procedure C using (*S*)-1-benzyl-4-(2-((5-methyl-4-oxo-2,3,4,5-tetrahydrobenzo­[*b*]­[1,4]­oxazepin-3-yl)­amino)­ethyl)-1*H*-pyrazole-3,5-dicarboxylic acid (as crude), HOBt hydrate (45 mg, 0.29 mmol, 1.1 equiv), EDC·HCl (70 mg, 0.37 mmol, 1.3 equiv), DIPEA (0.11 mL, 0.64 mmol, 2.4 equiv). Formation of (*S*)-2-benzyl-6-(5-methyl-4-oxo-2,3,4,5-tetrahydrobenzo­[*b*]­[1,4]­oxazepin-3-yl)-7-oxo-4,5,6,7-tetrahydro-2*H*-pyrazolo­[3,4-*c*]­pyridine-3-carboxylic acid and its regioisomer were observed in approximately 1:1 ratio. Product taken forward as crude to the next step. (iii) General Procedure C using (*S*)-2-benzyl-6-(5-methyl-4-oxo-2,3,4,5-tetrahydrobenzo­[*b*]­[1,4]­oxazepin-3-yl)-7-oxo-4,5,6,7-tetrahydro-2*H*-pyrazolo­[3,4-*c*]­pyridine-3-carboxylic acid (as crude mixture of two regioisomers), HOBt hydrate (45 mg, 0.29 mmol, 1.1 equiv), EDC·HCl (70 mg, 0.37 mmol, 1.3 equiv), DIPEA (0.11 mL, 0.64 mmol, 2.4 equiv) and NH_4_Cl (60 mg, 1.12 mmol). The reaction mixture was heated to 45 °C and stirred for 24 h. Purified by flash column chromatography (silica, 24 g, 1:0 petrol/EtOAc to 0:1 petrol/EtOAc over 25 CV’s) to afford (*S*)-2-benzyl-6-(5-methyl-4-oxo-2,3,4,5-tetrahydrobenzo­[*b*]­[1,4]­oxazepin-3-yl)-7-oxo-4,5,6,7-tetrahydro-2*H*-pyrazolo­[3,4-*c*]­pyridine-3-carboxamide (**21**, 38 mg, 0.08 mmol, 28% yield) as acolorless solid. m.p.: 190 °C (dec). ^1^H NMR (500 MHz, DMSO-*d*_6_) δ 7.83 (br s, 1H), 7.73 (br s, 1H), 7.50 (dd, *J* = 7.9, 1.6 Hz, 1H), 7.35–7.23 (m, 6H), 7.19–7.15 (m, 2H), 5.63 (s, 2H), 5.55 (dd, *J* = 12.0, 7.9 Hz, 1H), 4.85 (dd, *J* = 12.0, 10.1 Hz, 1H), 4.34 (dd, *J* = 10.0, 7.9 Hz, 1H), 4.00 (ddd, *J* = 12.1, 6.9, 5.0 Hz, 1H), 3.59 (ddd, *J* = 12.4, 8.9, 4.6 Hz, 1H), 3.30 (s, 3H), 3.05 (ddd, *J* = 15.9, 8.9, 5.0 Hz, 1H), 2.84 (ddd, *J* = 15.9, 6.9, 4.6 Hz, 1H). ^13^C­{^1^H} NMR (125 MHz, DMSO-*d*_6_) δ 168.9, 161.1, 160.2, 149.5, 141.0, 137.2, 136.7, 133.1, 128.6, 127.8, 127.6, 127.2, 125.9, 123.4, 122.6, 121.4, 74.4, 54.1, 50.7, 44.9, 34.8, 20.5. A HMBC cross peak is shared between the benzyl CH_2_, amide NH_2_ and lactam benzyl CH_2_ which is only consistent with isomer as drawn. 97% purity by ^1^H NMR despite residual MeOH present. ACQUITY UPLC BEH C_18_ 1.7 μm: Rt = 1.54 min; *m*/*z* 468.1 [M + Na]^+^. HRMS calculated for C_24_H_24_N_5_O_4_: 446.1828; found 446.1817 [M + H]^+^. ELSD/UV/^1^H NMR purity: 100/92/97%. Characterization data was consistent with previously published data.^43^

#### (*S*)-1-Benzyl-6-(5-methyl-4-oxo-2,3,4,5-tetrahydrobenzo­[*b*]­[1,4]­oxazepin-3-yl)-7-oxo-4,5,6,7-tetrahydro-1*H*-pyrazolo­[3,4-*c*]­pyridine-3-carboxamide (**22**)

Synthesized according to the synthesis of **21**, Step iii through separation of the other regioisomer. The semipurified product collected after flash column chromatography (silica, 24 g, 1:0 petrol/EtOAc to 0:1 petrol/EtOAc over 25 CV’s) was additionally purified by reverse-phase chromatography (9:1 H_2_O/MeOH to 0:1 H_2_O/MeOH over 25 min) to afford (*S*)-1-benzyl-6-(5-methyl-4-oxo-2,3,4,5-tetrahydrobenzo­[*b*]­[1,4]­oxazepin-3-yl)-7-oxo-4,5,6,7-tetrahydro-1*H*-pyrazolo­[3,4-*c*]­pyridine-3-carboxamide (**22**, 18 mg, 0.04 mmol, 15% yield) as a colorless solid. m.p.: 212–213 °C. ^1^H NMR (500 MHz, DMSO-*d*_6_) δ 7.61 (s, 1H), 7.51–7.46 (m, 1H), 7.40 (d, *J* = 1.7 Hz, 1H), 7.36–7.21 (m, 6H), 7.19–7.13 (m, 2H), 5.67–5.57 (m, 2H), 5.49 (dd, *J* = 12.0, 7.8 Hz, 1H), 4.86 (dd, *J* = 12.0, 10.1 Hz, 1H), 4.35 (dd, *J* = 10.1, 7.8 Hz, 1H), 4.02 (ddd, *J* = 12.6, 7.3, 5.4 Hz, 1H), 3.65 (ddd, *J* = 12.6, 8.4, 5.3 Hz, 1H), 3.29 (s, 3H), 3.09–2.93 (m, 2H). ^13^C­{^1^H} NMR (125 MHz, DMSO-*d*_6_) δ 168.6 (Cq), 163.1 (Cq), 158.2 (Cq), 149.4 (Cq), 140.8 (Cq), 136.9 (Cq), 136.6 (Cq), 131.7 (Cq), 128.6 (CH), 127.7 (CH), 127.4 (CH), 127.2 (CH), 125.9 (CH), 124.3 (CH), 123.4 (CH), 122.5 (Cq), 74.4 (CH_2_), 53.9 (CH_2_), 50.6 (CH), 45.3 (CH_2_), 34.8 (CH_3_), 20.7 (CH_2_). ACQUITY UPLC BEH C_18_ 1.7 μm: Rt = 1.66 min; *m*/*z* 468.1 [M + Na]^+^. HRMS calculated for C_24_H_24_N_5_O_4_: 446.1828, found: 446.1819 [M + H]^+^. ELSD/UV/^1^H NMR purity: 98/91/98%.

#### (*S*)-2-Benzyl-*N*-methyl-6-(5-methyl-4-oxo-2,3,4,5-tetrahydrobenzo­[*b*]­[1,4]­oxazepin-3-yl)-7-oxo-4,5,6,7-tetrahydro-2*H*-pyrazolo­[3,4-*c*]­pyridine-3-carboxamide (**23**)

Synthesized according to the synthesis of **21** using MeNH_2_·HCl in place of NH_4_Cl in the last step. (*S*)-2-Benzyl-6-(5-methyl-4-oxo-2,3,4,5-tetrahydrobenzo­[*b*]­[1,4]­oxazepin-3-yl)-7-oxo-4,5,6,7-tetrahydro-2*H*-pyrazolo­[3,4-*c*]­pyridine-3-carboxylic acid (177 mg, as crude mixture of two regioisomers), HOBt hydrate (60 mg, 0.39 mmol), EDC·HCl (95 mg, 0.50 mmol), DIPEA (1.45 mL, 0.85 mmol), MeNH_2_·HCl (60 mg, 2.47 mmol). The semipurified product collected after flash column chromatography (silica, 24 g, 1:0 petrol/EtOAc to 0:1 petrol/EtOAc over 25 CV’s) was additionally purified by reverse-phase chromatography (9:1 H_2_O/MeOH to 0:1 H_2_O/MeOH over 25 min) to afford (*S*)-2-benzyl-*N*-methyl-6-(5-methyl-4-oxo-2,3,4,5-tetrahydrobenzo­[*b*]­[1,4]­oxazepin-3-yl)-7-oxo-4,5,6,7-tetrahydro-2*H*-pyrazolo­[3,4-*c*]­pyridine-3-carboxamide (**23**, 10 mg, 0.02 mmol, 6% yield) as a colorless glass. ^1^H NMR (500 MHz, DMSO-*d*_6_) δ 8.23 (q, *J* = 4.5 Hz, 1H), 7.50 (dd, *J* = 7.9, 1.6 Hz, 1H), 7.35–7.23 (m, 6H), 7.20–7.15 (m, 2H), 5.59 (s, 2H), 5.55 (dd, *J* = 12.0, 7.9 Hz, 1H), 4.84 (dd, *J* = 12.0, 10.1 Hz, 1H), 4.34 (dd, *J* = 10.1, 7.9 Hz, 1H), 4.00 (ddd, *J* = 12.1, 6.8, 5.0 Hz, 1H), 3.59 (ddd, *J* = 12.3, 9.0, 4.6 Hz, 1H), 3.30 (s, 3H), 3.03 (ddd, *J* = 15.8, 9.0, 5.0 Hz, 1H), 2.82 (ddd, *J* = 15.9, 6.8, 4.6 Hz, 1H), 2.76 (d, *J* = 4.6 Hz, 3H). ACQUITY UPLC BEH C_18_ 1.7 μm: Rt = 1.71 min; *m*/*z* 460.2 [M + H]^+^. ELSD/UV/^1^H NMR purity: 98/95/90%.

#### (*S*)-1-Benzyl-*N*-methyl-6-(5-methyl-4-oxo-2,3,4,5-tetrahydrobenzo­[*b*]­[1,4]­oxazepin-3-yl)-7-oxo-4,5,6,7-tetrahydro-1*H*-pyrazolo­[3,4-*c*]­pyridine-3-carboxamide (**24**)

Synthesized according to the synthesis of **23** through separation of the other regioisomer. Purified by flash column chromatography (silica, 24 g, 7:3 petrol/EtOAc to 0:1 petrol/EtOAc over 25 CV’s) followed by reverse-phase chromatography (9:1 H_2_O/MeOH to 0:1 H_2_O/MeOH over 25 min) to afford (*S*)-1-benzyl-*N*-methyl-6-(5-methyl-4-oxo-2,3,4,5-tetrahydrobenzo­[*b*]­[1,4]­oxazepin-3-yl)-7-oxo-4,5,6,7-tetrahydro-1*H*-pyrazolo­[3,4-*c*]­pyridine-3-carboxamide (**24**, 6 mg, 0.01 mmol, 3% yield) as a colorless solid. ^1^H NMR (500 MHz, DMSO-*d*_6_) δ 8.17 (q, *J* = 4.6 Hz, 1H), 7.51–7.45 (m, 1H), 7.34–7.22 (m, 6H), 7.17–7.14 (m, 2H), 5.68–5.57 (m, 2H), 5.49 (dd, *J* = 12.0, 7.8 Hz, 1H), 4.86 (dd, *J* = 12.0, 10.1 Hz, 1H), 4.36 (dd, *J* = 10.1, 7.8 Hz, 1H), 4.03 (ddd, *J* = 12.7, 7.4, 5.4 Hz, 1H), 3.70–3.61 (m, 1H), 3.29 (s, 3H), 3.10–2.94 (m, 2H), 2.72 (d, *J* = 4.7 Hz, 3H). ACQUITY UPLC BEH C_18_ 1.7 μm: Rt = 1.83 min; *m*/*z* 460.2 [M + H]^+^. ELSD/UV/^1^H NMR purity: 100/91/94%.

#### (*S*)-2-Benzyl-*N*,*N*-dimethyl-6-(5-methyl-4-oxo-2,3,4,5-tetrahydrobenzo­[*b*]­[1,4]­oxazepin-3-yl)-7-oxo-4,5,6,7-tetrahydro-2*H*-pyrazolo­[3,4-*c*]­pyridine-3-carboxamide (**25**)

Synthesized according to the synthesis of **21** using Me_2_NH in place of NH_4_Cl in the last step. (*S*)-2-Benzyl-6-(5-methyl-4-oxo-2,3,4,5-tetrahydrobenzo­[*b*]­[1,4]­oxazepin-3-yl)-7-oxo-4,5,6,7-tetrahydro-2*H*-pyrazolo­[3,4-*c*]­pyridine-3-carboxylic acid (177 mg, as crude mixture of two regioisomers), HOBt hydrate (60 mg, 0.39 mmol), EDC·HCl (95 mg, 0.50 mmol), DIPEA (1.45 mL, 0.85 mmol), Me_2_NH (0.10 mL, 0.56 mmol). The semipurified product collected after flash column chromatography (silica, 24 g, 1:0 petrol/EtOAc to 0:1 petrol/EtOAc over 25 CV’s) was additionally purified by reverse-phase chromatography (9:1 H_2_O/MeOH to 0:1 H_2_O/MeOH over 25 min) to afford (*S*)-2-benzyl-*N*,*N*-dimethyl-6-(5-methyl-4-oxo-2,3,4,5-tetrahydrobenzo­[*b*]­[1,4]­oxazepin-3-yl)-7-oxo-4,5,6,7-tetrahydro-2*H*-pyrazolo­[3,4-*c*]­pyridine-3-carboxamide (**25**, 6 mg, 0.01 mmol, 3% yield) as a colorless glass. ^1^H NMR (500 MHz, DMSO-*d*_6_) δ 7.50 (dd, *J* = 7.7, 1.8 Hz, 1H), 7.36–7.22 (m, 6H), 7.19–7.12 (m, 2H), 5.57 (dd, *J* = 12.0, 7.9 Hz, 1H), 5.37 (s, 2H), 4.82 (dd, *J* = 12.0, 10.1 Hz, 1H), 4.33 (dd, *J* = 10.1, 7.9 Hz, 1H), 4.01 (ddd, *J* = 12.1, 6.9, 4.9 Hz, 1H), 3.60 (ddd, *J* = 12.8, 8.8, 4.6 Hz, 1H), 3.30 (s, 3H), 2.90 (s, 3H), 2.80 (ddd, *J* = 14.4, 8.9, 4.9 Hz, 1H), 2.71–2.65 (m, 4H). ACQUITY UPLC BEH C_18_ 1.7 μm: Rt = 1.72 min; *m*/*z* 474.2 [M + H]^+^. ELSD/UV/^1^H NMR purity: 99/94/94%.

#### (*S*)-1-Benzyl-*N*,*N*-dimethyl-6-(5-methyl-4-oxo-2,3,4,5-tetrahydrobenzo­[*b*]­[1,4]­oxazepin-3-yl)-7-oxo-4,5,6,7-tetrahydro-1*H*-pyrazolo­[3,4-*c*]­pyridine-3-carboxamide (**26**)

Synthesized according to the synthesis of **25** through separation of the other regioisomer. Purified by flash column chromatography (silica, 24 g, 7:3 petrol/EtOAc to 0:1 petrol/EtOAc over 25 CV’s) followed by reverse-phase chromatography (9:1 H_2_O/MeOH to 0:1 H_2_O/MeOH over 25 min) to afford (*S*)-1-benzyl-*N*,*N*-dimethyl-6-(5-methyl-4-oxo-2,3,4,5-tetrahydrobenzo­[*b*]­[1,4]­oxazepin-3-yl)-7-oxo-4,5,6,7-tetrahydro-1*H*-pyrazolo­[3,4-*c*]­pyridine-3-carboxamide (**26**, 7 mg, 0.01 mmol, 4% yield) as a colorless solid. ^1^H NMR (500 MHz, DMSO-*d*_6_) δ 7.49 (dd, *J* = 7.7, 1.8 Hz, 1H), 7.35–7.22 (m, 6H), 7.18–7.15 (m, 2H), 5.66–5.58 (m, 2H), 5.50 (dd, *J* = 12.0, 7.8 Hz, 1H), 4.86 (dd, *J* = 12.0, 10.1 Hz, 1H), 4.36 (dd, *J* = 10.1, 7.8 Hz, 1H), 4.02 (ddd, *J* = 12.7, 7.4, 5.3 Hz, 1H), 3.64 (ddd, *J* = 12.6, 8.5, 5.2 Hz, 1H), 3.30 (s, 3H), 3.20 (s, 3H), 3.00–2.80 (m, 5H). ACQUITY UPLC BEH C_18_ 1.7 μm: Rt = 1.85 min; *m*/*z* 474.2 [M + H]^+^. ELSD/UV/^1^H NMR purity: 100/92/90%.

#### (*S*)-1-Benzyl-*N* ^3^-(5-methyl-4-oxo-2,3,4,5-tetrahydrobenzo­[*b*]­[1,4]­oxazepin-3-yl)-1*H*-pyrazole-3,5-dicarboxamide (**29**)

Synthesized according to General Procedure C using 1-benzyl-5-carbamoyl-1*H*-pyrazole-3-carboxylic acid (80 mg, 0.33 mmol), HOBt hydrate (55 mg, 0.36 mmol, 1.1 equiv), EDC·HCl (75 mg, 0.39 mmol, 1.2 equiv), DIPEA (84 μL, 0.49 mmol, 1.5 equiv) and (S)-3-amino-5-methyl-2,3-dihydrobenzo­[*b*]­[1,4]­oxazepin-4­(5*H*)-one hydrochloride (82 mg, 0.36 mmol, 1.1 equiv). Purified by flash column chromatography (silica, 4 g, 1:0 petrol/EtOAc to 0:1 petrol/EtOAc over 25 CV’s) to afford (*S*)-1-benzyl-*N* ^3^-(5-methyl-4-oxo-2,3,4,5-tetrahydrobenzo­[*b*]­[1,4]­oxazepin-3-yl)-1*H*-pyrazole-3,5-dicarboxamide (**29**, 70 mg, 0.16 mmol, 49% yield) as a colorless solid. m.p.: 146–147 °C. ^1^H NMR (500 MHz, CDCl_3_) δ 7.86 (d, *J* = 7.1 Hz, 1H), 7.32–7.30 (m, 4H), 7.30–7.17 (m, 5H), 7.12 (s, 1H), 6.06 (s, 1H), 5.84 (d, *J* = 14.3 Hz, 1H), 5.77 (d, *J* = 14.3 Hz, 1H), 5.35 (s, 1H), 5.03 (dt, *J* = 11.2, 7.2 Hz, 1H), 4.72 (dd, *J* = 9.8, 7.4 Hz, 1H), 4.29 (dd, *J* = 11.2, 9.8 Hz, 1H), 3.45 (s, 3H). ^13^C­{^1^H} NMR (101 MHz, CDCl_3_) δ 169.1 (Cq), 161.0 (Cq), 160.6 (Cq), 150.2 (Cq), 144.3 (Cq), 136.6 (Cq), 136.3 (Cq), 135.6 (Cq), 128.7 (CH), 128.1 (CH), 128.0 (CH), 126.0 (CH), 123.5 (CH), 123.0 (CH), 109.1 (CH), 77.3 (CH_2_), 55.6 (CH_2_), 49.3 (CH), 35.6 (CH_3_). ACQUITY UPLC BEH C_18_ 1.7 μm: Rt = 1.73 min; *m*/*z* 420.2 [M + H]^+^. HRMS calculated for C_22_H_22_N_5_O_4_: 420.1672, found: 420.1669 [M + H]^+^. ELSD/UV/^1^H NMR purity: 99/91/96%.

#### 1-Benzyl-3,5-dibromo-1*H*-pyrazole (**36**)

A suspension of 3,5-dibromo-1*H*-pyrazole (2.50 g, 11.07 mmol) and K_2_CO_3_ (2.06 g, 14.68 mmol) in DMF (15 mL) was cooled in an ice-bath. Benzyl bromide (1.34 mL, 11.27 mmol) was added slowly over 5 min. The bath was removed and the mixture stirred at room temperature for 40 min, whereupon UPLC showed complete conversion to product. The reaction mixture was poured onto 350 mL ice-cold water, extracted with EtOAc (3 × 40 mL) and washed with brine (5 × 20 mL), dried over MgSO_4_, filtered and concentrated under reduced pressure to give 1-benzyl-3,5-dibromo-1*H*-pyrazole (**36**, 3.34 g, 10.04 mmol, 96% yield) as a light-yellow thick oil. ^1^H NMR (500 MHz, CDCl_3_) δ 7.41–7.31 (m, 3H), 7.30–7.22 (m, 2H), 6.36 (s, 1H), 5.35 (s, 2H). ACQUITY UPLC BEH C_18_ 1.7 μm: Rt = 1.83 min; *m*/*z* 316.8 [M + H, ^79^Br, ^81^Br]^+^.

#### 1-Benzyl-3-bromo-1*H*-pyrazole-5-carboxylic Acid (**37**)

To a cooled (−78 °C) solution of **36** (3.29 g, 10.4 mmol) in anhydrous THF (80 mL) was added dropwise iPrMgCl·LiCl (1.3 M in THF, 9.60 mL, 12.48 mmol). The solution took on a light-yellow color and was left to stir for 30 min. CO_2_ was bubbled through the solution *via* cannula over a period of 20 min before removal of the cooling bath. The reaction mixture was concentrated under reduced pressure and redissolved in saturated aqueous solution of NaHCO_3_ (50 mL) and extracted with DCM (20 mL). This extract was discarded and the aqueous brought to pH 3 and extracted with EtOAc (4 × 30 mL). The combined EtOAc extract was dried over MgSO_4_, filtered and concentrated under reduced pressure to give 1-benzyl-3-bromo-1*H*-pyrazole-5-carboxylic acid (**37**, 2.61 g, 9.30 mmol, 94% yield) as a colorless solid. ^1^H NMR (500 MHz, CD_3_OD) δ 7.33–7.23 (m, 3H), 7.22 (ddd, *J* = 8.1, 1.4, 0.7 Hz, 2H), 6.89 (s, 1H), 5.72 (s, 2H). ACQUITY UPLC BEH C_18_ 1.7 μm: Rt = 1.54 min; *m*/*z* 282.9 [M + H, ^81^Br]^+^.

#### 1-Benzyl-3-bromo-1*H*-pyrazole-5-carboxamide (**38**)

A mixture of **37** (198 mg, 0.70 mmol) and SOCl_2_ (1.0 mL, 13.71 mmol) was heated to 65 °C for 2 h. Once cooled, the mixture was concentrated under reduced pressure in a fumehood to give the acid chloride (colorless oil), which was used directly without further purification. The acid chloride was dissolved in DCM (5 mL) and cooled in an ice bath. Ammonia solution (0.20 mL, 35% by w/w aqueous solution) was added slowly dropwise. The cooling bath was removed and the reaction left to stir at room temperature overnight. The mixture was diluted with DCM (20 mL) and poured into a separating funnel containing a saturated aqueous solution of NaHCO_3_ (20 mL). The layers were separated and the organic extract washed with additional NaHCO_3_ solution (20 mL). The organic phase was dried over MgSO_4_, filtered and concentrated under reduced pressure to give 1-benzyl-3-bromo-1*H*-pyrazole-5-carboxamide (**38**, 171 mg, 0.58 mmol, 86% yield) as a colorless viscous oil. ^1^H NMR (500 MHz, CD_3_OD) δ 7.29–7.19 (m, 5H), 6.86 (s, 1H), 5.72 (s, 2H), 4.93 (s, 2H). ACQUITY UPLC BEH C_18_ 1.7 μm: Rt = 1.42 min; *m*/*z* 281.9 [M + H, ^81^Br]^+^.

#### (*E*)-1-Benzyl-3-(2-ethoxyvinyl)-1*H*-pyrazole-5-carboxamide (**39**)

A mixture of (*E*)-(2-ethoxyvinyl)­boronic pinacol ester (0.22 mL, 1.04 mmol), **38** (171 mg, 0.61 mmol) and Cs_2_CO_3_ (0.42 g, 1.29 mmol) in monoglyme (4 mL) and water (0.4 mL) was degassed with N_2_ for 15 min. [1,1′-Bis­(diphenylphosphino)­ferrocene]­dichloropalladium­(II) (0.04 g, 0.06 mmol) was added, the mixture degassed for a further 10 min before being heated to 90 °C overnight. The mixture was diluted with EtOAc (20 mL), washed with a saturated aqueous solution of NaHCO_3_ (20 mL), the aqueous back-extracted with EtOAc (20 mL), the combined organics dried over MgSO_4_, filtered and concentrated under reduced pressure to give the crude product, which was purified by automated column chromatography on silica (ISCO, 12 g, slow gradient from DCM to 20% MeOH in DCM with 1% Et_3_N) to give (*E*)-1-benzyl-3-(2-ethoxyvinyl)-1*H*-pyrazole-5-carboxamide (**39**, 162 mg, 0.54 mmol, 89% yield) as a light brown solid. ^1^H NMR (500 MHz, CDCl_3_) δ 7.32–7.19 (m, 5H), 7.07 (d, *J* = 13.2 Hz, 1H), 6.44 (s, 1H), 5.86 (s, 1H), 5.77 (d, *J* = 13.2 Hz, 1H), 5.70 (s, 2H), 5.64 (s, 1H), 3.87 (q, *J* = 7.0 Hz, 2H), 1.33 (t, *J* = 7.0 Hz, 3H). ACQUITY UPLC BEH C_18_ 1.7 μm: Rt = 1.42 min; *m*/*z* 272.0 [M + H]^+^. Regiochemistry shown by small X-ray crystal structure analysis.

#### Methyl 1-Benzyl-3-bromo-1*H*-pyrazole-5-carboxylate Hydrochloride (**41**)

To an ice-cold solution of **37** (2.00 g, 6.76 mmol) in MeOH (15 mL) was added dropwise SOCl_2_ (1.48 mL, 20.28 mmol). Upon completion of addition, the cooling bath was removed and the mixture allowed to stir at room temperature for 1 h, overnight at room temperature, and finally at 50 °C for a further 12 h. The mixture was concentrated under reduced pressure in a fumehood to give methyl 1-benzyl-3-bromo-1*H*-pyrazole-5-carboxylate hydrochloride (**41**, 2.20 g, 6.30 mmol, 98% yield) as a brown solid. ^1^H NMR (500 MHz, DMSO-*d*_*6*_) δ 7.36–7.24 (m, 3H), 7.17 (d, *J* = 7.3 Hz, 2H), 7.06 (s, 1H), 5.67 (s, 2H), 3.81 (s, 3H). ACQUITY UPLC BEH C_18_ 1.7 μm: Rt = 1.80 min; *m*/*z* 297.1 [M-HCl + H, ^81^Br]^+^.

#### Methyl (*E*)-1-Benzyl-3-(2-ethoxyvinyl)-1*H*-pyrazole-5-carboxylate (**42**)

A mixture of (*E*)-(2-ethoxyvinyl)­boronic pinacol ester (0.58 mL, 2.73 mmol) Cs_2_CO_3_ (2.03 g, 6.18 mmol) and **41** (683 mg, 2.06 mmol) in monoglyme (6.2 mL) and water (0.62 mL) was degassed with N_2_ for 15 min. [1,1′-Bis­(diphenylphosphino)­ferrocene]­dichloropalladium­(II) (0.12 g, 0.16 mmol) was added, the mixture degassed again for 10 min before heating overnight at 80 °C. The reaction mixture was diluted with EtOAc (40 mL), washed with water (15 mL), the organic phase dried over MgSO_4_, filtered and concentrated under reduced pressure to give the crude product, which was purified by automated column chromatography on silica (ISCO, 12 g, gradient from petroleum ether to EtOAc with 1% Et_3_N) to give methyl (*E*)-1-benzyl-3-(2-ethoxyvinyl)-1*H*-pyrazole-5-carboxylate (**42**, 230 mg, 0.76 mmol, 39% yield) as a colorless solid. ^1^H NMR (500 MHz, CDCl_3_) δ 7.31–7.26 (m, 2H), 7.26–7.21 (m, 3H), 7.10 (d, *J* = 13.2 Hz, 1H), 6.74 (s, 1H), 5.80 (d, *J* = 13.2 Hz, 1H), 5.70 (s, 2H), 3.88 (q, *J* = 7.0 Hz, 2H), 3.81 (s, 3H), 1.33 (t, *J* = 7.0 Hz, 3H). ACQUITY UPLC BEH C_18_ 1.7 μm: Rt = 1.78 min; *m*/*z* 287.1 [M + H]^+^.

#### Methyl 1-Benzyl-3-(2-oxoethyl)-1*H*-pyrazole-5-carboxylate (**43**)

**42** (230 mg, 0.8 mmol) was dissolved in DCM (2 mL) and 4 M HCl in dioxane (2 mL) was added. UPLC analysis after 1 h indicated reaction was complete. The mixture was taken to pH 9 by portion-wise addition of a saturated aqueous solution of NaHCO_3_ (30 mL) and extracted with EtOAc (3 × 30 mL). The organic phase was dried over MgSO_4_, filtered and concentrated under reduced pressure to give methyl 1-benzyl-3-(2-oxoethyl)-1*H*-pyrazole-5-carboxylate (**43**, 208 mg, 0.77 mmol, quantitative yield) as a colorless oil, which was used immediately in the next step due to stability concerns. ACQUITY UPLC BEH C_18_ 1.7 μm: Rt = 1.54 min; *m*/*z* 259.1 [M + H]^+^.

#### 2-(1-Benzyl-5-(methoxycarbonyl)-1*H*-pyrazol-3-yl)­acetic Acid (**44**)

To a solution of **43** (222 mg, 0.86 mmol) in DMF (8 mL) was added oxone (528 mg, 0.86 mmol). UPLC analysis after stirring at room temperature for 1 h showed complete conversion to the acid. Aqueous 1 M HCl (15 mL) and EtOAc (50 mL) were added to the mixture. The layers were separated and the aqueous back-extracted with EtOAc (20 mL). The combined organic extracts were dried over MgSO_4_, filtered and concentrated under reduced pressure to give the crude product, which was triturated with petroleum ether (3 × 35 mL) to give 2-(1-benzyl-5-(methoxycarbonyl)-1*H*-pyrazol-3-yl)­acetic acid (**44**, 143 mg, 0.47 mmol, 61% yield) as a colorless solid. ^1^H NMR (500 MHz, CDCl_3_) δ 7.32–7.26 (m, 2H), 7.26–7.19 (m, 3H), 6.85 (s, 1H), 5.73 (s, 2H), 3.84 (s, 3H), 3.78 (s, 2H). CO_2_H not observed. ACQUITY UPLC BEH C_18_ 1.7 μm: Rt = 1.52 min; *m*/*z* 275.1 [M + H]^+^.

#### Methyl 1-Benzyl-3-(2-(benzyl­(cyclopropylmethyl)­amino)-2-oxoethyl)-1*H*-pyrazole-5-carboxylate (**45**)

To a mixture of HOBt (69.7 mg, 0.45 mmol), EDC·HCl (103 mg, 0.54 mmol), Et_3_N (0.09 mL, 0.62 mmol) and **44** (126 mg, 0.41 mmol) in DCM (2 mL) was added a solution of *N*-benzyl-1-cyclopropylmethanamine (66.7 mg, 0.41 mmol) in DCM (2 mL). The mixture was left to stir overnight at room temperature. UPLC analysis showed the desired product, along with an unknown side-product. Saturated aqueous NaHCO_3_ (5 mL) was added and the mixture stirred vigorously for 5 min before being passed through a phase separator. The filtrate was purified by normal phase automated column chromatography (12 g silica, ISCO, gradient from petroleum ether to EtOAc) to give methyl 1-benzyl-3-(2-(benzyl­(cyclopropylmethyl)­amino)-2-oxoethyl)-1*H*-pyrazole-5-carboxylate (**45**, 39 mg, 0.09 mmol, 22% yield) as a light-yellow oil. ^1^H NMR (500 MHz, CDCl_3_) δ 7.27–6.97 (m, 10H), 6.81 (s, 0.5H), 6.77 (s, 0.5H), 5.61 (s, 1H), 5.56 (s, 1H), 4.64 (s, 1H), 4.61 (s, 1H), 3.76 (s, 1H), 3.71 (s, 1.5H), 3.70 (s, 1.5H), 3.65 (s, 1H), 3.19 (d, *J* = 6.9 Hz, 1H), 3.07 (d, *J* = 6.6 Hz, 1H), 0.90–0.81 (m, 0.5H), 0.78–0.68 (m, 0.5H), 0.40–0.35 (m, 1H), 0.35–0.29 (m, 1H), 0.09–0.03 (m, 1H), 0.02 to −0.04 (m, 1H). Rotamers observed in approximately 1:1 ratio. ACQUITY UPLC BEH C_18_ 1.7 μm: Rt = 1.84 min; *m*/*z* 418.3 [M + H]^+^.

#### 1-Benzyl-3-(2-(benzyl­(cyclopropylmethyl)­amino)-2-oxoethyl)-1*H*-pyrazole-5-carboxylic Acid (**46**)

**45** (39 mg, 0.09 mmol) was dissolved in MeOH (0.25 mL), water (0.25 mL) and THF (0.25 mL). LiOH·H_2_O (5.0 mg, 0.12 mmol) was added and the mixture stirred for 2 h at room temperature. The mixture was concentrated under reduced pressure and aqueous 1 M HCl (5.0 mL) was added. The product precipitated out, the supernatant was removed by pipet and the solid washed twice with water (5 mL). The solid was dried in a vacuum oven overnight at 40 °C to give 1-benzyl-3-(2-(benzyl­(cyclopropylmethyl)­amino)-2-oxoethyl)-1*H*-pyrazole-5-carboxylic acid (**46**, 39 mg, 0.09 mmol, 98% yield) as a colorless solid. ^1^H NMR (500 MHz, CDCl_3_) δ 7.35–7.10 (m, 10H), 6.96 (s, 0.5H), 6.91 (s, 0.5H), 5.71 (s, 1H), 5.66 (s, 1H), 4.78 (s, 1H), 4.76 (s, 1H), 3.92 (s, 1H), 3.81 (s, 1H), 3.33 (d, *J* = 6.9 Hz, 1H), 3.21 (d, *J* = 6.6 Hz, 1H), 0.97 (dddd, *J* = 11.9, 7.0, 4.0, 1.6 Hz, 0.5H), 0.90–0.80 (m, 0.5H), 0.51–0.46 (m, 1H), 0.46–0.40 (m, 1H), 0.19–0.14 (m, 1H), 0.13–0.08 (m, 1H). CO_2_H not observed. Rotamers observed in approximately 1:1 ratio. ACQUITY UPLC BEH C_18_ 1.7 μm: Rt = 1.68 min; *m*/*z* 404.3 [M + H]^+^.

#### 1-Benzyl-3-(2-(benzyl­(cyclopropylmethyl)­amino)-2-oxoethyl)-1*H*-pyrazole-5-carboxamide (**31**)

To a solution of PyBOP (54 mg, 0.14 mmol), HOBt (22.2 mg, 0.14 mmol) and **46** (39 mg, 0.1 mmol) in DMF (0.4 mL) was added DIPEA (0.07 mL, 0.39 mmol) followed by NH_4_Cl (10.3 mg, 0.19 mmol). The mixture was left to stir for 1 h, after which it was diluted in EtOAc (20 mL) and washed with a saturated solution of NaHCO_3_ (3 × 30 mL). The organic fraction was dried over MgSO_4_, filtered and concentrated under reduced pressure to give the crude product, which was purified by automated reverse-phase column chromatography (ACCQPrep, 30 min run from water to MeOH) to afford 1-benzyl-3-(2-(benzyl­(cyclopropylmethyl)­amino)-2-oxoethyl)-1*H*-pyrazole-5-carboxamide (**31**, 19 mg, 0.05 mmol, 48% yield) as a colorless viscous oil, which solidified upon standing. m.p.: 109–110 °C. ^1^H NMR (500 MHz, CDCl_3_) δ 7.32 (dd, *J* = 8.1, 6.5 Hz, 1H), 7.29–7.18 (m, 7H), 7.18–7.15 (m, 1H), 7.15–7.11 (m, 1H), 6.75 (s, 0.5H), 6.70 (s, 0.5H), 6.19 (s, 1H), 5.74 (s, 1H), 5.68 (s, 1H), 5.51 (s, 1H), 4.73 (s, 2H), 3.86 (s, 1H), 3.75 (s, 1H), 3.29 (d, *J* = 6.9 Hz, 1H), 3.20 (d, *J* = 6.6 Hz, 1H), 1.00–0.91 (m, 0.5H), 0.86 (dddd, *J* = 13.1, 9.7, 5.8, 1.5 Hz, 0.5H), 0.52–0.46 (m, 1H), 0.46–0.41 (m, 1H), 0.16 (dt, *J* = 6.1, 4.7 Hz, 1H), 0.11 (dt, *J* = 6.0, 4.8 Hz, 1H). ^13^C­{^1^H} NMR (101 MHz, CDCl_3_) δ 170.6 (Cq), 170.1 (Cq), 161.2 (Cq), 145.7 (Cq), 145.6 (Cq), 137.5 (Cq), 137.5 (Cq), 136.9 (Cq), 135.1 (Cq), 135.0 (Cq), 129.0 (CH), 128.7 (CH), 128.6 (CH), 128.6 (CH), 127.8 (CH), 127.8 (CH), 127.7 (CH), 127.7 (CH), 127.7 (CH), 127.4 (CH), 126.4 (CH), 108.1 (CH), 108.0 (CH), 54.6 (CH_2_), 54.5 (CH_2_), 51.9 (CH_2_), 51.5 (CH_2_), 50.4 (CH_2_), 48.5 (CH_2_), 47.8 (CH_2_), 47.8 (CH_2_), 34.0 (CH_2_), 33.8 (CH_2_), 10.2 (CH), 9.6 (CH), 3.9 (CH_2_), 3.7 (CH_2_). Rotamers observed in approximately 1:1 ratio. ACQUITY UPLC BEH C_18_ 1.7 μm: Rt = 1.63 min; *m*/*z* 403.3 [M + H]^+^. HRMS calculated for C_24_H_27_N_4_O_2_, 403.2134, found 403.2136 [M + H]^+^. ELSD/UV/^1^H NMR purity: nd/96/95%.

#### Dimethyl 1-Benzyl-1*H*-pyrazole-3,5-dicarboxylate (**48**)

A suspension of dimethyl 1*H*-pyrazole-3,5-dicarboxylate (5.00 g, 27.15 mmol) and K_2_CO_3_ (5.05 g, 36 mmol) in DMF (50 mL) was cooled in an ice-bath. Benzyl bromide (3.28 mL, 27.64 mmol) was added slowly over 5 min. The bath was removed and the mixture stirred at room temperature for 1 h, whereupon UPLC showed complete conversion to product. The reaction mixture was poured onto 2 L ice-cold water, which precipitated the desired product. The solid was collected by filtration under vacuum and dried in a vacuum oven overnight to give dimethyl 1-benzylpyrazole-3,5-dicarboxylate (**48**, 6.954 g, 24.087 mmol, 93% yield) as a colorless solid. ^1^H NMR (500 MHz, CDCl_3_) δ 7.31 (s, 1H), 7.25–7.16 (m, 5H), 5.77 (s, 2H), 3.88 (s, 3H), 3.79 (s, 3H). ACQUITY UPLC BEH C_18_ 1.7 μm: Rt = 1.65 min; *m*/*z* 275.0 [M + H]^+^.

#### Methyl 1-Benzyl-5-carbamoyl-1*H*-pyrazole-3-carboxylate (**49**)

Synthesized according to method described in US 10,966,961 B2.^52^ To an ice-cold solution of dimethyl 1-benzylpyrazole-3,5-dicarboxylate (5.00 g, 17.32 mmol) in MeOH (40 mL), THF (40 mL) and water (16 mL) was added LiOH·H_2_O (727 mg, 17.3 mmol). UPLC analysis after 2 h showed a mixture of starting material (30% by UV), monohydrolyzed material (66% by UV) and bis-hydrolyzed material (4%). The reaction mixture was concentrated under reduced pressure to give a basic aqueous mixture, which was diluted with water (40 mL) and extracted with DCM (2 × 40 mL). The aqueous phase was acidified and extracted with DCM (5 × 40 mL). The combined second extract was dried over MgSO_4_, filtered, and concentrated under reduced pressure to give the crude products of monohydrolysis, which was used directly in the next step. ACQUITY UPLC BEH C_18_ 1.7 μm: Rt = 1.48 min; *m*/*z* 261.0 [M + H]^+^.

The monohydrolyzed material from above (3.25 g, 12.48 mmol) was heated in thionyl chloride (19.15 mL, 262.55 mmol) for 2 h. Excess thionyl chloride was removed under reduced pressure in a fumehood and the residue dissolved in DCM (10 mL) and added dropwise to a solution of ammonia solution (35% by weight solution in water, 0.32 mL, 49.92 mmol). UPLC analysis showed reaction completion with both regioisomers were present (80:20 ratio). Additional DCM (20 mL) was added and the mixture washed with aqueous saturated NaHCO_3_ solution (2 × 30 mL). The organic phase was dried over MgSO_4_, filtered and concentrated under reduced pressure to give the crude product, which was purified by flash column chromatography (silica, 12 g, 1:0 petrol/EtOAc to 0:1 petrol/EtOAc containing 5% Et_3_N over 25 CV’s) to afford methyl 1-benzyl-5-carbamoyl-pyrazole-3-carboxylate (**49**, 3.00 g, 10.99 mmol, 63% yield over two steps) as a colorless solid. ^1^H NMR (500 MHz, CD_3_OD) δ 7.34 (s, 1H), 7.31–7.22 (m, 5H), 5.83 (s, 2H), 3.90 (s, 3H). CONH_2_ not observed. ACQUITY UPLC BEH C_18_ 1.7 μm: Rt = 1.42 min; *m*/*z* 260.0 [M + H]^+^. Regioselectivity of monohydrolysis proven by single crystal analysis.

#### 1-Benzyl-5-carbamoyl-1*H*-pyrazole-3-carboxylic Acid (**50**)

Synthesized according to General Procedure B using methyl 1-benzyl-5-carbamoyl-pyrazole-3-carboxylate (600 mg, 2.31 mmol) and LiOH·H_2_O (291 mg, 6.93 mmol) to give 1-benzyl-5-carbamoyl-1*H*-pyrazole-3-carboxylic acid (**50**, 454 mg, 1.75 mmol, 80% yield) as a colorless solid. ^1^H NMR (500 MHz, DMSO-*d*_6_) δ 12.96 (s, 1H), 8.11 (s, 1H), 7.66 (s, 1H), 7.40 (s, 1H), 7.36–7.24 (m, 3H), 7.20–7.14 (m, 2H), 5.82 (s, 2H). ACQUITY UPLC BEH C_18_ 1.7 μm: Rt = 1.23 min; *m*/*z* 244.0 [M–H]^−^.

#### *N* ^3^,1-Dibenzyl-*N* ^3^-(cyclopropylmethyl)-1*H*-pyrazole-3,5-dicarboxamide (**32**)

1-Benzyl-5-carbamoyl-1*H*-pyrazole-3-carboxylic acid (49 mg, 0.20 mmol, **50**) was heated in thionyl chloride (0.50 mL, 6.85 mmol) at 65 °C for 2 h. The mixture was concentrated under reduced pressure and dissolved in DCM (2 mL). Et_3_N (0.07 mL, 0.50 mmol) was added followed by the dropwise addition of a solution of *N*-benzyl-1-cyclopropylmethanamine (35 mg, 0.20 mmol) in DCM (2 mL). UPLC analysis after 5 min indicated reaction completion. A saturated aqueous solution of NaHCO_3_ was added and the mixture stirred vigorously for 5 min before being passed through a phase separator. The filtrate was concentrated under reduced pressure and purified by flash column chromatography (silica, 12 g, 1:0 petrol/EtOAc to 0:1 petrol/EtOAc containing 1% Et_3_N over 20 CV’s) to afford *N* ^3^,1-dibenzyl-*N* ^3^-(cyclopropylmethyl)­pyrazole-3,5-dicarboxamide (**32**, 16 mg, 0.04 mmol, 21% yield) as a colorless solid. ^1^H NMR (500 MHz, CD_3_OD) δ 7.35–7.10 (m, 11H), 5.81 (s, 1H), 5.71 (s, 1H), 5.16 (s, 1H), 3.56 (d, *J* = 6.9 Hz, 1H), 3.37–3.32 (m, 1H), 1.14–1.04 (m, 1H), 0.51–0.45 (m, 1H), 0.44–0.39 (m, 1H), 0.25–0.19 (m, 1H), 0.09–0.03 (m, 1H). Rotamers observed in approximately 1:1 ratio. One benzyl proton is missing as underneath HDO peak. ACQUITY UPLC BEH C_18_ 1.7 μm: Rt = 1.78 min; *m*/*z* 389.2 [M + H]^+^. ELSD/UV/^1^H NMR purity: 100/94/90%.

#### (*S*)-*N*-(8-Chloro-5-methyl-4-oxo-2,3,4,5-tetrahydrobenzo­[*b*]­[1,4]­oxazepin-3-yl)-4-(*N*-phenylsulfamoyl)­benzamide (**33**)

Synthesized according to General Procedure C using 4-(phenylsulfamoyl)­benzoic acid (42 mg, 0.14 mmol), (3*S*)-3-amino-8-chloro-5-methyl-2,3-dihydro-1,5-benzoxazepin-4-one hydrochloride salt (40 mg, 0.14 mmol), HOBt hydrate (24 mg, 0.16 mmol), EDC·HCl (33 mg, 0.17 mmol) and DIPEA (0.06 mL, 0.36 mmol). Purified by flash column chromatography (silica, 12 g, 1:0 petrol/EtOAc to 0:1 petrol/EtOAc containing 1% Et_3_N over 30 CV’s) to afford (*S*)-*N*-(8-chloro-5-methyl-4-oxo-2,3,4,5-tetrahydrobenzo­[*b*]­[1,4]­oxazepin-3-yl)-4-(*N*-phenylsulfamoyl)­benzamide (**33**, 66 mg, 0.13 mmol, 94% yield) as a beige solid. ^1^H NMR (500 MHz, CDCl_3_) δ 7.78–7.73 (m, 4H), 7.41 (d, *J* = 6.9 Hz, 1H), 7.26–7.21 (m, 4H), 7.17 (dd, *J* = 8.4, 0.5 Hz, 1H), 7.12 (ddt, *J* = 8.0, 6.9, 1.2 Hz, 1H), 7.09–7.06 (m, 2H), 5.07 (dt, *J* = 11.3, 7.1 Hz, 1H), 4.73 (dd, *J* = 9.8, 7.4 Hz, 1H), 4.30 (dd, *J* = 11.3, 9.9 Hz, 1H), 3.42 (s, 3H). NH not observed. ^13^C­{^1^H} NMR (101 MHz, CDCl_3_) δ 169.5 (Cq), 165.6 (Cq), 150.6 (Cq), 142.3 (Cq), 137.4 (Cq), 136.3 (Cq), 134.7 (Cq), 132.9 (Cq), 129.6 (CH), 128.1 (CH), 127.5 (CH), 126.2 (CH), 125.9 (CH), 124.3 (CH), 123.7 (CH), 121.9 (CH), 77.3 (CH_2_), 49.8 (CH), 35.9 (CH_3_). ACQUITY UPLC BEH C_18_ 1.7 μm: Rt = 1.76 min; *m*/*z* 486.2 [M + H, ^35^Cl]^+^. HRMS calculated for C_23_H_21_N_3_O_5_SCl: 486.0890, found: 486.0887 [M + H]^+^. ELSD/UV/^1^H NMR purity: 100/97/88%.

#### Dimethyl 4-Iodo-1*H*-pyrazole-3,5-dicarboxylate (**52**)

To a solution of dimethyl 1*H*-pyrazole-3,5-dicarboxylate (10.0 g, 54.3 mmol) in MeCN (1 L) under inert atmosphere were added CAN (32.7 g, 59.8 mmol) and I_2_ (17.9 g, 70.7 mmol). The reaction mixture was stirred at room temperature for 48 h. The reaction mixture was concentrated under reduced pressure, diluted with EtOAc (500 mL) and washed with 5% aq. Na_2_S_2_O_3_ solution (200 mL). The organic layer was separated, dried over NaSO_4_ and concentrated under *vacuo* to obtain the crude which was triturated with heptane (150 mL) and dried to afford dimethyl 4-iodo-1*H*-pyrazole-3,5-dicarboxylate (**52**, 15.0 g, 48.4 mmol, 89% yield) as an off-white solid. ^1^H NMR (400 MHz, CDCl_3_) δ 3.98 (s, 6H). NH not observed. ACQUITY UPLC BEH C_18_ 1.7 μm: Rt = 1.39 min; *m*/*z* 311.0 [M + H]^+^.

#### Dimethyl 1-Benzyl-4-iodo-pyrazole-3,5-dicarboxylate (**53**)

K_2_CO_3_ (3.00 g, 21.4 mmol) was added to a solution of 4-iodo-1*H*-pyrazole-3,5-dicarboxylate (5.00 g, 16.1 mmol) in DMF (30 mL). The reaction mixture was cooled to 0 °C and benzyl bromide (1.95 mL, 16.4 mmol) was slowly added over 5 min. The reaction mixture was stirred at 0 °C for 1 h, allowing to warm to room temperature. The mixture was poured onto ice-cold water (350 mL) and the resultant precipitate filtered, washed with additional cold water (100 mL) and dried thoroughly to afford dimethyl 1-benzyl-4-iodo-pyrazole-3,5-dicarboxylate (**53**, 6.10 g, 14.5 mmol, 90% yield) as a colorless solid. ^1^H NMR (500 MHz, CDCl_3_) δ 7.37–7.27 (m, 3H), 7.25–7.18 (m, 2H), 5.84 (s, 2H), 3.99 (s, 3H), 3.89 (s, 3H). ACQUITY UPLC BEH C_18_ 1.7 μm: Rt = 1.78 min; *m*/*z* 401.1 [M + H]^+^.

#### Dimethyl (*E*)-1-Benzyl-4-(2-ethoxyvinyl)-1*H*-pyrazole-3,5-dicarboxylate (**54**)

A 20 mL microwave vial was charged dimethyl 1-benzyl-4-iodo-pyrazole-3,5-dicarboxylate (2.00 g, 4.75 mmol), (*E*)-(2-ethoxyvinyl)­boronic acid pinacol ester (1.71 mL, 8.08 mmol), CsCO_3_ (3.30 g, 10.07 mmol), Pd­(dppf)_2_Cl_2_ (0.33 g, 0.45 mmol), monoglyme (20 mL) and water (2 mL). The reaction mixture was degassed with N_2_ for 10 min before heating to 90 °C and stirring overnight. The reaction mixture was diluted with EtOAc (60 mL) and filtered, separated and aqueous phase extracted with further EtOAc (20 mL). The organic phases were combined, dried over NaSO_4_, filtered and concentrated under reduced pressure with Celite. The crude mixture was purified by flash column chromatography (silica, 40 g, 1:0 petrol/EtOAc to 7:3 petrol/EtOAc over 25 CV’s). Fractions containing product were combined and concentrated under reduced pressure to afford dimethyl (*E*)-1-benzyl-4-(2-ethoxyvinyl)-1*H*-pyrazole-3,5-dicarboxylate (**54**, 1.40 g, 3.66 mmol, 77% yield) as a brown oil. ^1^H NMR (500 MHz, CDCl_3_) δ 7.40 (d, *J* = 12.9 Hz, 1H), 7.34–7.21 (m, 3H), 7.20–7.14 (m, 2H), 6.20 (d, *J* = 12.9 Hz, 1H), 5.77 (s, 2H), 3.97–3.92 (m, 5H), 3.83 (s, 3H), 1.35 (t, *J* = 7.0 Hz, 3H). ACQUITY UPLC BEH C_18_ 1.7 μm: Rt = 1.82 min; *m*/*z* 367.1 [M + Na]^+^.

#### Methyl 2,6-Dibenzyl-7-oxo-4,5,6,7-tetrahydro-2*H*-pyrazolo­[3,4-*c*]­pyridine-3-carboxylate (**55**)

Synthesized according to General Procedure A using dimethyl 1-benzyl-4-(2-oxoethyl)-1*H*-pyrazole-3,5-dicarboxylate (5 g, 15.8 mmol, **27**), benzylamine hydrochloride (2.20 g, 20.6 mmol, 1.3 equiv), acetic acid (0.3 mL) and 2-methylpyridine borane complex (2.56 g, 23.7 mmol, 1.5 equiv), MeOH (50 mL). Purified by flash column chromatography (silica, 24 g, 1:0 heptane/EtOAc to 1:1 heptane/EtOAc over 25 CV’s) to give methyl 2,6-dibenzyl-7-oxo-4,5,6,7-tetrahydro-2*H*-pyrazolo­[3,4-*c*]­pyridine-3-carboxylate (**55**, 2.50 g, 6.66 mmol, 42% yield) as a colorless solid. m.p.: 157–159 °C. ^1^H NMR (500 MHz, CDCl_3_) δ 7.40–7.23 (m, 10H), 5.83 (s, 2H), 4.78 (s, 2H), 3.85–3.81 (m, 3H), 3.48 (t, *J* = 6.7 Hz, 2H), 2.97 (t, *J* = 6.8 Hz, 2H). ^13^C­{^1^H} NMR (126 MHz, CDCl_3_) δ 160.9 (Cq), 159.9 (Cq), 142.4 (Cq), 137.3 (Cq), 136.4 (Cq), 128.8 (CH), 128.6 (CH), 128.3 (CH), 128.14 (Cq), 128.12 (CH), 128.1 (CH), 127.7 (CH), 125.9 (Cq), 56.2 (CH_2_), 52.1 (CH_3_), 49.7 (CH_2_), 46.6 (CH_2_), 21.5 (CH_2_). ACQUITY UPLC BEH C_18_ 1.7 μm: Rt = 1.78 min; *m*/*z* 376.3 [M + H]^+^. HRMS calculated for C_22_H_22_N_3_O_3_: 376.1661, found: 376.1665 [M + H]^+^. ELSD/UV/^1^H NMR purity: 100/93/99%.

#### Methyl 1,6-Dibenzyl-7-oxo-4,5,6,7-tetrahydro-1*H*-pyrazolo­[3,4-*c*]­pyridine-3-carboxylate (**56**)

Synthesized according to the synthesis of **55** through separation of the other regioisomer to give methyl 1,6-dibenzyl-7-oxo-4,5,6,7-tetrahydro-1*H*-pyrazolo­[3,4-*c*]­pyridine-3-carboxylate (**56**, 1.30 g, 3.46 mmol, 22% yield) as a colorless solid. ^1^H NMR (500 MHz, CDCl_3_) δ 7.39–7.34 (m, 2H), 7.29–7.20 (m, 8H), 5.81 (s, 2H), 4.63 (s, 2H), 3.85 (s, 3H), 3.43 (t, *J* = 6.9 Hz, 2H), 2.97 (t, *J* = 6.9 Hz, 2H). ACQUITY UPLC BEH C_18_ 1.7 μm: Rt = 1.82 min; *m*/*z* 376.3 [M + H]^+^.

#### 2,6-Dibenzyl-7-oxo-4,5,6,7-tetrahydro-2*H*-pyrazolo­[3,4-*c*]­pyridine-3-carboxamide (**34**)

Synthesized according to General Procedure C using 2,6-dibenzyl-7-oxo-4,5,6,7-tetrahydro-2*H*-pyrazolo­[3,4-*c*]­pyridine-3-carboxylic acid (150 mg, 0.41 mmol, **80**), HOBt hydrate (61 mg, 0.45 mmol, 1.1 equiv), EDC·HCl (103 mg, 0.54 mmol, 1.3 equiv), DIPEA (0.18 mL, 1.03 mmol, 2.4 equiv) and NH_4_Cl (66 mg, 1.24 mmol, 3 equiv). Purified by reverse-phase column chromatography (9:1 0.1% TFA in water/MeCN to 0:1 0.1% TFA in water/MeCN over 30 min) to afford 2,6-dibenzyl-7-oxo-4,5,6,7-tetrahydro-2*H*-pyrazolo­[3,4-*c*]­pyridine-3-carboxamide (**34**, 14 mg, 0.04 mmol, 9% yield) as an off-white solid. ^1^H NMR (500 MHz, DMSO-*d*_6_) δ 7.77 (br s, 1H), 7.62 (br s, 1H), 7.39–7.16 (m, 10H), 5.64 (s, 2H), 4.65 (s, 2H), 3.48 (t, *J* = 6.7 Hz, 2H), 2.89 (t, *J* = 6.7 Hz, 2H). ^13^C­{^1^H} NMR (101 MHz, DMSO-*d*_6_) δ 161.0, 160.0, 141.3, 137.7, 137.1, 132.9, 128.48, 127.64, 127.61, 127.57, 127.1, 125.3, 120.6, 54.0, 48.7, 46.7, 19.9. A HMBC correlation (in DMSO-*d*_6_) of amidic NH_2_ (7.62 and 7.77 ppm) with C5 (132.9 ppm) and benzyl CH_2_ (5.65 ppm) with C5 (132.9 ppm) was observed. Additionally, NOESY correlation was observed between amidic NH_2_ (7.62 and 7.77 ppm) with benzyl CH_2_ (5.65 ppm), which altogether is only consistent with isomer as drawn. ACQUITY UPLC BEH C_18_ 1.7 μm: Rt = 1.52 min; *m*/*z* 361.2 [M + H]^+^. HRMS calculated for C_21_H_21_N_4_O_2_, 361.1665, found 361.1675. ELSD/UV/^1^H NMR purity: 100/100/94%.

#### 1,6-Dibenzyl-7-oxo-4,5,6,7-tetrahydro-1*H*-pyrazolo­[3,4-*c*]­pyridine-3-carboxamide (**57**)

Synthesized according to General Procedure D using methyl 1,6-dibenzyl-7-oxo-4,5,6,7-tetrahydro-1*H*-pyrazolo­[3,4-*c*]­pyridine-3-carboxylate (150 mg, 0.40 mmol, 1 equiv) and 7 M NH_3_ in MeOH (5 mL). Purified by reverse-phase column chromatography (9:1 5 mM (NH_4_)­HCO_3_ in water/MeCN to 0:1 5 mM (NH_4_)­HCO_3_ in water/MeCN over 30 min) to 1,6-dibenzyl-7-oxo-4,5,6,7-tetrahydro-1*H*-pyrazolo­[3,4-*c*]­pyridine-3-carboxamide (**57**, 25 mg, 0.07 mmol, 17% yield) as an off-white solid. ^1^H NMR (500 MHz, CDCl_3_) δ 7.40–7.27 (m, 10H), 6.76 (s, 1H), 5.82 (s, 2H), 5.35 (s, 1H), 4.71 (s, 2H), 3.50 (t, *J* = 6.6 Hz, 2H), 3.10 (t, *J* = 6.9 Hz, 2H). ^13^C­{^1^H} NMR (100 MHz, DMSO-*d*_6_) δ 163.1, 158.0, 140.6, 137.3, 137.0, 131.9, 130.0, 128.53, 128.47, 127.49, 127.45, 127.2, 123.5, 53.8, 48.6, 47.3, 20.1. A HMBC correlation (in DMSO-*d*_6_) of benzyl CH_2_ (5.77 ppm) and C5 (131.9 ppm) was identified only and no NOESY was observed between amidic NH_2_ and benzyl CH_2_, which is only consistent with isomer as drawn. ACQUITY UPLC BEH C_18_ 1.7 μm: Rt = 1.81 min; *m*/*z* 361.2 [M + H]^+^. ELSD/UV/^1^H NMR purity: 100/89/98%.

#### Methyl 2-Benzyl-7-oxo-6-phenethyl-4,5,6,7-tetrahydro-2*H*-pyrazolo­[3,4-*c*]­pyridine-3-carboxylate (**73a**)

Synthesized according to General Procedure A using dimethyl 1-benzyl-4-(2-oxoethyl)-1*H*-pyrazole-3,5-dicarboxylate (0.90 g, 2.84 mmol, **27**), phenethylamine hydrochloride (0.67 g, 4.27 mmol, 1.5 equiv), acetic acid (0.3 mL) and 2-methylpyridine borane complex (0.27 g, 2.56 mmol, 0.9 equiv). The crude uncyclized product after workup was kept at room temperature overnight, resulting in two cyclized regioisomers. Purified by flash column chromatography (silica, 12 g, 1:0 heptane/EtOAc to 1:1 heptane/EtOAc) to give methyl 2-benzyl-7-oxo-6-phenethyl-4,5,6,7-tetrahydro-2*H*-pyrazolo­[3,4-*c*]­pyridine-3-carboxylate (**73a**, 240 mg, 0.62 mmol, 22% yield) as a pale yellow oil. X-Select LCMS CSH C_18_ 2.5 μm: Rt = 2.08 min; *m*/*z* 390.0 [M + H]^+^.

#### 2-Benzyl-7-oxo-6-phenethyl-4,5,6,7-tetrahydro-2*H*-pyrazolo­[3,4-*c*]­pyridine-3-carboxamide (**58**)

Synthesized according to General Procedure D using methyl 2-benzyl-7-oxo-6-phenethyl-4,5,6,7-tetrahydro-2*H*-pyrazolo­[3,4-*c*]­pyridine-3-carboxylate (100 mg, 0.25 mmol) and 7 M NH_3_ in MeOH (5 mL). Purified by reverse-phase column chromatography (9:1 5 mM (NH_4_)­HCO_3_ in water/MeCN to 0:1 5 mM (NH_4_)­HCO_3_ in water/MeCN over 30 min) to give 2-benzyl-7-oxo-6-phenethyl-4,5,6,7-tetrahydro-2*H*-pyrazolo­[3,4-*c*]­pyridine-3-carboxamide (**58**, 20 mg, 0.05 mmol, 21% yield) as an off-white solid. m.p.: 169–171 °C. ^1^H NMR (500 MHz, CDCl_3_) δ 7.38–7.33 (m, 2H), 7.33–7.19 (m, 8H), 5.78 (s, 2H), 5.58 (br s, 2H), 3.78 (t, *J* = 6.6 Hz, 2H), 3.37 (t, *J* = 6.6 Hz, 2H), 2.95 (t, *J* = 7.1 Hz, 2H), 2.75 (t, *J* = 6.6 Hz, 2H). ^13^C­{^1^H} NMR (126 MHz, CDCl_3_) δ 161.2 (Cq), 160.8 (Cq), 142.0 (Cq), 139.2 (Cq), 136.4 (Cq), 130.9 (CH), 129.0 (CH), 128.7 (CH), 128.3 (CH), 128.2 (CH), 126.7 (CH), 121.1 (Cq), 55.8 (CH_2_), 49.2 (CH_2_), 48.6 (CH_2_), 34.5 (CH_2_), 21.1 (CH_2_). A HMBC correlation (in DMSO-*d*_6_) of amidic NH_2_ (7.71 and 7.82 ppm) with C5 (132.8 ppm) and benzyl CH_2_ (5.69 ppm) with C5 (132.8 ppm) was observed, which is only consistent with isomer as drawn. ACQUITY UPLC BEH C_18_ 1.7 μm: Rt = 1.57 min; *m*/*z* 375.3 [M + H]^+^. HRMS calculated for C_22_H_23_N_4_O_2_: 375.1821, found: 375.1817 [M + H]^+^. ELSD/UV/^1^H NMR purity: 100/92/100%.

#### Methyl 2-Benzyl-7-oxo-6-(2-phenoxyethyl)-4,5,6,7-tetrahydro-2*H*-pyrazolo­[3,4-*c*]­pyridine-3-carboxylate (**73b**)

Synthesized according to General Procedure A but reversed limiting reagent. Dimethyl 1-benzyl-4-(2-oxoethyl)-1*H*-pyrazole-3,5-dicarboxylate (0.80 g, 2.53 mmol, **27**), 2-phenyoxyethylamine (0.50 mL, 3.79 mmol, 1.5 equiv), acetic acid (0.2 mL), 2-methylpyridine borane complex (243 mg, 2.27 mmol, 0.9 equiv). 4 M HCl in 1,4-dioxane (0.7 mL) was added to generate amine hydrochloride prior to reductive amination. The crude uncyclized product after workup was kept at room temperature overnight, resulting in two cyclized regioisomers. Purified by flash column chromatography (silica, 24 g, 7:3 heptane/EtOAc to 0:1 heptane/EtOAc) to give methyl 2-benzyl-7-oxo-6-(2-phenoxyethyl)-4,5,6,7-tetrahydro-2*H*-pyrazolo­[3,4-*c*]­pyridine-3-carboxylate (**73b**, 200 mg, 0.49 mmol, 20% yield) as an off-white solid. X-Select LCMS CSH C_18_ 1.5 μm: Rt = 2.17 min; *m*/*z* 406.2 [M + H]^+^.

#### 2-Benzyl-7-oxo-6-(2-phenoxyethyl)-4,5,6,7-tetrahydro-2*H*-pyrazolo­[3,4-*c*]­pyridine-3-carboxamide (**59**)

Synthesized according to General Procedure D using methyl 2-benzyl-7-oxo-6-(2-phenoxyethyl)-4,5,6,7-tetrahydro-2*H*-pyrazolo­[3,4-*c*]­pyridine-3-carboxylate (150 mg, 0.37 mmol) and 7 M NH_3_ in MeOH (5 mL). Purified by reverse-phase column chromatography (9:1 0.1% TFA in water/MeCN to 1:9 0.1% TFA in water/MeCN over 30 min) to give 2-benzyl-7-oxo-6-(2-phenoxyethyl)-4,5,6,7-tetrahydro-2*H*-pyrazolo­[3,4-*c*]­pyridine-3-carboxamide (**59**, 20 mg, 0.05 mmol, 14% yield) as an off-white solid. m.p.: 95–97 °C. ^1^H NMR (500 MHz, DMSO-*d*_6_) δ 7.77 (s, 1H), 7.65 (s, 1H), 7.35–7.24 (m, 5H), 7.21–7.17 (m, 2H), 6.97–6.91 (m, 3H), 5.63 (s, 2H), 4.14 (t, *J* = 5.7 Hz, 2H), 3.80 (t, *J* = 5.7 Hz, 2H), 3.69 (t, *J* = 6.6 Hz, 2H), 2.91 (t, *J* = 6.6 Hz, 2H). ^13^C­{^1^H} NMR (126 MHz, CDCl_3_) δ 161.0 (Cq), 158.6 (Cq), 142.1 (Cq), 136.5 (Cq), 130.9 (Cq), 129.7 (CH), 128.7 (CH), 128.2 (CH), 128.1 (CH), 121.4 (Cq), 121.2 (CH), 114.5 (CH), 67.3 (CH_2_), 55.8 (CH_2_), 49.8 (CH_2_), 47.0 (CH_2_), 21.4 (CH_2_). A HMBC correlation of amidic NH_2_ (7.65 and 7.77 ppm) with C5 (132.9 ppm) and benzyl CH_2_ (5.63 ppm) with C5 (132.9 ppm) was observed, which is only consistent with isomer as drawn. ACQUITY UPLC BEH C_18_ 1.7 μm: Rt = 1.57 min; *m*/*z* 389.2 [M – H]^−^. HRMS calculated for C_22_H_21_N_4_O_3_: 389.1616, found: 389.1598 [M – H]^−^. ELSD/UV/^1^H NMR purity: 100/93/100%.

#### Methyl 2-Benzyl-6-(4-hydroxybenzyl)-7-oxo-4,5,6,7-tetrahydro-2*H*-pyrazolo­[3,4-*c*]­pyridine-3-carboxylate and Methyl 1-Benzyl-6-(4-hydroxybenzyl)-7-oxo-4,5,6,7-tetrahydro-1*H*-pyrazolo­[3,4-*c*]­pyridine-3-carboxylate (**73c**)

Synthesized according to General Procedure A using dimethyl 1-benzyl-4-(2-oxoethyl)-1*H*-pyrazole-3,5-dicarboxylate (0.80 g, 2.53 mmol, **27**), 4-hydroxybenzylamine (0.47 g, 3.79 mmol, 1.5 equiv), acetic acid (0.3 mL) and 2-methylpyridine borane complex (243 mg, 2.27 mmol, 0.9 equiv). 4 M HCl in 1,4-dioxane (0.7 mL) was added to generate amine hydrochloride prior to reductive amination. The crude uncyclized product after workup was kept at room temperature overnight, resulting in two cyclized regioisomers. Purified by flash column chromatography (silica, 24 g, 1:0 heptane/EtOAc to 1:1 heptane/EtOAc) to afford a mixture of two regioisomers methyl 2-benzyl-6-(4-hydroxybenzyl)-7-oxo-4,5,6,7-tetrahydro-2*H*-pyrazolo­[3,4-*c*]­pyridine-3-carboxylate and methyl 1-benzyl-6-(4-hydroxybenzyl)-7-oxo-4,5,6,7-tetrahydro-1*H*-pyrazolo­[3,4-*c*]­pyridine-3-carboxylate (**73c**, 0.60 g, 1.53 mmol, 61% yield) as a pale yellow solid. X-Select LCMS CSH C_18_ 2.5 μm: Rt = 2.32 min; *m*/*z* 392.1 [M + H]^+^, Rt = 2.43; *m*/*z* 392.1 [M + H]^+^.

#### 2-Benzyl-6-(4-hydroxybenzyl)-7-oxo-4,5,6,7-tetrahydro-2*H*-pyrazolo­[3,4-*c*]­pyridine-3-carboxamide (**60**)

Synthesized according to General Procedure D using methyl 2-benzyl-6-(4-hydroxybenzyl)-7-oxo-4,5,6,7-tetrahydro-2*H*-pyrazolo­[3,4-*c*]­pyridine-3-carboxylate (0.50 g, 1.27 mmol, as mixture of two regioisomers) and 7 M NH_3_ in MeOH (15 mL). Purified by reverse-phase column chromatography (9:1 10 mM (NH_4_)­HCO_3_ in water/MeCN to 0:1 10 mM (NH_4_)­HCO_3_ in water/MeCN over 30 min) to give 2-benzyl-6-(4-hydroxybenzyl)-7-oxo-4,5,6,7-tetrahydro-2*H*-pyrazolo­[3,4-*c*]­pyridine-3-carboxamid*e* (**60**, 28 mg, 0.07 mmol, 6% yield) as an off-white solid. m.p.: 148–149 °C. ^1^H NMR (500 MHz, DMSO-*d*_6_) δ 9.33 (s, 1H), 7.76 (s, 1H), 7.61 (s, 1H), 7.36–7.25 (m, 3H), 7.23–7.18 (m, 2H), 7.13–7.07 (m, 2H), 6.73–6.69 (m, 2H), 5.64 (s, 2H), 4.52 (s, 2H), 3.43 (t, *J* = 6.7 Hz, 2H), 2.85 (t, *J* = 6.7 Hz, 2H). ^13^C­{^1^H} NMR (126 MHz, DMSO-*d*_6_) δ 161.1 (Cq), 159.8 (Cq), 156.6 (Cq), 141.5 (Cq), 137.2 (Cq), 132.9 (Cq), 129.1 (CH), 128.5 (CH), 127.8 (Cq), 127.7 (CH), 127.6 (CH), 120.6 (Cq), 115.2 (CH), 54.0 (CH_2_), 48.1 (CH_2_), 46.4 (CH_2_), 19.9 (CH_2_). A HMBC correlation of amidic NH (7.76 ppm) with C5 (132.9 ppm) and benzyl CH_2_ (5.64 ppm) with C5 (132.9 ppm) was observed, which is only consistent with isomer as drawn. ACQUITY UPLC BEH C_18_ 1.7 μm: Rt = 1.39 min; *m*/*z* 377.2 [M + H]^+^. HRMS calculated for C_21_H_21_N_4_O_3_: 377.1614, found: 377.1599 [M + H]^+^. ELSD/UV/^1^H NMR purity: 100/100/100%.

#### Methyl 2-Benzyl-6-(4-methoxybenzyl)-7-oxo-4,5,6,7-tetrahydro-2*H*-pyrazolo­[3,4-*c*]­pyridine-3-carboxylate (**73d**)

Synthesized according to General Procedure A using dimethyl 1-benzyl-4-(2-oxoethyl)-1*H*-pyrazole-3,5-dicarboxylate (0.80 g, 2.53 mmol, **27**), 4-methoxybenzylamine (0.50 g, 3.79 mmol, 1.5 equiv), acetic acid (0.3 mL) and 2-methylpyridine borane complex (0.24 g, 2.27 mmol, 0.9 equiv). 4 M HCl in 1,4-dioxane (0.8 mL) was added to generate amine hydrochloride prior to reductive amination. The crude uncyclized product after workup was kept at room temperature overnight, resulting in two cyclized regioisomers. Purified by flash column chromatography (silica, 24 g, 1:0 heptane/EtOAc to 1:1 heptane/EtOAc) to give methyl 2-benzyl-6-(4-methoxybenzyl)-7-oxo-4,5,6,7-tetrahydro-2*H*-pyrazolo­[3,4-*c*]­pyridine-3-carboxylate (**73d**, 0.20 g, 0.49 mmol, 20% yield) as a pale yellow solid. X-Select LCMS CSH C_18_ 2.5 μm: Rt = 1.87 min; *m*/*z* 406.2 [M + H]^+^.

#### 2-Benzyl-6-(4-methoxybenzyl)-7-oxo-4,5,6,7-tetrahydro-2*H*-pyrazolo­[3,4-*c*]­pyridine-3-carboxamide (**61**)

Synthesized according to General Procedure D using methyl 2-benzyl-6-(4-methoxybenzyl)-7-oxo-4,5,6,7-tetrahydro-2*H*-pyrazolo­[3,4-*c*]­pyridine-3-carboxylate (200 mg, 0.49 mmol) and 7 M NH_3_ in MeOH (10 mL). Purified by reverse-phase column chromatography (9:1 0.1% TFA in water/MeCN to 0:1 0.1% TFA in water/MeCN over 30 min) to give 2-benzyl-6-(4-methoxybenzyl)-7-oxo-4,5,6,7-tetrahydro-2*H*-pyrazolo­[3,4-*c*]­pyridine-3-carboxamide (**61**, 14 mg, 0.04 mmol, 7% yield) as an off-white solid. m.p.: 195–196 °C. ^1^H NMR (500 MHz, DMSO-*d*_6_) δ 7.76 (s, 1H), 7.61 (s, 1H), 7.36–7.25 (m, 3H), 7.24–7.19 (m, 4H), 6.92–6.86 (m, 2H), 5.64 (s, 2H), 4.57 (s, 2H), 3.73 (s, 3H), 3.44 (t, *J* = 6.7 Hz, 2H), 2.86 (t, *J* = 6.7 Hz, 2H). ^13^C­{^1^H} NMR (126 MHz, CDCl_3_) δ 160.9 (Cq), 160.8 (Cq), 159.3 (Cq), 142.1 (Cq), 136.6 (Cq), 130.9 (Cq), 129.7 (CH), 129.3 (Cq), 128.7 (CH), 128.3 (CH), 128.1 (CH), 120.9 (Cq), 114.2 (CH), 55.8 (CH_2_), 55.4 (CH_3_), 49.0 (CH_2_), 46.3 (CH_2_), 21.2 (CH_2_). ACQUITY UPLC BEH C_18_ 1.7 μm: Rt = 1.54 min; *m*/*z* 391.3 [M + H]^+^. HRMS calculated for C_22_H_23_N_4_O_3_: 391.1770, found: 391.1765 [M + H]^+^. ELSD/UV/^1^H NMR purity: 100/95/100%.

#### Methyl 2-Benzyl-6-(3-hydroxyphenethyl)-7-oxo-4,5,6,7-tetrahydro-2*H*-pyrazolo­[3,4-*c*]­pyridine-3-carboxylate and Methyl 1-Benzyl-6-(3-hydroxyphenethyl)-7-oxo-4,5,6,7-tetrahydro-1*H*-pyrazolo­[3,4-*c*]­pyridine-3-carboxylate (**73e**)

Synthesized according to General Procedure A using dimethyl 1-benzyl-4-(2-oxoethyl)-1*H*-pyrazole-3,5-dicarboxylate (0.80 g, 2.53 mmol, **27**), 3-(2-aminoethyl)­phenol (0.52 mL, 3.79 mmol, 1.5 equiv), acetic acid (0.3 mL) and 2-methylpyridine borane complex (0.24 g, 2.27 mmol, 0.9 equiv). 4 M HCl in 1,4-dioxane (0.7 mL) was added to generate amine hydrochloride prior to reductive amination. The crude uncyclized product after workup was kept at room temperature overnight, resulting in two cyclized regioisomers. Purified by flash column chromatography (silica, 24 g, 1:0 heptane/EtOAc to 3:7 heptane/EtOAc) to give methyl 2-benzyl-6-(3-hydroxyphenethyl)-7-oxo-4,5,6,7-tetrahydro-2*H*-pyrazolo­[3,4-*c*]­pyridine-3-carboxylate and methyl 1-benzyl-6-(3-hydroxyphenethyl)-7-oxo-4,5,6,7-tetrahydro-1*H*-pyrazolo­[3,4-*c*]­pyridine-3-carboxylate (**73e**, 0.40 g, 0.98 mmol, 39% yield) as a pale yellow solid. X-Select LCMS CSH C_18_ 2.5 μm: Rt = 1.13 min; *m*/*z* 406.3 [M + H]^+^, Rt = 1.16 min, *m*/*z* 406.3 [M + H]^+^.

#### 2-Benzyl-6-(3-hydroxyphenethyl)-7-oxo-4,5,6,7-tetrahydro-2*H*-pyrazolo­[3,4-*c*]­pyridine-3-carboxamide (**62**)

Synthesized according to General Procedure D using methyl 2-benzyl-6-(3-hydroxyphenethyl)-7-oxo-4,5,6,7-tetrahydro-2*H*-pyrazolo­[3,4-*c*]­pyridine-3-carboxylate (400 mg, 0.98 mmol, as mixture of two regioisomers) and 7 M NH_3_ in MeOH (15 mL). Purified by reverse-phase column chromatography (9:1 5 mM (NH_4_)­HCO_3_ in water/MeCN to 0:1 5 mM (NH_4_)­HCO_3_ in water/MeCN over 30 min) to give 2-benzyl-6-(3-hydroxyphenethyl)-7-oxo-4,5,6,7-tetrahydro-2*H*-pyrazolo­[3,4-*c*]­pyridine-3-carboxamide (**62**, 30 mg, 0.08 mmol, 8% yield) as an off-white solid. m.p.: 184–186 °C. ^1^H NMR (500 MHz, DMSO-*d*_6_) δ 9.27 (s, 1H), 7.76 (s, 1H), 7.65 (s, 1H), 7.35–7.25 (m, 3H), 7.21–7.17 (m, 2H), 7.07 (t, *J* = 7.7 Hz, 1H), 6.67–6.63 (m, 2H), 6.59 (ddd, *J* = 8.0, 2.5, 1.0 Hz, 1H), 5.63 (s, 2H), 3.64–3.57 (m, 2H), 3.48 (t, *J* = 6.6 Hz, 2H), 2.84 (t, *J* = 6.6 Hz, 2H), 2.73 (t, *J* = 7.5 Hz, 2H). ^13^C­{^1^H} NMR (101 MHz, DMSO-*d*_6_) δ 161.1 (Cq), 159.6 (Cq), 157.3 (Cq), 141.6 (Cq), 140.5 (Cq), 137.2 (Cq), 132.8 (Cq), 129.3 (CH), 128.5 (CH), 127.61 (CH), 127.57 (CH), 120.6 (Cq), 119.3 (CH), 115.5 (CH), 113.2 (CH), 54.0 (CH_2_), 47.6 (CH_2_), 47.3 (CH_2_), 33.5 (CH_2_), 20.0 (CH_2_). A HMBC correlation of amidic NH_2_ (7.65 and 7.76 ppm) with C5 (132.8 ppm) was observed, similarly benzyl CH_2_ (5.63 ppm) with C5 (132.8 ppm) and lactam CH_2_ (2.84 ppm) with C5 (132.8 ppm) was observed, which is only consistent with isomer as drawn. ACQUITY UPLC BEH C_18_ 1.7 μm: Rt = 1.44 min; *m*/*z* 391.3 [M + H]^+^. HRMS calculated for C_22_H_23_N_4_O_3_: 391.1770, found: 391.1770 [M + H]^+^. ELSD/UV/^1^H NMR purity: 100/87/97%.

#### Methyl 2-Benzyl-6-(3-methoxyphenethyl)-7-oxo-4,5,6,7-tetrahydro-2*H*-pyrazolo­[3,4-*c*]­pyridine-3-carboxylate (**73f**)

Synthesized according to General Procedure A using dimethyl 1-benzyl-4-(2-oxoethyl)-1*H*-pyrazole-3,5-dicarboxylate (0.80 g, 2.53 mmol, **27**), 3-methoxyphenethylamine (0.55 mL, 3.79 mmol, 1.5 equiv), acetic acid (0.3 mL) and 2-methylpyridine borane complex (0.24 g, 2.27 mmol, 0.9 equiv). 4 M HCl in 1,4-dioxane (0.7 mL) was added to generate amine hydrochloride prior to reductive amination. The crude uncyclized product after workup was kept at room temperature overnight, resulting in two cyclized regioisomers. Purified by flash column chromatography (silica, 24 g, 7:3 heptane/EtOAc to 1:4 heptane/EtOAc) to give methyl 2-benzyl-6-(3-methoxyphenethyl)-7-oxo-4,5,6,7-tetrahydro-2*H*-pyrazolo­[3,4-*c*]­pyridine-3-carboxylate (**73f**, 0.20 g, 0.48 mmol, 19% yield) as a brown oil. X-Select LCMS CSH C_18_ 2.5 μm: Rt = 2.65 min; *m*/*z* 420.2 [M + H]^+^.

#### 2-Benzyl-6-(3-methoxyphenethyl)-7-oxo-4,5,6,7-tetrahydro-2*H*-pyrazolo­[3,4-*c*]­pyridine-3-carboxamide (**63**)

Synthesized according to General Procedure D using methyl 2-benzyl-6-(3-methoxyphenethyl)-7-oxo-4,5,6,7-tetrahydro-2*H*-pyrazolo­[3,4-*c*]­pyridine-3-carboxylate (200 mg, 0.47 mmol) and 7 M NH_3_ in MeOH (10 mL). Purified by reverse-phase column chromatography (9:1 10 mM (NH_4_)­HCO_3_ in water/MeCN to 0:1 10 mM (NH_4_)­HCO_3_ in water/MeCN over 30 min) to give 2-benzyl-6-(3-methoxyphenethyl)-7-oxo-4,5,6,7-tetrahydro-2*H*-pyrazolo­[3,4-*c*]­pyridine-3-carboxamide (**63**, 21 mg, 0.05 mmol, 11% yield) as an off-white solid. m.p.: 162–163 °C. ^1^H NMR (500 MHz, DMSO-*d*_6_) δ 7.76 (s, 1H), 7.65 (s, 1H), 7.36–7.24 (m, 3H), 7.23–7.17 (m, 3H), 6.85–6.80 (m, 2H), 6.78–6.74 (m, 1H), 5.63 (s, 2H), 3.72 (s, 3H), 3.67–3.62 (m, 2H), 3.49 (t, *J* = 6.6 Hz, 2H), 2.88–2.78 (m, 4H). ^13^C­{^1^H} NMR (126 MHz, CDCl_3_) δ 160.9 (Cq), 160.6 (Cq), 159.9 (Cq), 142.2 (Cq), 140.9 (Cq), 136.6 (Cq), 130.9 (Cq), 129.7 (CH), 128.7 (CH), 128.3 (CH), 128.1 (CH), 121.4 (CH), 121.0 (Cq), 114.6 (CH), 112.1 (CH), 55.8 (CH_2_), 55.4 (CH_3_), 49.0 (CH_2_), 48.5 (CH_2_), 34.6 (CH_2_), 21.2 (CH_2_). ACQUITY UPLC BEH C_18_ 1.7 μm: Rt = 1.57 min; *m*/*z* 405.3 [M + H]^+^. HRMS calculated for C_23_H_25_N_4_O_3_: 405.1927, found: 405.1912 [M + H]^+^. ELSD/UV/^1^H NMR purity: 100/100/98%.

#### Methyl 2-Benzyl-6-(2-methoxyethyl)-7-oxo-4,5,6,7-tetrahydro-2*H*-pyrazolo­[3,4-*c*]­pyridine-3-carboxylate (**73g**)

To a solution of dimethyl 1-benzyl-4-(2-oxoethyl)-1*H*-pyrazole-3,5-dicarboxylate (0.70 g, 2.21 mmol) in DCE (10 mL) were added 2-methoxyethylamine (0.29 mL, 3.32 mmol), Na­(OAc)_3_BH (1.17 g, 5.53 mmol) and AcOH (0.3 mL). The reaction mixture was stirred at room temperature overnight. The reaction mixture was diluted with DCM (50 mL) and washed with saturated NaHCO_3_ solution (30 mL). The organic layer was dried over NaSO_4_, filtered and concentrated under reduced pressure to obtain the crude uncyclized product which was kept at room temperature overnight, resulting in formation of two cyclized regioisomers. The crude mixture was purified by flash column chromatography (silica, 24 g, 1:0 heptane/EtOAc to 0:1 heptane/EtOAc over 25 CV’s). Fractions containing product were combined and concentrated under reduced pressure to afford methyl 2-benzyl-6-(2-methoxyethyl)-7-oxo-4,5,6,7-tetrahydro-2*H*-pyrazolo­[3,4-*c*]­pyridine-3-carboxylate (**73g**, 100 mg, 0.29 mmol, 13% yield) as a pale yellow solid. X-Select LCMS CSH C_18_ 2.5 μm: Rt = 2.80 min; *m*/*z* 344.2 [M + H]^+^.

#### 2-Benzyl-6-(2-methoxyethyl)-7-oxo-4,5,6,7-tetrahydro-2*H*-pyrazolo­[3,4-*c*]­pyridine-3-carboxamide (**64**)

Synthesized according to General Procedure D using methyl 2-benzyl-6-(2-methoxyethyl)-7-oxo-4,5,6,7-tetrahydro-2*H*-pyrazolo­[3,4-*c*]­pyridine-3-carboxylate (100 mg, 0.29 mmol) and 7 M NH_3_ in MeOH (5 mL). Purified by reverse-phase column chromatography (9:1 10 mM (NH_4_)­HCO_3_ in water/MeCN to 0:1 10 mM (NH_4_)­HCO_3_ in water/MeCN over 30 min) to give 2-benzyl-6-(2-methoxyethyl)-7-oxo-4,5,6,7-tetrahydro-2*H*-pyrazolo­[3,4-*c*]­pyridine-3-carboxamide (**64**, 15 mg, 0.05 mmol, 16% yield) as an off-white solid. m.p.: 143–145 °C. ^1^H NMR (500 MHz, DMSO-*d*_6_) δ 7.77 (s, 1H), 7.64 (s, 1H), 7.35–7.24 (m, 3H), 7.22–7.17 (m, 2H), 5.63 (s, 2H), 3.61–3.55 (m, 4H), 3.48 (t, *J* = 5.6 Hz, 2H), 3.25 (s, 3H), 2.89 (t, *J* = 6.6 Hz, 2H). ^13^C­{^1^H} NMR (126 MHz, CDCl_3_) δ 161.1 (Cq), 160.8 (Cq), 142.2 (Cq), 136.6 (Cq), 131.0 (Cq), 128.7 (CH), 128.3 (CH), 128.1 (CH), 121.2 (Cq), 71.8 (CH_2_), 59.0 (CH_3_), 55.7 (CH_2_), 49.3 (CH_2_), 46.9 (CH_2_), 21.3 (CH_2_). ACQUITY UPLC BEH C_18_ 1.7 μm: Rt = 1.32 min; *m*/*z* 329.2 [M + H]^+^. HRMS calculated for C_17_H_21_N_4_O_3_: 329.1614, found: 329.1621 [M + H]^+^. ELSD/UV/^1^H NMR purity: 100/93/100%.

#### Methyl 2-Benzyl-6-(2-(dimethylamino)-2-oxoethyl)-7-oxo-4,5,6,7-tetrahydro-2*H*-pyrazolo­[3,4-*c*]­pyridine-3-carboxylate (**73h**)

To a solution of dimethyl 1-benzyl-4-(2-oxoethyl)-1*H*-pyrazole-3,5-dicarboxylate (0.70 g, 2.21 mmol) in DCE (7 mL) were added 2-amino-*N*,*N*-dimethylacetamide (0.31 mL, 2.21 mmol), Na­(OAc)_3_BH (1.17 g, 5.53 mmol) and AcOH (0.1 mL). The reaction mixture was stirred at room temperature overnight. The reaction mixture was diluted with DCM (50 mL) and washed with saturated NaHCO_3_ solution (30 mL). The organic layer was dried over NaSO_4_, filtered and concentrated under reduced pressure to obtain the crude uncyclized product which was kept at room temperature overnight, resulting in formation of two cyclized regioisomers. The crude mixture was purified by flash column chromatography (silica, 24 g, 1:0 DCM/MeOH to 49:1 DCM/MeOH over 25 CV’s). Fractions containing product were combined and concentrated under reduced pressure to afford methyl 2-benzyl-6-(2-(dimethylamino)-2-oxoethyl)-7-oxo-4,5,6,7-tetrahydro-2*H*-pyrazolo­[3,4-*c*]­pyridine-3-carboxylate (**73h**, 0.42 g, 1.13 mmol, 51% yield) as an off-white solid. X-Bridge LCMS BEH C_18_ 2.5 μm: Rt = 1.17 min; *m*/*z* 371.1 [M + H]^+^.

#### 2-Benzyl-6-(2-(dimethylamino)-2-oxoethyl)-7-oxo-4,5,6,7-tetrahydro-2*H*-pyrazolo­[3,4-*c*]­pyridine-3-carboxamide (**65**)

Synthesized according to General Procedure D using methyl 2-benzyl-6-(2-(dimethylamino)-2-oxoethyl)-7-oxo-4,5,6,7-tetrahydro-2*H*-pyrazolo­[3,4-*c*]­pyridine-3-carboxylate (0.40 g, 1.08 mmol) and 7 M NH_3_ in MeOH (5 mL). Purified by reverse-phase column chromatography (9:1 5 mM (NH_4_)­HCO_3_ in water/MeCN to 0:1 5 mM (NH_4_)­HCO_3_ in water/MeCN over 30 min) to give 2-benzyl-6-(2-(dimethylamino)-2-oxoethyl)-7-oxo-4,5,6,7-tetrahydro-2*H*-pyrazolo­[3,4-*c*]­pyridine-3-carboxamide (**65**, 15 mg, 0.04 mmol, 6% yield) as an off-white solid. m.p.: 160–161 °C. ^1^H NMR (500 MHz, DMSO-*d*_6_) δ 7.78 (s, 1H), 7.67 (s, 1H), 7.35–7.25 (m, 3H), 7.22–7.18 (m, 2H), 5.64 (s, 2H), 4.31 (s, 2H), 3.56 (t, *J* = 6.7 Hz, 2H), 2.98 (s, 3H), 2.92 (t, *J* = 6.7 Hz, 2H), 2.83 (s, 3H). ^13^C­{^1^H} NMR (126 MHz, CDCl_3_) δ 167.9 (Cq), 161.0 (Cq), 141.7 (Cq), 136.6 (Cq), 131.1 (Cq), 128.7 (CH), 128.2 (CH), 128.1 (CH), 121.7 (Cq), 55.8 (CH_2_), 48.5 (CH_2_), 47.6 (CH_2_), 36.7 (CH_3_), 35.8 (CH_3_), 21.1 (CH_2_). ACQUITY UPLC BEH C_18_ 1.7 μm: Rt = 1.24 min; *m*/*z* 354.1 [M-H]^−^. HRMS calculated for C_18_H_20_N_5_O_3_: 354.1566, found: 354.1561 [M-H]^−^. ELSD/UV/^1^H NMR purity: 100/92/100%.

#### Methyl 2-Benzyl-6-(3-hydroxy-1-phenylpropyl)-7-oxo-4,5,6,7-tetrahydro-2*H*-pyrazolo­[3,4-*c*]­pyridine-3-carboxylate (**73i**)

Synthesized in two steps according to (i) General Procedure A using dimethyl 1-benzyl-4-(2-oxoethyl)-1*H*-pyrazole-3,5-dicarboxylate (1.50 g, 4.74 mmol, **27**), 3-amino-3-phenyl-1-propanol (0.93 g, 6.17 mmol, 1.3 equiv), acetic acid (0.2 mL) and 2-methylpyridine borane complex (0.46 g, 4.27 mmol, 0.9 equiv). Purified by flash column chromatography (silica, 24 g, 1:0 DCM/MeOH to 9:1 DCM/MeOH) to afford dimethyl 1-benzyl-4-(2-((3-hydroxy-1-phenylpropyl)­amino)­ethyl)-1*H*-pyrazole-3,5-dicarboxylate (0.90 g, 1.99 mmol, 42% yield) as a yellow liquid. Product taken forward as crude to the next step. (ii) To a solution of dimethyl 1-benzyl-4-(2-((3-hydroxy-1-phenylpropyl)­amino)­ethyl)-1*H*-pyrazole-3,5-dicarboxylate (0.30 g, 0.66 mmol) in toluene (5 mL) was added AlMe_3_ (2 M in toluene, 1.10 mL, 2.25 mmol). The reaction mixture was heated to 100 °C and stirred for 3 h. The reaction mixture was cooled to room temperature, quenched with saturated Rochelle salt solution (60 mL) and extracted with EtOAc (2 × 70 mL). The organic layers were combined, dried over NaSO_4_, filtered and concentrated under reduced pressure. The crude mixture was purified by flash column chromatography (silica, 12 g, 1:0 heptane/EtOAc to 3:7 heptane/EtOAc over 25 CV’s). Fractions containing product were combined and concentrated under reduced pressure to afford methyl 2-benzyl-6-(3-hydroxy-1-phenylpropyl)-7-oxo-4,5,6,7-tetrahydro-2*H*-pyrazolo­[3,4-*c*]­pyridine-3-carboxylate (**73i**, 0.15 g, 0.35 mmol, 53% yield) as a brown liquid. XSelect LCMS CSH C_18_ 2.5 μm: 2.15 min; *m*/*z* 420.3 [M + H]^+^.

#### 2-Benzyl-6-(3-hydroxy-1-phenylpropyl)-7-oxo-4,5,6,7-tetrahydro-2*H*-pyrazolo­[3,4-*c*]­pyridine-3-carboxamide (**66**)

Synthesized according to General Procedure D using methyl 2-benzyl-6-(3-hydroxy-1-phenylpropyl)-7-oxo-4,5,6,7-tetrahydro-2*H*-pyrazolo­[3,4-*c*]­pyridine-3-carboxylate (150 mg, 0.35 mmol) and 7 M NH_3_ in MeOH (5 mL). Purified by flash column chromatography (silica, 12 g, 1:0 DCM/MeOH to 19:1 DCM/MeOH over 25 CV’s) to give 2-benzyl-6-(3-hydroxy-1-phenylpropyl)-7-oxo-4,5,6,7-tetrahydro-2*H*-pyrazolo­[3,4-*c*]­pyridine-3-carboxamide (**66**, 29 mg, 0.07 mmol, 20% yield) as an off-white solid. m.p.: 182–184 °C. ^1^H NMR (500 MHz, DMSO-*d*_6_) δ 7.74 (s, 1H), 7.59 (s, 1H), 7.39–7.31 (m, 6H), 7.30–7.25 (m, 2H), 7.23–7.20 (m, 2H), 5.91 (dd, *J* = 9.6, 6.0 Hz, 1H), 5.67–5.58 (m, 2H), 4.50 (t, *J* = 5.1 Hz, 1H), 3.50–3.39 (m, 3H), 3.09–3.02 (m, 1H), 2.86–2.78 (m, 1H), 2.74–2.66 (m, 1H), 2.19–2.07 (m, 2H). ^13^C­{^1^H} NMR (100 MHz, DMSO-*d*_6_) δ 161.5 (Cq), 160.5 (Cq), 142.1 (Cq), 140.6 (Cq), 137.6 (Cq), 133.3 (Cq), 129.0 (CH), 128.9 (CH), 128.2 (CH), 128.0 (CH), 127.8 (CH), 120.9 (Cq), 58.6 (CH_2_), 54.5 (CH_2_), 51.5 (CH), 42.1 (CH_2_), 33.2 (CH_2_), 20.6 (CH_2_). A HMBC correlation of amidic NH_2_ (7.59 and 7.74 ppm) with C5 (133.3 ppm) and benzyl CH_2_ (5.58–5.67 ppm) with C5 (133.3 ppm) was observed, which is only consistent with isomer as drawn. ACQUITY UPLC BEH C_18_ 1.7 μm: Rt = 1.49 min; *m*/*z* 405.3 [M + H]^+^. HRMS calculated for C_23_H_25_N_4_O_3_: 405.1927, found 405.1929 [M + H]^+^. ELSD/UV/^1^H NMR purity: 95/95/95%.

#### 2-Benzyl-7-oxo-6-phenethyl-4,5,6,7-tetrahydro-2*H*-pyrazolo­[3,4-*c*]­pyridine-3-carboxylic acid (**67**)

Synthesized according to synthesis of **58** and isolated as a major side-product. Purified by reverse-phase column chromatography (9:1 5 mM (NH_4_)­HCO_3_ in water/MeCN to 0:1 5 mM (NH_4_)­HCO_3_ in water/MeCN over 30 min). Fractions containing product were combined and concentrated under reduced pressure to give 2-benzyl-7-oxo-6-phenethyl-4,5,6,7-tetrahydro-2*H*-pyrazolo­[3,4-*c*]­pyridine-3-carboxylic acid (**67**, 28 mg, 0.07 mmol, 30% yield) as an off-white solid. m.p.: 80 °C. ^1^H NMR (500 MHz, CDCl_3_) δ 7.36–7.19 (m, 10H), 5.78 (s, 2H), 3.84–3.77 (m, 2H), 3.39 (t, *J* = 6.8 Hz, 2H), 2.96 (t, *J* = 7.2 Hz, 2H), 2.91 (t, *J* = 6.8 Hz, 2H). OH not observed. ^13^C­{^1^H} NMR (126 MHz, CDCl_3_) δ 162.3 (Cq), 161.3 (Cq), 142.0 (Cq), 138.9 (Cq), 136.1 (Cq), 129.0 (CH), 128.8 (CH), 128.7 (CH), 128.2 (CH), 128.2 (CH), 127.9 (Cq), 127.1 (Cq), 126.8 (CH), 56.2 (CH_2_), 49.6 (CH_2_), 48.8 (CH_2_), 34.4 (CH_2_), 21.2 (CH_2_). The structure was assigned based on structural confirmation by HMBC of **58**. ACQUITY UPLC BEH C_18_ 1.7 μm: Rt = 1.63 min; *m*/*z* 376.3 [M + H]^+^. HRMS calculated for C_22_H_22_N_3_O_3_: 376.1661, found: 376.1653 [M + H]^+^. ELSD/UV/^1^H NMR purity: 100/91/98%.

#### 2-((2-Methoxyethyl)­amino)­benzonitrile (**75**)

To a stirred solution of 2-fluorobenzonitrile (5.00 g, 41.3 mmol) in MeCN (50 mL) was added 2-methoxyethylamine (7.18 mL, 82.6 mmol). The reaction mixture was heated to 100 °C and stirred overnight in a sealed tube. The reaction mixture was cooled to room temperature and concentrated under reduced pressure. The crude mixture was purified by flash column chromatography (silica, 24 g, 1:0 heptane/EtOAc to 9:1 heptane/EtOAc over 25 CV’s). Fractions containing product were combined and concentrated under reduced pressure to afford 2-((2-methoxyethyl)­amino)­benzonitrile (**75**, 2.50 g, 14.19 mmol, 34% yield) as a colorless liquid. ^1^H NMR (400 MHz, CDCl_3_) δ 7.42–7.35 (m, 2H), 6.72–6.64 (m, 2H), 4.86 (br s, 1H), 3.62 (t, *J* = 5.3 Hz, 2H), 3.41 (s, 3H), 3.39–3.34 (m, 2H). X-Select LCMS CSH C_18_ 2.5 μm: Rt = 1.43 min; *m*/*z* 177.0 [M + H]^+^.

#### 2-(Amino­(cyclopropyl)­methyl)-*N*-(2-methoxyethyl)­aniline (**76**)

To a stirred solution of 2-((2-methoxyethyl)­amino)­benzonitrile (0.30 g, 1.70 mmol) in THF (3 mL) was added cyclopropylmagnesium bromide (0.5 M in THF, 10 mL, 5.11 mmol). The reaction mixture was irradiated in a microwave at 100 °C for 15 min. After completion of the reaction, NaBH_4_ (0.32 g, 8.52 mmol) in MeOH (6 mL) was added at 0 °C. The reaction mixture was stirred at 0 °C for 30 min, allowing to warm to room temperature. The reaction mixture was diluted with water (5 mL) and extracted with EtOAc (2 × 5 mL). The organic layers were combined, dried over NaSO_4_, filtered and concentrated under reduced pressure. The crude mixture was purified by flash column chromatography (silica, 12 g, 1:0 heptane/EtOAc to 3:2 heptane/EtOAc over 25 CV’s). Fractions containing product were combined and concentrated under reduced pressure to afford 2-(amino­(cyclopropyl)­methyl)-*N*-(2-methoxyethyl)­aniline (**76**, 0.70 g, 3.18 mmol, 47% yield) as a yellow liquid. ^1^H NMR (400 MHz, CDCl_3_) δ 7.23–7.13 (m, 2H), 6.71–6.61 (m, 2H), 3.66 (t, *J* = 5.4 Hz, 3H), 3.40 (s, 4H), 3.36–3.28 (m, 3H), 3.07 (br d, *J* = 9.3 Hz, 1H), 1.47–1.41 (m, 1H), 0.66–0.52 (m, 2H), 0.30–0.20 (m, 2H).

#### Dimethyl 1-Benzyl-4-(2-((cyclopropyl­(phenyl)­methyl)­amino)­ethyl)-1*H*-pyrazole-3,5-dicarboxylate (**77a**)

Synthesized according to General Procedure A but reversed limiting reagent. Dimethyl 1-benzyl-4-(2-oxoethyl)-1*H*-pyrazole-3,5-dicarboxylate (1.11 g, 3.53 mmol, 1.3 eq, **27**), cyclopropyl­(phenyl)­methanamine hydrochloride (0.40 g, 2.72 mmol), AcOH (0.3 mL), 2-methylpyridine borane complex (0.44 g, 4.08 mmol, 1.5 equiv). Purified by flash column chromatography (silica, 24 g, 1:0 heptane/EtOAc to 0:1 heptane/EtOAc over 25 CV’s) to give dimethyl 1-benzyl-4-(2-((cyclopropyl­(phenyl)­methyl)­amino)­ethyl)-1*H*-pyrazole-3,5-dicarboxylate (**77a**, 0.55 g, 1.23 mmol, 45% yield) as a yellow gum. ^1^H NMR (400 MHz, CDCl_3_) δ 7.32–7.16 (m, 10H), 5.77 (s, 2H), 3.92 (s, 3H), 3.76–3.70 (m, 3H), 3.28–3.14 (m, 2H), 2.91 (d, *J* = 8.8 Hz, 1H), 2.73–2.61 (m, 2H), 2.06 (s, 1H), 1.11–1.02 (m, 1H), 0.62–0.55 (m, 1H), 0.43–0.35 (m, 1H), 0.32–0.27 (m, 1H), 0.24–0.15 (m, 1H).

#### 1-Benzyl-4-(2-((cyclopropyl­(phenyl)­methyl)­amino)­ethyl)-1*H*-pyrazole-3,5-dicarboxylic Acid (**78a**)

Synthesized according to General Procedure B using dimethyl 1-benzyl-4-(2-((cyclopropyl­(phenyl)­methyl)­amino)­ethyl)-1*H*-pyrazole-3,5-dicarboxylate (0.55 g, 1.23 mmol) and LiOH·H_2_O (0.26 g, 6.15 mmol, 5 equiv) to give 1-benzyl-4-(2-((cyclopropyl­(phenyl)­methyl)­amino)­ethyl)-1*H*-pyrazole-3,5-dicarboxylic acid (**78a**, 0.40 g, 0.95 mmol, 78% yield) as an off-white solid. ^1^H NMR (400 MHz, DMSO-*d*_6_) δ 12.92 (br s, 1H), 9.94 (br s, 1H), 7.46–7.17 (m, 10H), 5.83–5.63 (m, 2H), 3.56–3.46 (m, 1H), 3.23–3.20 (m, 2H), 3.12–3.00 (m, 1H), 2.96–2.81 (m, 1H), 1.30–1.15 (m, 1H), 0.70–0.50 (m, 1H), 0.45–0.34 (m, 1H), 0.19–0.09 (m, 1H), 0.01 to −0.18 (m, 1H). CH not observed as overlapping with HDO peak. X-Select LCMS BEH C_18_ 2.5 μm: Rt = 0.74 min; *m*/*z* 420.0 [M + H]^+^.

#### 2-Benzyl-6-(cyclopropyl­(phenyl)­methyl)-7-oxo-4,5,6,7-tetrahydro-2*H*-pyrazolo­[3,4-*c*]­pyridine-3-carboxamide (**68**)

Synthesized according to General Procedure C using 1-benzyl-4-(2-((cyclopropyl­(phenyl)­methyl)­amino)­ethyl)-1*H*-pyrazole-3,5-dicarboxylic acid (400 mg, 0.95 mmol), HOBt hydrate (142 mg, 1.04 mmol, 1.1 equiv), EDC·HCl (237 mg, 1.23 mmol, 1.3 equiv), DIPEA (0.40 mL, 2.38 mmol, 2.5 equiv) and NH_4_Cl (254 mg, 4.75 mmol, 5 equiv). Purified by reverse-phase column chromatography (9:1 5 mM (NH_4_)­HCO_3_ in water/MeCN to 0:1 5 mM (NH_4_)­HCO_3_ in water/MeCN over 30 min) to afford *rac*-2-benzyl-6-(cyclopropyl­(phenyl)­methyl)-7-oxo-4,5,6,7-tetrahydro-2*H*-pyrazolo­[3,4-*c*]­pyridine-3-carboxamide (**68**) as a colorless solid. ^1^H NMR (500 MHz, DMSO-*d*_6_) δ 7.78 (s, 1H), 7.63 (s, 1H), 7.49–7.19 (m, 10H), 5.68–5.58 (m, 2H), 5.00–4.90 (m, 1H), 3.65–3.54 (m, 1H), 2.94–2.72 (m, 2H), 1.58–1.44 (m, 1H), 1.30–1.18 (m, 1H), 0.90–0.74 (m, 1H), 0.60–0.42 (m, 1H), 0.37–0.27 (m, 1H). 1H for CH_2_ for one enantiomer not observed as overlapping with HDO peak. ACQUITY UPLC BEH C_18_ 1.7 μm: Rt = 1.64 min, *m*/*z* 401.3 [M + H]^+^. HRMS calculated for C_24_H_25_N_4_O_2_: 401.1978, found: 401.1970 [M + H]^+^. ELSD/UV/^1^H NMR purity: 100/96/98%.

#### (*R*)-2-Benzyl-6-(cyclopropyl­(phenyl)­methyl)-7-oxo-4,5,6,7-tetrahydro-2*H*-pyrazolo­[3,4-*c*]­pyridine-3-carboxamide (MDI-117740, **69**)

Racemate **68** was resolved by chiral separation (Daicel CHIRALPAK IG 250 mm × 4.6 mm, 5 μm, 7:3 0.1% DEA in *n*-hexane/1:1 DCM/MeOH, flow rate: 1 mL/min) to afford (*R*)-2-benzyl-6-(cyclopropyl­(phenyl)­methyl)-7-oxo-4,5,6,7-tetrahydro-2*H*-pyrazolo­[3,4-*c*]­pyridine-3-carboxamide (**69**, 130 mg, 0.32 mmol, 34% yield) as a colorless solid. m.p.: 203–205 °C. ^1^H NMR (500 MHz, CDCl_3_) δ 7.46–7.43 (m, 2H, H26,30), 7.33–7.30 (m, 2H, H16,20), 7.29–7.20 (m, 6H, H17–19, H27–29), 5.79–5.72 (m, 2H, H24), 5.43 (s, 2H, NH_2_), 5.19 (d, *J* = 10.3 Hz, 1H, H14), 3.63–3.55 (m, 1H, H5′), 3.30–3.22 (m, 1H, H5), 2.82–2.70 (m, 2H, H4,4’), 1.32–1.23 (m, 1H, H21), 0.84–0.78 (m, 1H, H22’/23’), 0.65–0.58 (m, 1H, H22’/23’), 0.54–0.47 (m, 2H, H22’,H23’). ^13^C­{^1^H} NMR (126 MHz, CDCl_3_) δ 160.86 (C1/C10), 160.80 (C1/C10) 142.4 (C2), 140.3 (C15), 136.6 (C25), 130.7 (C9), 128.7 (C27,29), 128.6 (C17,19), 128.4 (C16,20), 128.2 (C18), 127.7 (C26,30), 127.5 (C28), 120.9 (C3), 59.8 (C14), 55.9 (C24), 42.5 (C5), 21.6 (C4), 12.4 (C21), 6.3 (C22/23), 3.5 (C22/23). Absolute stereochemistry confirmed by small X-ray crystal structure analysis. ACQUITY UPLC BEH C_18_ 1.7 μm: Rt = 1.67 min; *m*/*z* 401.3 [M + H]^+^. HRMS calculated for C_24_H_25_N_4_O_2_: 401.1978, found: 401.1981 [M + H]^+^. ELSD/UV/^1^H NMR purity: 100/94/96%.

#### (*S*)-2-Benzyl-6-(cyclopropyl­(phenyl)­methyl)-7-oxo-4,5,6,7-tetrahydro-2*H*-pyrazolo­[3,4-*c*]­pyridine-3-carboxamide (**70**)

Synthesized according to the synthesis of **68** through separation of the other enantiomer to afford (*S*)-2-benzyl-6-(cyclopropyl­(phenyl)­methyl)-7-oxo-4,5,6,7-tetrahydro-2*H*-pyrazolo­[3,4-*c*]­pyridine-3-carboxamide (**70**, 20 mg, 0.05 mmol, 5% yield) as a colorless solid. m.p: 204–205 °C; ^1^H NMR (500 MHz, CDCl_3_) δ 7.54–7.49 (m, 2H), 7.41–7.27 (m, 8H), 5.88–5.77 (m, 2H), 5.46 (s, 2H), 5.27 (d, *J* = 10.3 Hz, 1H), 3.70–3.61 (m, 1H), 3.38–3.29 (m, 1H), 2.91–2.76 (m, 2H), 1.38–1.29 (m, 1H), 0.93–0.84 (m, 1H), 0.73–0.64 (m, 1H), 0.63–0.52 (m, 2H). ^13^C­{^1^H} NMR (126 MHz, CDCl_3_) δ 160.86 (Cq), 160.79 (Cq) 142.4 (Cq), 140.3 (Cq), 136.6 (Cq), 130.7 (Cq), 128.7 (CH), 128.6 (CH), 128.4 (CH), 128.2 (CH), 127.7 (CH), 127.5 (CH), 120.9 (Cq), 59.8 (CH), 55.9 (CH_2_), 42.5 (CH_2_), 21.5 (CH_2_), 12.4 (CH), 6.3 (CH_2_), 3.5 (CH_2_). Absolute stereochemistry confirmed based on single X-ray structure analysis of **69**. ACQUITY UPLC BEH C_18_ 1.7 μm: Rt = 1.66 min; *m*/*z* 401.3 [M + H]^+^. HRMS calculated for C_24_H_25_N_4_O_2_: 401.1978, found: 401.1982 [M + H]^+^. ELSD/UV/^1^H NMR purity: 100/92/93%.

#### Dimethyl 1-Benzyl-4-(2-((cyclopropyl­(2-((2-methoxyethyl)­amino)­phenyl)­methyl)­amino)­ethyl)-1*H*-pyrazole-3,5-dicarboxylate (**77b**)

Synthesized according to General Procedure A but reversed limiting reagent. Dimethyl 1-benzyl-4-(2-oxoethyl)-1*H*-pyrazole-3,5-dicarboxylate (0.55 g, 1.74 mmol, 1.1 equiv, **27**), 2-(amino­(cyclopropyl)­methyl)-*N*-(2-methoxyethyl)­aniline (0.35 g, 1.59 mmol, **76**), AcOH (0.1 mL), 2-methylpyridine borane complex (0.19 g, 1.79 mmol, 1.2 equiv). Purified by flash column chromatography (silica, 12 g, 1:0 heptane/EtOAc to 7:3 heptane/EtOAc over 25 CV’s) to give dimethyl 1-benzyl-4-(2-((cyclopropyl­(2-((2-methoxyethyl)­amino)­phenyl)­methyl)­amino)­ethyl)-1*H*-pyrazole-3,5-dicarboxylate (**77b**, 0.30 g, 0.58 mmol, 36% yield) as a yellow gum. X-Select LCMS BEH C_18_ 2.5 μm: Rt = 1.95 min; *m*/*z* 521.5 [M + H]^+^.

#### 1-Benzyl-4-(2-((cyclopropyl­(2-((2-methoxyethyl)­amino)­phenyl)­methyl)­amino)­ethyl)-1*H*-pyrazole-3,5-dicarboxylic acid (**78b**)

Synthesized according to General Procedure B using dimethyl 1-benzyl-4-(2-((cyclopropyl­(2-((2-methoxyethyl)­amino)­phenyl)­methyl)­amino)­ethyl)-1*H*-pyrazole-3,5-dicarboxylate (300 mg, 0.58 mmol) and LiOH·H_2_O (121 mg, 2.88 mmol, 5 equiv) to give 1-benzyl-4-(2-((cyclopropyl­(2-((2-methoxyethyl)­amino)­phenyl)­methyl)­amino)­ethyl)-1*H*-pyrazole-3,5-dicarboxylic acid (**78b**, 250 mg, 0.50 mmol, 88% yield) as an off-white solid. X-Select LCMS BEH C_18_ 2.5 μm: Rt = 0.88 min; *m*/*z* 533.6 [M + MeCN]^+^.

#### 2-Benzyl-6-(cyclopropyl­(2-((2-methoxyethyl)­amino)­phenyl)­methyl)-7-oxo-4,5,6,7-tetrahydro-2*H*-pyrazolo­[3,4-*c*]­pyridine-3-carboxamide (**71**)

Synthesized through two-step telescope according to General Procedure C (for both steps) using 1-benzyl-4-(2-((cyclopropyl­(2-((2-methoxyethyl)­amino)­phenyl)­methyl)­amino)­ethyl)-1*H*-pyrazole-3,5-dicarboxylic acid (250 mg, 0.50 mmol), HOBt hydrate (75 mg, 0.55 mmol, 1.1 equiv), EDC·HCl (126 mg, 0.66 mmol, 1.3 equiv) and DIPEA (0.22 mL, 1.27 mmol, 2.4 equiv).

After cyclization in the first step, NH_4_Cl (27 mg, 0.50 mmol, 5 equiv), HOBt hydrate (75 mg, 0.55 mmol, 1.1 equiv), EDC·HCl (126 mg, 0.66 mmol, 1.3 equiv) and DIPEA (0.22 mL, 1.27 mmol, 2.4 equiv) were added to the crude mixture. Purified by reverse-phase column chromatography (9:1 5 mM (NH_4_)­HCO_3_ in water/MeCN to 0:1 5 mM (NH_4_)­HCO_3_ in water/MeCN over 30 min) to give 2-benzyl-6-(cyclopropyl­(2-((2-methoxyethyl)­amino)­phenyl)­methyl)-7-oxo-4,5,6,7-tetrahydro-2*H*-pyrazolo­[3,4-*c*]­pyridine-3-carboxamide (**71**, 46 mg, 0.10 mmol, 19% yield) as a colorless solid. m.p: 97–99 °C. ^1^H NMR (500 MHz, CDCl_3_) δ 7.80 (d, *J* = 7.9 Hz, 0.26H), 7.67 (d, *J* = 2.3 Hz, 0.80H), 7.53 (d, *J* = 8.0 Hz, 0.24H), 7.49 (d, *J* = 2.3 Hz, 0.85H), 7.47–7.44 (m, 0.21H), 7.39–7.35 (m, 2.48H), 7.31–7.22 (m, 5H), 5.79–5.63 (m, 2H), 5.28–5.22 (m, 1H), 4.06–3.98 (m, 0.40H), 3.94–3.85 (m, 1.87H), 3.78–3.68 (m, 0.74H), 3.62–3.52 (m, 0.93H), 3.42–3.28 (m, 2.49H), 3.24 (s, 2.33H), 3.20 (s, 0.73H), 2.86 (d, *J* = 12.6 Hz, 1.27H), 1.49–1.38 (m, 1H), 0.92–0.81 (m, 2H), 0.79–0.73 (m, 1H), 0.48–0.40 (m, 1H). Atropisomers observed in approximately 3:1 ratio. ^13^C­{^1^H} NMR (126 MHz, CDCl_3_) δ 160.9 (Cq), 142.3 (Cq), 136.6 (Cq), 130.6 (Cq), 129.1 (CH), 128.7 (CH), 128.49 (CH), 128.47 (CH), 128.2 (CH), 121.0 (Cq), 70.9 (CH_2_), 58.9 (CH_3_), 56.3 (CH), 55.8 (CH_2_), 42.1 (CH_2_), 21.4 (CH_2_), 12.6 (CH), 5.0 (CH_2_), 4.8 (CH_2_). CH_2_ and 2 x Cq not observed. ACQUITY UPLC BEH C_18_ 1.7 μm: Rt = 1.69 min; *m*/*z* 474.3 [M + H]^+^. HRMS calculated for C_27_H_32_N_5_O_3_: 474.2505, found: 474.2505 [M + H]^+^. ELSD/UV/^1^H NMR purity: 100/93/92%.

#### Dimethyl 1-Benzyl-4-(2-((2-((*tert-*butoxycarbonyl)­amino)-1-phenylethyl)­amino)­ethyl)-1*H*-pyrazole-3,5-dicarboxylate (**77c**)

Synthesized according to General Procedure A using dimethyl 1-benzyl-4-(2-oxoethyl)-1*H*-pyrazole-3,5-dicarboxylate (0.40 g, 1.26 mmol, **27**), *tert*-butyl (2-amino-2-phenylethyl)­carbamate (0.30 g, 1.26 mmol, 1 equiv), AcOH (0.1 mL) and 2-methylpyridine borane complex (0.14 g, 1.26 mmol, 1 equiv). Purified by flash column chromatography (silica, 12 g, 1:0 heptane/EtOAc to 6:4 heptane/EtOAc over 25 CV’s) to give dimethyl 1-benzyl-4-(2-((2-((tert-butoxycarbonyl)­amino)-1-phenylethyl)­amino)­ethyl)-1*H*-pyrazole-3,5-dicarboxylate (**77c**, 0.15 g, 0.27 mmol, 22% yield) as a pale-yellow gum. X-Select LCMS BEH C_18_ 2.5 μm: Rt = 1.56 min; *m*/*z* 537.2 [M + H]^+^.

#### 1-Benzyl-4-(2-((2-((*tert*-butoxycarbonyl)­amino)-1-phenylethyl)­amino)­ethyl)-1*H*-pyrazole-3,5-dicarboxylic Acid (**78c**)

Synthesized according to General Procedure B using dimethyl 1-benzyl-4-(2-((2-((*tert*-butoxycarbonyl)­amino)-1-phenylethyl)­amino)­ethyl)-1*H*-pyrazole-3,5-dicarboxylate (150 mg, 0.27 mmol) and LiOH·H_2_O (35 mg, 0.83 mmol, 3 equiv) to give 1-benzyl-4-(2-((2-((tert-butoxycarbonyl)­amino)-1-phenylethyl)­amino)­ethyl)-1*H*-pyrazole-3,5-dicarboxylic acid (**78c**, 130 mg, 0.26 mmol, 91% yield) as an off-white solid. X-Select LCMS BEH C_18_ 2.5 μm: Rt = 0.70 min; *m*/*z* 509.6 [M + H]^+^.

#### *tert-*Butyl (2-(2-Benzyl-3-carbamoyl-7-oxo-2,4,5,7-tetrahydro-6*H*-pyrazolo­[3,4-*c*]­pyridin-6-yl)-2-phenylethyl)­carbamate (**79**)

Synthesized through two-step telescope according to General Procedure C (for both steps) using 1-benzyl-4-(2-((2-(*(tert*-butoxycarbonyl)­amino)-1-phenylethyl)­amino)­ethyl)-1*H*-pyrazole-3,5-dicarboxylic acid (130 mg, 0.25 mmol), HOBt hydrate (37 mg, 0.28 mmol, 1.1 equiv), EDC·HCl (63 mg, 0.33 mmol, 1.3 equiv) and DIPEA (0.11 mL, 0.61 mmol, 2.4 equiv).

After cyclization in the first step, NH_4_Cl (68 mg, 1.27 mmol, 5 equiv), HOBt hydrate (37 mg, 0.28 mmol, 1.1 equiv), EDC·HCl (63 mg, 0.33 mmol, 1.3 equiv) and DIPEA (0.11 mL, 0.61 mmol, 2.4 equiv) were added to the crude mixture. Purified by reverse-phase column chromatography (9:1 5 mM (NH_4_)­HCO_3_ in water/MeCN to 0:1 5 mM (NH_4_)­HCO_3_ in water/MeCN over 30 min) to give tert-butyl (2-(2-benzyl-3-carbamoyl-7-oxo-2,4,5,7-tetrahydro-6*H*-pyrazolo­[3,4-*c*]­pyridin-6-yl)-2-phenylethyl)­carbamate (**79**, 60 mg, 0.12 mmol, 48% yield) as a colorless solid. X-Select LCMS BEH C_18_ 2.5 μm: Rt = 1.33 min; *m*/*z* 488.0 [M – H]^−^.

#### 6-(2-Amino-1-phenylethyl)-2-benzyl-7-oxo-4,5,6,7-tetrahydro-2*H*-pyrazolo­[3,4-*c*]­pyridine-3-carboxamide Hydrochloride (**80**)

To a stirred solution of *tert*-butyl (2-(2-benzyl-3-carbamoyl-7-oxo-2,4,5,7-tetrahydro-6*H*-pyrazolo­[3,4-*c*]­pyridin-6-yl)-2-phenylethyl)­carbamate (50 mg, 0.12 mmol) in DCM (1 mL) was added 4 M HCl in 1,4 dioxane (1 mL). The reaction mixture was stirred at room temperature for 2 h. The reaction mixture was concentrated under reduced pressure to afford 6-(2-amino-1-phenylethyl)-2-benzyl-7-oxo-4,5,6,7-tetrahydro-2*H*-pyrazolo­[3,4-*c*]­pyridine-3-carboxamide (**80**, 50 mg, 0.12 mmol) as a crude off-white solid, which was taken forward to the next step.

#### *N*-(2-(2-Benzyl-3-carbamoyl-7-oxo-2,4,5,7-tetrahydro-6*H*-pyrazolo­[3,4-*c*]­pyridin-6-yl)-2-phenylethyl)­thiazole-5-carboxamide (**72**)

Synthesized according to General Procedure C using 6-(2-amino-1-phenylethyl)-2-benzyl-7-oxo-4,5,6,7-tetrahydro-2*H*-pyrazolo­[3,4-*c*]­pyridine-3-carboxamide hydrochloride (50 mg, 0.12 mmol), thiazole-5-carboxylic acid (16 mg, 0.12 mmol, 1 equiv), HOBt hydrate (19 mg, 0.14 mmol, 1.1 equiv), EDC·HCl (31 mg, 0.16 mmol, 1.3 equiv) and DIPEA (66 μL, 0.38 mmol, 2.4 equiv). Purified by reverse-phase column chromatography (9:1 5 mM (NH_4_)­HCO_3_ in water/MeCN to 0:1 5 mM (NH_4_)­HCO_3_ in water/MeCN over 30 min) to give *N*-(2-(2-benzyl-3-carbamoyl-7-oxo-2,4,5,7-tetrahydro-6*H*-pyrazolo­[3,4-*c*]­pyridin-6-yl)-2-phenylethyl)­thiazole-5-carboxamide (**72**, 27 mg, 0.05 mmol, 42% yield) as an off-white solid. m.p.: 143–145 °C. ^1^H NMR (500 MHz, DMSO-*d*_6_) δ 9.20 (d, *J* = 0.7 Hz, 1H), 8.89 (t, *J* = 5.9 Hz, 1H), 8.36 (d, *J* = 0.7 Hz, 1H), 7.74 (s, 1H), 7.61 (s, 1H), 7.35–7.41 (m, 4H), 7.29–7.33 (m, 3H), 7.25–7.28 (m, 1H), 7.17–7.20 (m, 2H), 6.05 (t, *J* = 7.6 Hz, 1H), 5.65 (d, *J* = 14.6 Hz, 1H), 5.57 (d, *J* = 14.7 Hz, 1H), 3.91 (dd, *J* = 7.7, 5.9 Hz, 2H), 3.61 (ddd, *J* = 13.0, 8.4, 5.1 Hz, 1H), 3.09 (ddd, *J* = 12.5, 7.3, 5.3 Hz, 1H), 2.84 (ddd, *J* = 15.9, 7.3, 5.1 Hz), 2.70 (ddd, *J* = 15.8, 8.4, 5.2 Hz, 1H). ^13^C­{^1^H} NMR (126 MHz, DMSO-*d*_6_) δ 161.0 (Cq), 160.3 (Cq), 160.0 (Cq), 157.9 (CH), 143.6 (CH), 141.5 (Cq), 138.2 (Cq), 137.1 (Cq), 135.3 (Cq), 132.8 (Cq), 128.6 (CH), 128.5 (CH), 127.69 (CH), 127.67 (CH), 127.5 (CH), 120.6 (Cq), 54.1 (CH_2_), 53.7 (CH), 42.0 (CH_2_), 38.8 (CH_2_), 20.1 (CH_2_). ACQUITY UPLC BEH C_18_ 1.7 μm: Rt = 1.50 min; *m*/*z* 501.3 [M + H]^+^. HRMS calculated for C_26_H_25_N_6_O_3_S: 501.1709, found: 501.1708 [M + H]^+^. ELSD/UV/^1^H NMR purity: 100/88/96%.

#### 2,6-Dibenzyl-7-oxo-4,5,6,7-tetrahydro-2*H*-pyrazolo­[3,4-*c*]­pyridine-3-carboxylic Acid (**81**)

Synthesized according to General Procedure B using methyl 2,6-dibenzyl-7-oxo-4,5,6,7-tetrahydro-2*H*-pyrazolo­[3,4-*c*]­pyridine-3-carboxylate (0.80 g, 2.13 mmol, **55**) and LiOH·H_2_O (0.27 g, 6.39 mmol, 3 equiv). The reaction mixture was concentrated under reduced pressure, diluted with water (10 mL) and acidified with 1 M HCl. The aqueous layer was extracted with EtOAc (2 × 20 mL). The organic layers were combined, dried over NaSO_4_ and concentrated under reduced pressure. The crude mixture was purified by reverse-phase column chromatography (9:1 0.1% TFA in water/MeCN to 0:1 0.1% TFA in water/MeCN over 30 min) to afford 2,6-dibenzyl-7-oxo-4,5,6,7-tetrahydro-2*H*-pyrazolo­[3,4-*c*]­pyridine-3-carboxylic acid (**81**, 130 mg, 0.36 mmol, 17% yield) as an off-white solid. m.p.: 86–88 °C (dec). ^1^H NMR (500 MHz, DMSO-*d*_6_) δ 13.72 (br s, 1H), 7.37–7.16 (m, 10H), 5.78 (s, 2H), 4.66 (s, 2H), 2.97 (t, *J* = 6.7 Hz, 2H). CH_2_ not observed as overlapping with HDO peak. ^13^C­{^1^H} NMR (126 MHz, DMSO-*d*_6_) δ 160.5 (Cq), 159.7 (Cq), 141.8 (Cq), 137.6 (Cq), 137.1 (Cq), 129.0 (Cq), 128.6 (CH), 127.64 (CH), 127.60 (CH), 127.3 (CH), 127.2 (CH), 125.6 (Cq), 54.8 (CH_2_), 48.9 (CH_2_), 46.7 (CH_2_), 20.7 (CH_2_). ACQUITY UPLC BEH C_18_ 1.7 μm: Rt = 1.61 min; *m*/*z* 362.2 [M + H]^+^. HRMS calculated for C_21_H_20_N_3_O_3_, 362.1505, found 362.1520 [M + H]^+^. ELSD/UV/^1^H NMR purity: 100/91/95%.

#### 2,6-Dibenzyl-*N*-methyl-7-oxo-4,5,6,7-tetrahydro-2*H*-pyrazolo­[3,4-*c*]­pyridine-3-carboxamide (**82**)

To a solution of 2,6-dibenzyl-7-oxo-4,5,6,7-tetrahydro-2*H*-pyrazolo­[3,4-*c*]­pyridine-3-carboxylic acid (100 mg, 0.27 mmol) in THF (1 mL) were added DIPEA (0.12 mL, 0.69 mmol) and T3P (50% in EtOAc, 0.53 g, 0.83 mmol). The reaction mixture was stirred at room temperature for 10 min, followed by addition of CH_3_NH_2_ (2 M in THF, 0.2 mL, 0.41 mmol) and further stirred at room temperature overnight. The reaction mixture was diluted with water (25 mL) and extracted with EtOAc (2 × 25 mL). The organic layers were combined, dried over NaSO_4_, filtered and concentrated under reduced pressure. The crude mixture was purified by reverse-phase column chromatography (9:1 0.1% TFA in water/MeCN to 0:1 0.1% TFA in water/MeCN over 30 min). Fractions containing product were combined and concentrated under reduced pressure to afford 2,6-dibenzyl-*N*-methyl-7-oxo-4,5,6,7-tetrahydro-2*H*-pyrazolo­[3,4-*c*]­pyridine-3-carboxamide (**82**, 5 mg, 0.01 mmol, 5% yield) as an off-white solid. ^1^H NMR (500 MHz, DMSO-*d*_6_) δ 8.16 (q, *J* = 4.5 Hz, 1H), 7.37–7.21 (m, 10H), 5.61 (s, 2H), 4.65 (s, 2H), 3.48 (t, *J* = 6.7 Hz, 2H), 2.86 (t, *J* = 6.7 Hz, 2H), 2.74 (d, *J* = 4.6 Hz, 3H). ACQUITY UPLC BEH C_18_ 1.7 μm: Rt = 1.59 min; *m*/*z* 375.3 [M + H]^+^. ELSD/UV/^1^H NMR purity: 100/43/92%.

#### 2,6-Dibenzyl-*N*-cyclopropyl-7-oxo-4,5,6,7-tetrahydro-2H-pyrazolo­[3,4-*c*]­pyridine-3-carboxamide (**83**)

Synthesized according to General Procedure C using 2,6-dibenzyl-7-oxo-4,5,6,7-tetrahydro-2*H*-pyrazolo­[3,4-*c*]­pyridine-3-carboxylic acid (100 mg, 0.27 mmol), HOBt hydrate (10 mg, 0.30 mmol, 1.1 equiv), EDC·HCl (69 mg, 0.36 mmol, 1.3 equiv), DIPEA (0.12 mL, 0.69 mmol, 2.4 equiv) and cyclopropylamine (24 μL, 0.36 mmol, 1.3 equiv). Purified by reverse-phase column chromatography (9:1 0.1% TFA in water/MeCN to 0:1 0.1% TFA in water/MeCN over 30 min) to give 2,6-dibenzyl-*N*-cyclopropyl-7-oxo-4,5,6,7-tetrahydro-2*H*-pyrazolo­[3,4-*c*]­pyridine-3-carboxamide (**83**, 20 mg, 0.05 mmol, 18% yield) as an off-white solid. m.p.: 138–140 °C. ^1^H NMR (500 MHz, CDCl_3_) δ 8.31 (d, *J* = 4.1 Hz, 1H), 7.38–7.20 (m, 10H), 5.58 (s, 2H), 4.64 (s, 2H), 3.46 (t, *J* = 6.7 Hz, 2H), 2.83–2.72 (m, 3H), 0.73–0.65 (m, 2H), 0.51–0.41 (m, 2H). ^13^C­{^1^H} NMR (126 MHz, DMSO-*d*_6_) δ 160.6 (Cq), 160.0 (Cq), 141.3 (Cq), 137.8 (Cq), 136.9 (Cq), 133.1 (Cq), 128.5 (CH), 127.83 (CH), 127.80 (CH), 127.5 (CH), 127.2 (CH), 120.5 (Cq), 54.1 (CH_2_), 48.7 (CH_2_), 46.8 (CH_2_), 22.6 (CH), 19.8 (CH_2_), 5.8 (CH_2_). ACQUITY UPLC BEH C_18_ 1.7 μm: Rt = 1.66 min; *m*/*z* 401.3 [M + H]^+^. HRMS calculated for C_24_H_25_N_4_O_2_: 401.1978, found: 401.1980 [M + H]^+^. ELSD/UV/^1^H NMR purity: 100/100/100%.

#### 2,6-Dibenzyl-7-oxo-*N*-phenyl-4,5,6,7-tetrahydro-2*H*-pyrazolo­[3,4-*c*]­pyridine-3-carboxamide (**84**)

Synthesized according to General Procedure C using 2,6-dibenzyl-7-oxo-4,5,6,7-tetrahydro-2*H*-pyrazolo­[3,4-*c*]­pyridine-3-carboxylic acid (100 mg, 0.27 mmol), HOBt hydrate (40 mg, 0.30 mmol, 1.1 equiv), EDC·HCl (69 mg, 0.36 mmol, 1.3 equiv), DIPEA (0.12 mL, 0.69 mmol, 2.4 equiv) and aniline (37 μL, 0.41 mmol, 1.5 equiv). Purified by flash column chromatography (silica, 4 g, 1:0 DCM/MeOH to 9:1 DCM/MeOH over 25 CV’s) to give 2,6-dibenzyl-7-oxo-*N*-phenyl-4,5,6,7-tetrahydro-2*H*-pyrazolo­[3,4-*c*]­pyridine-3-carboxamide (**84**, 18 mg, 0.04 mmol, 15% yield) as an off-white solid. m.p.: 174–175 °C. ^1^H NMR (500 MHz, DMSO-*d*_6_) δ 10.32–10.10 (m, 1H), 7.71–7.53 (m, 2H), 7.44–7.02 (m, 13H), 5.68–5.53 (m, 2H), 4.75–4.56 (m, 2H), 3.63–3.40 (m, 2H), 3.00–2.81 (m, 2H). ^13^C­{^1^H} NMR (126 MHz, DMSO-*d*_6_) δ 159.9 (Cq), 157.9 (Cq), 141.5 (Cq), 138.2 (Cq), 137.8 (Cq), 136.8 (Cq), 133.3 (Cq), 128.9 (CH), 128.60 (CH), 128.55 (CH), 127.9 (CH), 127.8 (CH), 127.6 (CH), 127.2 (CH), 124.4 (CH), 121.2 (Cq), 120.1 (CH), 54.2 (CH_2_), 48.8 (CH_2_), 46.8 (CH_2_), 19.9 (CH_2_). ACQUITY UPLC BEH C_18_ 1.7 μm: Rt = 1.80 min; *m*/*z* 437.3 [M + H]^+^. HRMS calculated for C_27_H_25_N_4_O_2_: 437.1978, found: 437.1980 [M + H]^+^. ELSD/UV/^1^H NMR purity: 100/97/98%.

#### 2,6-Dibenzyl-*N*-(2-methoxyethyl)-7-oxo-4,5,6,7-tetrahydro-2*H*-pyrazolo­[3,4-*c*]­pyridine-3-carboxamide (**85**)

To a solution of 2,6-dibenzyl-7-oxo-4,5,6,7-tetrahydro-2*H*-pyrazolo­[3,4-*c*]­pyridine-3-carboxylic acid (100 mg, 0.27 mmol) in DCM (2 mL) were added DIPEA (0.12 mL, 0.69 mmol) and T3P (50% in EtOAc, 0.53 g, 0.83 mmol). The reaction mixture was stirred at room temperature for 10 min, followed by addition of 2-methoxyethylamine (36 μL, 0.41 mmol) and further stirred at room temperature overnight. The reaction mixture was diluted with water (2 mL) and extracted with DCM (2 × 5 mL). The organic layers were combined, dried over NaSO_4_, filtered and concentrated under reduced pressure. The crude mixture was purified by reverse-phase column chromatography (9:1 0.1% TFA in water/MeCN to 0:1 0.1% TFA in water/MeCN over 30 min). Fractions containing product were combined and concentrated under reduced pressure to afford 2,6-dibenzyl-*N*-(2-methoxyethyl)-7-oxo-4,5,6,7-tetrahydro-2*H*-pyrazolo­[3,4-*c*]­pyridine-3-carboxamide (**85**, 40 mg, 0.10 mmol, 35% yield) as an off-white solid. m.p.: 92–94 °C. ^1^H NMR (500 MHz, DMSO-*d*_6_) δ 8.33 (t, *J* = 5.1 Hz, 1H), 7.36–7.22 (m, 10H), 5.59 (s, 2H), 4.65 (s, 2H), 3.48 (t, *J* = 6.7 Hz, 2H), 3.39–3.37 (m, 2H), 3.23 (s, 3H), 2.84 (t, *J* = 6.7 Hz, 2H). CH_2_ not observed as overlapping with HDO peak. ^13^C­{^1^H} NMR (126 MHz, DMSO-*d*_6_) δ 160.0 (Cq), 159.5 (Cq), 141.4 (Cq), 137.8 (Cq), 137.0 (Cq), 133.2 (Cq), 128.54 (CH), 128.52 (CH), 127.9 (CH), 127.8 (CH), 127.6 (CH), 127.2 (CH), 120.5 (Cq), 70.2 (CH_2_), 57.9 (CH_3_), 54.1 (CH_2_), 48.8 (CH_2_), 46.8 (CH_2_), 38.7 (CH_2_), 19.8 (CH_2_). ACQUITY UPLC BEH C_18_ 1.7 μm: Rt = 1.63 min; *m*/*z* 419.3 [M + H]^+^. HRMS calculated for C_24_H_27_N_4_O_3_: 419.2085, found: 419.2083 [M + H]^+^. ELSD/UV/^1^H NMR purity: 98/92/94%.

#### 2,6-Dibenzyl-*N*-(2-(dimethylamino)­ethyl)-7-oxo-4,5,6,7-tetrahydro-2*H*-pyrazolo­[3,4-*c*]­pyridine-3-carboxamide (**86**)

To a solution of 2,6-dibenzyl-7-oxo-4,5,6,7-tetrahydro-2*H*-pyrazolo­[3,4-*c*]­pyridine-3-carboxylic acid (0.15 g, 0.41 mmol) in DCM (1 mL) were added DIPEA (0.18 mL, 1.03 mmol) and T3P (50% in EtOAc, 0.40 g, 1.24 mmol). The reaction mixture was stirred at room temperature for 10 min, followed by addition of *N*,*N*-dimethylethylenediamine (67 μL, 0.62 mmol) and further stirred at room temperature overnight. The reaction mixture was diluted with water (2 mL) and extracted with DCM (2 × 5 mL). The organic layers were combined, dried over NaSO_4_, filtered and concentrated under reduced pressure. The crude mixture was purified by reverse-phase column chromatography (9:1 5 mM (NH_4_)­HCO_3_ in water/MeCN to 0:1 5 mM (NH_4_)­HCO_3_ in water/MeCN over 30 min). Fractions containing product were combined and concentrated under reduced pressure to afford 2,6-dibenzyl-*N*-(2-(dimethylamino)­ethyl)-7-oxo-4,5,6,7-tetrahydro-2*H*-pyrazolo­[3,4-*c*]­pyridine-3-carboxamide (**86**, 15 mg, 0.03 mmol, 8% yield) as an off-white solid. ^1^H NMR (500 MHz, DMSO-*d*_6_) δ 8.17 (t, *J* = 5.8 Hz, 1H), 7.37–7.22 (m, 10H), 5.59 (s, 2H), 4.65 (s, 2H), 3.48 (t, *J* = 6.6 Hz, 2H), 2.85 (t, *J* = 6.7 Hz, 2H), 2.31 (t, *J* = 6.6 Hz, 2H), 2.13 (s, 6H). CH_2_ not observed as overlapping with HDO peak. ^13^C­{^1^H} NMR (126 MHz, DMSO-*d*_6_) δ 160.0 (Cq), 159.3 (Cq), 141.4 (Cq), 137.8 (Cq), 137.0 (Cq), 133.2 (Cq), 128.54 (CH), 128.51 (CH), 127.9 (CH), 127.8 (CH), 127.6 (CH), 127.2 (CH), 120.4 (Cq), 57.8 (CH_2_), 54.1 (CH_2_), 48.8 (CH_2_), 46.8 (CH_2_), 45.1 (CH_3_), 37.0 (CH_2_), 19.8 (CH_2_). ACQUITY UPLC BEH C_18_ 1.7 μm: Rt = 1.31 min; *m*/*z* 432.3 [M + H]^+^. HRMS calculated for C_25_H_30_N_5_O_2_: 432.2400, found: 432.2406 [M + H]^+^. ELSD/UV/^1^H NMR purity: 100/91/97%. ELSD/UV/^1^H NMR purity: 96/96/97%.

#### 2,6-Dibenzyl-7-oxo-4,5,6,7-tetrahydro-2*H*-pyrazolo­[3,4-*c*]­pyridine-3-carbohydrazide (**87**)

To a solution of methyl 2,6-dibenzyl-7-oxo-4,5,6,7-tetrahydro-2*H*-pyrazolo­[3,4-*c*]­pyridine-3-carboxylate (250 mg, 0.66 mmol) in EtOH (5 mL) was added NH_2_NH_2_ (50% in H_2_O, 1 mL). The reaction mixture was heated to 90 °C and stirred for 6 h. The reaction mixture was cooled to room temperature and concentrated under reduced pressure to afford 2,6-dibenzyl-7-oxo-4,5,6,7-tetrahydro-2*H*-pyrazolo­[3,4-*c*]­pyridine-3-carbohydrazide (**87**, 300 mg, 0.80 mmol) as a crude yellow gum, which was taken forward as crude to the next step.

#### 2,6-Dibenzyl-3-(1,3,4-oxadiazol-2-yl)-2,4,5,6-tetrahydro-7*H*-pyrazolo­[3,4-*c*]­pyridin-7-one (**89**)

2,6-dibenzyl-7-oxo-4,5,6,7-tetrahydro-2*H*-pyrazolo­[3,4-*c*]­pyridine-3-carbohydrazide (300 mg, 0.80 mmol) was added to triethyl orthoformate (5 mL) and the reaction mixture heated to 100 °C and stirred overnight. The reaction mixture was cooled to room temperature and concentrated under reduced pressure. The crude mixture was purified by reverse-phase column chromatography (9:1 5 mM (NH_4_)­HCO_3_ in water/MeCN to 0:1 5 mM (NH_4_)­HCO_3_ in water/MeCN over 30 min). Fractions containing product were combined and concentrated under reduced pressure to afford 2,6-dibenzyl-3-(1,3,4-oxadiazol-2-yl)-2,4,5,6-tetrahydro-7*H*-pyrazolo­[3,4-*c*]­pyridin-7-one (**89**, 42 mg, 0.11 mmol, 7% yield) as an off-white solid. m.p.: 150–152 °C. ^1^H NMR (500 MHz, CDCl_3_) δ 8.45 (s, 1H), 7.44–7.41 (m, 2H), 7.35–7.33 (m, 4H), 7.32–7.23 (m, 4H), 5.98 (s, 2H), 4.81 (s, 2H), 3.55 (t, *J* = 6.7 Hz, 2H), 3.05 (t, *J* = 6.7 Hz, 2H). ^13^C­{^1^H} NMR (126 MHz, CDCl_3_) δ 160.6 (Cq), 157.0 (Cq), 152.1 (CH), 143.4 (Cq), 137.2 (Cq), 135.6 (Cq), 128.9 (CH), 128.8 (CH), 128.34 (CH), 128.33 (CH), 127.8 (CH), 123.7 (Cq), 123.1 (Cq), 56.2 (CH_2_), 49.8 (CH_2_), 46.7 (CH_2_), 20.8 (CH_2_). ACQUITY UPLC BEH C_18_ 1.7 μm: Rt = 1.68 min; *m*/*z* 386.3 [M + H]^+^. HRMS calculated for C_22_H_20_N_5_O_2_: 386.1617, found: 386.1619 [M + H]^+^. ELSD/UV/^1^H NMR purity: 100/91/97%.

#### 2,6-Dibenzyl-*N*-(2-hydroxyethyl)-7-oxo-4,5,6,7-tetrahydro-2*H*-pyrazolo­[3,4-*c*]­pyridine-3-carboxamide (**88**)

To a solution of methyl 2,6-dibenzyl-7-oxo-4,5,6,7-tetrahydro-2*H*-pyrazolo­[3,4-*c*]­pyridine-3-carboxylate (200 mg, 0.52 mmol) and ethanolamine (0.16 mL, 2.66 mmol) in MeOH (10 mL) was added DIPEA (0.28 mL, 1.58 mmol). The reaction mixture was heated to 130 °C and stirred overnight in a sealed tube. The reaction mixture was cooled, concentrated under reduced pressure, diluted with water (5 mL) and extracted with EtOAc (2 × 10 mL). The organic layers were combined, dried over NaSO_4_, filtered and concentrated under reduced pressure. The crude mixture was purified by flash column chromatography (silica, 12 g, 1:0 heptane/EtOAc to 7:3 heptane/EtOAc over 25 CV’s). Fractions containing product were combined and concentrated under reduced pressure to afford 2,6-dibenzyl-*N*-(2-hydroxyethyl)-7-oxo-4,5,6,7-tetrahydro-2*H*-pyrazolo­[3,4-*c*]­pyridine-3-carboxamide (**88**, 160 mg, 0.40 mmol, 86% yield) as a pale yellow solid. m.p.: 140–142 °C. ^1^H NMR (500 MHz, DMSO-*d*_6_) δ 8.21 (t, *J* = 5.7 Hz, 1H), 7.39–7.22 (m, 10H), 5.59 (s, 2H), 4.73–4.70 (m, 1H), 4.65 (s, 2H), 3.51–3.42 (m, 4H), 3.28 (q, *J* = 6.0 Hz, 2H), 2.86 (t, *J* = 6.6 Hz, 2H). ^13^C­{^1^H} NMR (126 MHz, DMSO-*d*_6_) δ 160.0 (Cq), 159.5 (Cq), 141.4 (Cq), 137.8 (Cq), 137.0 (Cq), 133.2 (Cq), 128.54 (CH), 128.51 (CH), 127.9 (CH), 127.8 (CH), 127.6 (CH), 127.2 (CH), 120.4 (Cq), 59.4 (CH_2_), 54.1 (CH_2_), 48.8 (CH_2_), 46.8 (CH_2_), 41.8 (CH_2_), 19.9 (CH_2_). ACQUITY UPLC BEH C_18_ 1.7 μm: Rt = 1.50 min; *m*/*z* 405.3 [M + H]^+^. HRMS calculated for C_23_H_25_N_4_O_3_: 405.1927, found: 405.1929 [M + H]^+^. ELSD/UV/^1^H NMR purity: 100/88/99%.

#### 2,6-Dibenzyl-3-(4,5-dihydrooxazol-2-yl)-2,4,5,6-tetrahydro-7*H*-pyrazolo­[3,4-*c*]­pyridin-7-one (**90**)

To a stirred solution of 2,6-dibenzyl-*N*-(2-hydroxyethyl)-7-oxo-4,5,6,7-tetrahydro-2*H*-pyrazolo­[3,4-*c*]­pyridine-3-carboxamide (150 mg, 0.37 mmol) in toluene (2 mL) was added PPh_3_ (146 mg, 0.55 mmol) and DEAD (97 mg, 0.55 mmol). The reaction mixture was stirred at room temperature overnight. The reaction mixture was concentrated under reduced pressure. The crude mixture was diluted with ice-cold water (2 mL) and extracted with EtOAc (2 × 5 mL). The organic layers were combined, dried over NaSO_4_, filtered and concentrated under reduced pressure. The crude mixture was purified by reverse-phase column chromatography (9:1 5 mM (NH_4_)­HCO_3_ in water/MeCN to 0:1 5 mM (NH_4_)­HCO_3_ in water/MeCN over 30 min). Fractions containing product were combined and concentrated under reduced pressure to afford 2,6-dibenzyl-3-(4,5-dihydrooxazol-2-yl)-2,4,5,6-tetrahydro-7*H*-pyrazolo­[3,4-*c*]­pyridin-7-one (**90**, 7 mg, 0.02 mmol, 4%) as an off-white solid. ^1^H NMR (500 MHz, DMSO-*d*_6_) δ 7.37–7.23 (m, 8H), 7.22–7.18 (m, 2H), 5.87 (s, 2H), 4.65 (s, 2H), 4.36 (t, *J* = 9.6 Hz, 2H), 3.98 (t, *J* = 9.6 Hz, 2H), 3.50 (t, *J* = 6.7 Hz, 2H), 2.95 (t, *J* = 6.7 Hz, 2H). ACQUITY UPLC BEH C_18_ 1.7 μm: Rt = 1.77 min; *m*/*z* 387.3 [M + H]^+^. ELSD/UV/^1^H NMR purity: 100/100/96%.

#### 2,6-Dibenzyl-7-oxo-4,5,6,7-tetrahydro-2*H*-pyrazolo­[3,4-*c*]­pyridine-3-carbonitrile (**91**)

To a stirred solution of 2,6-dibenzyl-7-oxo-4,5,6,7-tetrahydro-2*H*-pyrazolo­[3,4-*c*]­pyridine-3-carboxamide (50 mg, 0.13 mmol) in DCM (3 mL) was added Et_3_N (1.35 mL, 0.97 mmol) and trifluoroacetic anhydride (96 μL, 0.69 mmol). The reaction mixture was stirred at room temperature overnight. The reaction mixture was concentrated under reduced pressure. The crude mixture was diluted with water (1 mL) and extracted with DCM (2 × 2 mL). The organic layers were combined, dried over NaSO_4_, filtered and concentrated under reduced pressure. The crude mixture was purified by reverse-phase column chromatography (9:1 5 mM (NH_4_)­HCO_3_ in water/MeCN to 0:1 5 mM (NH_4_)­HCO_3_ in water/MeCN over 30 min). Fractions containing product were combined and concentrated under reduced pressure to afford 2,6-dibenzyl-7-oxo-4,5,6,7-tetrahydro-2*H*-pyrazolo­[3,4-*c*]­pyridine-3-carbonitrile (**91**, 19 mg, 0.01 mmol, 40%) as an off-white solid. m.p.: 105–106 °C. ^1^H NMR (500 MHz, CDCl_3_) δ 7.44–7.41 (m, 2H), 7.39–7.27 (m, 8H), 5.52 (s, 2H), 4.76 (s, 2H), 3.51 (dd, *J* = 7.0, 6.4 Hz, 2H), 2.86 (t, *J* = 6.7 Hz, 2H). ^13^C­{^1^H} NMR (126 MHz, CDCl_3_) δ 159.8 (Cq), 143.2 (Cq), 136.9 (Cq), 134.2 (Cq), 129.2 (CH), 129.1 (CH), 128.9 (CH), 128.6 (CH), 128.3 (CH), 127.9 (CH), 111.8 (Cq), 109.8 (Cq), 56.8 (CH_2_), 49.8 (CH_2_), 46.4 (CH_2_), 19.8 (CH_2_). Cq not observed. ACQUITY UPLC BEH C_18_ 1.7 μm: Rt = 1.77 min; *m*/*z* 343.2 [M + H]^+^. HRMS calculated for C_21_H_19_N_4_O: 343.1559, found: 343.1564 [M + H]^+^. ELSD/UV/^1^H NMR purity: 100/92/100%.

### Molecular Docking

The predicted binding mode of MDI-117740 (**69**) was derived using a truncated version of chain A from protein structure 7ATU using Schrodinger software (Schrodinger, Inc., NY 10036). Although docking took a core constraint based on reference ligand LIJTF500025 (**7**) into consideration, no fixed constraints, features or voids were imposed. The system was then minimized to give the final pose.

### Cell Lines and Growth Conditions

HEK293 and SH-SY5Y cells used in this study were purchased from Sigma/Merck (Dorset, U.K.) and were cultured in Dulbecco’s Modified Eagle’s Medium (DMEM)/F12 (#11320033, Thermofisher Scientific, U.K.), supplemented with 10% fetal calf serum, 1% penicillin/streptomycin (Sigma-Aldrich, Dorset U.K.). MDA-MB-231 (human breast adenocarcinoma TNBC) cells were purchased from the American Type Culture Collection (ATCC, Virginia) and were cultured in DMEM containing sodium pyruvate (#11574446, Gibco, Fisher Scientific, U.K.). Cells were cultured in a standard T75 tissue-culture treated flask at 37 °C, 5% CO_2_ in a humidified sterile incubator. HEK293, SH-SY5Y and MDA-MB-231 cell lines used in this study were not obtained from animal or human participants and did not require ethical approval or informed consent.

### RapidFire Mass Spectrometry (RF-MS) Kinase Assays

A 50 μL reaction was prepared in 384-well polypropylene plates. First, compounds were transferred in duplicate in a 16-point 2-fold serial dilution in DMSO (maximum inhibitor concentration of 40 μM in the LIMK1/2 assay) using an ECHO 550 acoustic dispenser (Labcyte). To these plates, 25 μL of 80 nM LIMK1_330–637_ (for a final concentration of 40 nM) or 30 nM LIMK2_347–659_ (final concentration of 15 nM) in assay buffer was dispensed into each well using a COMBI multidrop dispenser and the plates were incubated for 45 min at room temperature. Then, 25 μL of 8 μM CFL1 (for a final concentration of 4 μM) and 4 mM ATP (final concentration 2 mM) in assay buffer was added to each well and incubated for 105 and 180 min for LIMK1 and LIMK2, respectively. The composition of the assay buffer used for LIMK1 was 50 mM tris pH 7.5, 0.1 mM EDTA, 0.1 mM EGTA, 1 mM MgCl_2_, while that of the assay buffer used for LIMK2 was 50 mM HEPES pH 7.5, 0.1 mM EGTA, 1 mM EDTA, and 1 mM MnCl_2_.

The LIMK phosphorylation reactions were halted by the addition of 5 μL of 10% formic acid (final concentration of 1%), and the assay plates were transferred onto a RapidFire RF360 instrument (Agilent). Once loaded, the samples were aspirated under vacuum and the salts and the nonvolatile buffer components were removed by loading onto a C4 solid-phase extraction (SPE) cartridge (Agilent Technologies) in 0.1% formic acid in water at a flow rate of 1.5 mL/min. Elution using 85% acetonitrile and 0.1% formic acid was then used to elute analytes into the mass spectrometer (Agilent 6530 QTOF) at a flow rate of 1.2 mL/min. The resulting data were analyzed using RapidFire integrator software (Agilent), and GraphPad Prism 7 was used to calculate IC_50_ values.

### Transient Transfection of HEK293 Cells

The transfection reagent mix of 1.25 mL Opti-MEM without phenol red (Fisher Life Technologies, U.K.) and 1.25 μg NanoLuc LIMK1 or LIMK2 kinase fusion vector, 11.25 μg transfection carrier DNA and 37.5 μL FuGENE HD transfection reagent (all Promega, Hampshire, U.K.) was prepared according to manufacturer’s protocol. HEK293 cells were resuspended in 5 mL growth media following trypsinization, neutralization and sedimentation. Cell density was calculated and adjusted to 1 × 10^5^ cells/mL for each transfection of either LIMK1 or LIMK2 in 25 mL growth media. The transfection mix was added directly to cells and mixed gently *via* inversion. Cells were then plated into T75 tissue culture flasks and incubated for 20 h at 37 °C, 5% CO_2_.

### Cellular NanoBRET LIMK1/2 Assay

The NanoBRET cellular target engagement assay was performed as previously described.^29^ Briefly, white 96-well plates containing LIMK1/2 transfected HEK293 cells and extracellular NanoLuc inhibitor was added either positive control (NanoBRET Tracer #10), negative control (DMSO) or test compound (8-point dose–response curve in DMSO in duplicate, final concentration of 0.5% for control wells). Following incubation of plates under standard conditions (37 °C, 5% CO_2_) for 2 h, plates were removed and allowed to reach RT for 15 min. Freshly prepared Nano-Glo substrate was then added to each well and luminescence measured using dual emission for the donor at 450 nm and the acceptor 610 nm on a BMG Pherastar plate reader. Kit components were purchased from Promega (Hampshire, U.K.).

### AlphaLISA SureFire Assay

The AlphaLISA assay for detection of p-cofilin Ser3 levels was followed as previously described.^29^ Briefly, 96-well plates containing SH-SY5Y cells was added either positive control (LIMKi3, 10 μM), negative control (0.5% DMSO) or test compound (8-point, 3-fold serial dilution in DMSO from 10 μM to 30 nM in duplicate). Cells were placed in the incubator for 2 h, after which the media was removed and the cells were lysed using 50 μL AlphaLISA 1× lysis buffer (PerkinElmer) containing protease inhibitor cocktail (Sigma/Merck, Dorset, U.K.) and Pierce phosphatase inhibitor cocktail (Thermofisher Scientific, U.K.). The cell lysate was then transferred to a clean, flat-bottom, white 384-well plate, to which 5 μL/well of acceptor bead solution consisting of Reaction buffer 1, Reaction buffer 2, Activation Buffer and Acceptor Beads from pCofilin SureFire Ultra assay kit (PerkinElmer, Cat# ALSU-PCOF-A500) was added under dim light. After plate shaking for 2 min at 450 rpm, centrifuged briefly and incubation at RT for 1 h, 5 μL/well of donor solution consisting of Dilution Buffer and Donor beads was added. The plate was read on a Pherastar reader (BMD Labtech Ltd., Aylesbury, U.K.) using an AlphaLISA cartridge and AlphaLISA plate settings. The AlphaLISA assay was robust and reproducible (*Z*′ = 0.7).

### Binding Affinity (*K*_d_) Assay

Apparent binding dissociation constants (*K*_d_) were determined using *K*_d_ELECT platform provided by Eurofins/DiscoverX (San Diego). Briefly, LIMK1/2 transfected HEK293 cells were tagged with DNA and incubated until lysis. Streptavidin-coated magnetic beads were treated with biotinylated small molecule ligands for 30 min at RT to generate affinity resins. Liganded beads were blocked with excess biotin and washed with blocking buffer (SeaBlock (Pierce), 1% BSA, 0.05% Tween 20, 1 mM DTT) to remove unbound ligand and reduce nonspecific binding. Test compound (11-point, 3-fold serial dilution from 100 μM to 1 nM in duplicate, final DMSO concentration of 0.9%) were transferred to 384-well plates containing beads and DNA-tagged LIMK1/2 by acoustic transfer (noncontact dispensing). The assay plates were incubated at RT with shaking for 1 h and the affinity beads washed with wash buffer (1× PBS, 0.05% Tween20). The beads were then resuspended in elution buffer (1× PBS, 0.05% Tween20, 0.5 μM nonbiotinylated affinity ligand) and incubated at RT with shaking for 30 min. The kinase concentration in the eluates was measured by qPCR. Binding constants *K*_d_ were calculated from the dose–response curve using the Hill equation

$$\mathrm{response}=\mathrm{background}-\frac{\mathrm{signal}-\mathrm{background}}{1+\left(\frac{{K_{d}}^{\mathrm{hillslope}}}{\mathrm{dos}e^{\mathrm{hillslope}}}\right)}$$

### Microsomal Stability

Five microliter microsomes (20 mg/mL, Corning BV) diluted into 95 μL PBS (pH 7.4 with 0.6% MeCN) containing 0.04% DMSO and 4 μM compound were incubated with 100 μL of prewarmed 4 mM NADPH in PBS (final concentrations: 0.5 mg/mL microsomes, 2 μM compound, 0.02% DMSO, 0.3% MeCN and 2 mM NADPH). After mixing thoroughly the *T* = 0 sample (40 μL) was immediately quenched into 80 μL ice cold MeOH containing 4 μM internal standard (Carbemazepine). Three further samples were quenched in the same way at *T* = 3, 9, and 30 min. Samples were incubated on ice for 30 min before centrifugation at 4700 rpm for 20 min. The supernatant was analyzed via LC-MS/MS and compound/Carbemazepine peak area ratios calculated to determine the rate of substrate depletion.

### Thermodynamic Solubility

1–2 mg of accurately weighed compound was suspended in 1 mL PBS (pH 7.0) and incubated (rotating end over end) at room temperature for 24 h. The samples were then centrifuged at >10,000 rpm for 10 min to pellet any remaining solid. The supernatant was then diluted sequentially (1:5, 1:50, 1:500, and 1:5000) in MeCN and mixed 1:1 with MeCN containing 4 μM of carbamazepine. To prepare the standard, an 8-point, 1:3 dilution curve was prepared in DMSO with a top concentration of 1 mM, which was then diluted to 1:100 in MeCN containing 2 μM carbamazepine. Standards and samples were analyzed *via* LCMS/MS. The compound carbamazepine peak area ratios were calculated, and the test article solubility was determined by interpolation from the standard curve.

### Caco-2-Permeability and Efflux

Bidirectional permeabilities and drug efflux were performed at Cyprotex (Alderley Park, U.K.). Caco-2 cells (derived from ATCC) were seeded onto Transwell plates at 1 × 10^5^ cells/cm^2^ and cultured in DMEM. The monolayers are prepared by rinsing both apical and basolateral surfaces twice with HBSS at pH 7.4 and incubating both compartments for 40 min to stabilize physiological parameters. For assessment of A → B permeability, HBSS was removed from the apical compartment and replaced with either positive control (antipyrine, atenolol, talinolol, estrone 3-sulfate) or test compound dosing solution (10 μM in assay buffer in duplicate, final DMSO concentration of ≤1%). For assessment of B → A permeability, HBSS was removed from the basolateral compartment and replaced with positive control or test compound dosing solution as above. After 2 h, apical compartment inserts and companion plates are separated and apical and basolateral samples diluted for analysis. Test and control compounds are quantified by LCMS/MS cassette analysis using a 7-point calibration with appropriate dilution of the samples. Permeability coefficient (*P*_app_) for each compound is calculated from the following equation

$$P_{\mathrm{app}}=\frac{dQ/dT}{C_{0}\times A}$$

where d*Q*/d*T* is the rate of permeation of compound across the cells, *C*_0_ is the donor compartment concentration at time zero, and *A* is the area of the cell monolayer. Only *P*_app_ A → B is reported for clarity. Efflux ratio (ER) is derived from *P*_app(B–A)_/*P*_app(A–B)_.

### *In Vivo* DMPK

*In vivo* studies were carried out under appropriate licenses at Sai Life (India) or internally using male Sprague–Dawley rats. MDI-117740 (**69**) was either dosed: (i) i.v. in a formulation of 10% DMSO, 20% Cremophor EL, 70% saline (v/v/v) at a concentration of 0.04 mg/mL with dose volume of 5 mL/kg for final dose of 0.2 mg/kg, (ii) p.o. in a formulation of 20% hydroxypropyl-β-cyclodextrin in dH_2_O (w/v) at a concentration of 0.3 mg/mL with dose volume of 10 mL/kg for final dose of 3 mg/kg, or, (iii) i.p. in a formulation of 20% hydroxypropyl-β-cyclodextrin in dH_2_O (w/v) at a concentration of 2 mg/mL with dose volume 5 mL/kg for final dose of 10 mg/kg. Serial plasma samples were obtained at predetermined time points and then stored at −20 °C. Brain samples from a CNS satellite group were isolated at 1 h, homogenized and stored at −20 °C. Samples were then thawed, protein precipitated with MeCN followed by LC-MS/MS quantification. No adverse effects were noted for the duration of the *in vivo* experiments.

### Wound Healing Assay

The wound healing or scratch assay used for monitoring adherent cell migration over time following the induction of a “scratched” area in the monolayer with a 200 μL pipet tip. The MDA-MB-231 breast cancer cell line was seeded in a clear, 12-well plate, approximately 24 h prior to the experiment to form a complete monolayer. Following wound induction, the disrupted cells were gently removed from the wells and fresh medium supplemented with or without compound was added. The cells were incubated for 48 h and images were taken using the ZOE cell imager (BIO-RAD) to monitor the wounded gap. The initial width of the wound was measured, followed by measurements after 48 h. The images were measured using ImageJ software and the wound closure rate, indicative of cellular motility and migration, was calculated using GraphPad Prism 10.5.0.

### Statistical Analysis

Statistical analysis was performed using GraphPad Prism (v10.5.0). For wound healing assays, percentage wound closure at 48 h was compared across treatment groups using an ordinary one-way ANOVA followed by Dunnett’s post hoc test to determine significance (*p* < 0.05) of differences between control sample and test samples. Data are presented as mean ± SEM from *n* = 4 independent experiments. Statistical significance was set at *p* < 0.05. Details about the multiple comparisons test can be found in Table S2.

### X-ray Crystallography

X-ray diffraction structures of MDI-117740 (**69**) and intermediates **39** and **49** are deposited with Cambridge Crystallographic Data Centre (CCDC) under deposition numbers 2467213, 2440761 and 2440762, respectively. Thermal ellipsoids for compound **69** and intermediates **39** and **49** can be found in Figures S1–S3.

## Supplementary Material





## Notes

DiscoverX scanMAX panel for compound **69**; thermal ellipsoids for compound **69** and intermediates **39** and **49**; statistical data used to compare effect of LIMK inhibitors on cell migration in the wound healing assay; ^1^H, ^13^C NMR, and UPLC-MS characterization data and details of chemical synthesis of all final compounds (PDF)
Formula Strings containing SMILES string and associated biochemical and biological data (CSV)

## ∥

Tay Therapeutics Limited, Dundee University Incubator, 3 James Lindsay Place, Dundee DD1 5JJ, United Kingdom

## ⊥

Division of Structural Biology, The Institute of Cancer Research (ICR), London SW3 6JB, United Kingdom

## #

Recursion plc, 155 Brook Drive, Milton Park, Abingdon OX14 4SD, United Kingdom

## ∇

Institute of Systems, Molecular and Integrative Biology, University of Liverpool, Biosciences Building, Crown Street, Liverpool L69 7BE, United Kingdom

## ○

European Cancer Stem Cell Research Institute, School of Biosciences, Cardiff University, Haydn Ellis Building, Maindy Road, Cardiff CF24 4HQ, United Kingdom

## Abbreviations Used

ADF: actin-depolymerizing factor
ASD: autism spectrum disorder
AUC_inf_: area under the curve (to infinity)
B/P: brain-to-plasma ratio
BMPR2: bone morphogenetic protein receptor type 2
CaMKIV: calcium/calmodulin-dependent protein kinase IV
CL_int_: intrinsic clearance
*C*_max_: maximum plasma concentration
DMPK: drug metabolism and pharmacokinetics
ER: efflux ratio
*F*%: % oral bioavailability
FMRP: fragile X mental retardation protein
FXS: fragile X syndrome
i.p.: intraperitoneal
i.v.: intravenous
LIMK: LIM domain kinase
MRCKα: myotonic dystrophy kinase-related Cdc42-binding kinase α
PAK: p21-activated kinase
*P*_app_: apparent permeability
p.o.: oral administration
RIPK1: receptor-interacting protein 1 kinase
ROCK: Rho-associated protein kinase
*T*_1/2_: half-life
TESK: testis specific protein kinase
TKL: tyrosine kinase-like kinase
*T*_max_: time taken to reach maximum plasma concentration
*V*_D_: volume of distribution
