# Supplementary material for: Tetrahydropyrazolopyridinones as a Novel Class of Potent and Highly Selective LIMK Inhibitors
Source: J Med Chem. 2025 Aug 6;68(16):17427–56. doi: 10.1021/acs.jmedchem.5c00974 (PMC12406257; doi:10.1021/acs.jmedchem.5c00974)
Supplement: Supplementary file 1 [file jm5c00974_si_001.pdf]

Supporting Information

Tetrahydropyrazolopyridinones as a novel class of potent and highly selective LIMK inhibitors

Alex G. Baldwin[1], David W. Foley[1†], D. Heulyn Jones[1], Hyunah Lee[2§], Ross Collins[1‡], Ben Wahab[1], Josephine Pedder[1‖], Loren Waters[1], Marie Paine[1], Lauramariú Schino[1], Gui Jie Feng[1⊥], Benson Kariuki[3], Jonathan M. Elkins[2], John R. Atack[1], Simon E. Ward[1*]

[1]Medicines Discovery Institute, School of Biosciences, Cardiff University, Main Building, Park Place, Cardiff, CF10 3AT, United Kingdom. [2]Centre for Medicines Discovery, University of Oxford, Roosevelt Drive, Oxford, OX3 7DQ, United Kingdom. [3]School of Chemistry, Cardiff University, Main Building, Park Place, Cardiff, CF10 3AT, United Kingdom.

Corresponding Author email address: WardS10@cardiff.ac.uk

Contents

Supplementary Schemes and Tables 4

Scheme S1 | Attempted synthesis of **12**. 4

Table S1 | KINOMEscan™ of MDI-117740 (**69**) tested against 468 kinases (Eurofins/DiscoverX scanMAX panel) at 300 nM. 6

Figure S1 | Thermal ellipsoids for intermediate **39**. 17

Figure S2 | Thermal ellipsoids for intermediate **49**. 18

Figure S3 | Thermal ellipsoids for MDI-117740 (**69**). 19

Table S2 | Statistical data used to compare effect of LIMK inhibitors on cell migration in a wound healing assay in MDA-MB-231 cells 20

^{1}H, ^{13}C, COSY, HSQC, HMBC NMR and UPLC data for final compounds 21

Figure S4 | Stacked ^{1}H NMR spectra of **34** (blue line) and regioisomer **57** (red line) in $CDCl_3$ 21

(*S*)-3-(2-Benzyl-3-chloro-7-oxo-2,4,5,7-tetrahydro-6*H*-pyrazolo[3,4-*c*]pyridin-6-yl)-5-methyl-4-oxo-2,3,4,5-tetrahydrobenzo[*b*][1,4]oxazepine-8-carbonitrile (**8**, compound 22) 22

(*S*)-3-Benzyl-*N*-(5-methyl-4-oxo-2,3,4,5-tetrahydrobenzo[*b*][1,4]oxazepin-3-yl)-1*H*-1,2,4-triazole-5-carboxamide (**9**, GSK2982772) 24

(*S*)-5-Benzyl-*N*-(5-methyl-4-oxo-2,3,4,5-tetrahydrobenzo[*b*][1,4]oxazepin-3-yl)isoxazole-3-carboxamide (**10**, GSK'481) 26

(*S*)-3-Benzyl-*N*-(7,9-difluoro-2-oxo-2,3,4,5-tetrahydro-1H-benzo[*b*]azepin-3-yl)-1*H*-1,2,4-triazole-5-carboxamide (**11**, GSK3145095) 28

(*S*)-3-(2-Benzyl-3-bromo-7-oxo-2,4,5,7-tetrahydro-6*H*-pyrazolo[3,4-*c*]pyridin-6-yl)-8-chloro-5-methyl-2,3-dihydrobenzo[*b*][1,4]oxazepin-4(5*H*)-one (**19**) 29

(*S*)-1-Benzyl-6-(8-chloro-5-methyl-4-oxo-2,3,4,5-tetrahydrobenzo[*b*][1,4]oxazepin-3-yl)-7-oxo-4,5,6,7-tetrahydro-1*H*-pyrazolo[3,4-*c*]pyridine-3-carboxamide (**20**) 30

(*S*)-2-Benzyl-6-(5-methyl-4-oxo-2,3,4,5-tetrahydrobenzo[*b*][1,4]oxazepin-3-yl)-7-oxo-4,5,6,7-tetrahydro-2*H*-pyrazolo[3,4-*c*]pyridine-3-carboxamide (**21**) 31

(*S*)-1-Benzyl-6-(5-methyl-4-oxo-2,3,4,5-tetrahydrobenzo[*b*][1,4]oxazepin-3-yl)-7-oxo-4,5,6,7-tetrahydro-1*H*-pyrazolo[3,4-*c*]pyridine-3-carboxamide (**22**) 35

(*S*)-2-Benzyl-*N*-methyl-6-(5-methyl-4-oxo-2,3,4,5-tetrahydrobenzo[*b*][1,4]oxazepin-3-yl)-7-oxo-4,5,6,7-tetrahydro-2*H*-pyrazolo[3,4-*c*]pyridine-3-carboxamide (**23**) 39

(*S*)-1-Benzyl-*N*-methyl-6-(5-methyl-4-oxo-2,3,4,5-tetrahydrobenzo[*b*][1,4]oxazepin-3-yl)-7-oxo-4,5,6,7-tetrahydro-1*H*-pyrazolo[3,4-*c*]pyridine-3-carboxamide (**24**) 40

(*S*)-2-Benzyl-*N*,*N*-dimethyl-6-(5-methyl-4-oxo-2,3,4,5-tetrahydrobenzo[*b*][1,4]oxazepin-3-yl)-7-oxo-4,5,6,7-tetrahydro-2*H*-pyrazolo[3,4-*c*]pyridine-3-carboxamide (**25**) 41

(*S*)-1-Benzyl-*N*,*N*-dimethyl-6-(5-methyl-4-oxo-2,3,4,5-tetrahydrobenzo[*b*][1,4]oxazepin-3-yl)-7-oxo-4,5,6,7-tetrahydro-1*H*-pyrazolo[3,4-*c*]pyridine-3-carboxamide (**26**) 42

(*S*)-1-Benzyl-N^3-(5-methyl-4-oxo-2,3,4,5-tetrahydrobenzo[*b*][1,4]oxazepin-3-yl)-1*H*-pyrazole-3,5-dicarboxamide (**29**) 43

1-Benzyl-3-(2-(benzyl(cyclopropylmethyl)amino)-2-oxoethyl)-1H-pyrazole-5-carboxamide (**31**) .. 46

N^3,1-Dibenzyl-N^3-(cyclopropylmethyl)-1*H*-pyrazole-3,5-dicarboxamide (**32**).................... 49

(*S*)-*N*-(8-Chloro-5-methyl-4-oxo-2,3,4,5-tetrahydrobenzo[*b*][1,4]oxazepin-3-yl)-4-(*N*-phenylsulfamoyl)benzamide (**33**) 50

2,6-Dibenzyl-7-oxo-4,5,6,7-tetrahydro-2*H*-pyrazolo[3,4-*c*]pyridine-3-carboxamide (**34**) 53

Methyl 2,6-dibenzyl-7-oxo-4,5,6,7-tetrahydro-2*H*-pyrazolo[3,4-*c*]pyridine-3-carboxylate (**55**) 56

1,6-Dibenzyl-7-oxo-4,5,6,7-tetrahydro-1*H*-pyrazolo[3,4-*c*]pyridine-3-carboxamide (**57**) 59

2-Benzyl-7-oxo-6-phenethyl-4,5,6,7-tetrahydro-2*H*-pyrazolo[3,4-*c*]pyridine-3-carboxamide (**58**) 62

2-Benzyl-7-oxo-6-(2-phenoxyethyl)-4,5,6,7-tetrahydro-2*H*-pyrazolo[3,4-*c*]pyridine-3-carboxamide (**59**) 66

2-Benzyl-6-(4-hydroxybenzyl)-7-oxo-4,5,6,7-tetrahydro-2*H*-pyrazolo[3,4-*c*]pyridine-3-carboxamide (**60**) 69

2-Benzyl-6-(4-methoxybenzyl)-7-oxo-4,5,6,7-tetrahydro-2*H*-pyrazolo[3,4-*c*]pyridine-3-carboxamide (**61**) 72

2-Benzyl-6-(3-hydroxyphenethyl)-7-oxo-4,5,6,7-tetrahydro-2*H*-pyrazolo[3,4-*c*]pyridine-3-carboxamide (**62**) 75

2-Benzyl-6-(3-methoxyphenethyl)-7-oxo-4,5,6,7-tetrahydro-2*H*-pyrazolo[3,4-*c*]pyridine-3-carboxamide (**63**) 79

2-Benzyl-6-(2-methoxyethyl)-7-oxo-4,5,6,7-tetrahydro-2*H*-pyrazolo[3,4-*c*]pyridine-3-carboxamide (**64**) 82

2-Benzyl-6-(2-(dimethylamino)-2-oxoethyl)-7-oxo-4,5,6,7-tetrahydro-2*H*-pyrazolo[3,4-*c*]pyridine-3-carboxamide (**65**) 85

2-Benzyl-6-(3-hydroxy-1-phenylpropyl)-7-oxo-4,5,6,7-tetrahydro-2*H*-pyrazolo[3,4-*c*]pyridine-3-carboxamide (**66**) 88

2-Benzyl-7-oxo-6-phenethyl-4,5,6,7-tetrahydro-2*H*-pyrazolo[3,4-*c*]pyridine-3-carboxylic acid (**67**) 92

2-Benzyl-6-(cyclopropyl(phenyl)methyl)-7-oxo-4,5,6,7-tetrahydro-2*H*-pyrazolo[3,4-*c*]pyridine-3-carboxamide (**68**) 95

(*R*)-2-Benzyl-6-(cyclopropyl(phenyl)methyl)-7-oxo-4,5,6,7-tetrahydro-2*H*-pyrazolo[3,4-*c*]pyridine-3-carboxamide (**69**, MDI-117740) 97

(*S*)-2-Benzyl-6-(cyclopropyl(phenyl)methyl)-7-oxo-4,5,6,7-tetrahydro-2*H*-pyrazolo[3,4-*c*]pyridine-3-carboxamide (**70**) 101

2-Benzyl-6-(cyclopropyl(2-((2-methoxyethyl)amino)phenyl)methyl)-7-oxo-4,5,6,7-tetrahydro-2*H*-pyrazolo[3,4-*c*]pyridine-3-carboxamide (**71**) 104

2,6-Dibenzyl-7-oxo-4,5,6,7-tetrahydro-2*H*-pyrazolo[3,4-*c*]pyridine-3-carboxylic acid (**81**) 110

2,6-Dibenzyl-*N*-methyl-7-oxo-4,5,6,7-tetrahydro-2*H*-pyrazolo[3,4-*c*]pyridine-3-carboxamide (**82**) 113

2,6-Dibenzyl-*N*-cyclopropyl-7-oxo-4,5,6,7-tetrahydro-2*H*-pyrazolo[3,4-*c*]pyridine-3-carboxamide (**83**) 114

2,6-Dibenzyl-7-oxo-*N*-phenyl-4,5,6,7-tetrahydro-2*H*-pyrazolo[3,4-*c*]pyridine-3-carboxamide (**84**) 117

2,6-Dibenzyl-*N*-(2-methoxyethyl)-7-oxo-4,5,6,7-tetrahydro-2*H*-pyrazolo[3,4-*c*]pyridine-3-carboxamide (**85**) 120

2,6-Dibenzyl-*N*-(2-(dimethylamino)ethyl)-7-oxo-4,5,6,7-tetrahydro-2*H*-pyrazolo[3,4-*c*]pyridine-3-carboxamide (**86**) 123

2,6-Dibenzyl-*N*-(2-hydroxyethyl)-7-oxo-4,5,6,7-tetrahydro-2*H*-pyrazolo[3,4-*c*]pyridine-3-carboxamide (**88**) 126

2,6-Dibenzyl-3-(1,3,4-oxadiazol-2-yl)-2,4,5,6-tetrahydro-7*H*-pyrazolo[3,4-c]pyridin-7-one (**89**) . 129

2,6-Dibenzyl-3-(4,5-dihydrooxazol-2-yl)-2,4,5,6-tetrahydro-7*H*-pyrazolo[3,4-*c*]pyridin-7-one (**90**) 132

2,6-Dibenzyl-7-oxo-4,5,6,7-tetrahydro-2*H*-pyrazolo[3,4-*c*]pyridine-3-carbonitrile (**91**) 133

Supplementary Schemes and Tables

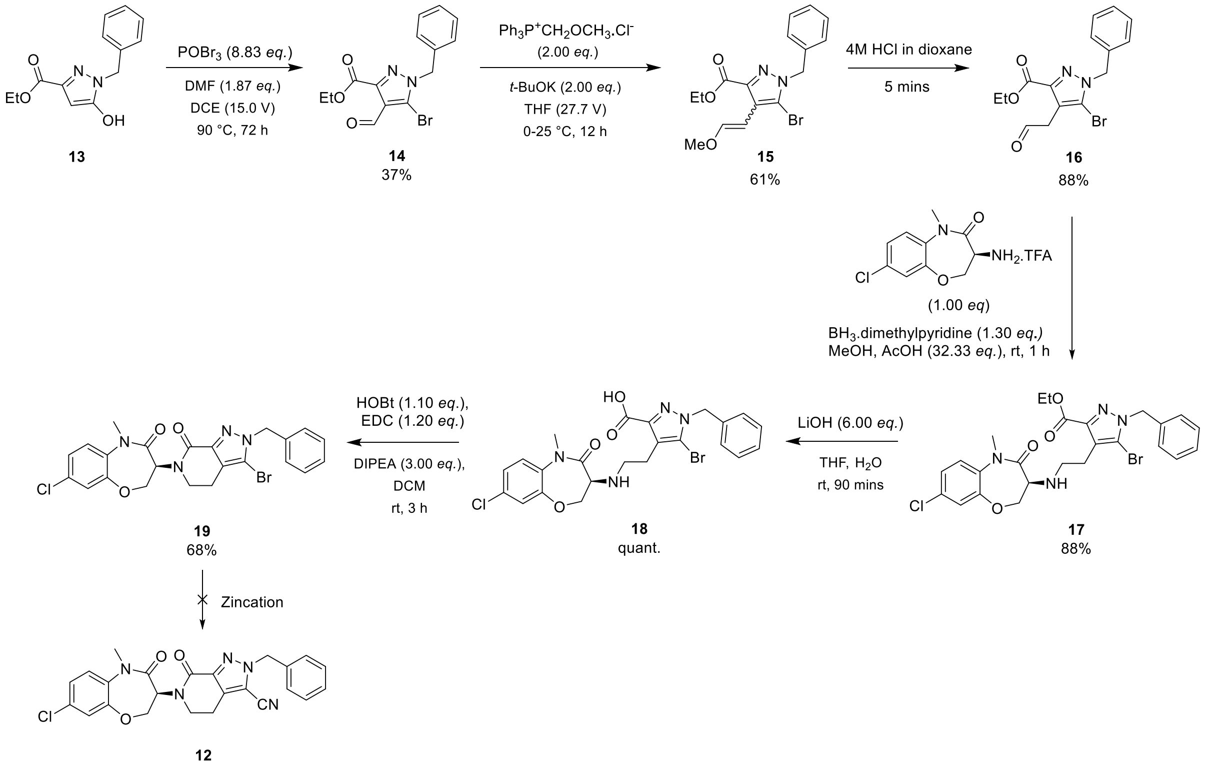

Scheme S1 | Attempted synthesis of **12**.

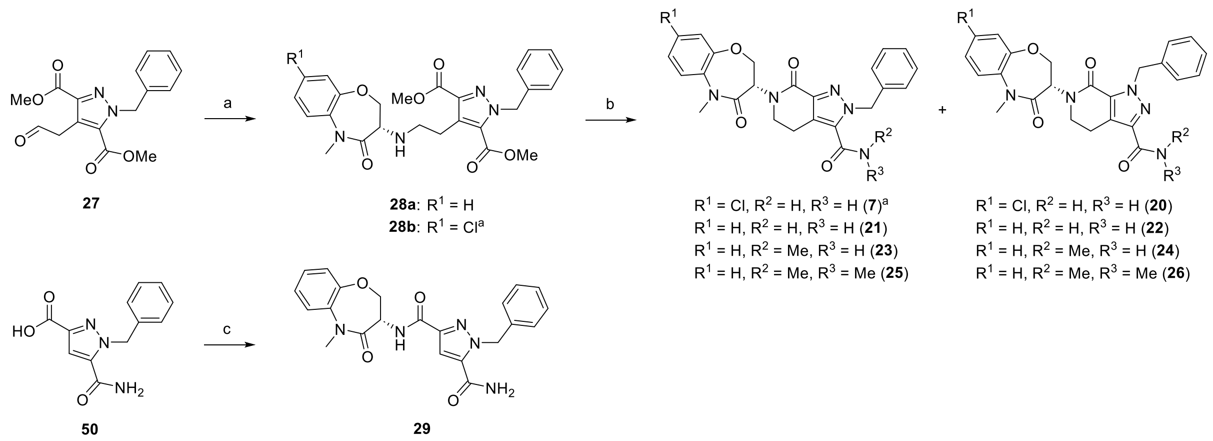

Scheme S2 | Synthesis of LIJTF500025 (**7**) and analogues **20**-**26** and **29**. (a) (3*S*)-3-amino-5-methyl-2,3,4,5-tetrahydro-1,5-benzoxazepin-4-one hydrochloride (1.1-1.5 equiv.), 2-methylpyridine·BH_3 complex (0.9-1.5 equiv.), MeOH, 0 °C, 3 h, 52%; (b) i) LiOH·H_2O (3.0-5.0 equiv.), 1:1:1 MeOH:THF:H_2O, rt, 18 h, ii) HOBt (1.1 equiv.), EDC·HCl (1.2-1.3 equiv.), DIPEA (1.5-2.4 equiv.), DCM, rt, 18 h, iii) R^2R^3NH·HCl (1.5-6.9 equiv.), HOBt (1.1 equiv.), EDC·HCl (1.3 equiv.), DIPEA (2.4 equiv.), DCM, rt, 18 h, 3-28%; (c) (*S*)-3-amino-5-methyl-2,3-dihydrobenzo[*b*][1,4]oxazepin-4(5*H*)-one hydrochloride (1.1 equiv.), HOBt (1.1 equiv.), EDC·HCl (1.2 equiv.), DIPEA (1.5 equiv.), DCM, rt, 18 h, 49%. [a]Synthesis previously reported in Collins *et al*, *J. Med. Chem.* **2022**, *65* (20), 13705-13713.

Table S1 | KINOMEscan™ of MDI-117740 (**69**) tested against 468 kinases (Eurofins/DiscoverX scanMAX panel) at 300 nM. Kinases with binding interaction < 50% relative to control (% Ctrl) are highlighted.

DiscoveRx Gene Symbol	Entrez Gene Symbol	% Ctrl
AAK1	AAK1	100
ABL1(E255K)-phosphorylated	ABL1	72
ABL1(F317I)-nonphosphorylated	ABL1	95
ABL1(F317I)-phosphorylated	ABL1	97
ABL1(F317L)-nonphosphorylated	ABL1	100
ABL1(F317L)-phosphorylated	ABL1	100
ABL1(H396P)-nonphosphorylated	ABL1	87
ABL1(H396P)-phosphorylated	ABL1	70
ABL1(M351T)-phosphorylated	ABL1	97
ABL1(Q252H)-nonphosphorylated	ABL1	78
ABL1(Q252H)-phosphorylated	ABL1	83
ABL1(T315I)-nonphosphorylated	ABL1	100
ABL1(T315I)-phosphorylated	ABL1	83
ABL1(Y253F)-phosphorylated	ABL1	81
ABL1-nonphosphorylated	ABL1	81
ABL1-phosphorylated	ABL1	74
ABL2	ABL2	89
ACVR1	ACVR1	95
ACVR1B	ACVR1B	88
ACVR2A	ACVR2A	100
ACVR2B	ACVR2B	100
ACVRL1	ACVRL1	99
ADCK3	CABC1	91
ADCK4	ADCK4	100
AKT1	AKT1	100
AKT2	AKT2	88
AKT3	AKT3	88
ALK	ALK	100
ALK(C1156Y)	ALK	100
ALK(L1196M)	ALK	100
AMPK-alpha1	PRKAA1	100
AMPK-alpha2	PRKAA2	85
ANKK1	ANKK1	81
ARK5	NUAK1	100
ASK1	MAP3K5	90
ASK2	MAP3K6	81
AURKA	AURKA	100
AURKB	AURKB	98
AURKC	AURKC	87

AXL	AXL	99
BIKE	BMP2K	88
BLK	BLK	87
BMPR1A	BMPR1A	89
BMPR1B	BMPR1B	83
BMPR2	BMPR2	100
BMX	BMX	89
BRAF	BRAF	79
BRAF(V600E)	BRAF	85
BRK	PTK6	100
BRSK1	BRSK1	100
BRSK2	BRSK2	97
BTK	BTK	91
BUB1	BUB1	100
CAMK1	CAMK1	94
CAMK1B	PNCK	100
CAMK1D	CAMK1D	100
CAMK1G	CAMK1G	100
CAMK2A	CAMK2A	100
CAMK2B	CAMK2B	100
CAMK2D	CAMK2D	97
CAMK2G	CAMK2G	100
CAMK4	CAMK4	100
CAMKK1	CAMKK1	86
CAMKK2	CAMKK2	98
CASK	CASK	100
CDC2L1	CDK11B	98
CDC2L2	CDC2L2	95
CDC2L5	CDK13	93
CDK11	CDK19	100
CDK2	CDK2	100
CDK3	CDK3	100
CDK4	CDK4	96
CDK4-cyclinD1	CDK4	98
CDK4-cyclinD3	CDK4	71
CDK5	CDK5	97
CDK7	CDK7	91
CDK8	CDK8	100
CDK9	CDK9	99
CDKL1	CDKL1	82
CDKL2	CDKL2	100
CDKL3	CDKL3	96
CDKL5	CDKL5	87
CHEK1	CHEK1	100

CHEK2	CHEK2	87
CIT	CIT	96
CLK1	CLK1	91
CLK2	CLK2	100
CLK3	CLK3	97
CLK4	CLK4	91
CSF1R	CSF1R	99
CSF1R-autoinhibited	CSF1R	96
CSK	CSK	100
CSNK1A1	CSNK1A1	100
CSNK1A1L	CSNK1A1L	100
CSNK1D	CSNK1D	100
CSNK1E	CSNK1E	100
CSNK1G1	CSNK1G1	100
CSNK1G2	CSNK1G2	92
CSNK1G3	CSNK1G3	96
CSNK2A1	CSNK2A1	67
CSNK2A2	CSNK2A2	95
CTK	MATK	89
DAPK1	DAPK1	100
DAPK2	DAPK2	100
DAPK3	DAPK3	100
DCAMKL1	DCLK1	95
DCAMKL2	DCLK2	96
DCAMKL3	DCLK3	100
DDR1	DDR1	98
DDR2	DDR2	100
DLK	MAP3K12	100
DMPK	DMPK	90
DMPK2	CDC42BPG	79
DRAK1	STK17A	96
DRAK2	STK17B	98
DYRK1A	DYRK1A	71
DYRK1B	DYRK1B	97
DYRK2	DYRK2	78
EGFR	EGFR	88
EGFR(E746-A750del)	EGFR	64
EGFR(G719C)	EGFR	100
EGFR(G719S)	EGFR	94
EGFR(L747-E749del, A750P)	EGFR	83
EGFR(L747-S752del, P753S)	EGFR	100
EGFR(L747-T751del,Sins)	EGFR	98
EGFR(L858R)	EGFR	80
EGFR(L858R,T790M)	EGFR	63

EGFR(L861Q)	EGFR	100
EGFR(S752-I759del)	EGFR	89
EGFR(T790M)	EGFR	87
EIF2AK1	EIF2AK1	86
EPHA1	EPHA1	83
EPHA2	EPHA2	93
EPHA3	EPHA3	97
EPHA4	EPHA4	90
EPHA5	EPHA5	97
EPHA6	EPHA6	95
EPHA7	EPHA7	89
EPHA8	EPHA8	93
EPHB1	EPHB1	99
EPHB2	EPHB2	100
EPHB3	EPHB3	90
EPHB4	EPHB4	97
EPHB6	EPHB6	100
ERBB2	ERBB2	100
ERBB3	ERBB3	100
ERBB4	ERBB4	100
ERK1	MAPK3	100
ERK2	MAPK1	97
ERK3	MAPK6	90
ERK4	MAPK4	98
ERK5	MAPK7	100
ERK8	MAPK15	90
ERN1	ERN1	87
FAK	PTK2	100
FER	FER	96
FES	FES	94
FGFR1	FGFR1	100
FGFR2	FGFR2	88
FGFR3	FGFR3	76
FGFR3(G697C)	FGFR3	83
FGFR4	FGFR4	99
FGR	FGR	93
FLT1	FLT1	92
FLT3	FLT3	90
FLT3(D835H)	FLT3	86
FLT3(D835V)	FLT3	100
FLT3(D835Y)	FLT3	84
FLT3(ITD)	FLT3	100
FLT3(ITD,D835V)	FLT3	81
FLT3(ITD,F691L)	FLT3	100

FLT3(K663Q)	FLT3	96
FLT3(N841I)	FLT3	90
FLT3(R834Q)	FLT3	100
FLT3-autoinhibited	FLT3	88
FLT4	FLT4	86
FRK	FRK	99
FYN	FYN	100
GAK	GAK	100
GCN2(Kin.Dom.2,S808G)	EIF2AK4	100
GRK1	GRK1	100
GRK2	ADRBK1	94
GRK3	ADRBK2	67
GRK4	GRK4	100
GRK7	GRK7	98
GSK3A	GSK3A	100
GSK3B	GSK3B	93
HASPIN	GSG2	87
HCK	HCK	98
HIPK1	HIPK1	96
HIPK2	HIPK2	64
HIPK3	HIPK3	100
HIPK4	HIPK4	100
HPK1	MAP4K1	83
HUNK	HUNK	97
ICK	ICK	81
IGF1R	IGF1R	88
IKK-alpha	CHUK	100
IKK-beta	IKBKB	100
IKK-epsilon	IKBKE	95
INSR	INSR	88
INSRR	INSRR	100
IRAK1	IRAK1	75
IRAK3	IRAK3	100
IRAK4	IRAK4	83
ITK	ITK	92
JAK1(JH1domain-catalytic)	JAK1	98
JAK1(JH2domain-pseudokinase)	JAK1	100
JAK2(JH1domain-catalytic)	JAK2	100
JAK3(JH1domain-catalytic)	JAK3	96
JNK1	MAPK8	88
JNK2	MAPK9	79
JNK3	MAPK10	84
KIT	KIT	96
KIT(A829P)	KIT	96

KIT(D816H)	KIT	82
KIT(D816V)	KIT	86
KIT(L576P)	KIT	90
KIT(V559D)	KIT	90
KIT(V559D,T670I)	KIT	100
KIT(V559D,V654A)	KIT	100
KIT-autoinhibited	KIT	84
LATS1	LATS1	86
LATS2	LATS2	99
LCK	LCK	96
LIMK1	LIMK1	17
LIMK2	LIMK2	12
LKB1	STK11	100
LOK	STK10	99
LRRK2	LRRK2	85
LRRK2(G2019S)	LRRK2	100
LTK	LTK	93
LYN	LYN	100
LZK	MAP3K13	100
MAK	MAK	92
MAP3K1	MAP3K1	100
MAP3K15	MAP3K15	58
MAP3K2	MAP3K2	83
MAP3K3	MAP3K3	100
MAP3K4	MAP3K4	80
MAP4K2	MAP4K2	74
MAP4K3	MAP4K3	94
MAP4K4	MAP4K4	100
MAP4K5	MAP4K5	97
MAPKAPK2	MAPKAPK2	88
MAPKAPK5	MAPKAPK5	77
MARK1	MARK1	100
MARK2	MARK2	100
MARK3	MARK3	92
MARK4	MARK4	96
MAST1	MAST1	99
MEK1	MAP2K1	79
MEK2	MAP2K2	98
MEK3	MAP2K3	87
MEK4	MAP2K4	71
MEK5	MAP2K5	94
MEK6	MAP2K6	100
MELK	MELK	89
MERTK	MERTK	98

MET	MET	100
MET(M1250T)	MET	100
MET(Y1235D)	MET	95
MINK	MINK1	74
MKK7	MAP2K7	100
MKNK1	MKNK1	100
MKNK2	MKNK2	75
MLCK	MYLK3	98
MLK1	MAP3K9	100
MLK2	MAP3K10	89
MLK3	MAP3K11	91
MRCKA	CDC42BPA	100
MRCKB	CDC42BPB	94
MST1	STK4	100
MST1R	MST1R	90
MST2	STK3	100
MST3	STK24	100
MST4	MST4	86
MTOR	MTOR	84
MUSK	MUSK	100
MYLK	MYLK	94
MYLK2	MYLK2	84
MYLK4	MYLK4	90
MYO3A	MYO3A	91
MYO3B	MYO3B	92
NDR1	STK38	86
NDR2	STK38L	74
NEK1	NEK1	97
NEK10	NEK10	100
NEK11	NEK11	98
NEK2	NEK2	98
NEK3	NEK3	100
NEK4	NEK4	100
NEK5	NEK5	93
NEK6	NEK6	91
NEK7	NEK7	100
NEK9	NEK9	97
NIK	MAP3K14	56
NIM1	MGC42105	100
NLK	NLK	90
OSR1	OXSR1	75
p38-alpha	MAPK14	93
p38-beta	MAPK11	93
p38-delta	MAPK13	98

p38-gamma	MAPK12	100
PAK1	PAK1	96
PAK2	PAK2	100
PAK3	PAK3	96
PAK4	PAK4	93
PAK6	PAK6	100
PAK7	PAK7	97
PCTK1	CDK16	95
PCTK2	CDK17	99
PCTK3	CDK18	90
PDGFRA	PDGFRA	92
PDGFRB	PDGFRB	100
PDPK1	PDPK1	100
PFCDPK1(P.falciparum)	CDPK1	100
PFPK5(P.falciparum)	MAL13P1.279	69
PFTAIRE2	CDK15	98
PFTK1	CDK14	98
PHKG1	PHKG1	100
PHKG2	PHKG2	100
PIK3C2B	PIK3C2B	100
PIK3C2G	PIK3C2G	60
PIK3CA	PIK3CA	100
PIK3CA(C420R)	PIK3CA	100
PIK3CA(E542K)	PIK3CA	83
PIK3CA(E545A)	PIK3CA	100
PIK3CA(E545K)	PIK3CA	69
PIK3CA(H1047L)	PIK3CA	94
PIK3CA(H1047Y)	PIK3CA	94
PIK3CA(I800L)	PIK3CA	69
PIK3CA(M1043I)	PIK3CA	95
PIK3CA(Q546K)	PIK3CA	92
PIK3CB	PIK3CB	86
PIK3CD	PIK3CD	100
PIK3CG	PIK3CG	100
PIK4CB	PI4KB	41
PIKFYVE	PIKFYVE	100
PIM1	PIM1	100
PIM2	PIM2	100
PIM3	PIM3	92
PIP5K1A	PIP5K1A	93
PIP5K1C	PIP5K1C	80
PIP5K2B	PIP4K2B	96
PIP5K2C	PIP4K2C	86
PKAC-alpha	PRKACA	92

PKAC-beta	PRKACB	83
PKMYT1	PKMYT1	81
PKN1	PKN1	88
PKN2	PKN2	82
PKNB(M.tuberculosis)	pknB	88
PLK1	PLK1	100
PLK2	PLK2	100
PLK3	PLK3	100
PLK4	PLK4	91
PRKCD	PRKCD	100
PRKCE	PRKCE	99
PRKCH	PRKCH	87
PRKCI	PRKCI	88
PRKCQ	PRKCQ	100
PRKD1	PRKD1	100
PRKD2	PRKD2	100
PRKD3	PRKD3	100
PRKG1	PRKG1	90
PRKG2	PRKG2	98
PRKR	EIF2AK2	89
PRKX	PRKX	97
PRP4	PRPF4B	86
PYK2	PTK2B	100
QSK	KIAA0999	85
RAF1	RAF1	98
RET	RET	100
RET(M918T)	RET	87
RET(V804L)	RET	84
RET(V804M)	RET	100
RIOK1	RIOK1	100
RIOK2	RIOK2	87
RIOK3	RIOK3	100
RIPK1	RIPK1	91
RIPK2	RIPK2	100
RIPK4	RIPK4	98
RIPK5	DSTYK	91
ROCK1	ROCK1	99
ROCK2	ROCK2	97
ROS1	ROS1	92
RPS6KA4(Kin.Dom.1-N-terminal)	RPS6KA4	78
RPS6KA4(Kin.Dom.2-C-terminal)	RPS6KA4	94
RPS6KA5(Kin.Dom.1-N-terminal)	RPS6KA5	94
RPS6KA5(Kin.Dom.2-C-terminal)	RPS6KA5	91
RSK1(Kin.Dom.1-N-terminal)	RPS6KA1	100

RSK1(Kin.Dom.2-C-terminal)	RPS6KA1	100
RSK2(Kin.Dom.1-N-terminal)	RPS6KA3	100
RSK2(Kin.Dom.2-C-terminal)	RPS6KA3	98
RSK3(Kin.Dom.1-N-terminal)	RPS6KA2	100
RSK3(Kin.Dom.2-C-terminal)	RPS6KA2	99
RSK4(Kin.Dom.1-N-terminal)	RPS6KA6	81
RSK4(Kin.Dom.2-C-terminal)	RPS6KA6	99
S6K1	RPS6KB1	100
SBK1	SBK1	70
SGK	SGK1	98
SgK110	SgK110	94
SGK2	SGK2	95
SGK3	SGK3	59
SIK	SIK1	94
SIK2	SIK2	100
SLK	SLK	88
SNARK	NUAK2	66
SNRK	SNRK	93
SRC	SRC	98
SRMS	SRMS	95
SRPK1	SRPK1	100
SRPK2	SRPK2	98
SRPK3	SRPK3	98
STK16	STK16	100
STK33	STK33	86
STK35	STK35	100
STK36	STK36	100
STK39	STK39	100
SYK	SYK	81
TAK1	MAP3K7	100
TAOK1	TAOK1	100
TAOK2	TAOK2	97
TAOK3	TAOK3	100
TBK1	TBK1	100
TEC	TEC	90
TESK1	TESK1	79
TGFBR1	TGFBR1	100
TGFBR2	TGFBR2	92
TIE1	TIE1	100
TIE2	TEK	91
TLK1	TLK1	100
TLK2	TLK2	93
TNIK	TNIK	100
TNK1	TNK1	89

TNK2	TNK2	87
TNNI3K	TNNI3K	100
TRKA	NTRK1	100
TRKB	NTRK2	97
TRKC	NTRK3	100
TRPM6	TRPM6	100
TSSK1B	TSSK1B	100
TSSK3	TSSK3	93
TTK	TTK	100
TXK	TXK	86
TYK2(JH1domain-catalytic)	TYK2	100
TYK2(JH2domain-pseudokinase)	TYK2	97
TYRO3	TYRO3	93
ULK1	ULK1	86
ULK2	ULK2	83
ULK3	ULK3	100
VEGFR2	KDR	99
VPS34	PIK3C3	68
VRK2	VRK2	100
WEE1	WEE1	100
WEE2	WEE2	99
WNK1	WNK1	94
WNK2	WNK2	92
WNK3	WNK3	95
WNK4	WNK4	81
YANK1	STK32A	76
YANK2	STK32B	85
YANK3	STK32C	80
YES	YES1	88
YSK1	STK25	99
YSK4	MAP3K19	73
ZAK	ZAK	89
ZAP70	ZAP70	100

Figure S1 | Thermal ellipsoids for intermediate **39**.

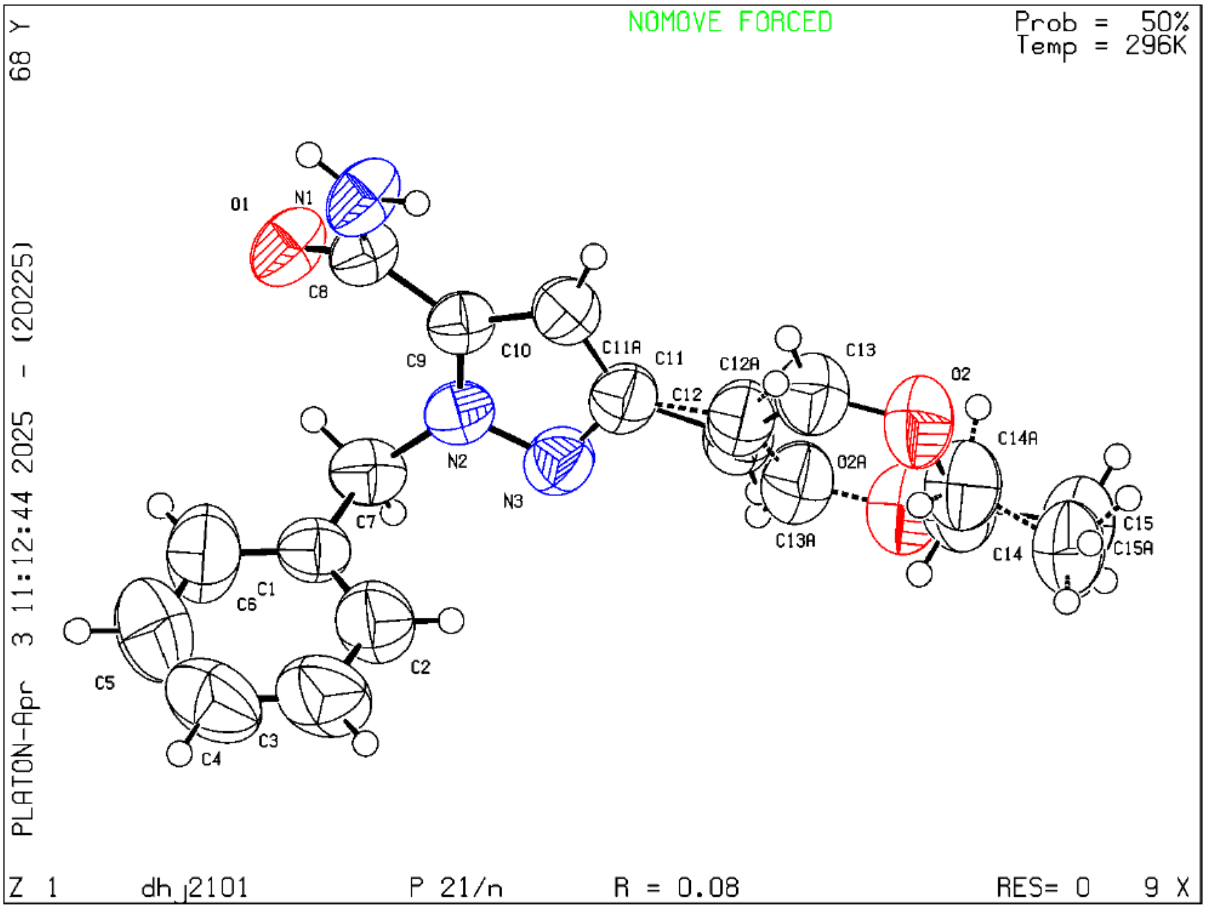

Figure S2 | Thermal ellipsoids for intermediate **49**.

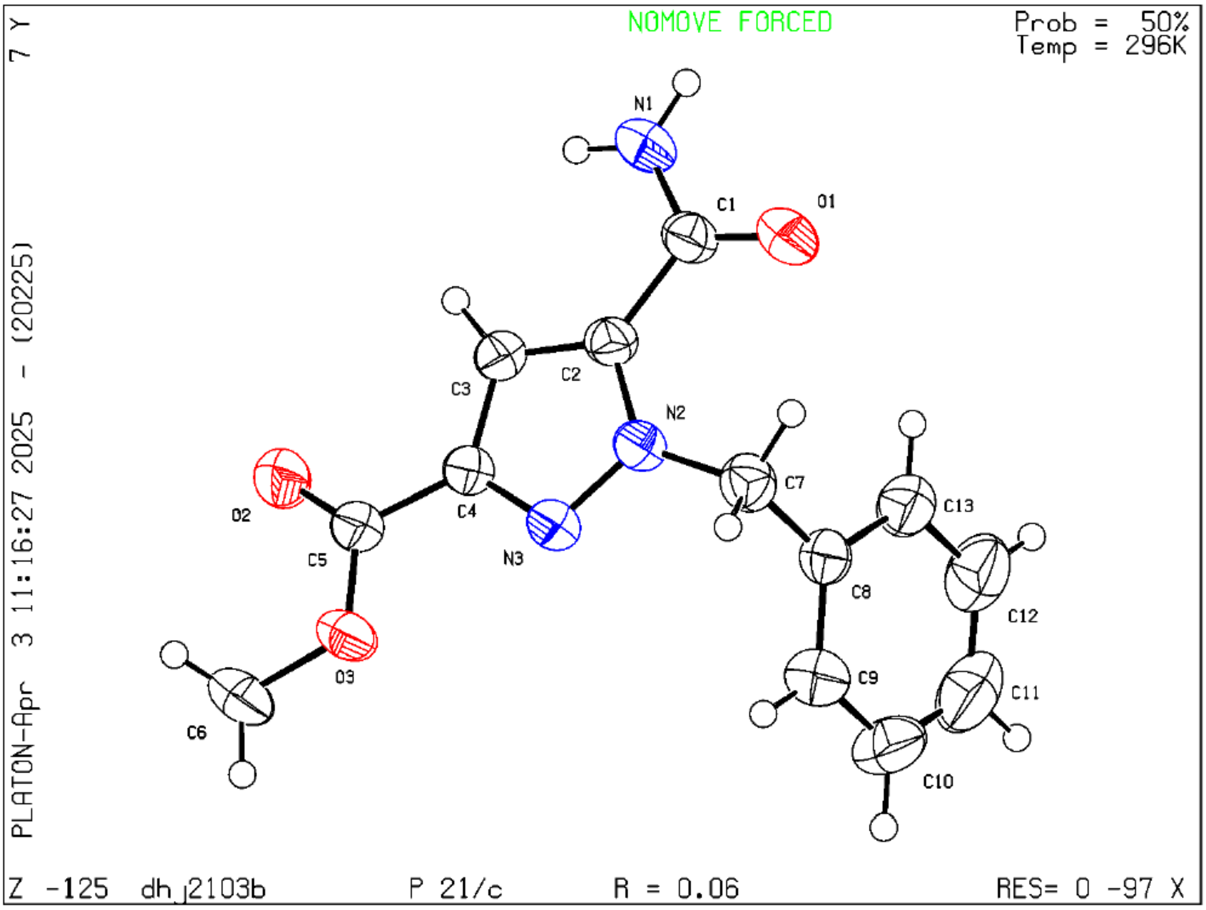

Figure S3 | Thermal ellipsoids for MDI-117740 (**69**).

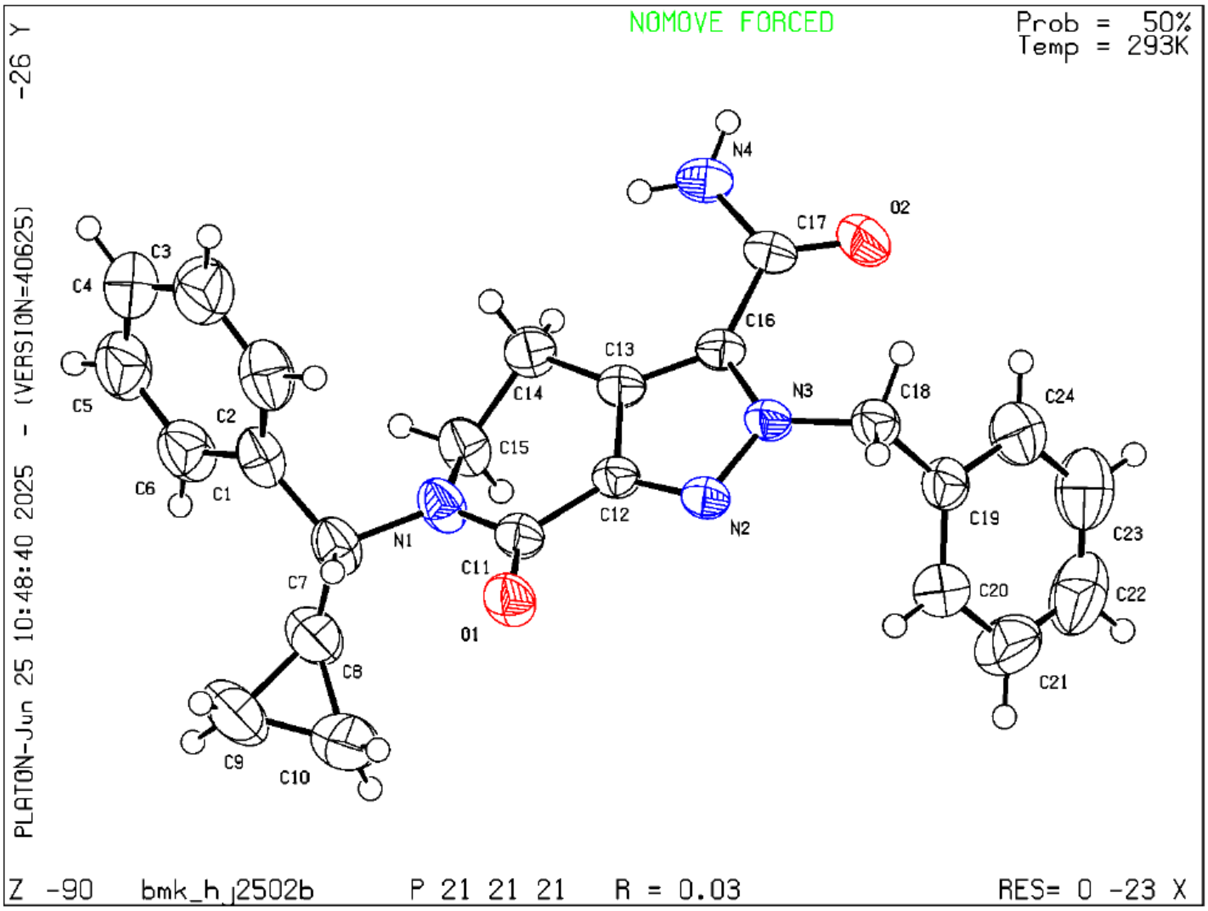

Table S2 | Statistical data used to compare effect of LIMK inhibitors on cell migration in a wound healing assay in MDA-MB-231 cells. Ordinary one-way ANOVA summary results from GraphPad Prism shown for wound healing after 48 h for MDI-117740 (**69**, *n* = 4) and FRAX486 (**2**, *n* = 4). The test statistics for control and test samples are reported.

Compound	*F* value	*P* value	*P* value summary	Significant diff. among means ($p < 0.05$)?	*R* squared
MDI-117740 (**69**)	5.144	0.0104	*	Yes	0.6128
FRAX486 (**2**)	8.835	0.0011	**	Yes	0.7311

Control vs Compound	Mean difference	Adjusted *P* value	*P* value
MDI-117740 (**69**)			
Untreated vs. 0.25 µM	29.92	0.0493	*
Untreated vs. 1 µM	32.72	0.0193	*
Untreated vs. 3 µM	42.05	0.0060	**
Untreated vs. Negative control (**57**, 3 µM)	14.05	0.4603	ns
FRAX486 (**2**)			
Untreated vs. 0.25 µM	30.85	0.0146	*
Untreated vs. 1 µM	38.37	0.0016	**
Untreated vs. 3 µM	43.70	0.0011	**
Untreated vs. Negative control (**57**, 3 µM)	14.05	0.3124	ns

^{1}H, ^{13}C, COSY, HSQC, HMBC NMR and UPLC data for final compounds

Confirming regiochemistry by ^{1}H NMR

During the course of our investigations, we consistently observed the amidic NH_2 protons coalesce (br s, 2H) for 1-benzylpyrazolo-5-carboxamides (*e.g.* **34**) whereas the amidic NH_2 protons split (2 x br s, 1H) for 1-benzylpyrazolo-3-carboxamides (*e.g.* **57**, Figure S4), which was corroborated by HMBC experiments. As a result, regiochemical assignment was possible in the absence of HMBC experiments.

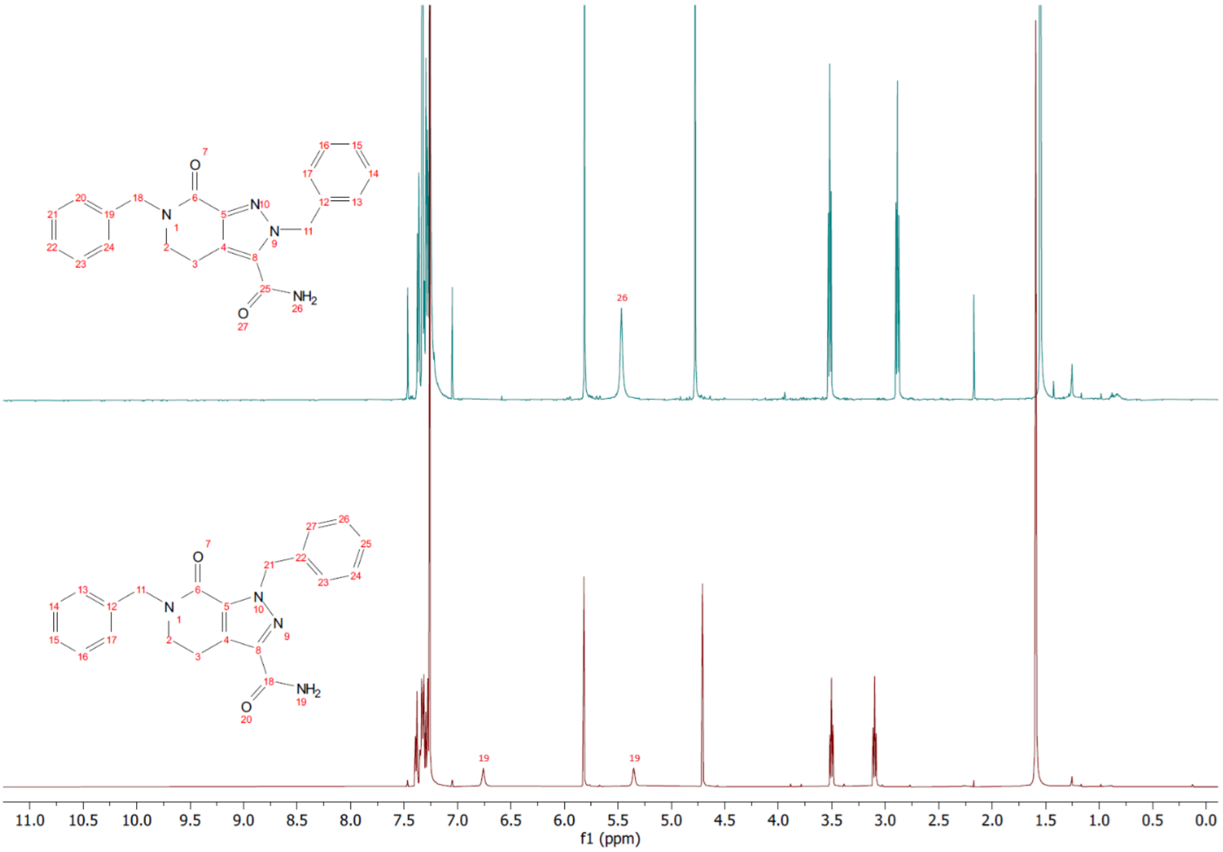

Figure S4 | Stacked ^{1}H NMR spectra of **34** (blue line) and regioisomer **57** (red line) in $CDCl_3$. Amidic NH_2 protons are highlighted and numbered according to structural numbering.

(*S*)-3-(2-Benzyl-3-chloro-7-oxo-2,4,5,7-tetrahydro-6*H*-pyrazolo[3,4-*c*]pyridin-6-yl)-5-methyl-4-oxo-2,3,4,5-tetrahydrobenzo[*b*][1,4]oxazepine-8-carbonitrile (**8**, compound 22)

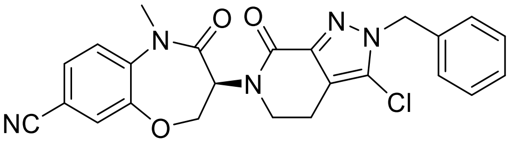

Purchased from MedChemExpress (Cat. No.: HY-111409).

^{1}H NMR (500 MHz, $CDCl_3$) δ 7.57 (dd, *J* = 8.3, 1.9 Hz, 1H), 7.48 (dd, *J* = 1.9, 0.4 Hz, 1H), 7.35 – 7.28 (m, 4H), 7.28 – 7.24 (m, 2H), 5.88 (dd, *J* = 11.9, 8.0 Hz, 1H), 5.39 (s, 2H), 4.69 (dd, *J* = 11.9, 10.1 Hz, 1H), 4.45 (dd, *J* = 10.0, 8.0 Hz, 1H), 4.21 (dt, *J* = 12.1, 5.2 Hz, 1H), 3.56 (ddd, *J* = 12.0, 10.3, 4.4 Hz, 1H), 3.39 (s, 3H), 3.05 (ddd, *J* = 15.5, 10.3, 5.1 Hz, 1H), 2.69 (dt, *J* = 15.7, 4.9 Hz, 1H). ACQUITY UPLC® BEH C_{18} 1.7 µm: Rt = 1.76 min; *m/z* 462.1 [M+H, ^{35}Cl]$^+$. ELSD/UV/^{1}H NMR purity: 100/96/95%.

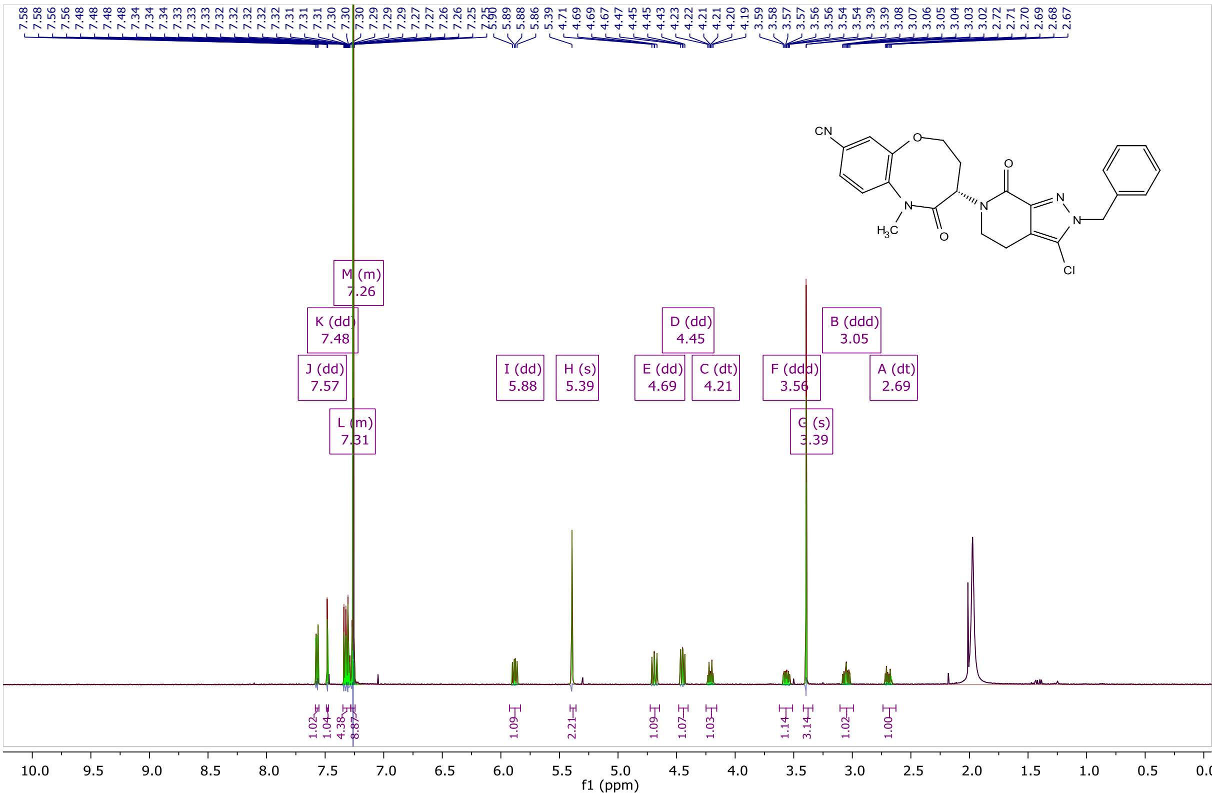

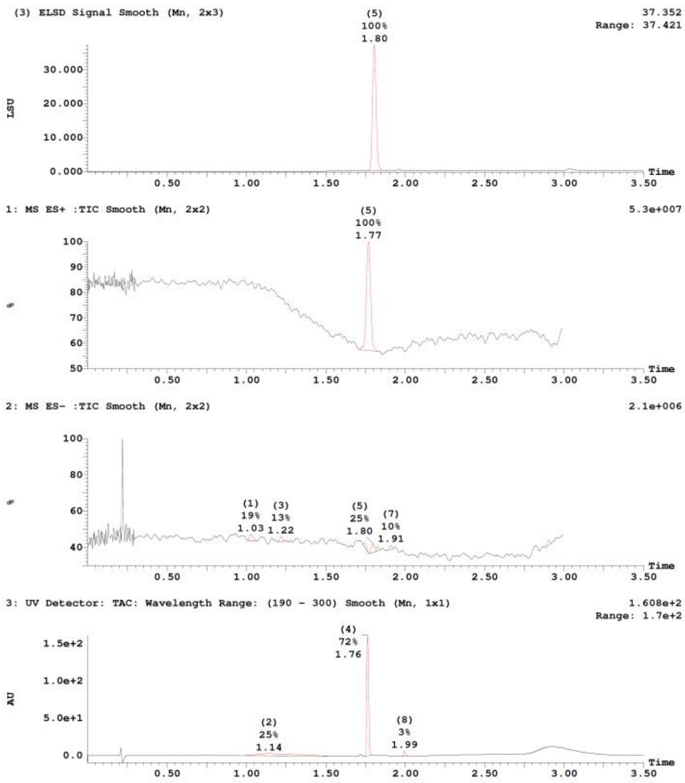

Peak ID	Compound	Time	Mass Found
5		1.77	Not Found

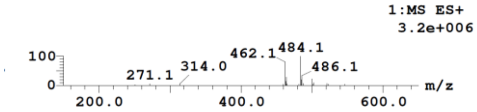

(*S*)-3-Benzyl-*N*-(5-methyl-4-oxo-2,3,4,5-tetrahydrobenzo[*b*][1,4]oxazepin-3-yl)-1*H*-1,2,4-triazole-5-carboxamide (**9**, GSK2982772)

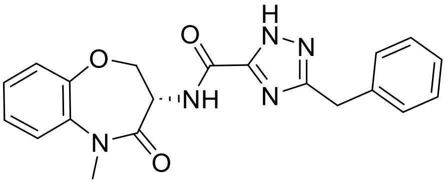

Supplied by MedChemExpress (Cat. No.: HY-101760).

Brown solid. ^{1}H NMR (400 MHz, DMSO-d_6) δ 14.36 (s, 1H), 8.39 (s, 1H), 7.50 (d, *J* = 7.7 Hz, 1H), 7.38 – 7.19 (m, 8H), 4.88 – 4.76 (m, 1H), 4.66 – 4.50 (m, 1H), 4.45 – 4.36 (m, 1H), 4.11 (s, 2H). CH_3 not observed as overlapping with HDO peak. ACQUITY UPLC® BEH C18 1.7 µm: Rt = 1.58 min; *m/z* 378.1 $[M+H]^+$. ELSD/UV/^{1}H NMR purity: 100/96/99%.

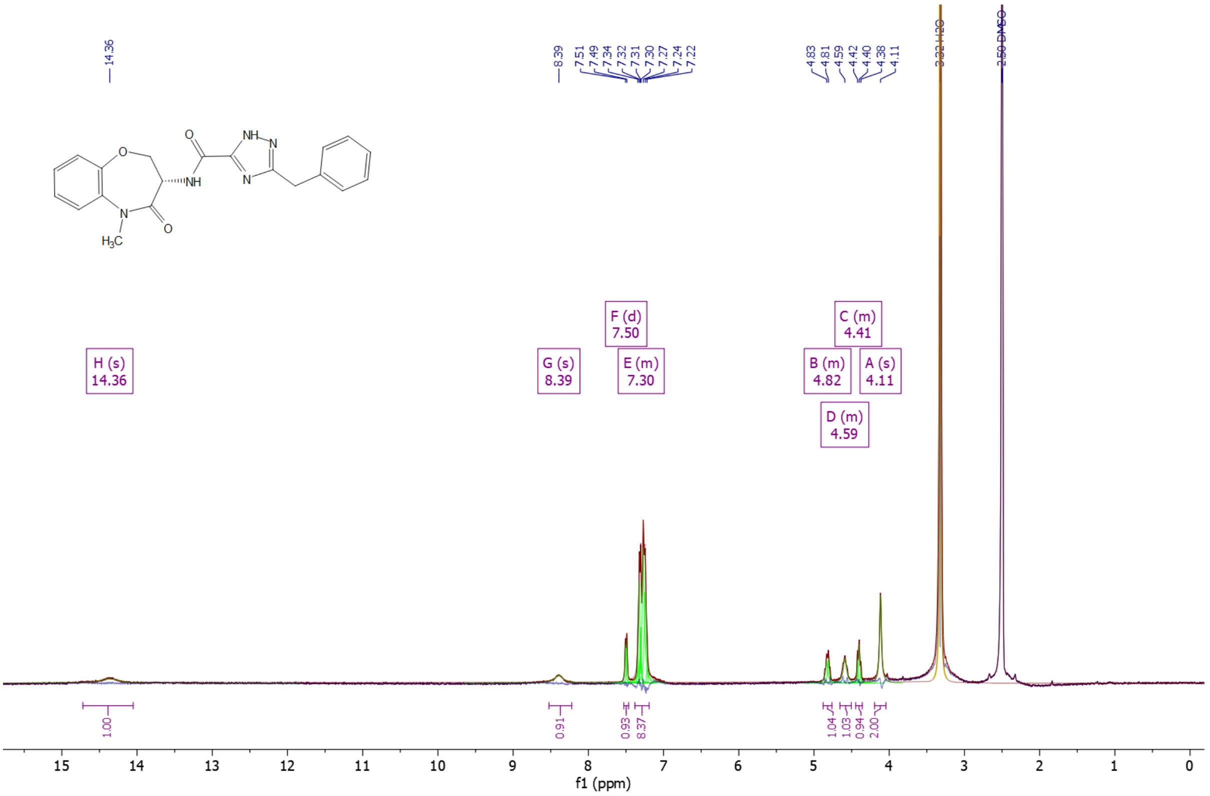

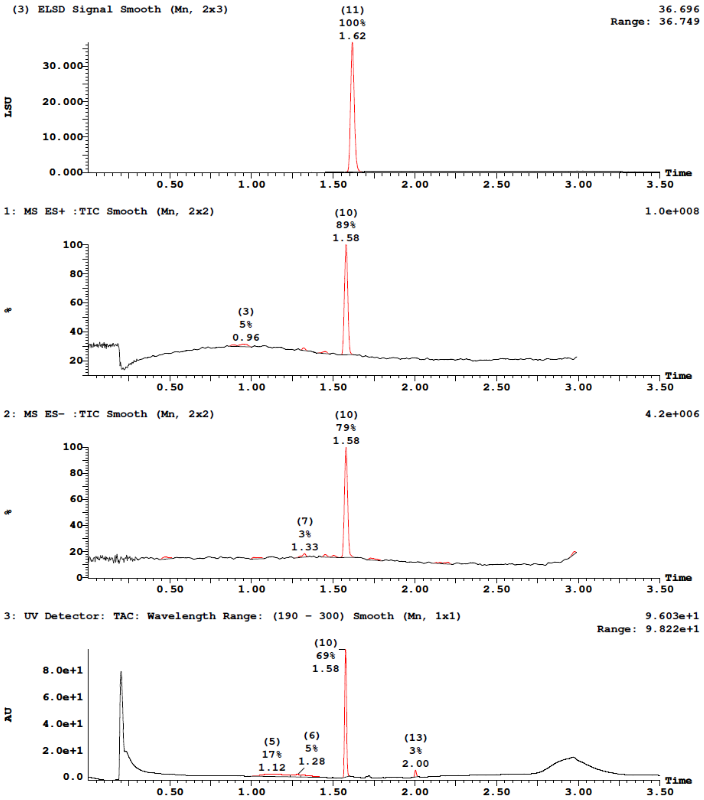

Peak ID	Compound	Time	Mass Found
10		1.58	Not Found

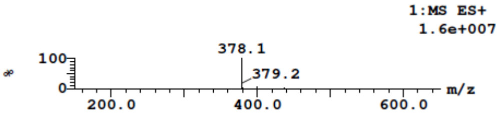

(*S*)-5-Benzyl-*N*-(5-methyl-4-oxo-2,3,4,5-tetrahydrobenzo[*b*][1,4]oxazepin-3-yl)isoxazole-3-carboxamide (**10**, GSK'481)

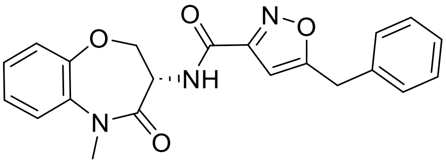

Supplied by MedChemExpress (Cat. No.: HY-100131).

Brown solid. ^{1}H NMR (400 MHz, DMSO-d_6) δ 8.84 (d, *J* = 8.2 Hz, 1H), 7.54 – 7.47 (m, 1H), 7.42 – 7.17 (m, 8H), 6.54 (s, 1H), 4.83 (q, *J* = 9.0 Hz, 1H), 4.57 (t, *J* = 10.7 Hz, 1H), 4.47 – 4.34 (m, 1H), 4.21 (s, 2H), 3.30 (s, 3H). ACQUITY UPLC® BEH C18 1.7 µm: Rt = 1.78 min; *m/z* 378.1 [M+H]$^+$. ELSD/UV/^{1}H NMR purity: 100/94/100%.

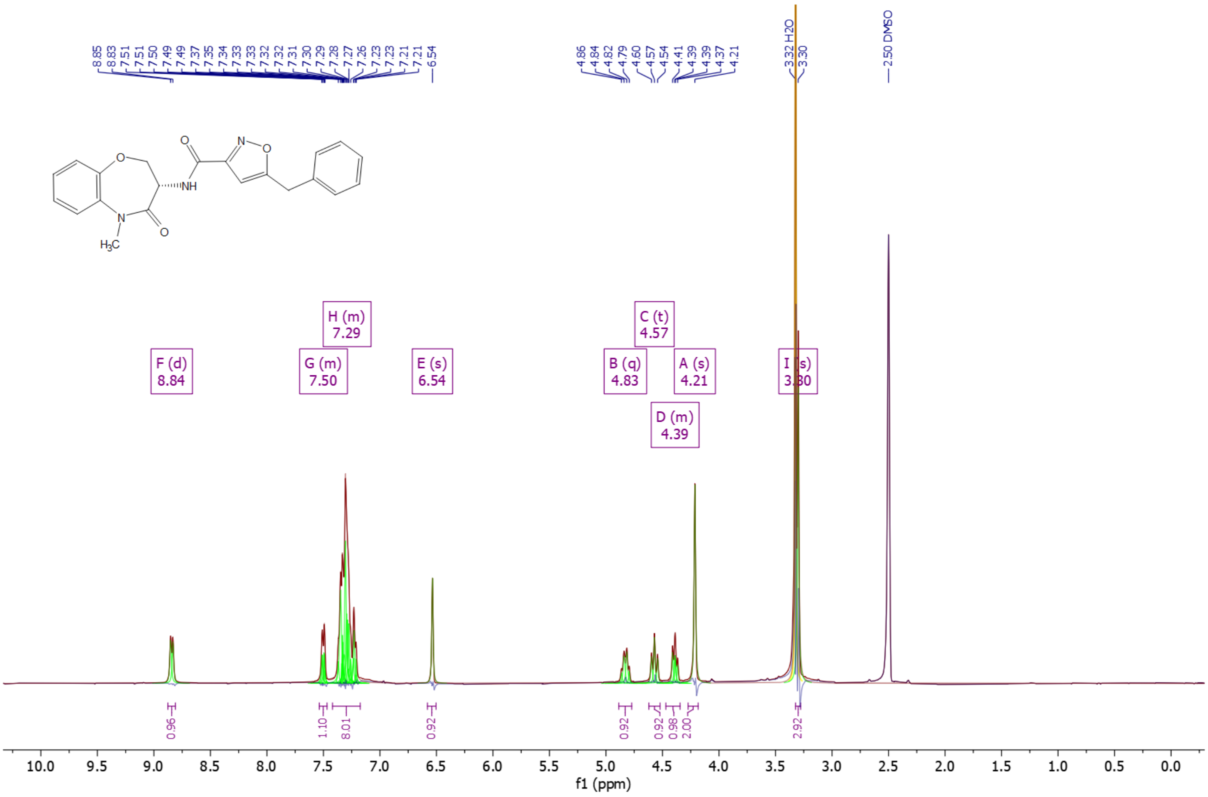

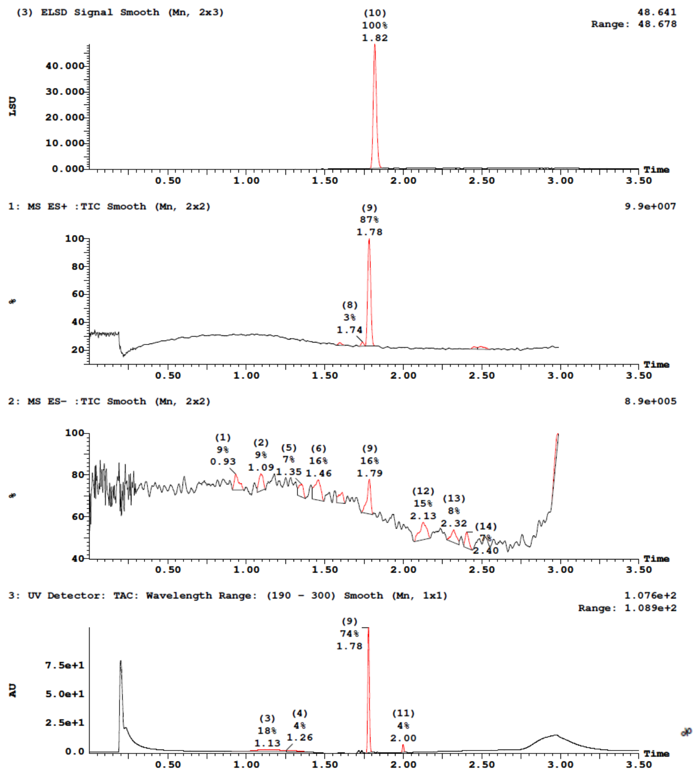

Peak ID	Compound	Time	Mass Found
9		1.78	Not Found

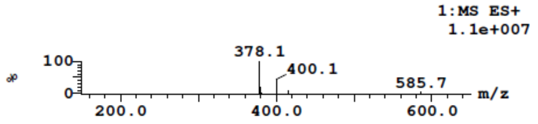

(*S*)-3-Benzyl-*N*-(7,9-difluoro-2-oxo-2,3,4,5-tetrahydro-1H-benzo[*b*]azepin-3-yl)-1*H*-1,2,4-triazole-5-carboxamide (**11**, GSK3145095)

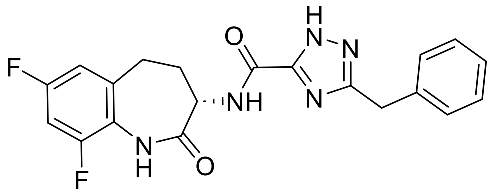

Supplied by MedChemExpress (Cat. No.: HY-111946).

Colourless liquid. ACQUITY UPLC® BEH C18 1.7 µm: Rt = 1.53 min; *m/z* 398.1 $[M+H]^+$. ELSD/UV/^{1}H NMR purity: 100/95%.

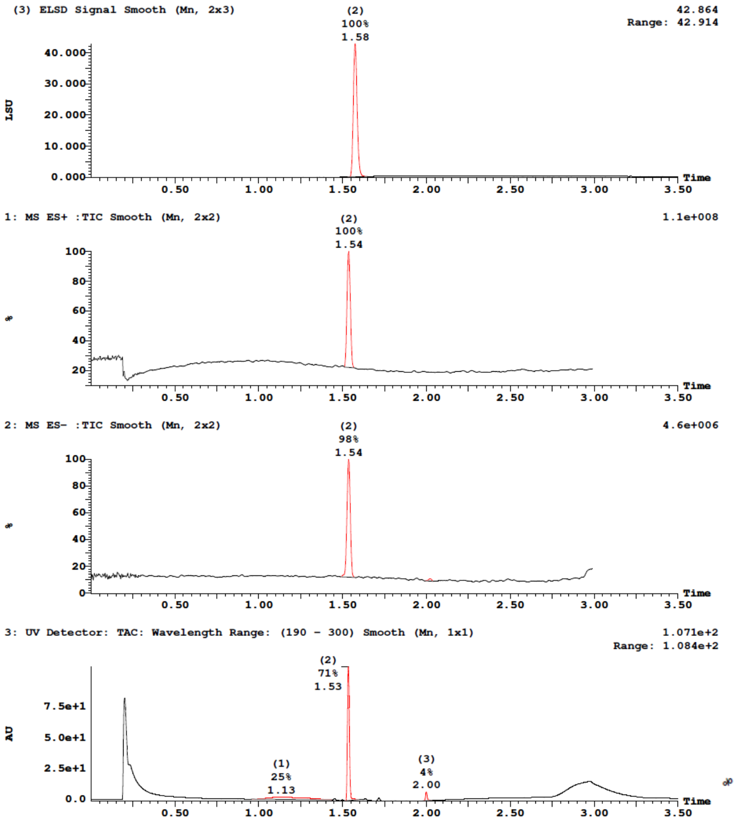

Peak ID	Compound	Time	Mass Found
2		1.54	Not Found

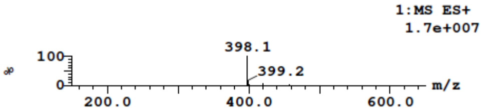

(*S*)-3-(2-Benzyl-3-bromo-7-oxo-2,4,5,7-tetrahydro-6*H*-pyrazolo[3,4-*c*]pyridin-6-yl)-8-chloro-5-methyl-2,3-dihydrobenzo[*b*][1,4]oxazepin-4(5*H*)-one (**19**)

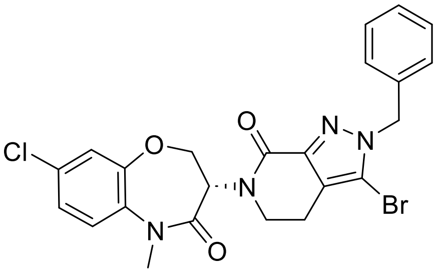

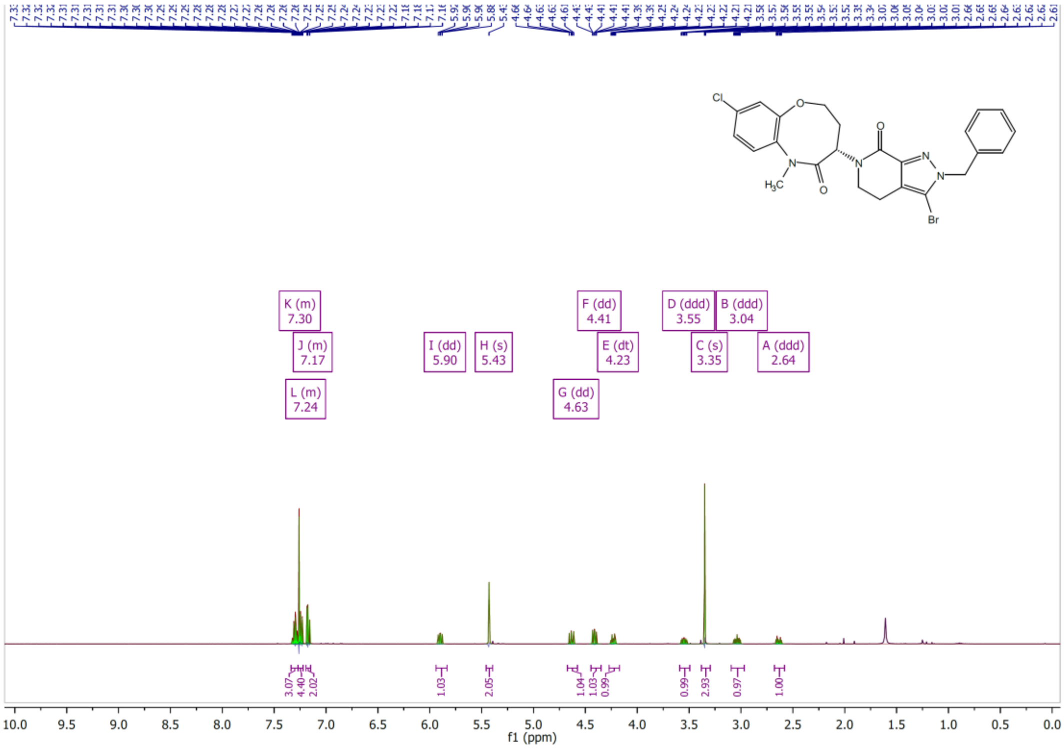

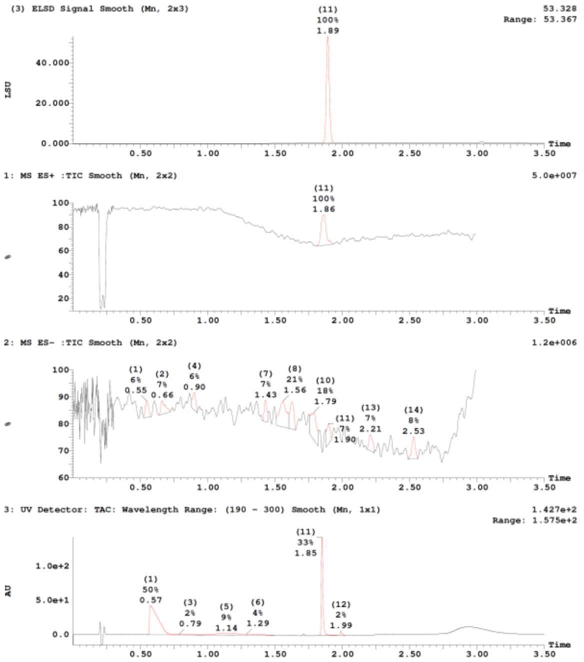

Peak ID	Compound	Time	Mass Found
11		1.86	Not Found

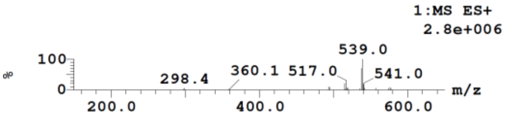

(*S*)-1-Benzyl-6-(8-chloro-5-methyl-4-oxo-2,3,4,5-tetrahydrobenzo[*b*][1,4]oxazepin-3-yl)-7-oxo-4,5,6,7-tetrahydro-1*H*-pyrazolo[3,4-*c*]pyridine-3-carboxamide (**20**)

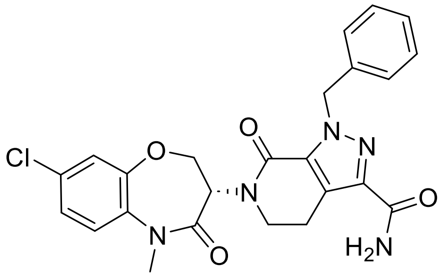

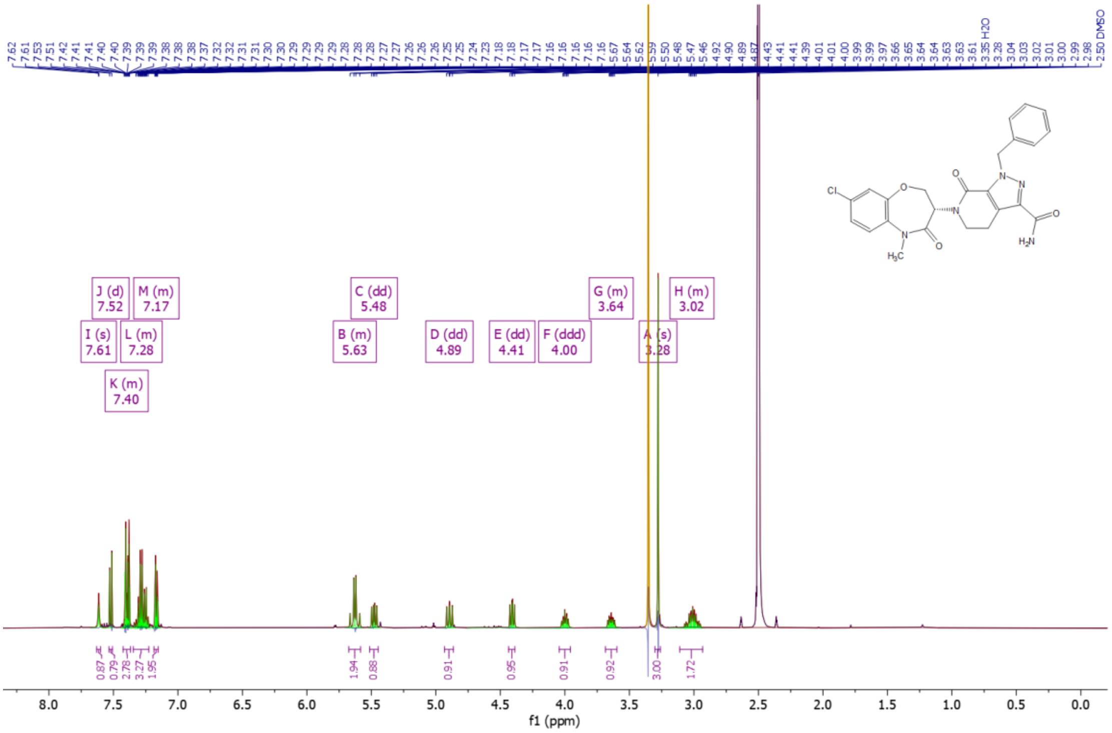

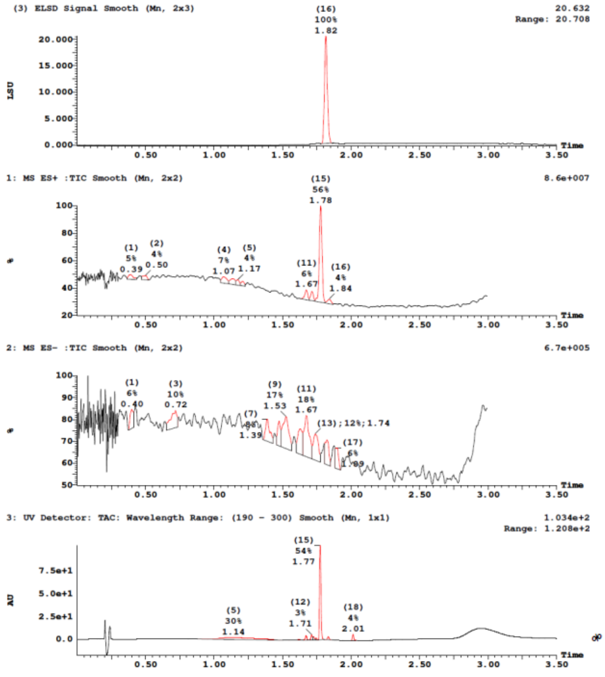

Peak ID	Compound	Time	Mass Found
15		1.78	Not Found

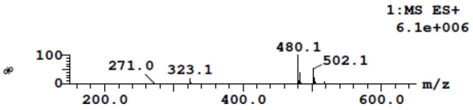

(*S*)-2-Benzyl-6-(5-methyl-4-oxo-2,3,4,5-tetrahydrobenzo[*b*][1,4]oxazepin-3-yl)-7-oxo-4,5,6,7-tetrahydro-2*H*-pyrazolo[3,4-*c*]pyridine-3-carboxamide (**21**)

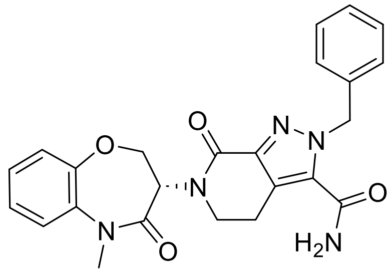

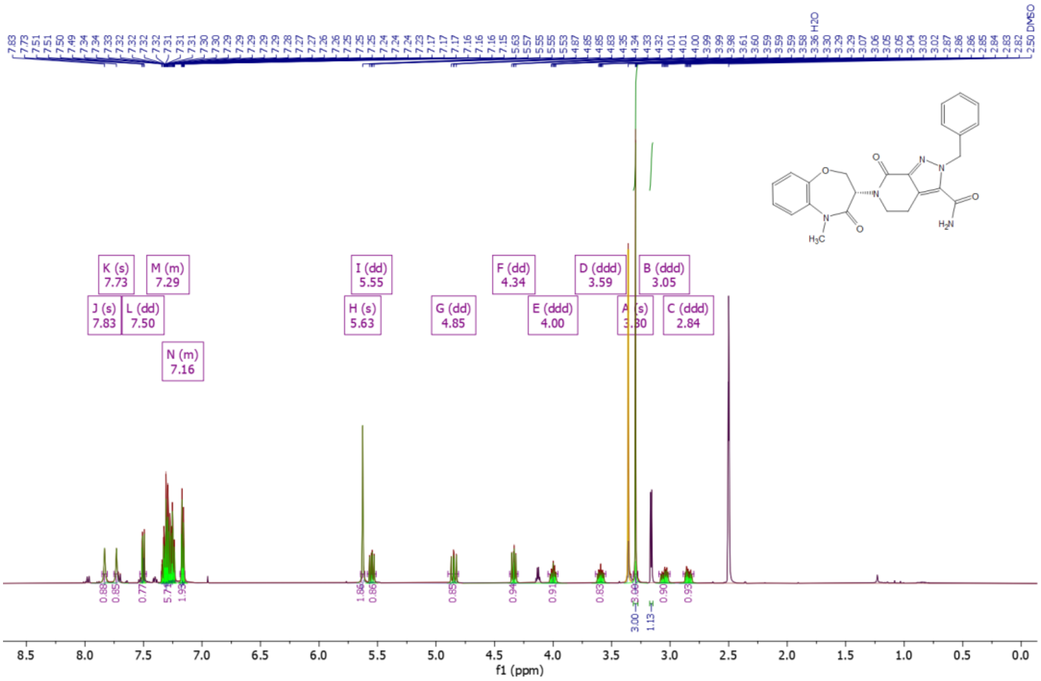

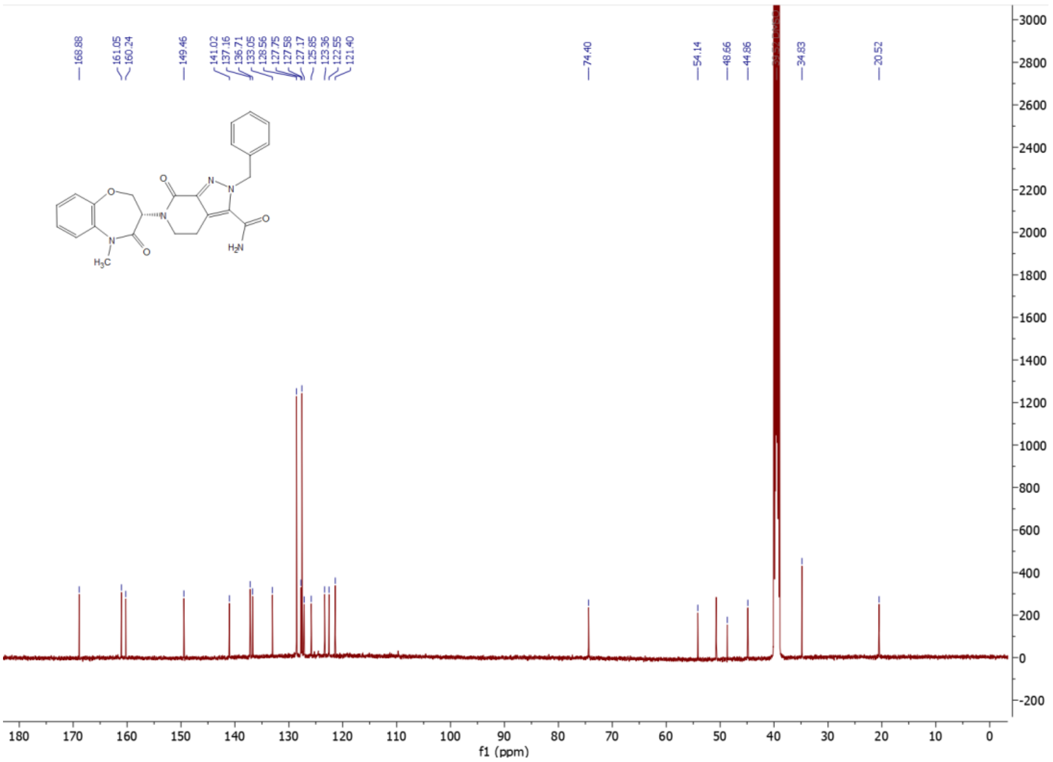

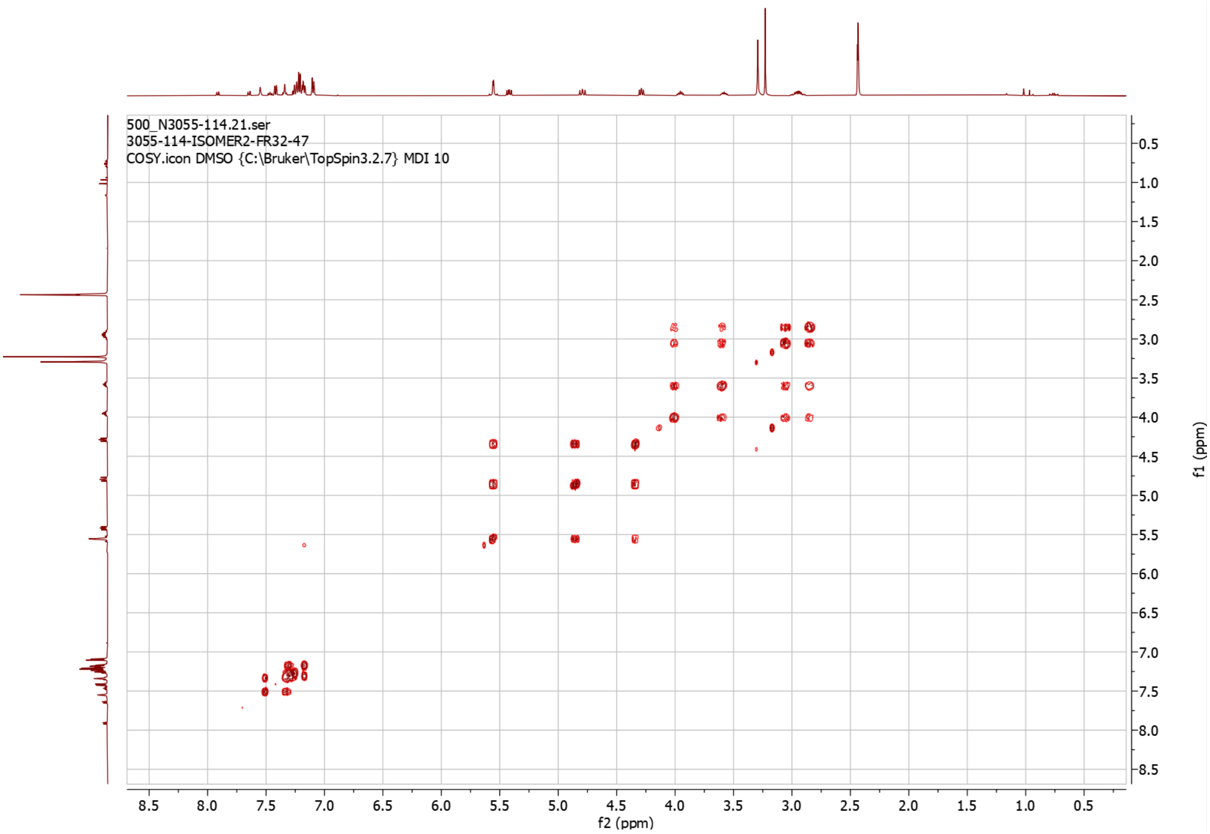
500_N3055-114.21.ser
3055-114-ISOMER2-FR32-47
COSY.icon DMSO {C:\Bruker\TopSpin3.2.7} MDI 10
f2 (ppm)
f1 (ppm)

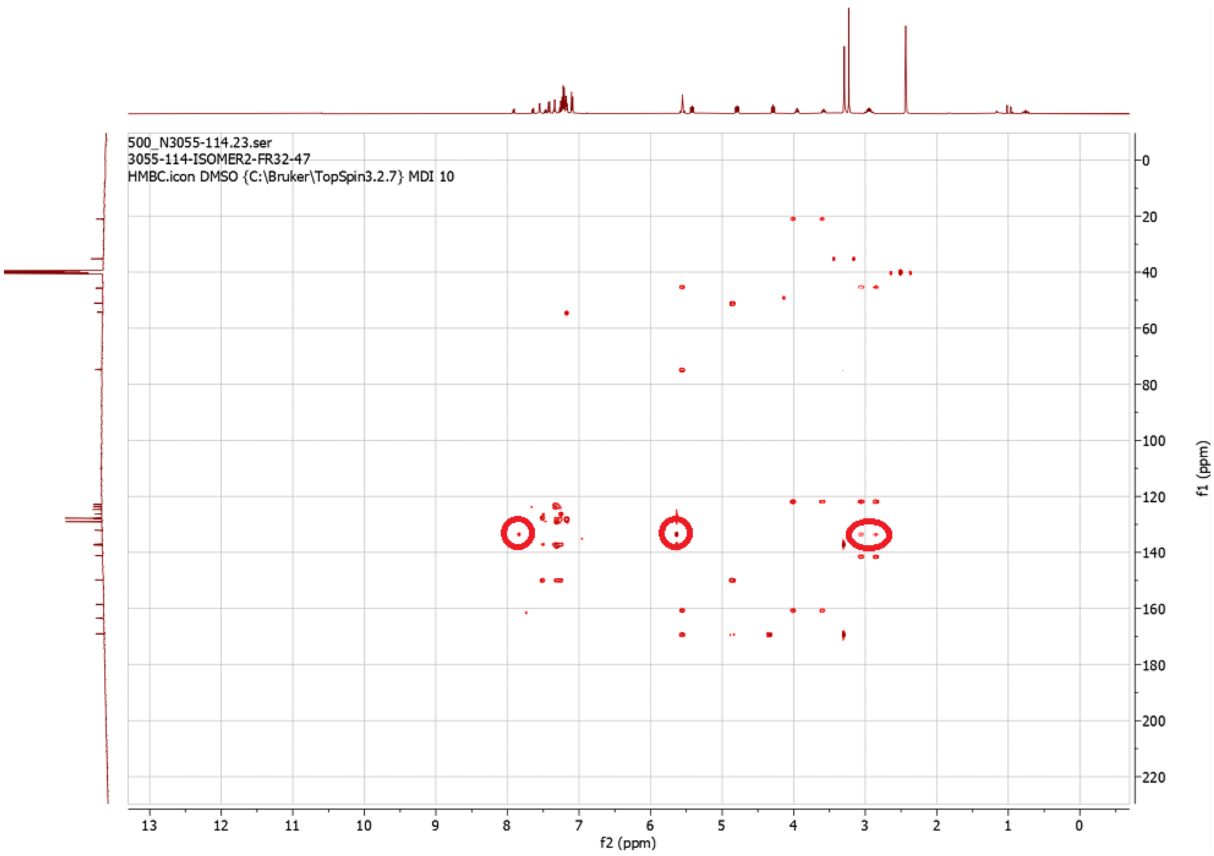
500_N3055-114.23.ser
3055-114-ISOMER2-FR32-47
HMBC.icon DMSO {C:\Bruker\TopSpin3.2.7} MDI 10
f2 (ppm)
f1 (ppm)

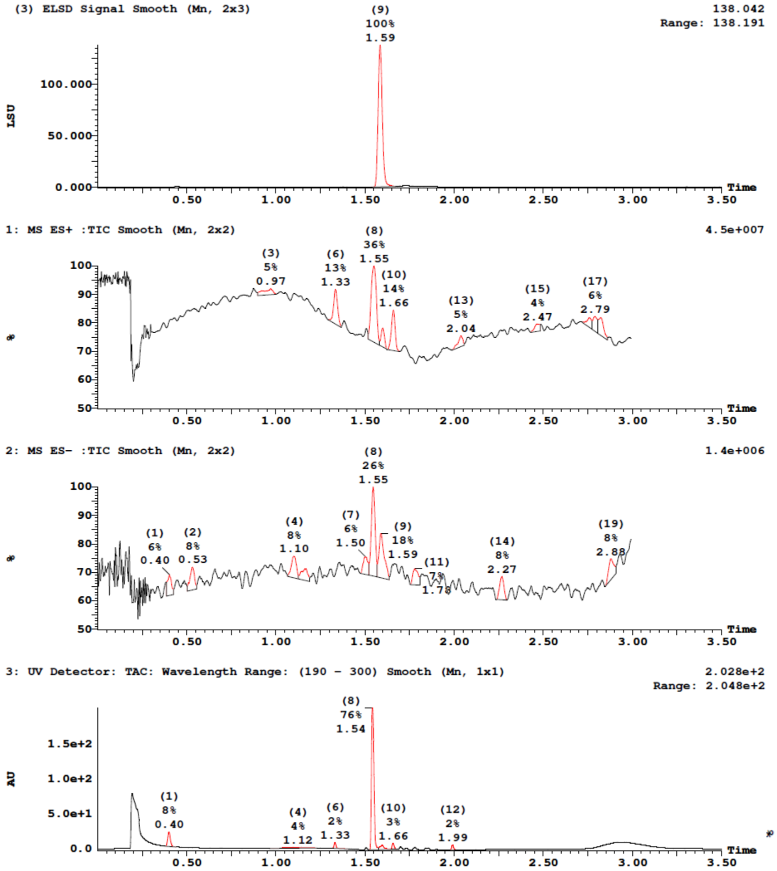

Peak ID	Compound	Time	Mass Found
8		1.55	Not Found

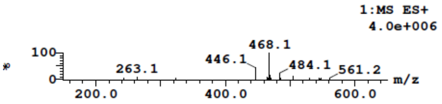

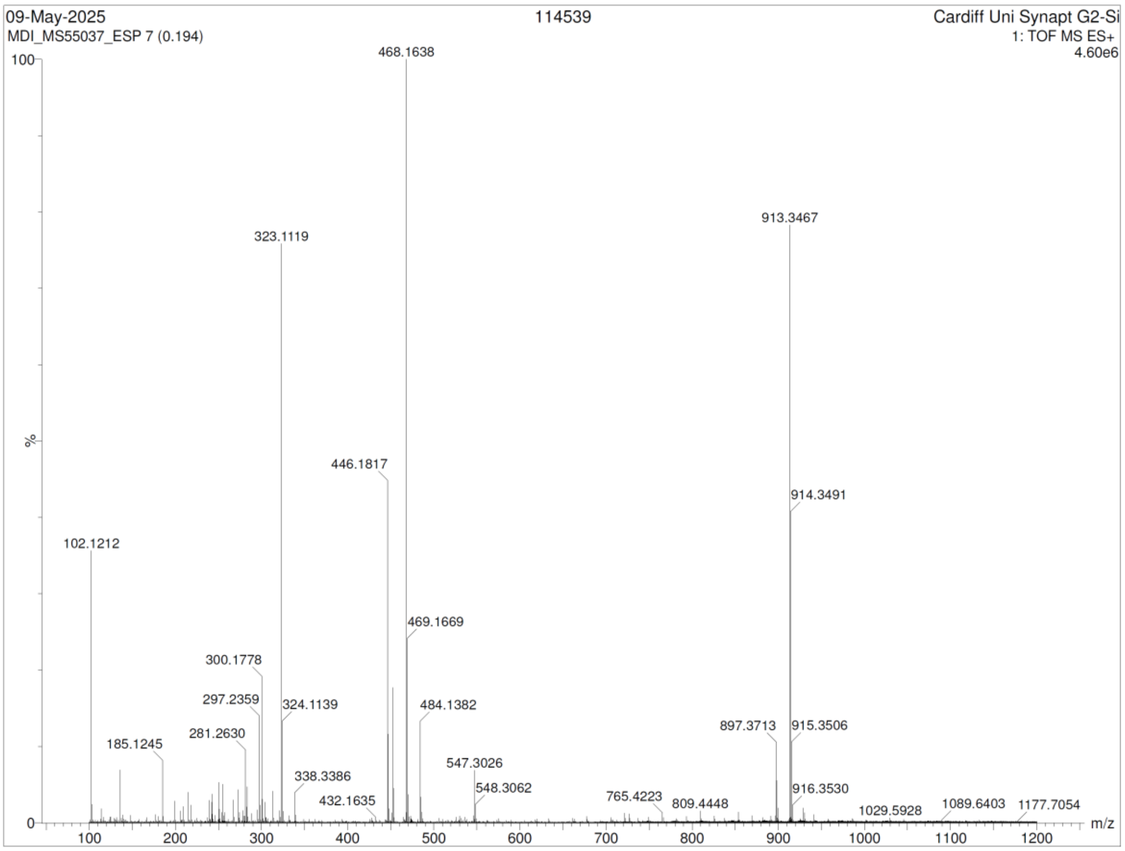

Elemental Composition Report

Single Mass Analysis

Tolerance = 10.0 PPM / DBE: min = -1.5, max = 50.0
Element prediction: Off
Number of isotope peaks used for i-FIT = 3

Monoisotopic Mass, Odd and Even Electron Ions
25 formula(e) evaluated with 1 results within limits (up to 50 closest results for each mass)
Elements Used:
C: 0-24 H: 0-24 N: 0-5 O: 0-4

09-May-2025
MDI_MS55037_ESP 7 (0.194)

114539

Cardiff Uni Synapt G2-Si
1: TOF MS ES+
2.06e+006

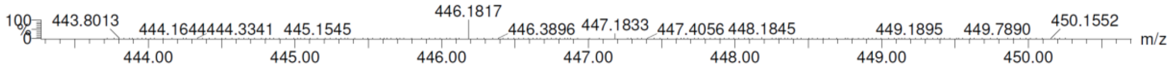

Minimum:				-1.5				
Maximum:		5.0	10.0	50.0				
Mass	Calc. Mass	mDa	PPM	DBE	i-FIT	Norm	Conf(%)	Formula
446.1817	446.1828	-1.1	-2.5	15.5	932.7	n/a	n/a	C24 H24 N5 O4

(*S*)-1-Benzyl-6-(5-methyl-4-oxo-2,3,4,5-tetrahydrobenzo[*b*][1,4]oxazepin-3-yl)-7-oxo-4,5,6,7-tetrahydro-1*H*-pyrazolo[3,4-*c*]pyridine-3-carboxamide (**22**)

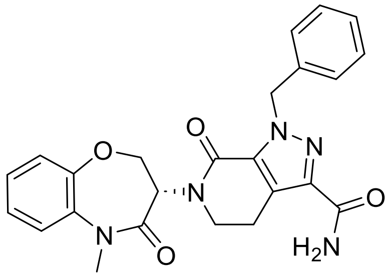

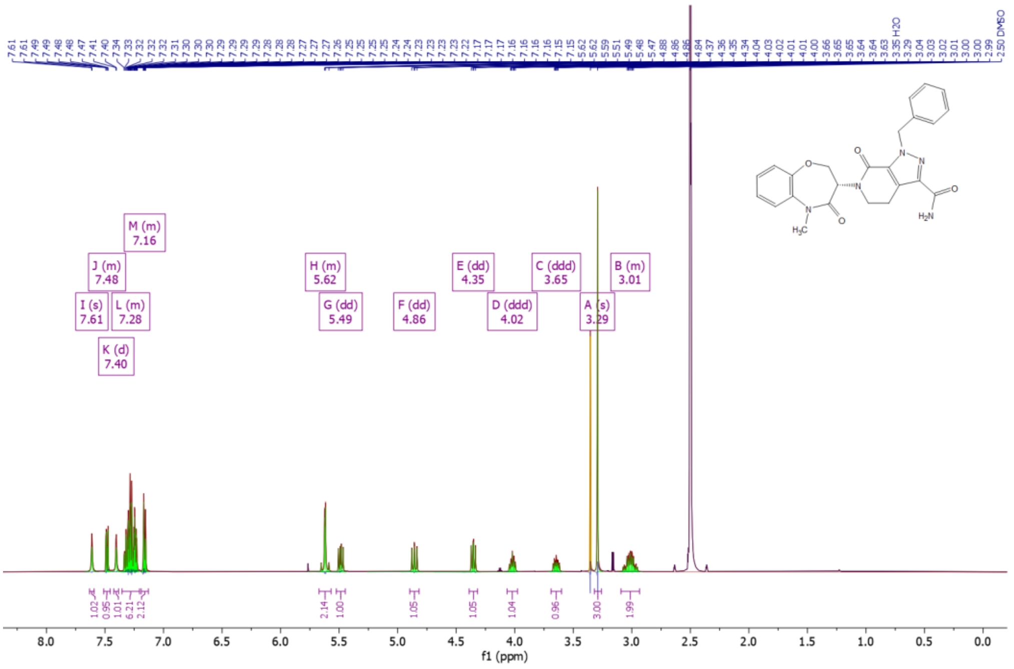

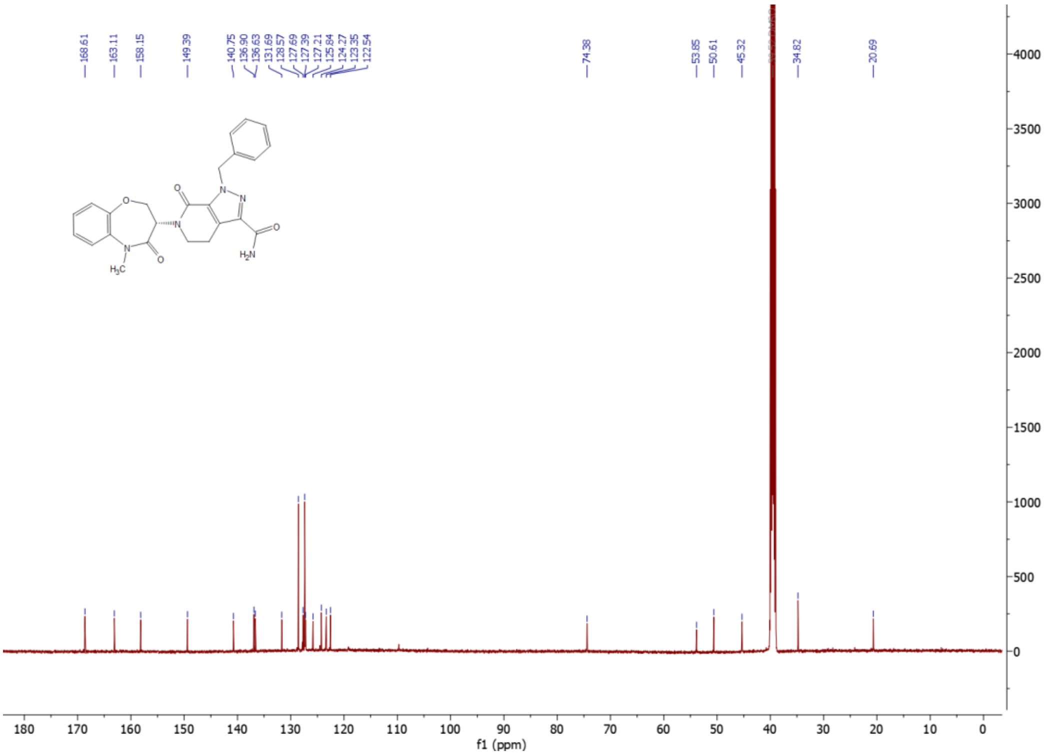

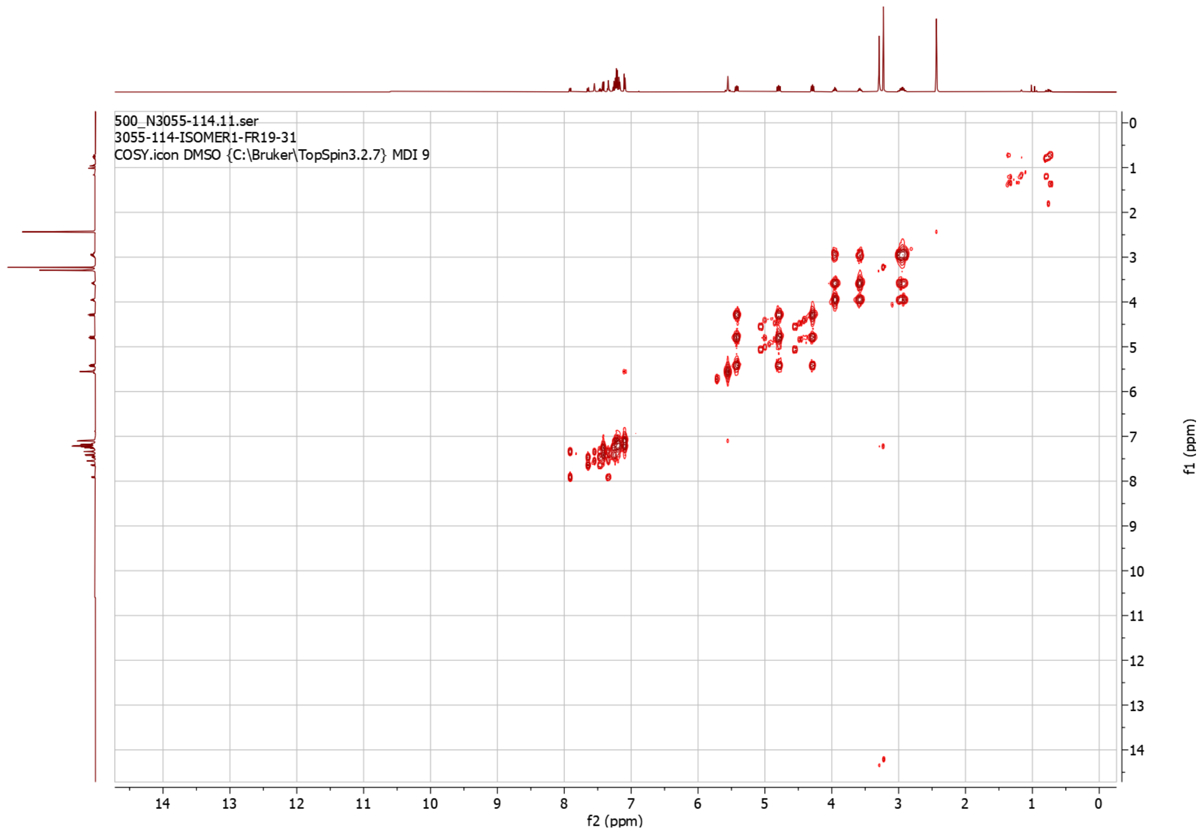
500_N3055-114.11.ser
3055-114-ISOMER1-FR19-31
COSY.icon DMSO {C:\Bruker\TopSpin3.2.7} MDI 9
f2 (ppm)
f1 (ppm)

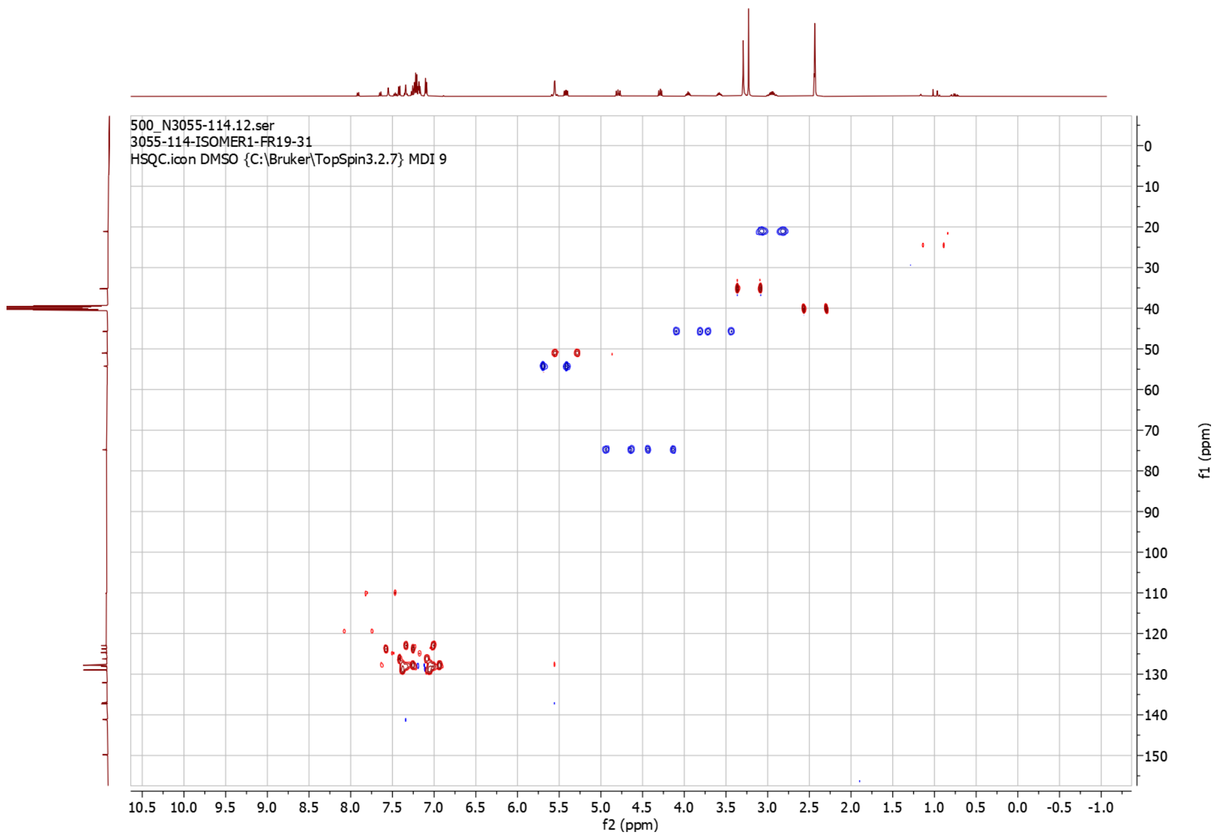
500_N3055-114.12.ser
3055-114-ISOMER1-FR19-31
HSQC.icon DMSO {C:\Bruker\TopSpin3.2.7} MDI 9
f2 (ppm)
f1 (ppm)

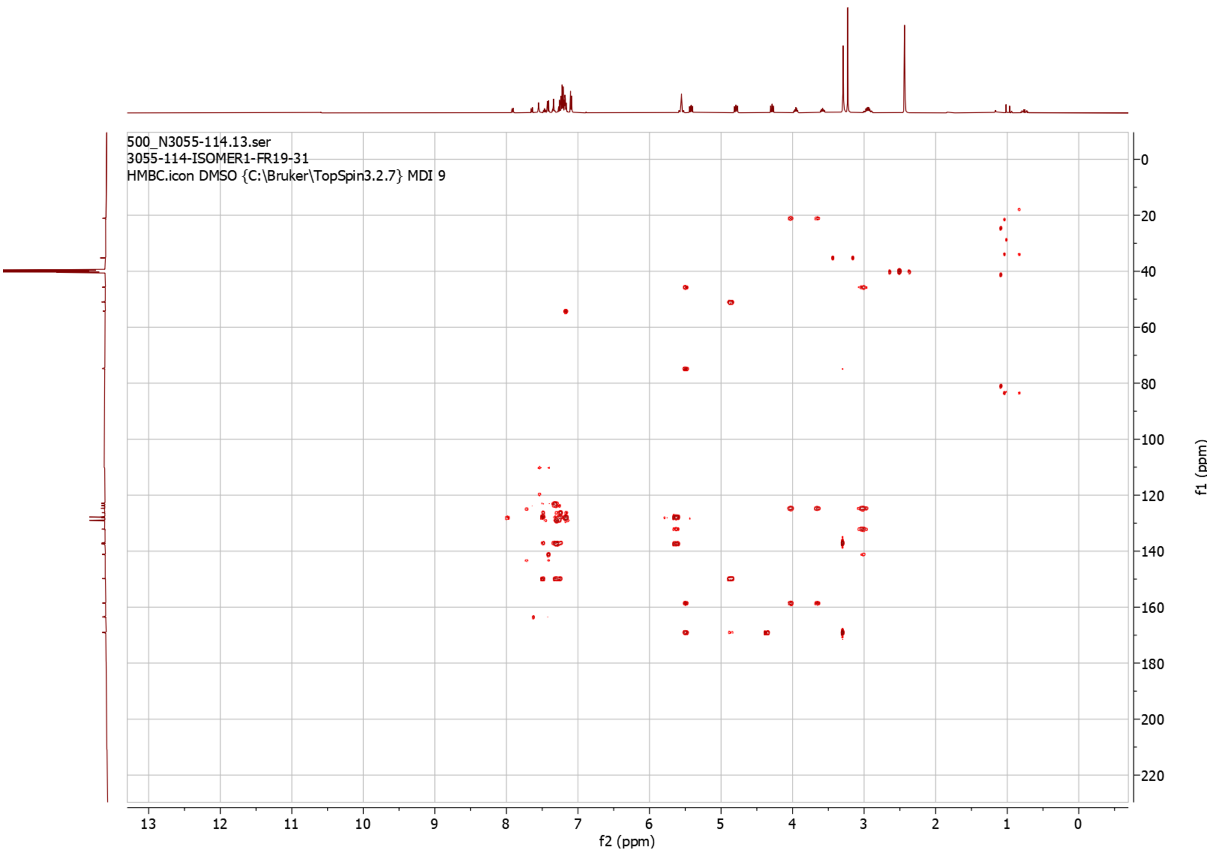

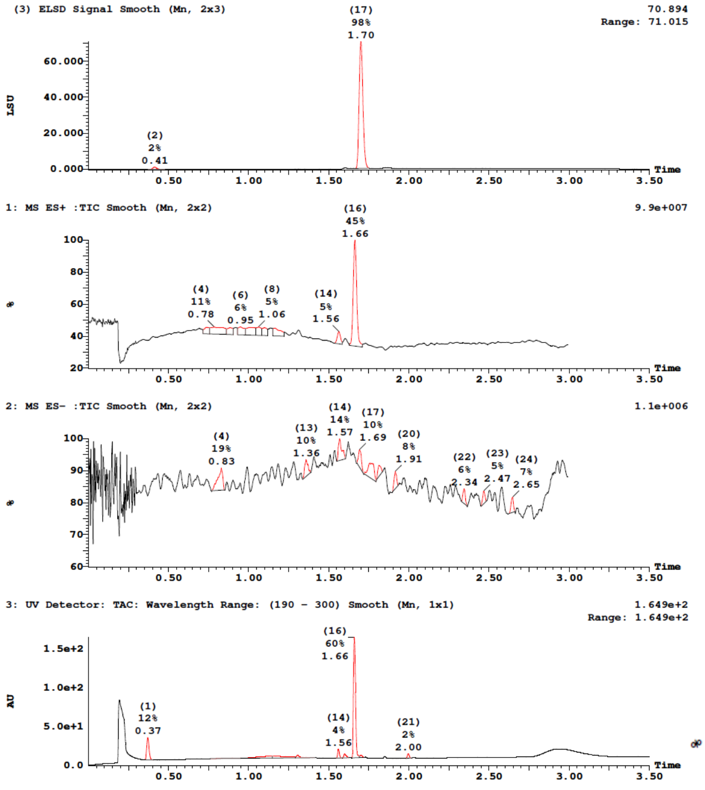

Peak ID	Compound	Time	Mass Found
16		1.66	Not Found

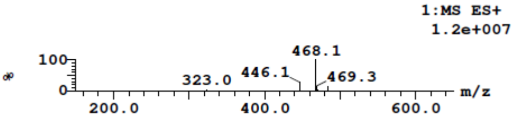

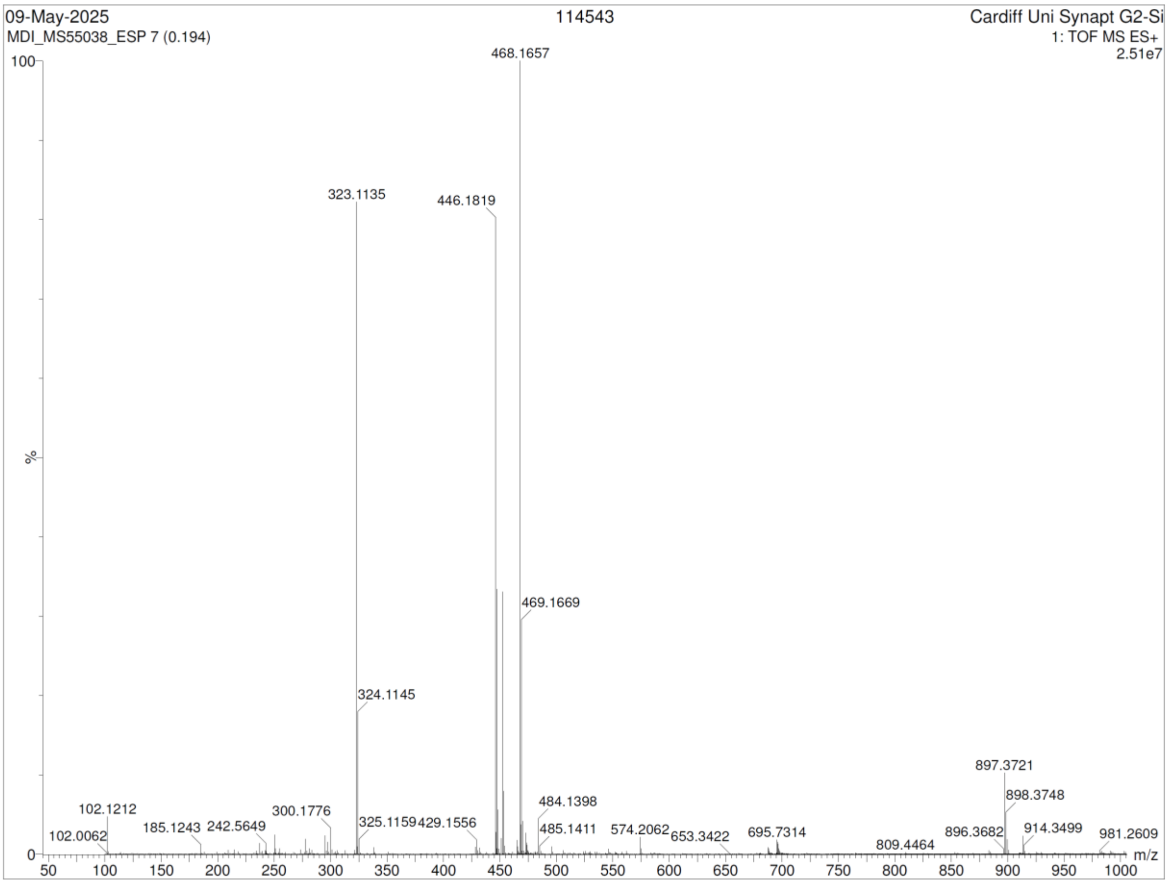

Elemental Composition Report

Single Mass Analysis

Tolerance = 10.0 PPM / DBE: min = -1.5, max = 50.0
Element prediction: Off
Number of isotope peaks used for i-FIT = 3

Monoisotopic Mass, Odd and Even Electron Ions
25 formula(e) evaluated with 1 results within limits (up to 50 closest results for each mass)
Elements Used:
C: 0-24 H: 0-24 N: 0-5 O: 0-4

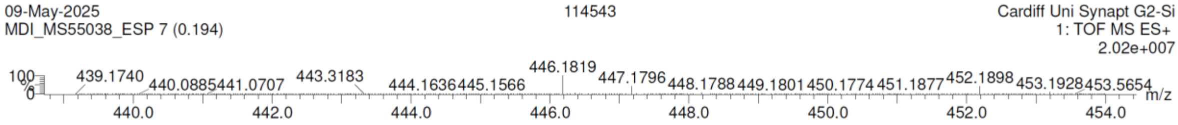

Mass	Calc. Mass	mDa	PPM	DBE	i-FIT	Norm	Conf(%)	Formula
Minimum:				-1.5				
Maximum:		5.0	10.0	50.0				
446.1819	446.1828	-0.9	-2.0	15.5	978.6	n/a	n/a	C24 H24 N5 O4

(*S*)-2-Benzyl-*N*-methyl-6-(5-methyl-4-oxo-2,3,4,5-tetrahydrobenzo[*b*][1,4]oxazepin-3-yl)-7-oxo-4,5,6,7-tetrahydro-2*H*-pyrazolo[3,4-*c*]pyridine-3-carboxamide (**23**)

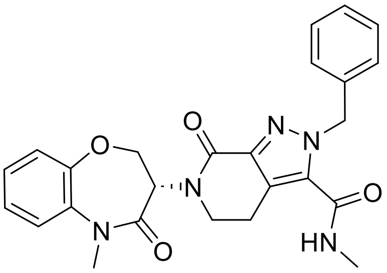

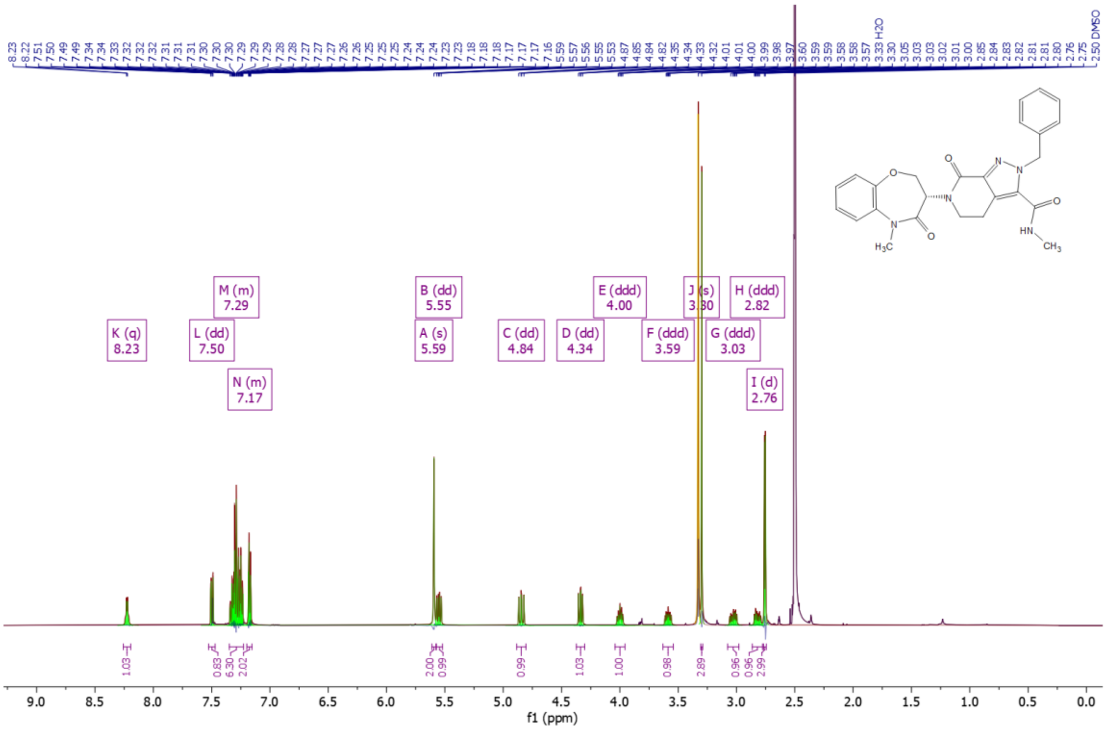

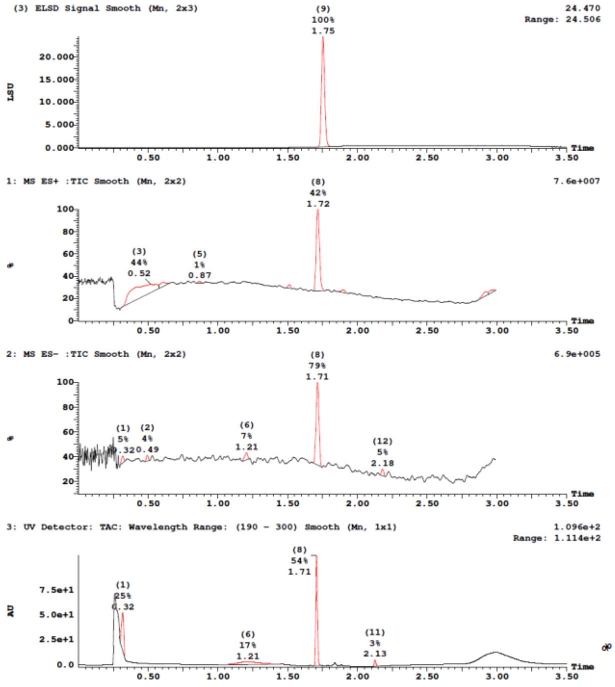

Peak ID	Compound	Time	Mass Found
8		1.72	Not Found

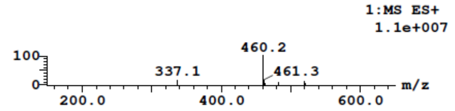

(*S*)-1-Benzyl-*N*-methyl-6-(5-methyl-4-oxo-2,3,4,5-tetrahydrobenzo[*b*][1,4]oxazepin-3-yl)-7-oxo-4,5,6,7-tetrahydro-1*H*-pyrazolo[3,4-*c*]pyridine-3-carboxamide (**24**)

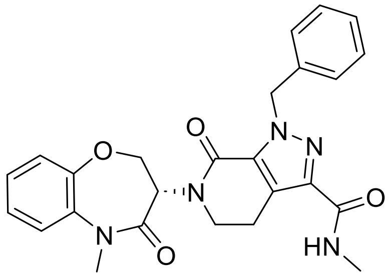

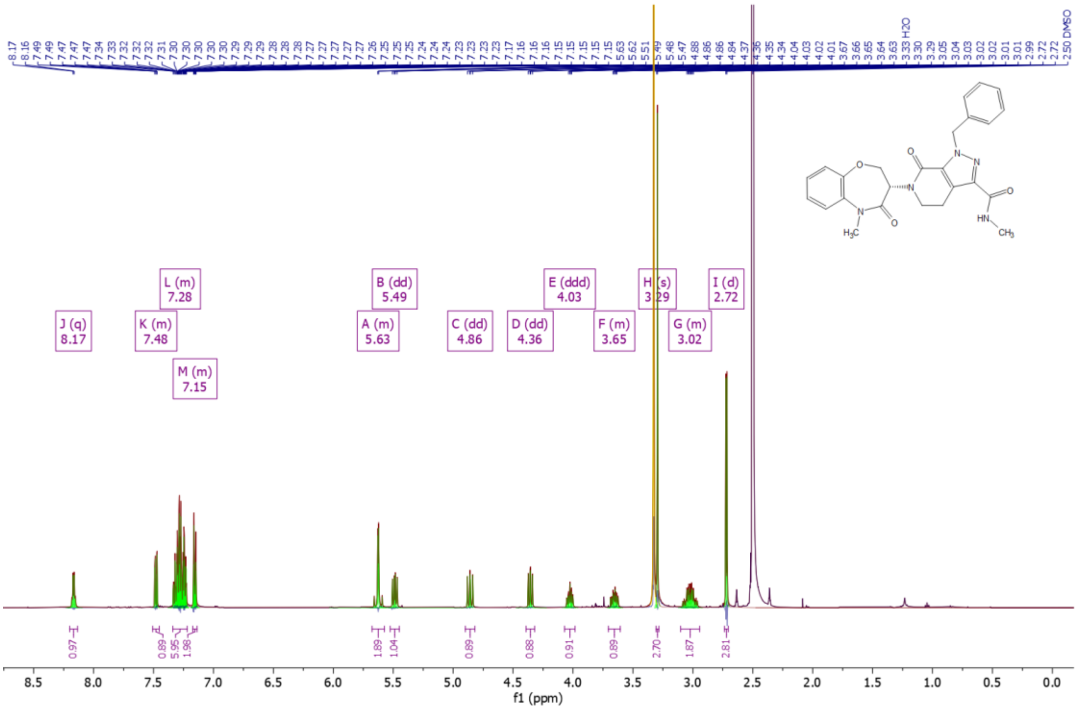

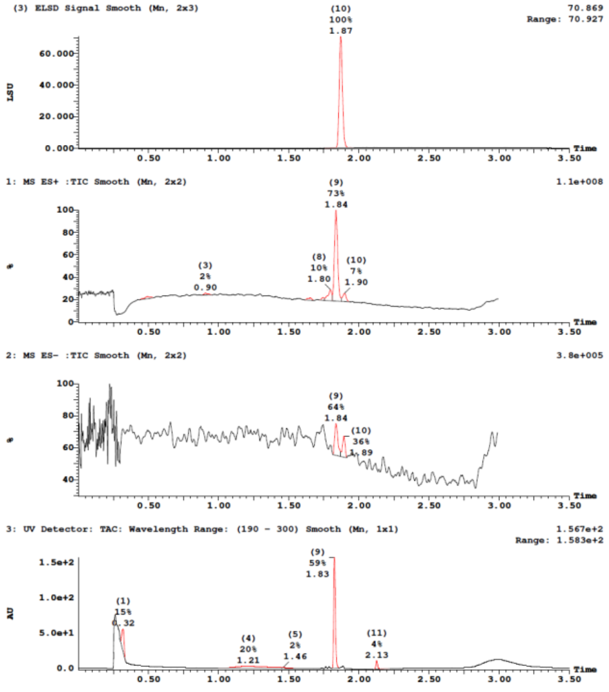

Peak ID	Compound	Time	Mass Found
9		1.84	Not Found

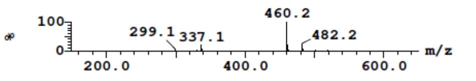

(*S*)-2-Benzyl-*N*,*N*-dimethyl-6-(5-methyl-4-oxo-2,3,4,5-tetrahydrobenzo[*b*][1,4]oxazepin-3-yl)-7-oxo-4,5,6,7-tetrahydro-2*H*-pyrazolo[3,4-*c*]pyridine-3-carboxamide (**25**)

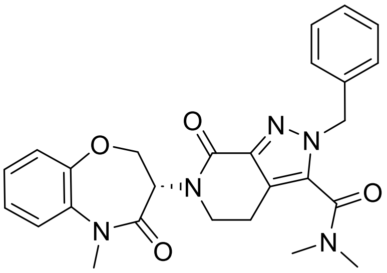

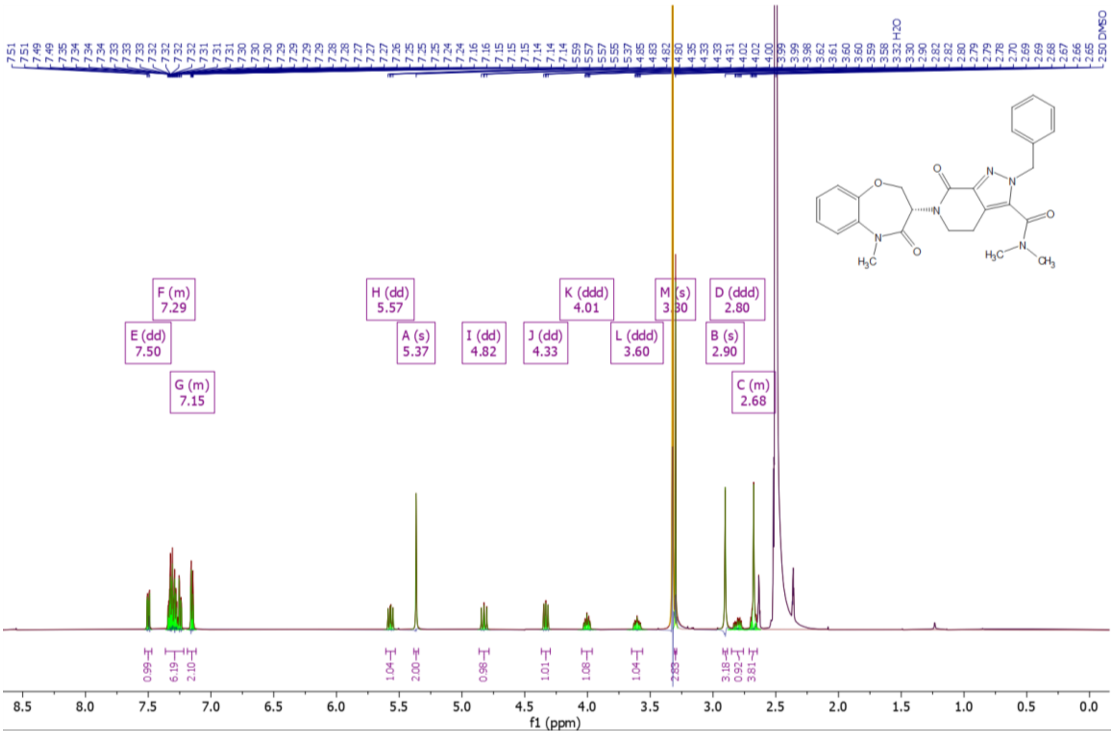

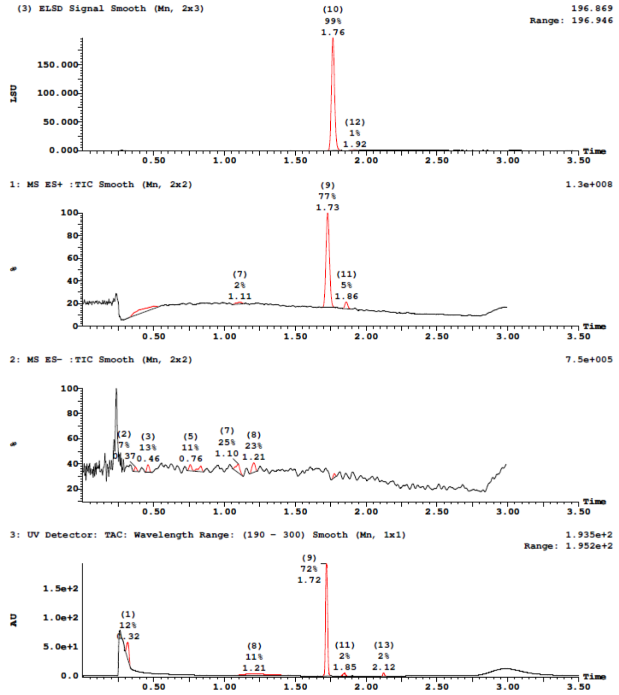

Peak ID	Compound	Time	Mass Found
9		1.73	Not Found

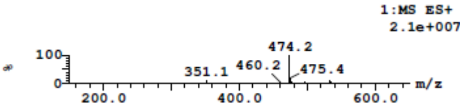

(*S*)-1-Benzyl-*N*,*N*-dimethyl-6-(5-methyl-4-oxo-2,3,4,5-tetrahydrobenzo[*b*][1,4]oxazepin-3-yl)-7-oxo-4,5,6,7-tetrahydro-1*H*-pyrazolo[3,4-*c*]pyridine-3-carboxamide (**26**)

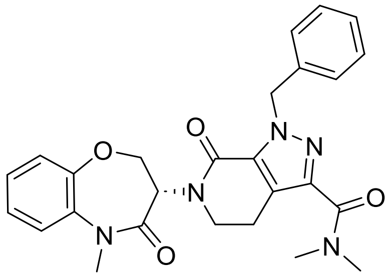

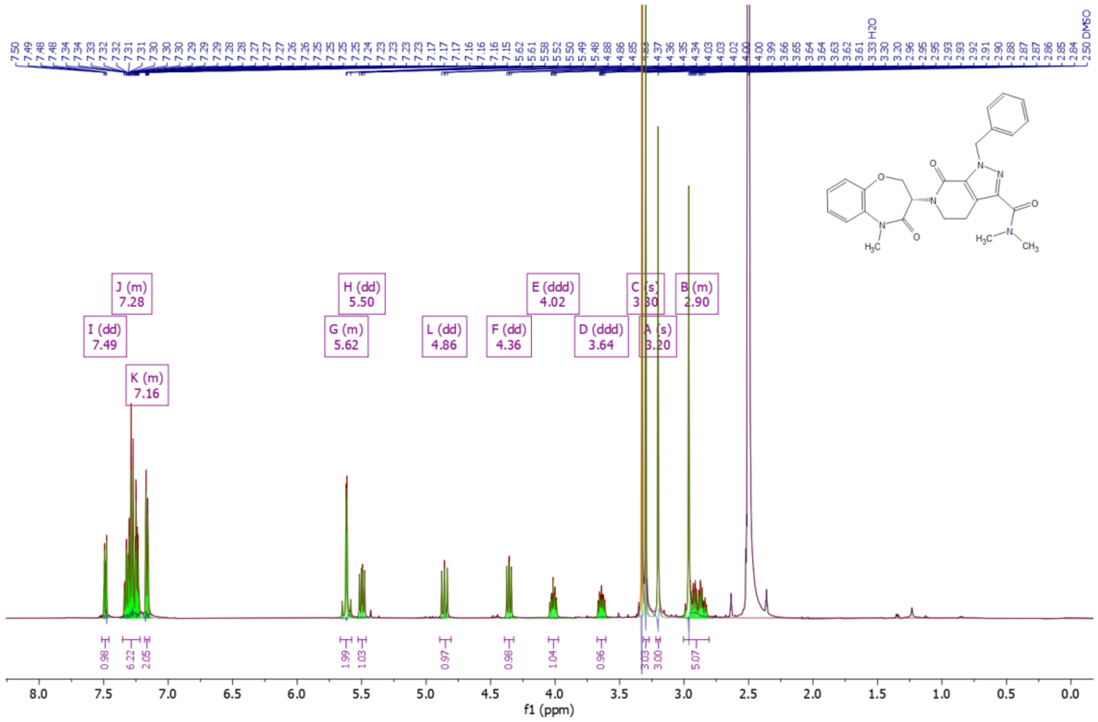

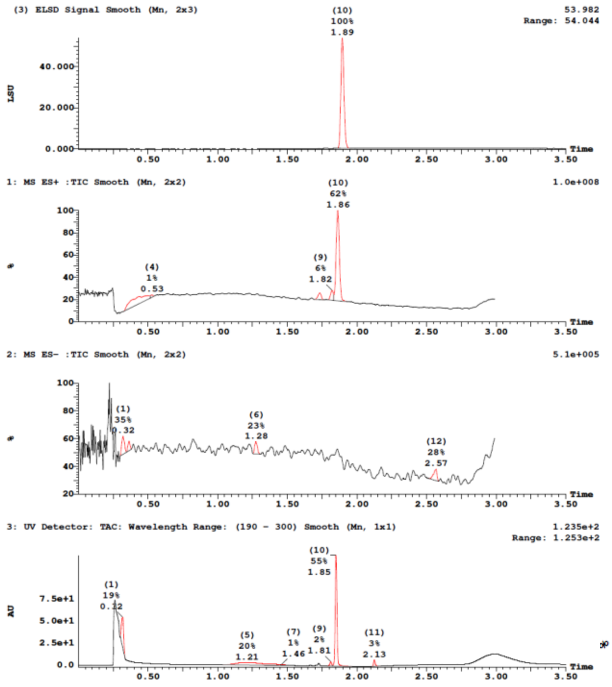

Peak ID	Compound	Time	Mass Found
10		1.86	Not Found

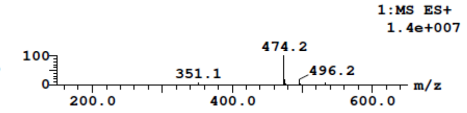

(*S*)-1-Benzyl-*N*[3]-(5-methyl-4-oxo-2,3,4,5-tetrahydrobenzo[*b*][1,4]oxazepin-3-yl)-1*H*-pyrazole-3,5-dicarboxamide (**29**)

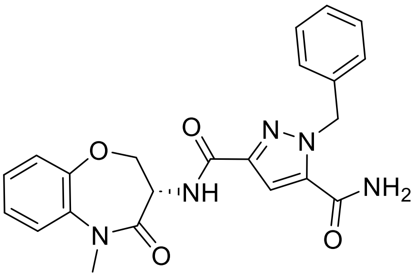

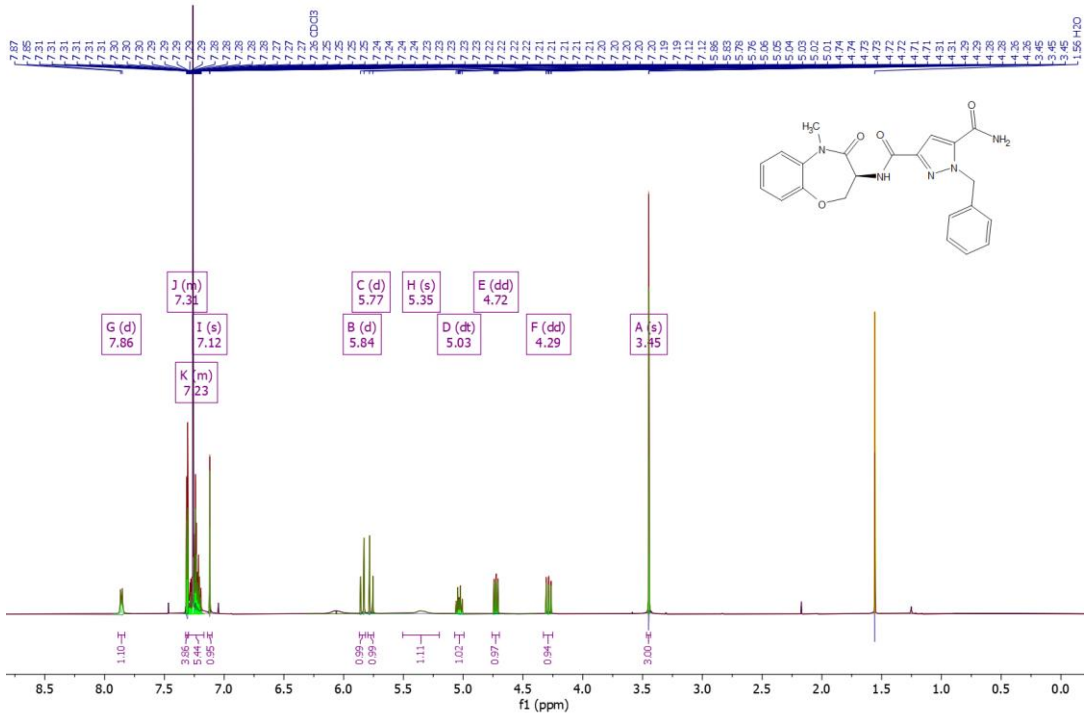

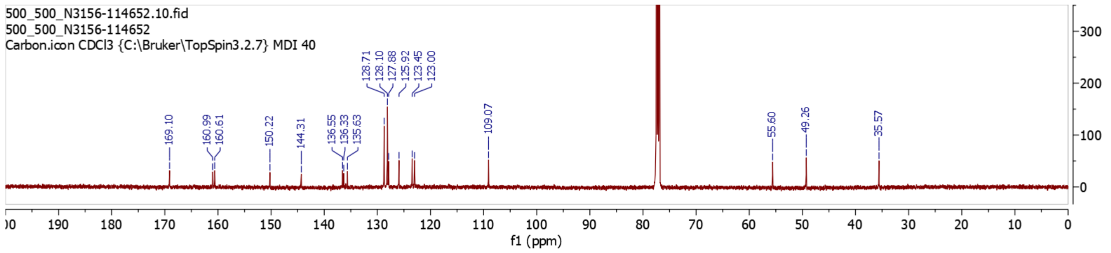

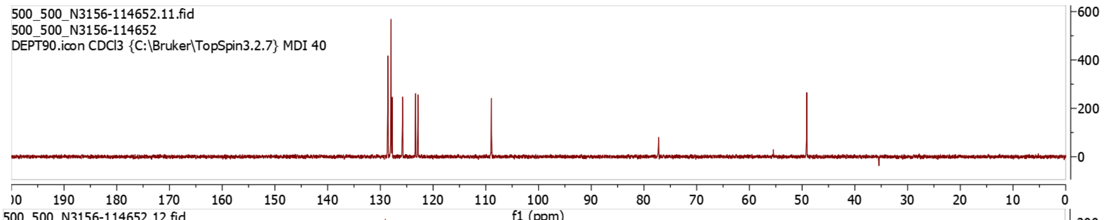

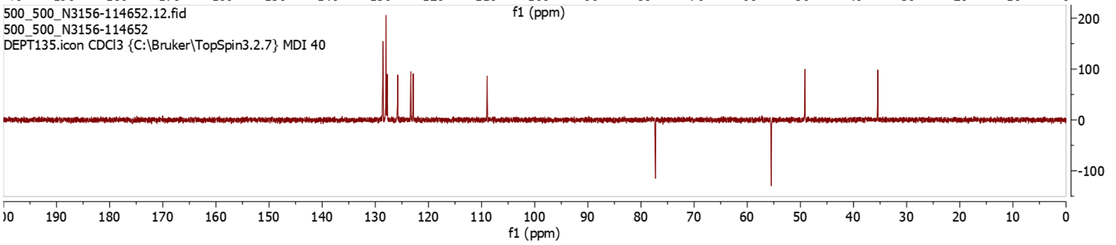

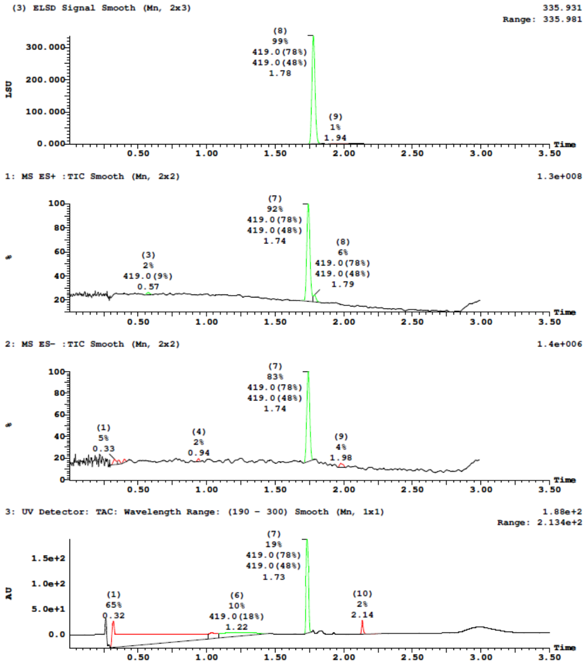

Peak ID	Compound	Time	Mass Found
7	Found	1.74	442,420

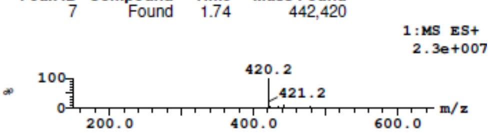

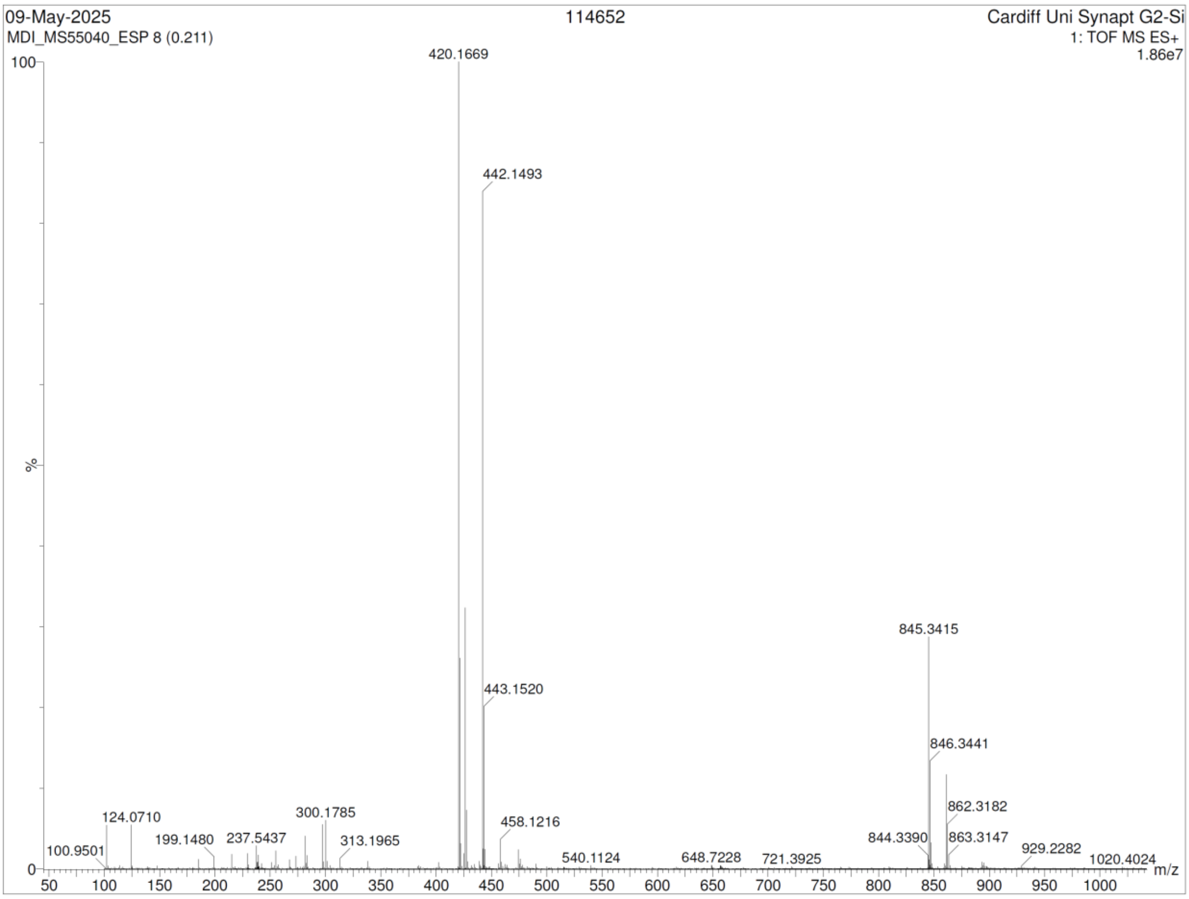

Elemental Composition Report

Single Mass Analysis

Tolerance = 10.0 PPM / DBE: min = -1.5, max = 50.0
Element prediction: Off
Number of isotope peaks used for i-FIT = 3

Monoisotopic Mass, Odd and Even Electron Ions
25 formula(e) evaluated with 1 results within limits (up to 50 closest results for each mass)
Elements Used:
C: 0-22 H: 0-22 N: 0-5 O: 0-4

09-May-2025 114652 Cardiff Uni Synapt G2-Si
MDI_MS55040_ESP 8 (0.211) 1: TOF MS ES+
1.86e+007

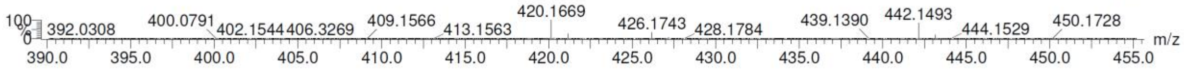

Mass	Calc. Mass	mDa	PPM	DBE	i-FIT	Norm	Conf(%)	Formula
Minimum:				-1.5				
Maximum:		5.0	10.0	50.0				
420.1669	420.1672	-0.3	-0.7	14.5	1009.8	n/a	n/a	C22 H22 N5 O4

1-Benzyl-3-(2-(benzyl(cyclopropylmethyl)amino)-2-oxoethyl)-1H-pyrazole-5-carboxamide (**31**)

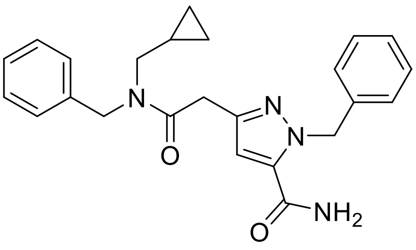

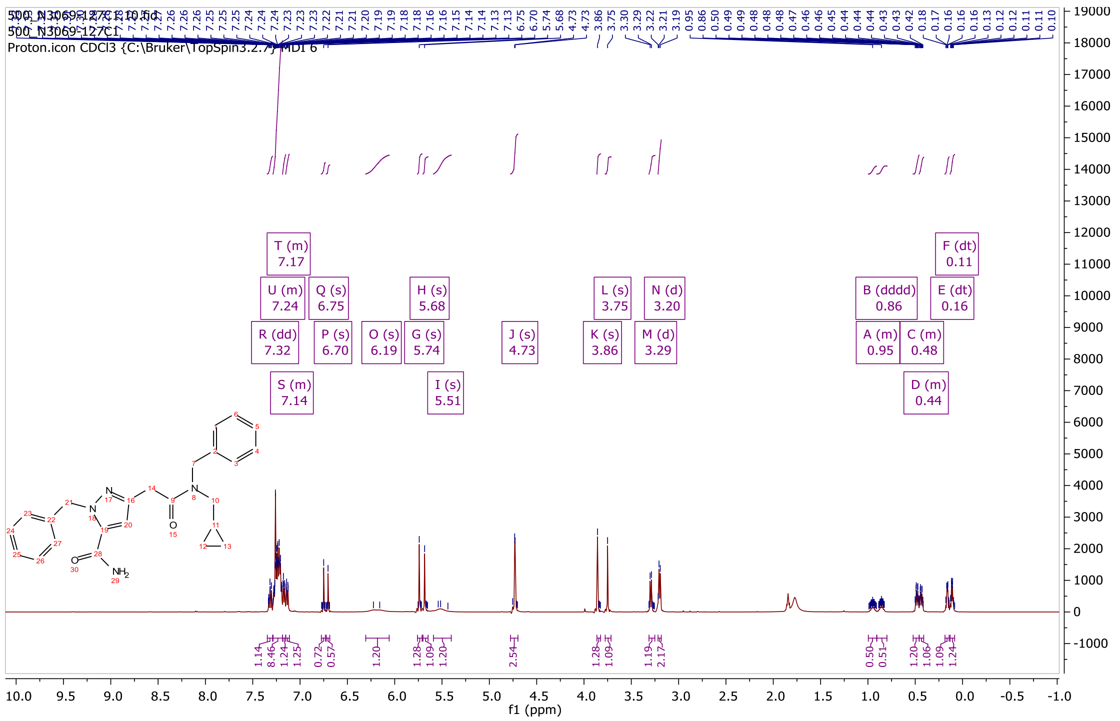

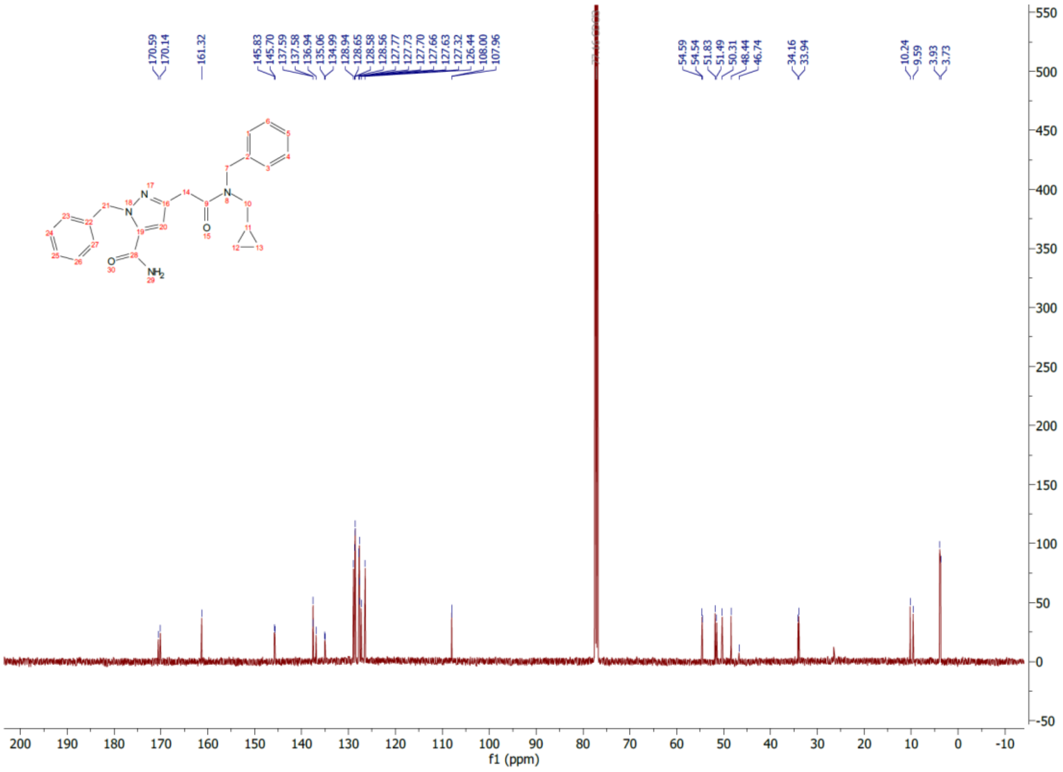

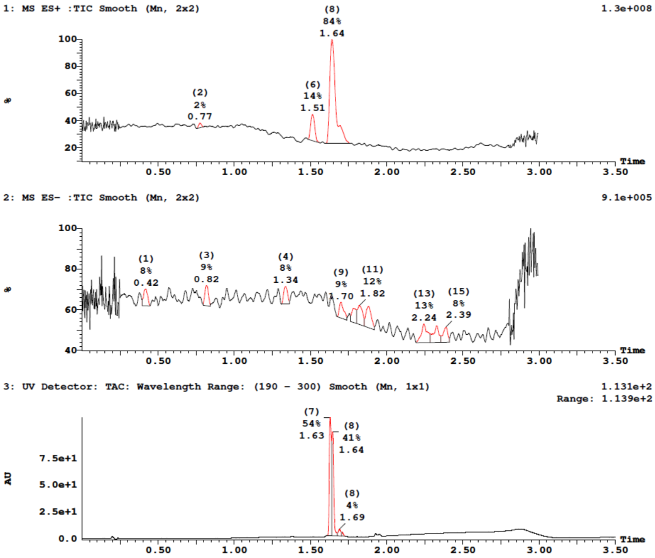

Peak ID	Compound	Time	Mass Found
8		1.64	Not Found

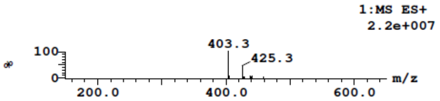

20-Feb-2025

N3069128

XEVO-G2XSQTOF#NotSet
Cardiff University
1: TOF MS ES+
1.53e7

MDI_MS54194_ESP 5 (0.209)

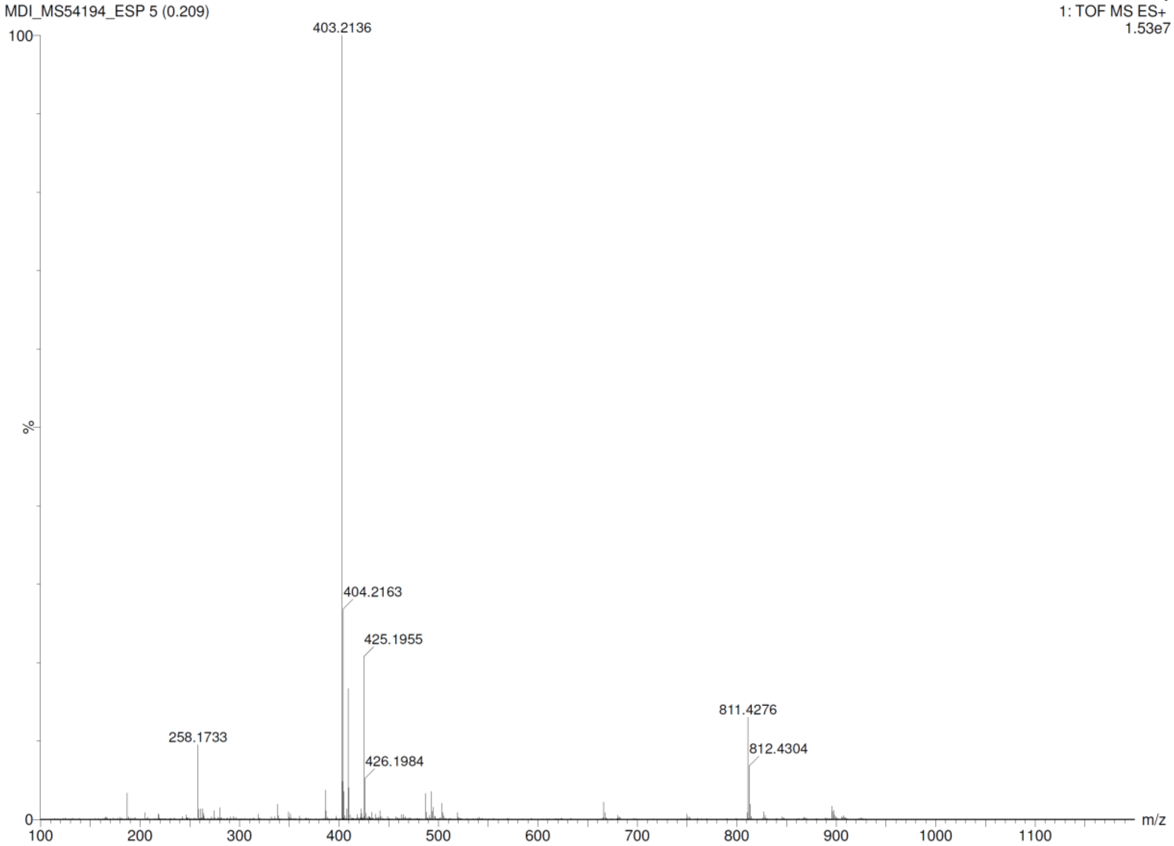

Elemental Composition Report

Single Mass Analysis

Tolerance = 5.0 PPM / DBE: min = -1.5, max = 50.0
Element prediction: Off
Number of isotope peaks used for i-FIT = 5

Monoisotopic Mass, Odd and Even Electron Ions
10 formula(e) evaluated with 1 results within limits (up to 1 best isotopic matches for each mass)
Elements Used:
C: 0-24 H: 0-27 N: 0-4 O: 0-2

Minimum:				-1.5				
Maximum:		5.0	5.0	50.0				
Mass	Calc. Mass	mDa	PPM	DBE	i-FIT	Norm	Conf(%)	Formula
403.2136	403.2134	0.2	0.5	13.5	2029.3	n/a	n/a	C24 H27 N4 O2

N^3,1-Dibenzyl-N^3-(cyclopropylmethyl)-1*H*-pyrazole-3,5-dicarboxamide (**32**)

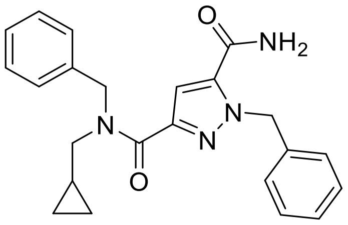

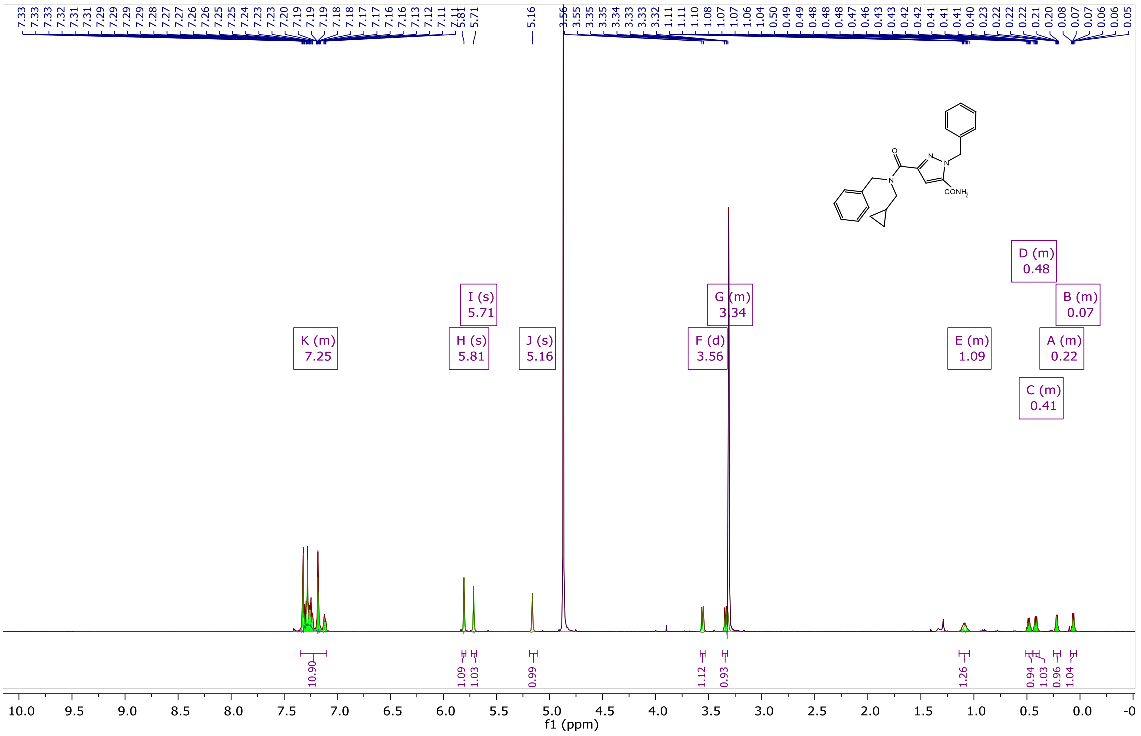

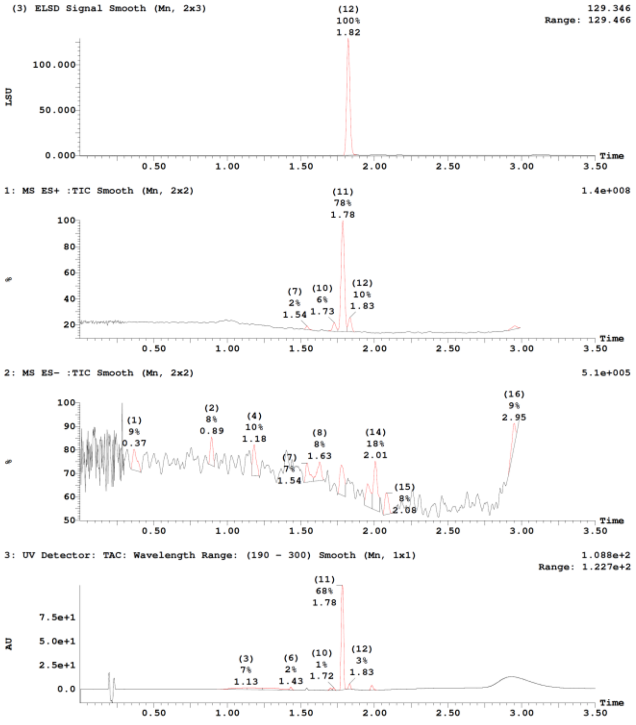

Peak ID	Compound	Time	Mass Found
11		1.78	Not Found

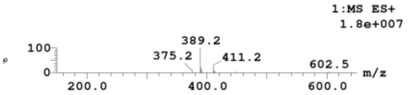

(*S*)-*N*-(8-Chloro-5-methyl-4-oxo-2,3,4,5-tetrahydrobenzo[*b*][1,4]oxazepin-3-yl)-4-(*N*-phenylsulfamoyl)benzamide (**33**)

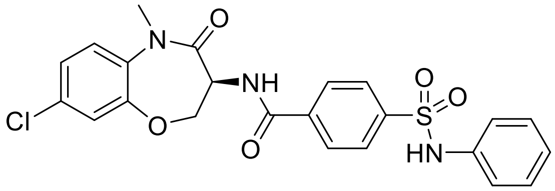

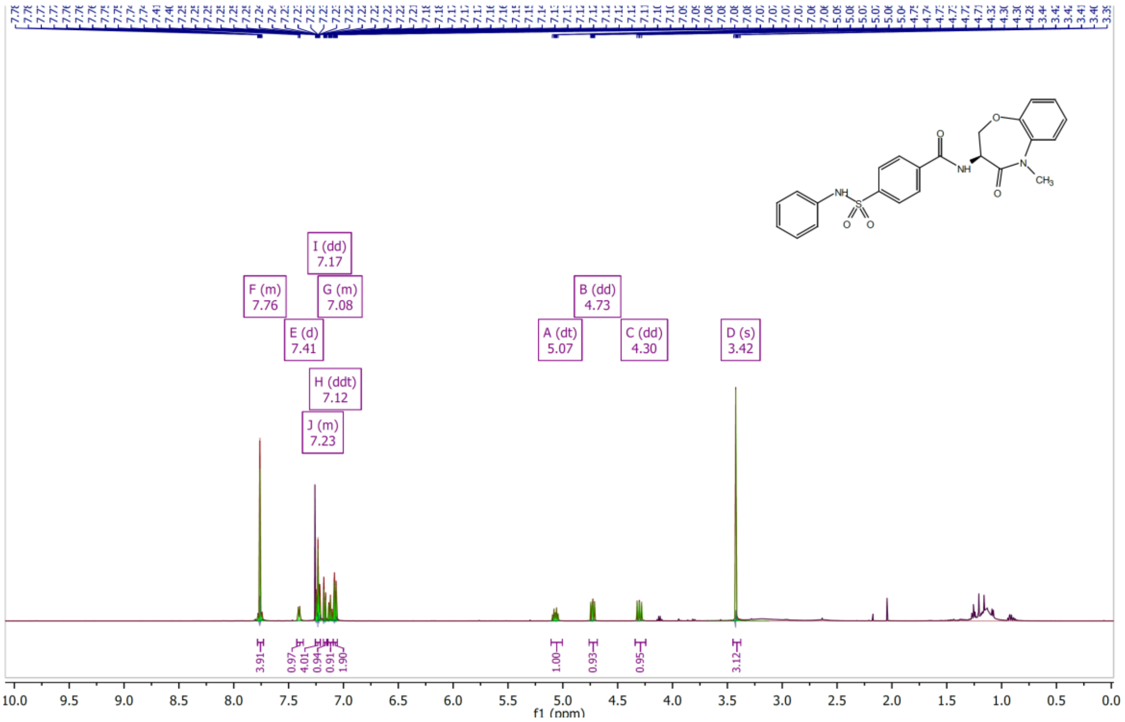

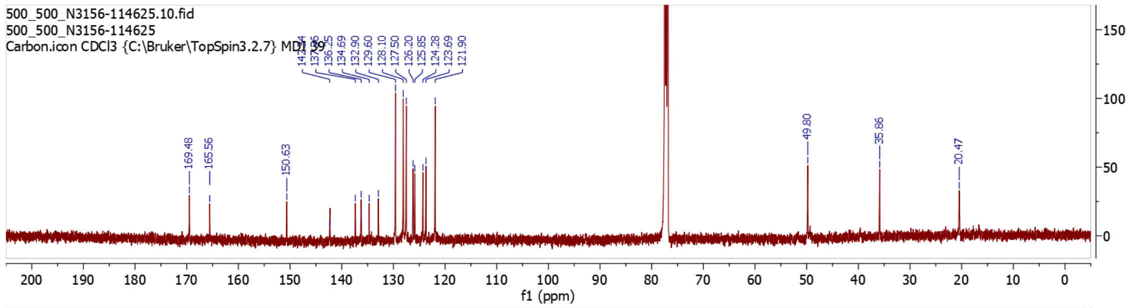

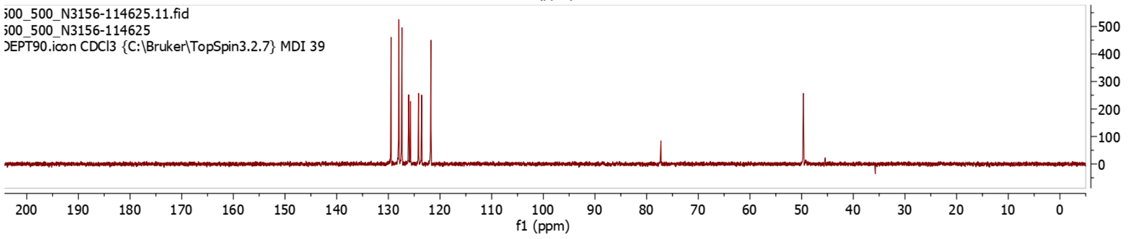

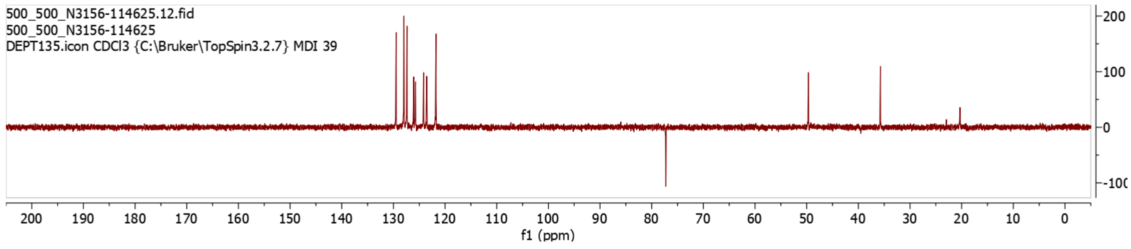

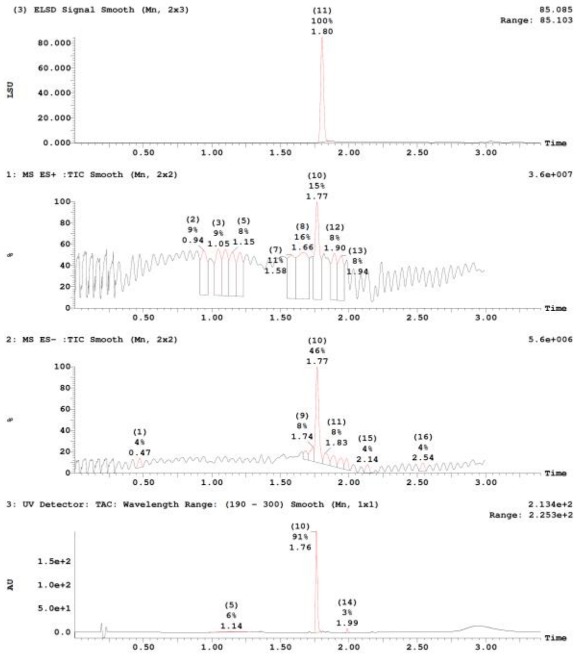

Peak ID	Compound	Time	Mass Found
11		1.83	Not Found

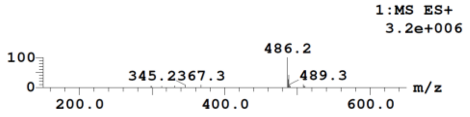

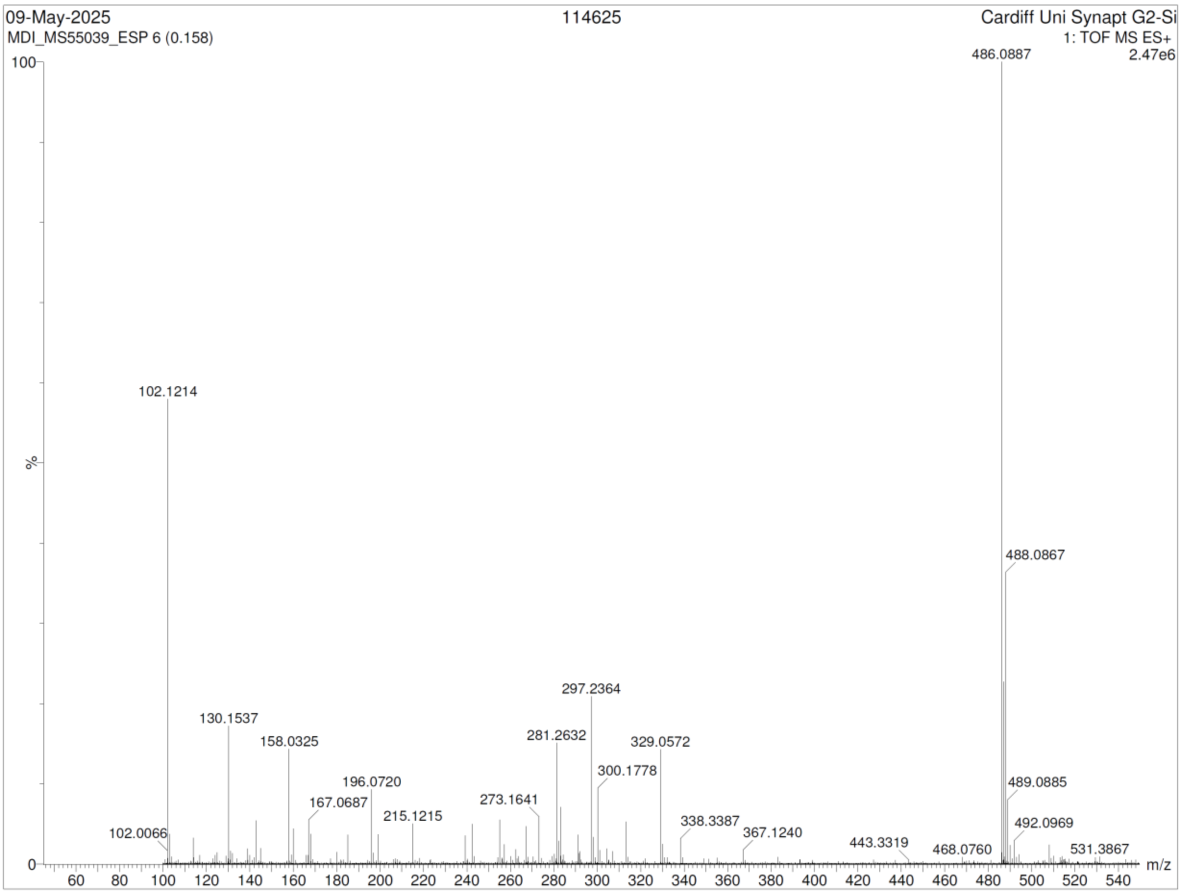

Elemental Composition Report **Page 1**

Single Mass Analysis

Tolerance = 10.0 PPM / DBE: min = -1.5, max = 50.0
Element prediction: Off
Number of isotope peaks used for i-FIT = 3

Monoisotopic Mass, Odd and Even Electron Ions
89 formula(e) evaluated with 1 results within limits (up to 50 closest results for each mass)
Elements Used:
C: 0-23 H: 0-21 N: 0-3 O: 0-5 S: 0-1 Cl: 0-1

09-May-2025 114625 Cardiff Uni Synapt G2-Si
MDI_MS55039_ESP 6 (0.158) 1: TOF MS ES+
2.47e+006

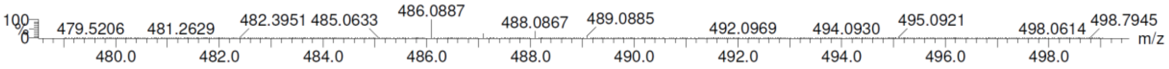

Minimum:				-1.5				
Maximum:		5.0	10.0	50.0				
Mass	Calc. Mass	mDa	PPM	DBE	i-FIT	Norm	Conf(%)	Formula
486.0887	486.0890	-0.3	-0.6	14.5	917.3	n/a	n/a	C23 H21 N3 O5 S Cl

2,6-Dibenzyl-7-oxo-4,5,6,7-tetrahydro-2*H*-pyrazolo[3,4-*c*]pyridine-3-carboxamide (**34**)

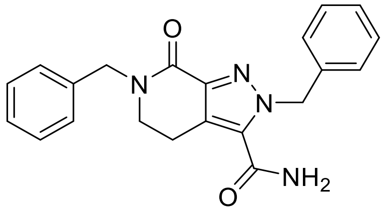

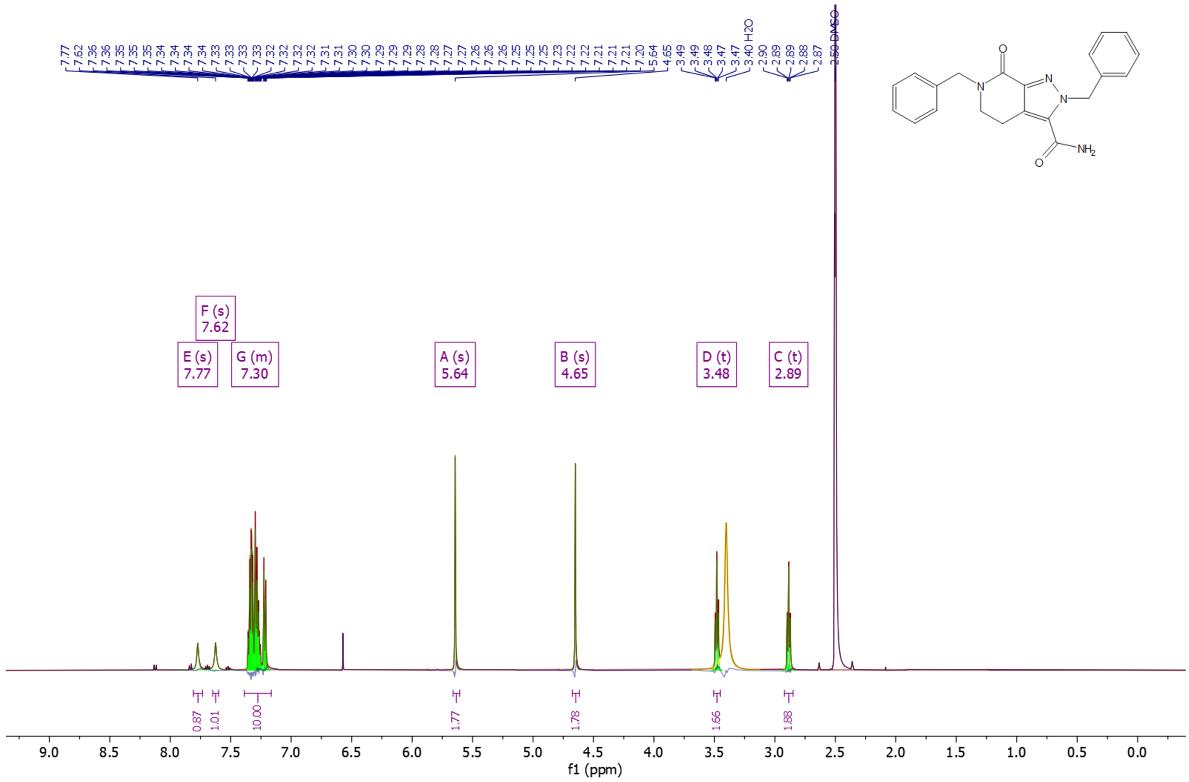

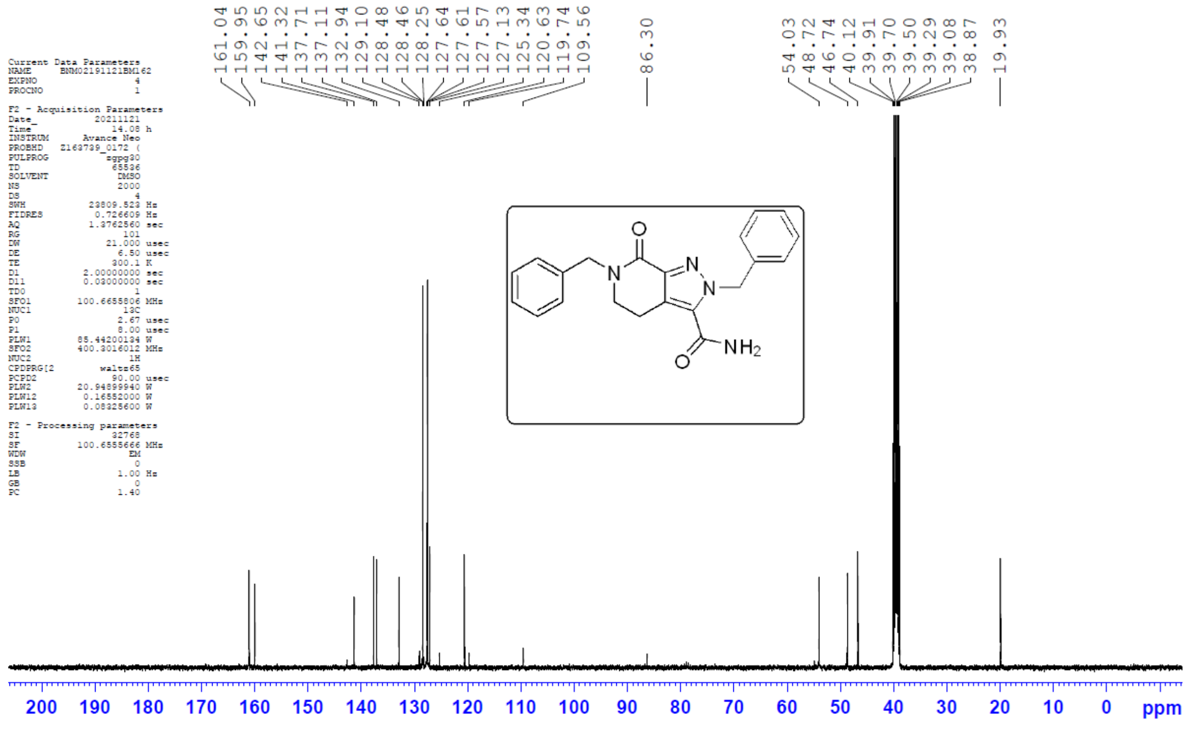

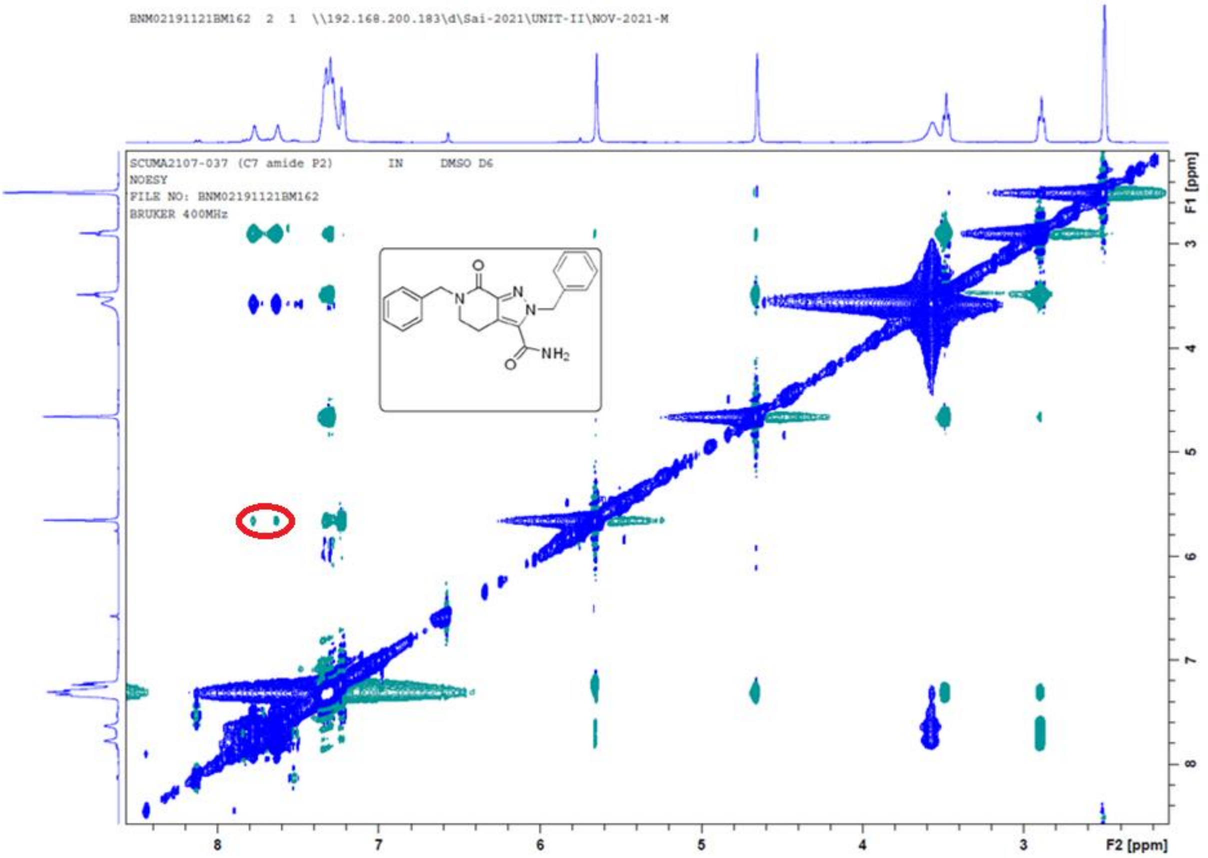
BNM02191121BM162 2 1 \\192.168.200.183\d\Sai-2021\UNIT-II\NOV-2021-M
SCUMA2107-037 (C7 amide P2) IN DMSO D6
NOESY
FILE NO: BNM02191121BM162
BRUKER 400MHz
F2 [ppm]
F1 [ppm]
NH_2

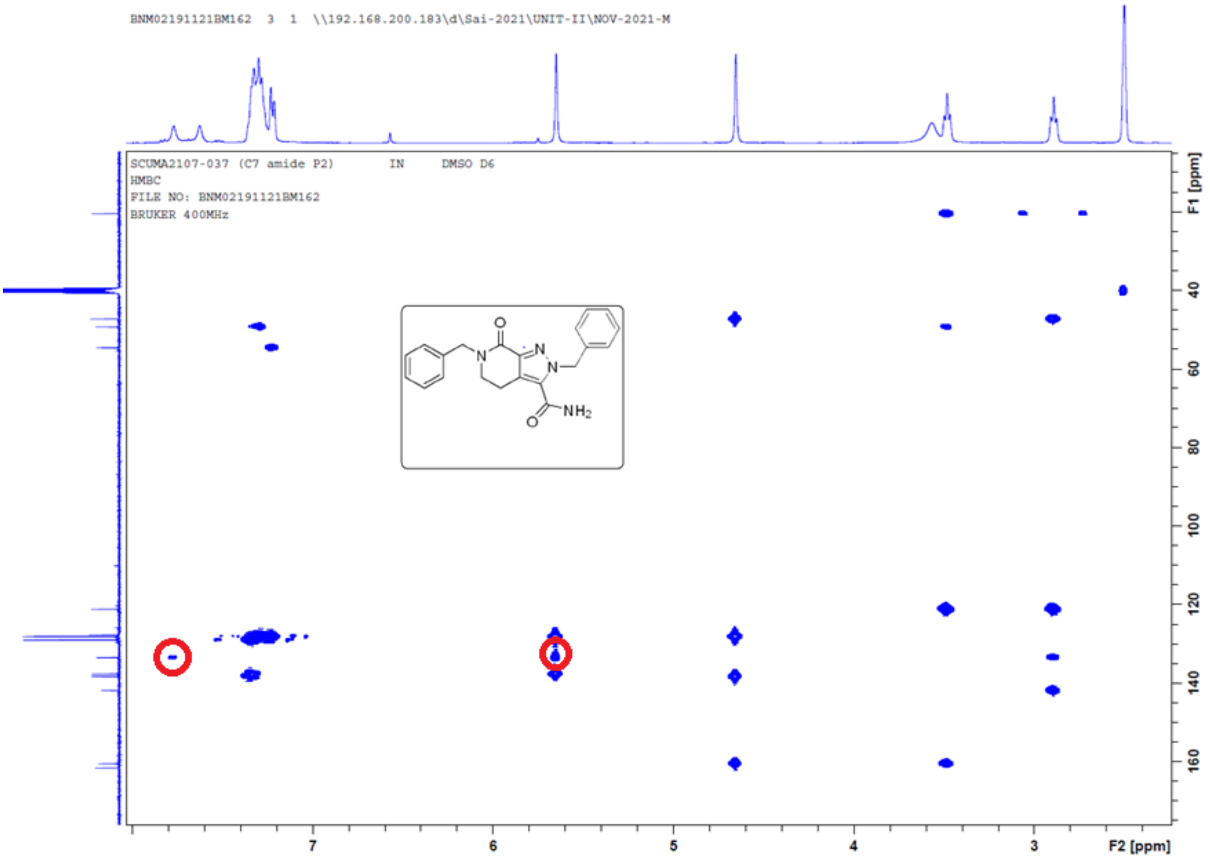
BNM02191121BM162 3 1 \\192.168.200.183\d\Sai-2021\UNIT-II\NOV-2021-M
SCUMA2107-037 (C7 amide P2) IN DMSO D6
HMBC
FILE NO: BNM02191121BM162
BRUKER 400MHz
F2 [ppm]
F1 [ppm]
NH_2

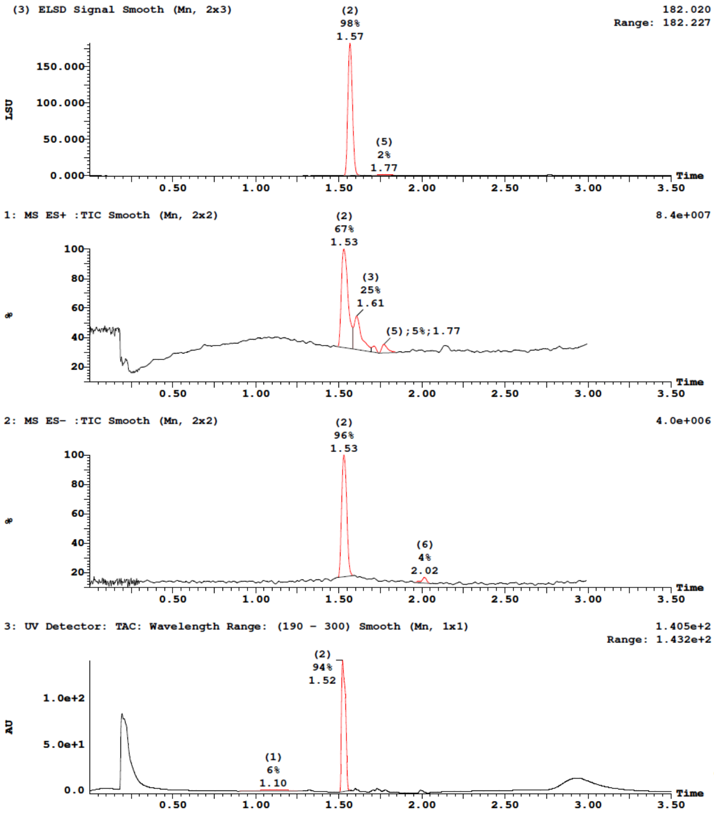

Peak ID	Compound	Time	Mass Found
2		1.53	Not Found

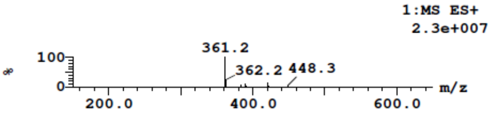

Methyl 2,6-dibenzyl-7-oxo-4,5,6,7-tetrahydro-2*H*-pyrazolo[3,4-*c*]pyridine-3-carboxylate (**55**)

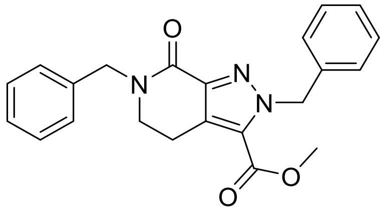

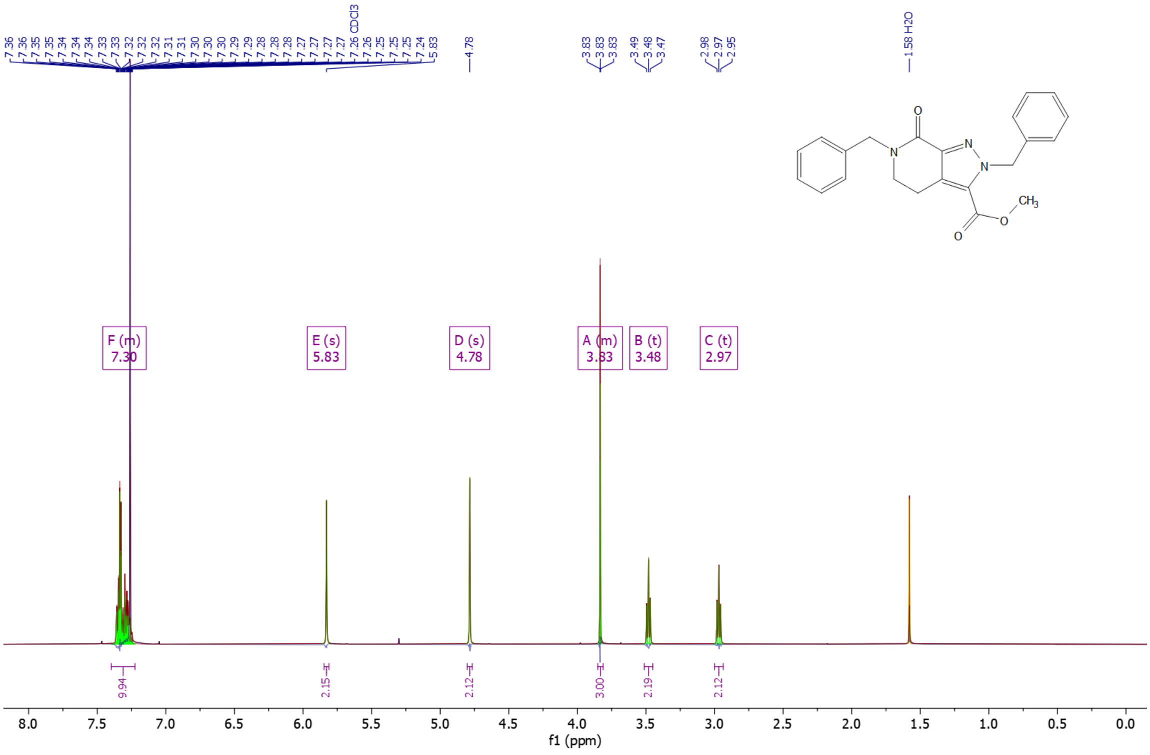

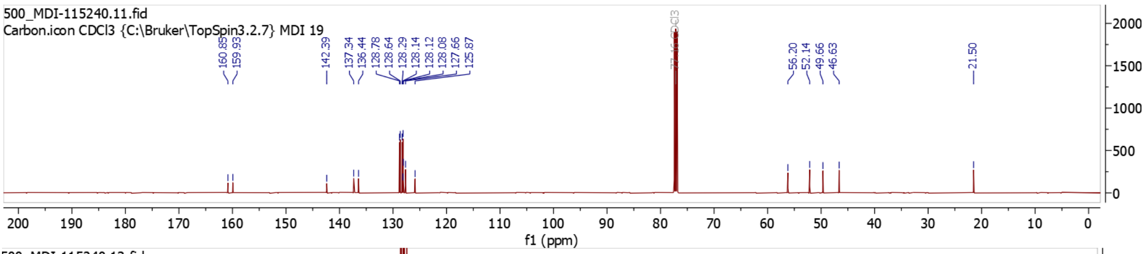

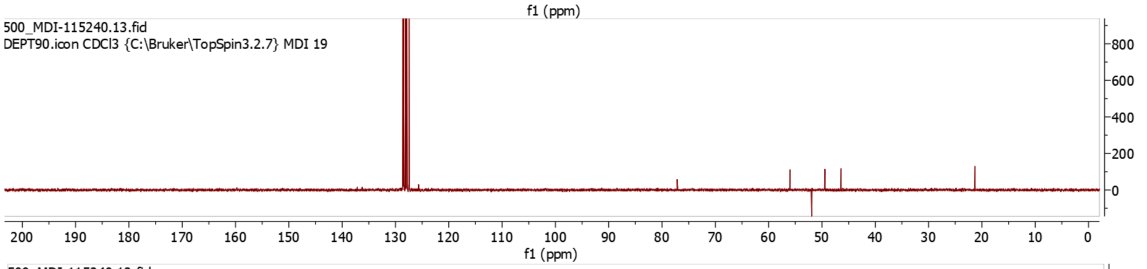

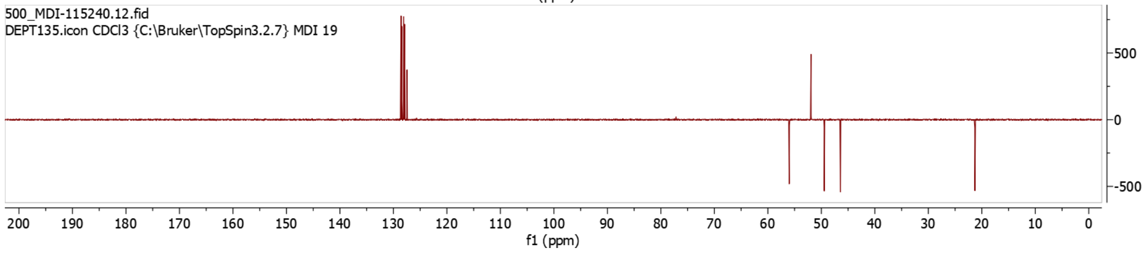

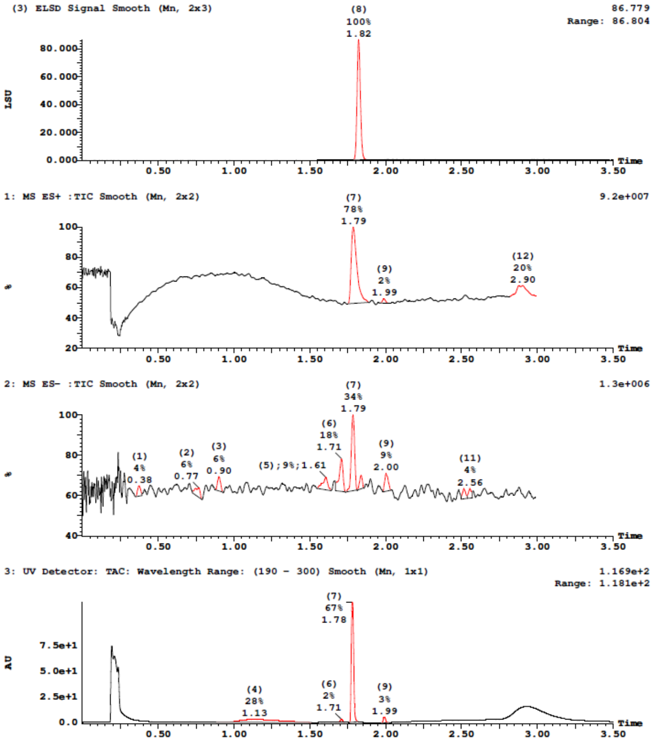

Peak ID	Compound	Time	Mass Found
7		1.79	Not Found

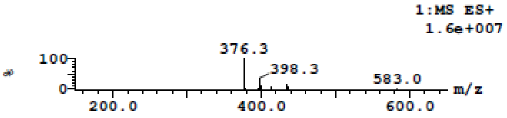

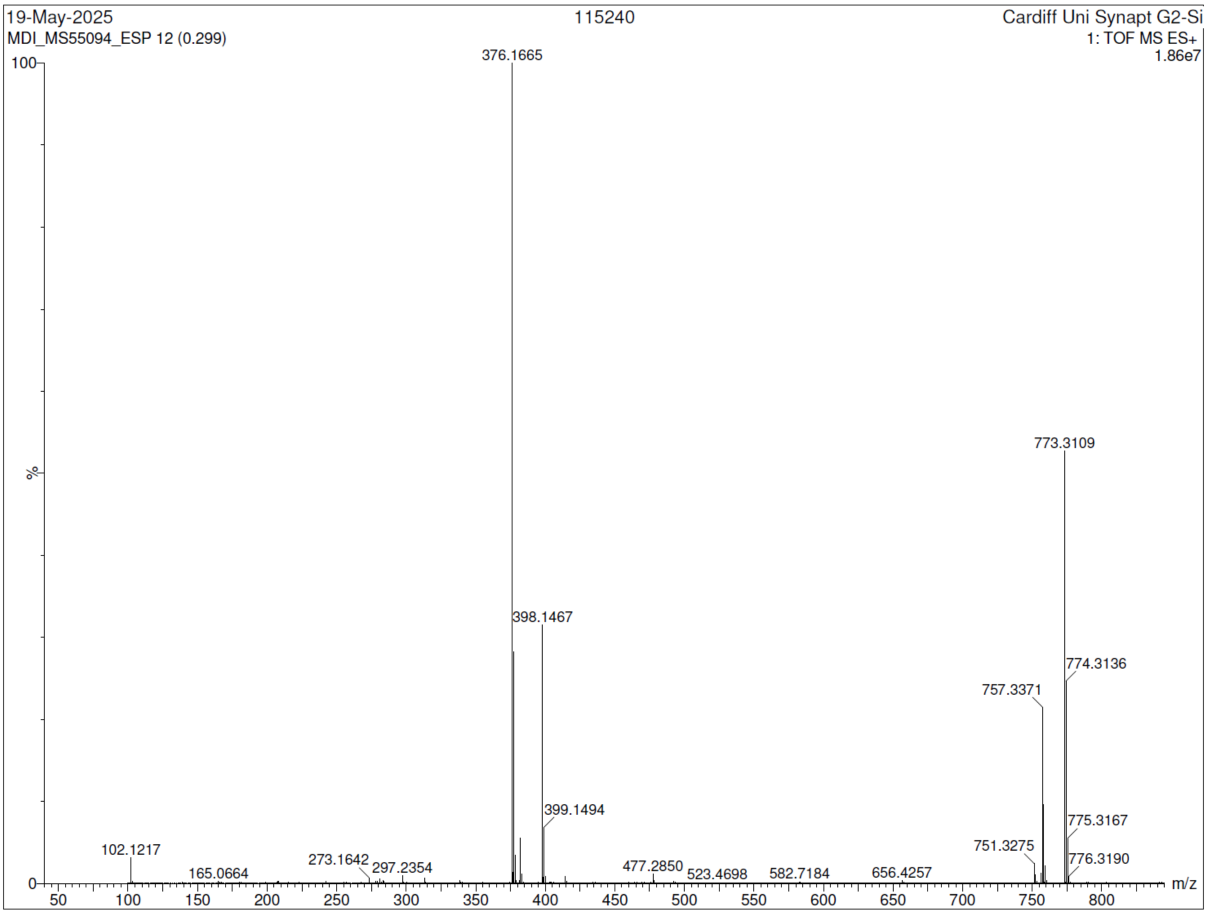

Elemental Composition Report

Single Mass Analysis

Tolerance = 50.0 PPM / DBE: min = -1.5, max = 50.0
Element prediction: Off
Number of isotope peaks used for i-FIT = 3

Monoisotopic Mass, Odd and Even Electron Ions
11 formula(e) evaluated with 1 results within limits (up to 50 closest results for each mass)
Elements Used:
C: 0-22 H: 0-22 N: 0-3 O: 0-3

19-May-2025 115240 Cardiff Uni Synapt G2-Si
MDI_MS55094_ESP 12 (0.299) 1: TOF MS ES+
1.86e+007

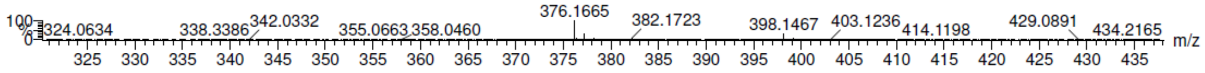

Minimum:				-1.5				
Maximum:		5.0	50.0	50.0				
Mass	Calc. Mass	mDa	PPM	DBE	i-FIT	Norm	Conf(%)	Formula
376.1665	376.1661	0.4	1.1	13.5	1207.7	n/a	n/a	C22 H22 N3 O3

1,6-Dibenzyl-7-oxo-4,5,6,7-tetrahydro-1*H*-pyrazolo[3,4-*c*]pyridine-3-carboxamide (**57**)

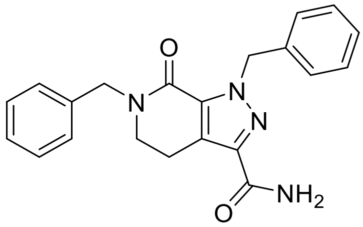

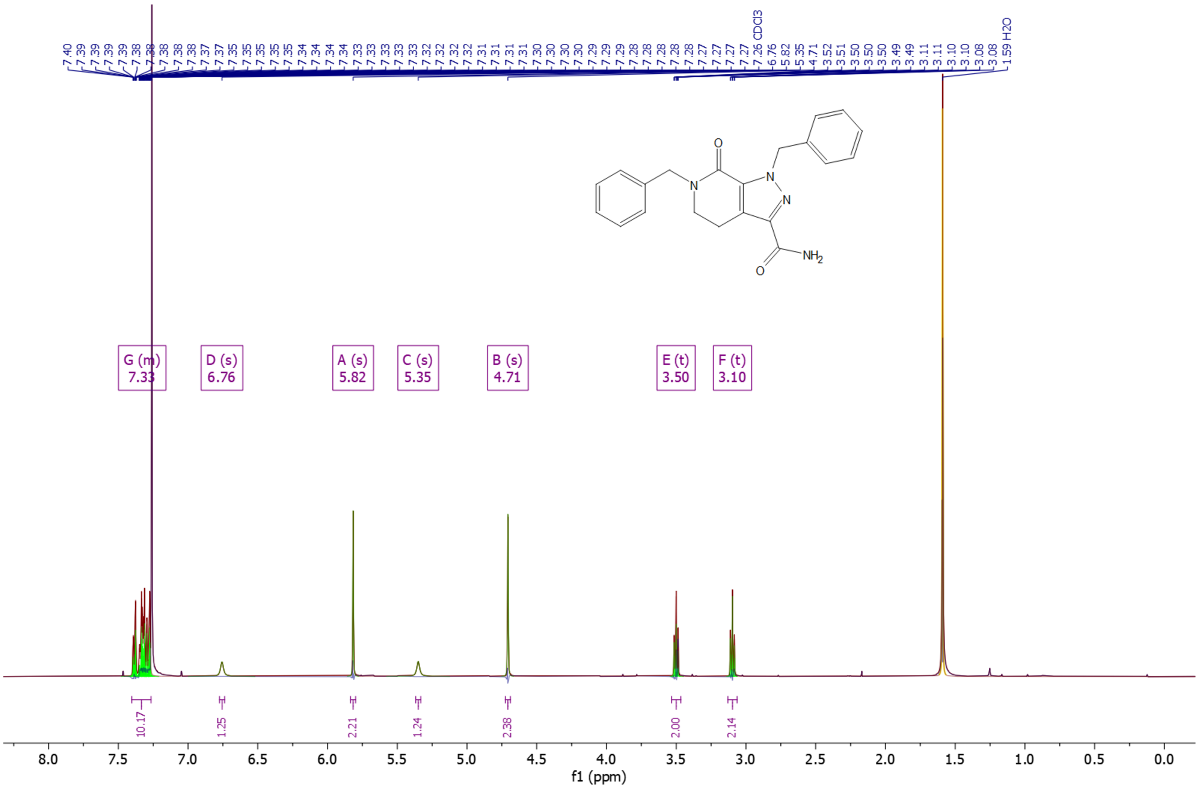

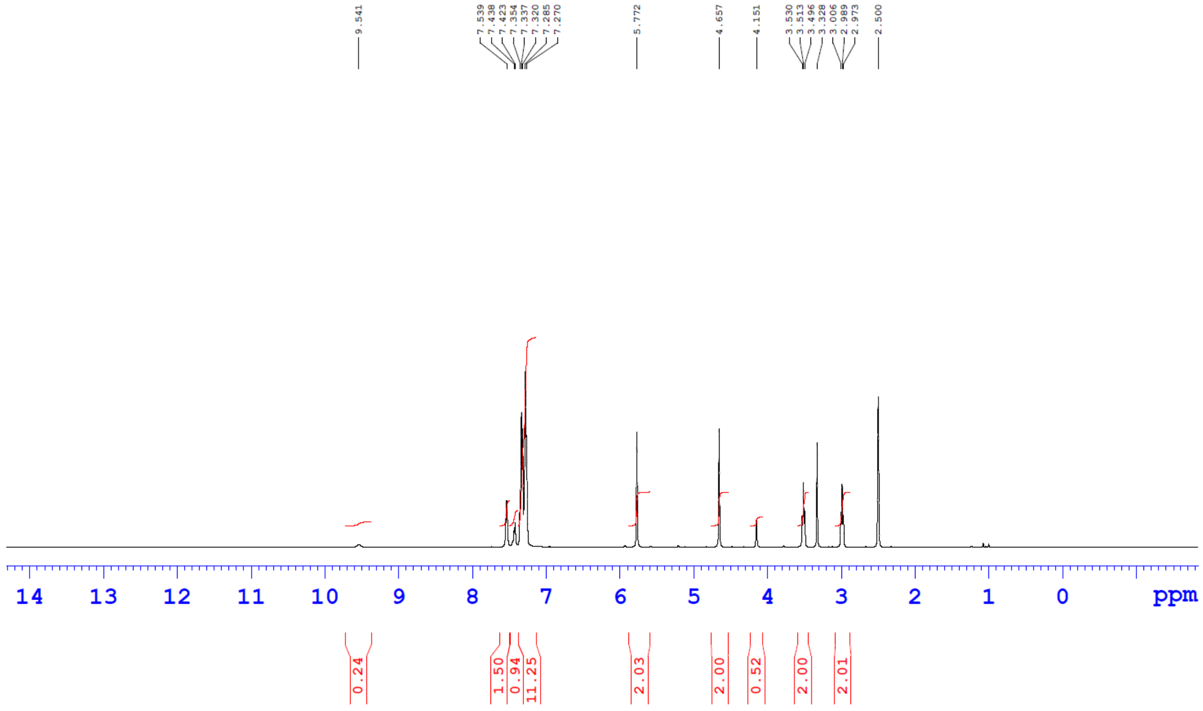

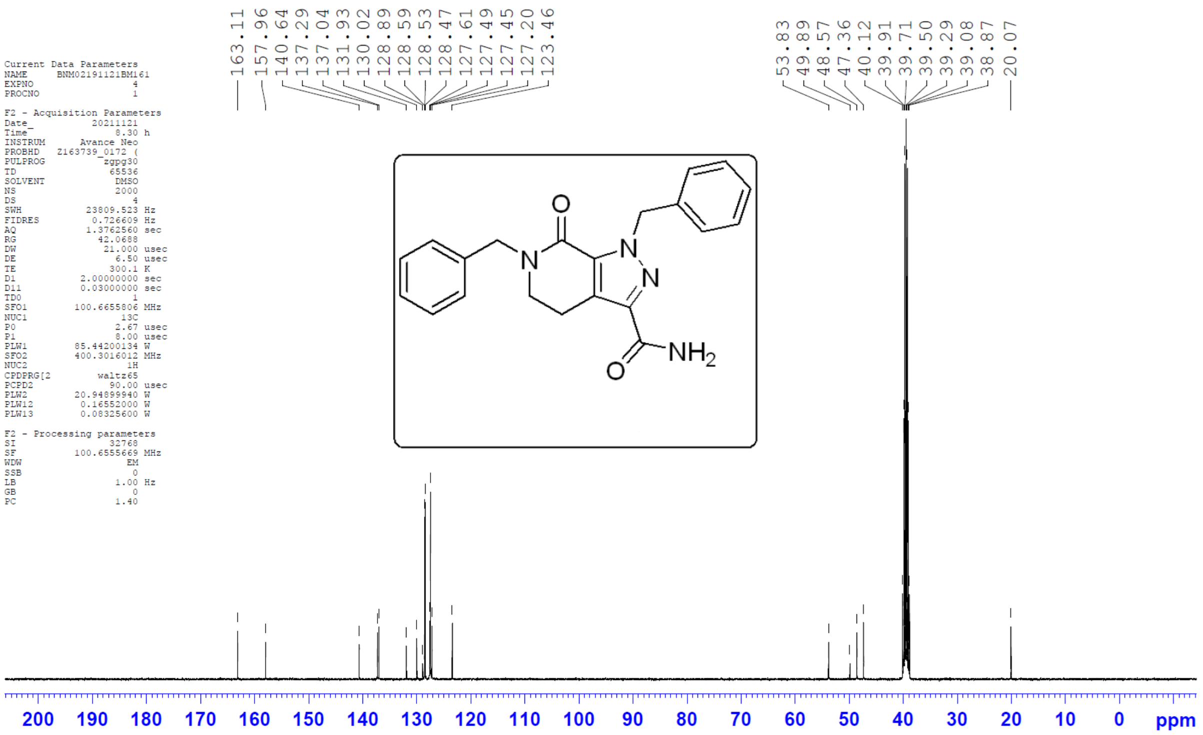
163.11
157.96
140.64
137.29
137.04
131.93
130.02
128.89
128.59
128.53
128.47
127.61
127.49
127.45
127.20
123.46
53.83
49.89
48.57
47.36
40.12
39.91
39.71
39.50
39.29
39.08
38.87
20.07
Current Data Parameters
NAME BNM02191121BM161
EXPNO 4
PROCNO 1
F2 - Acquisition Parameters
Date_ 20211121
Time 8.30 h
INSTRUM Avance Neo
PROBHD Z163739_0172 (
PULPROG zgpg30
TD 65536
SOLVENT DMSO
NS 2000
DS 4
SWH 23809.523 Hz
FIDRES 0.726609 Hz
AQ 1.3762560 sec
RG 42.0688
DW 21.000 usec
DE 6.50 usec
TE 300.1 K
D1 2.00000000 sec
D11 0.03000000 sec
TD0 1
SFO1 100.6655806 MHz
NUC1 13C
P0 2.67 usec
P1 8.00 usec
PLW1 85.44200134 W
SFO2 400.3016012 MHz
NUC2 1H
CPDPRG[2 waltz65
PCPD2 90.00 usec
PLW2 20.94899940 W
PLW12 0.16552000 W
PLW13 0.08325600 W
F2 - Processing parameters
SI 32768
SF 100.6555669 MHz
WDW EM
SSB 0
LB 1.00 Hz
GB 0
PC 1.40
O
N
N
N
NH2
O
200 190 180 170 160 150 140 130 120 110 100 90 80 70 60 50 40 30 20 10 0 ppm

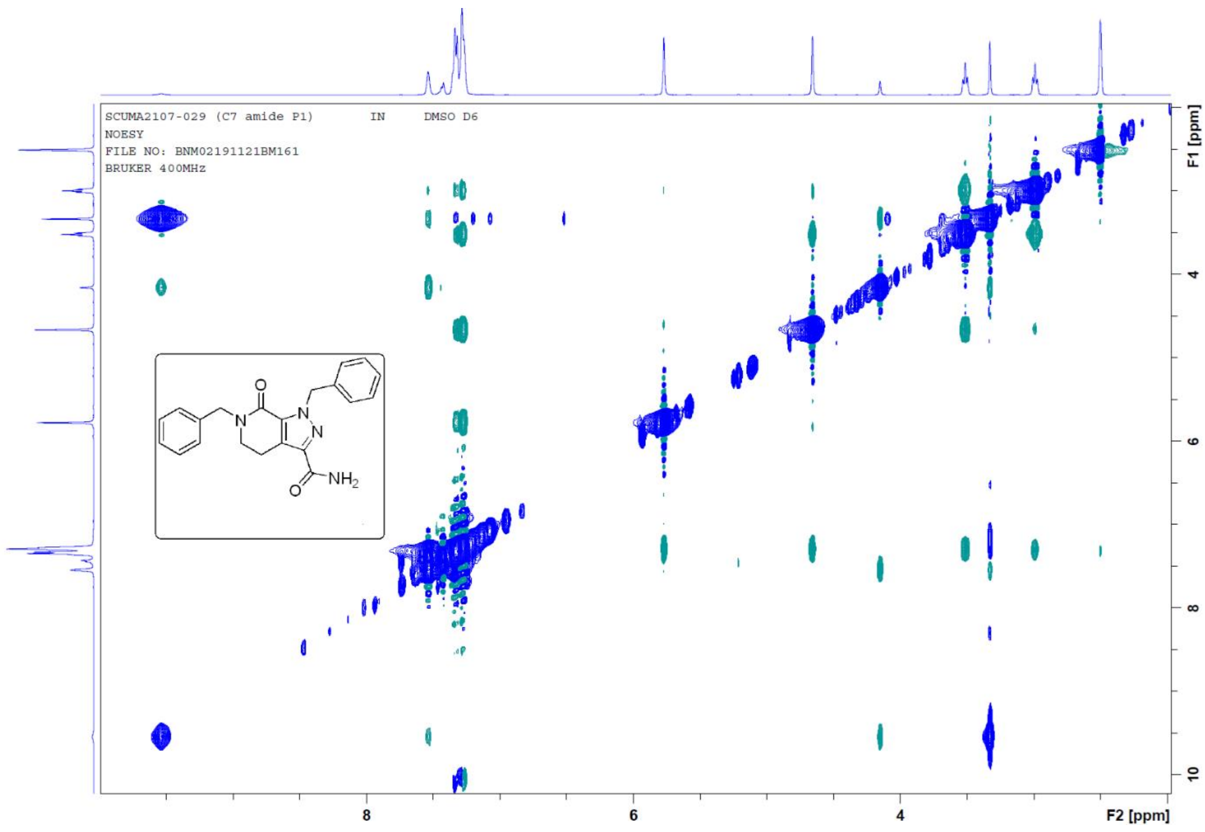
SCUMA2107-029 (C7 amide P1) IN DMSO D6
NOESY
FILE NO: BNM02191121BM161
BRUKER 400MHz
O
N
N
N
NH2
O
8 6 4 F2 [ppm]
4 6 8 10 F1 [ppm]

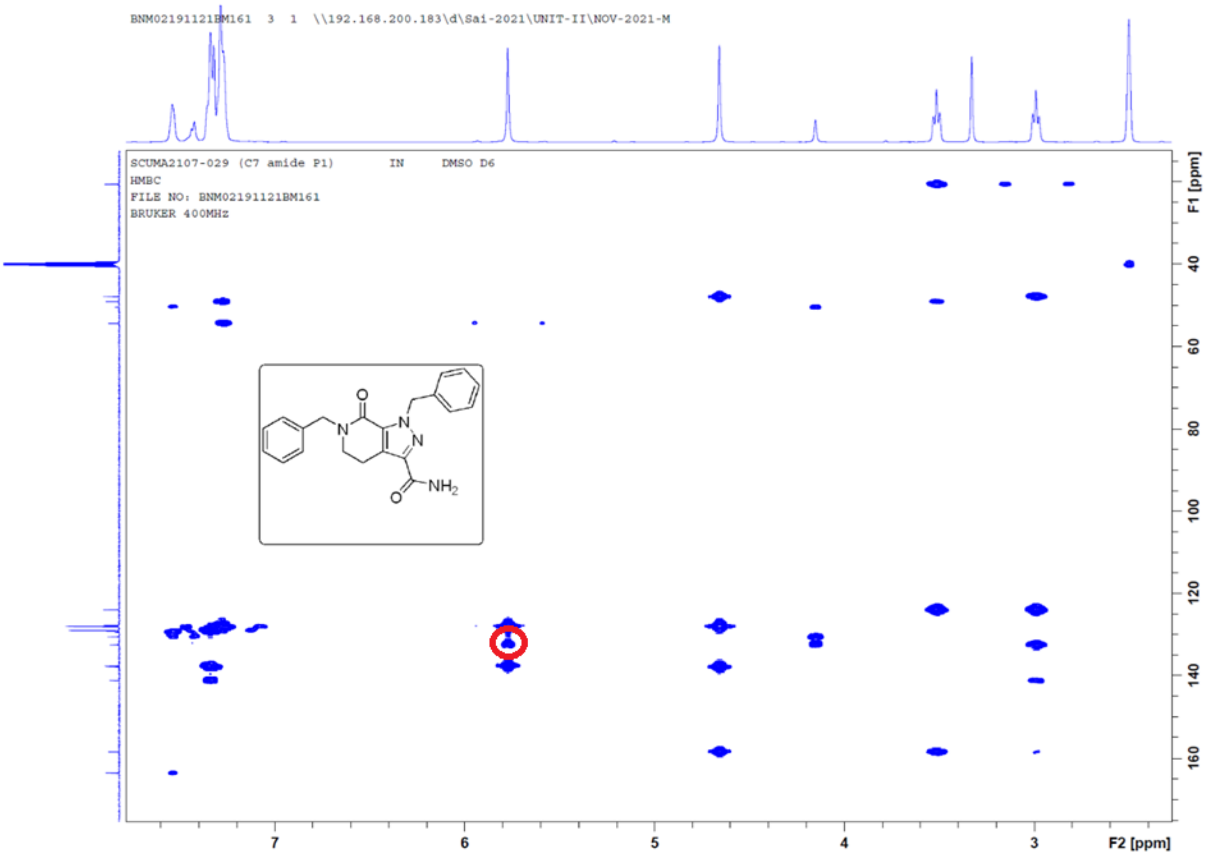

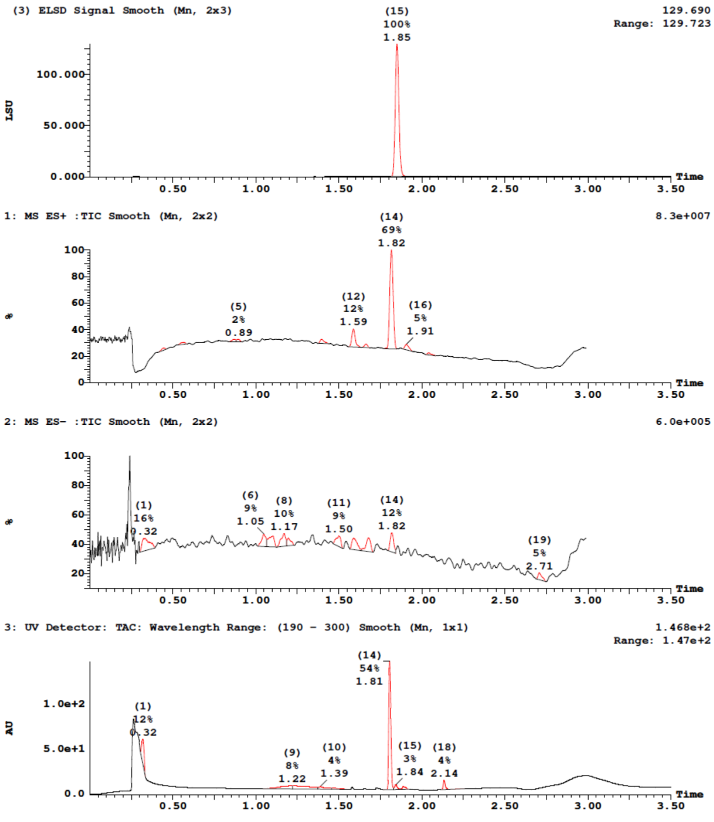

Peak ID	Compound	Time	Mass Found
14		1.82	Not Found

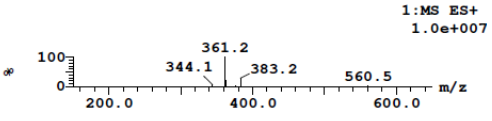

2-Benzyl-7-oxo-6-phenethyl-4,5,6,7-tetrahydro-2*H*-pyrazolo[3,4-*c*]pyridine-3-carboxamide (**58**)

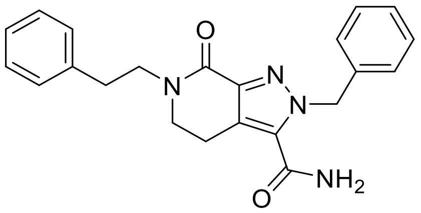

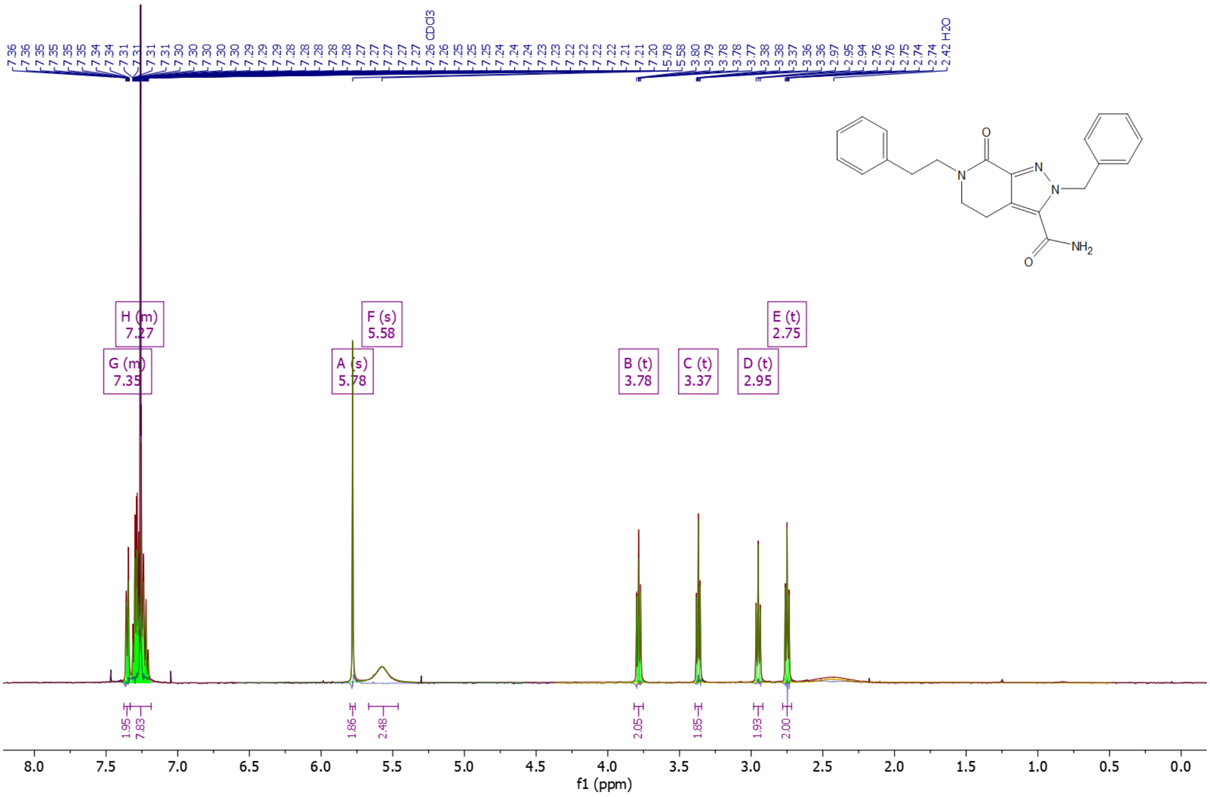

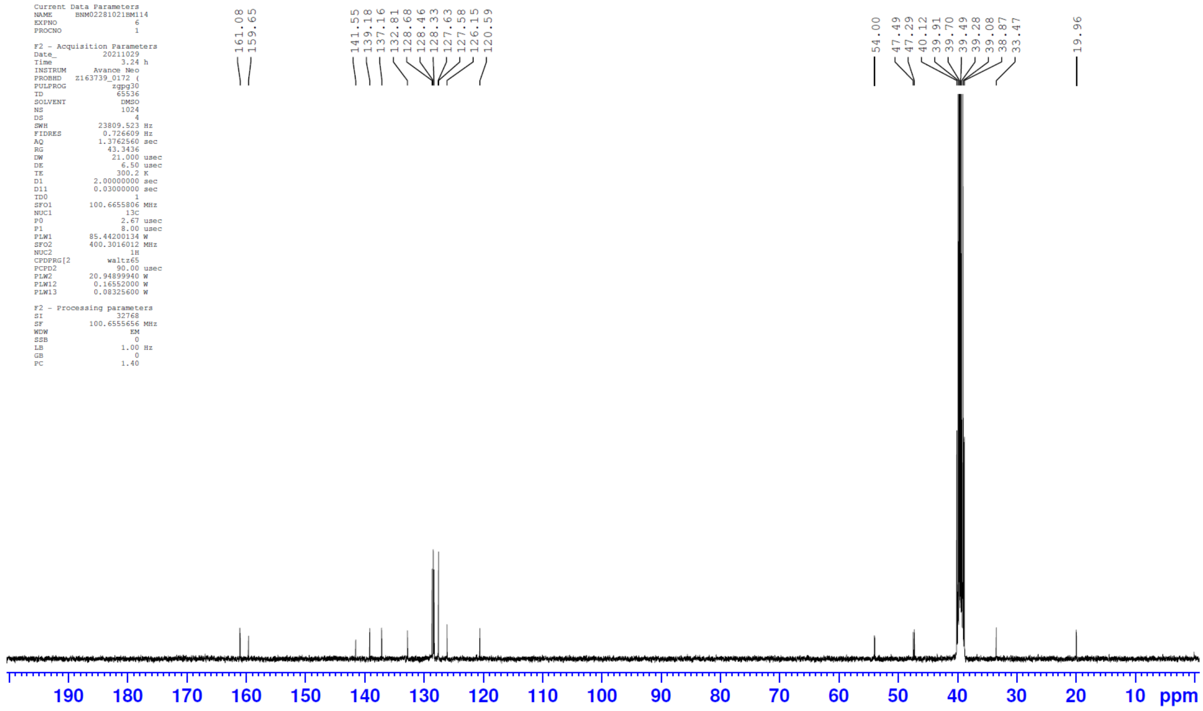

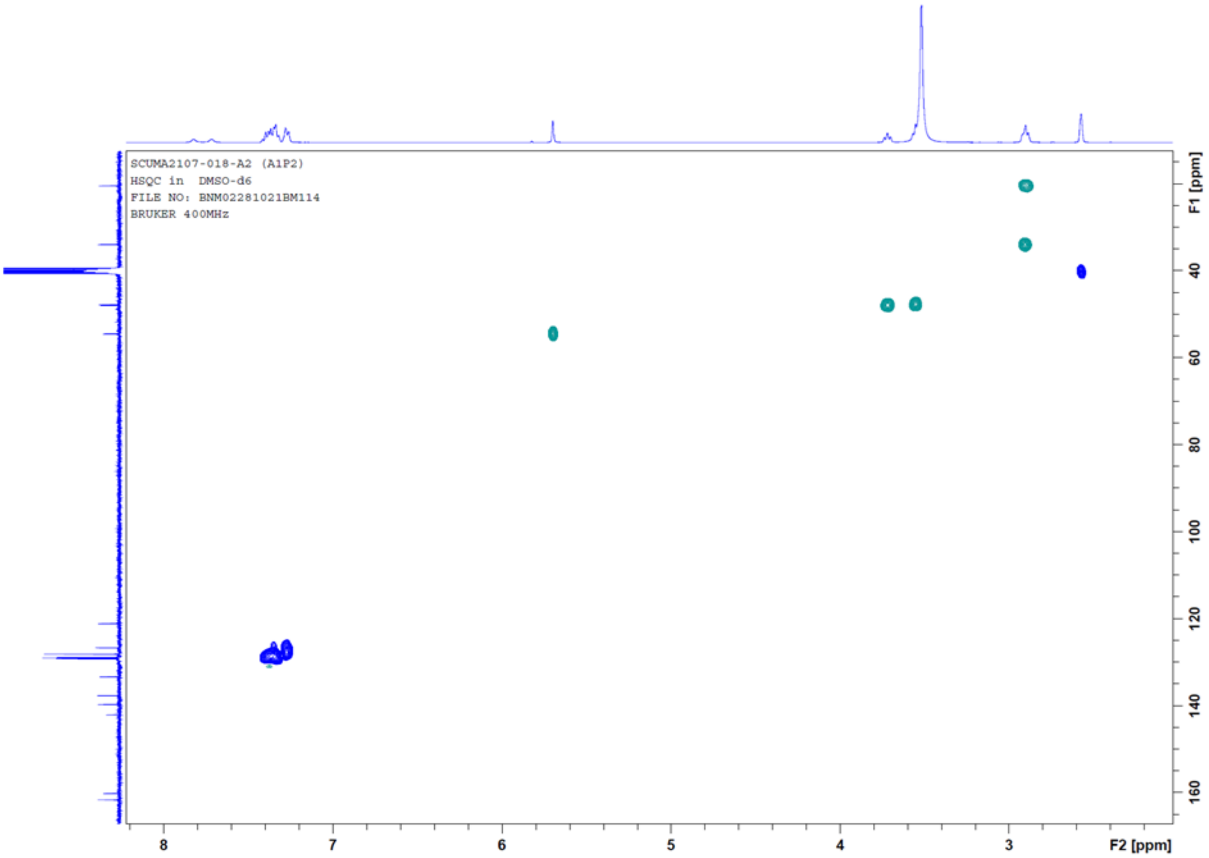
SCUMA2107-018-A2 (A1P2)
HSQC in DMSO-d6
FILE NO: BNM02281021BM114
BRUKER 400MHz
F1 [ppm]
F2 [ppm]

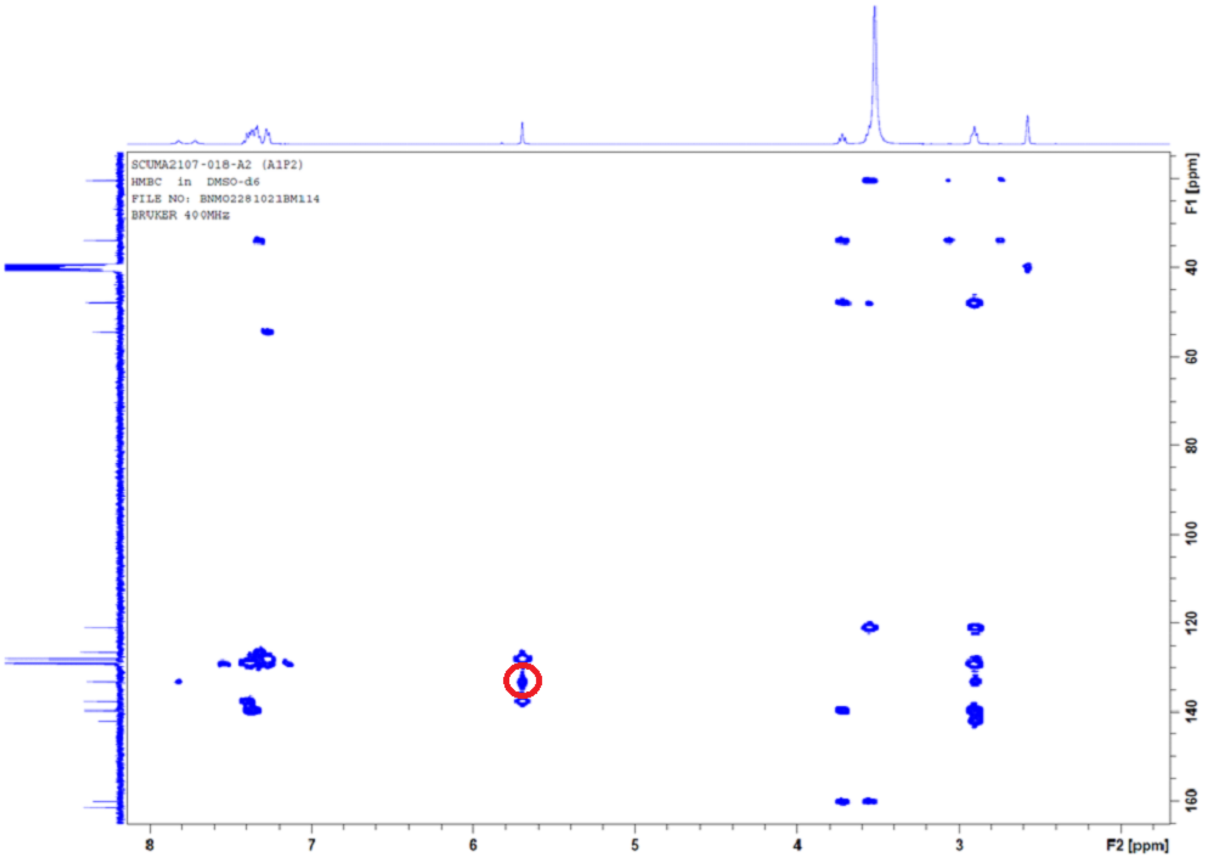
SCUMA2107-018-A2 (A1P2)
HMBC in DMSO-d6
FILE NO: BNM02281021BM114
BRUKER 400MHz
F1 [ppm]
F2 [ppm]

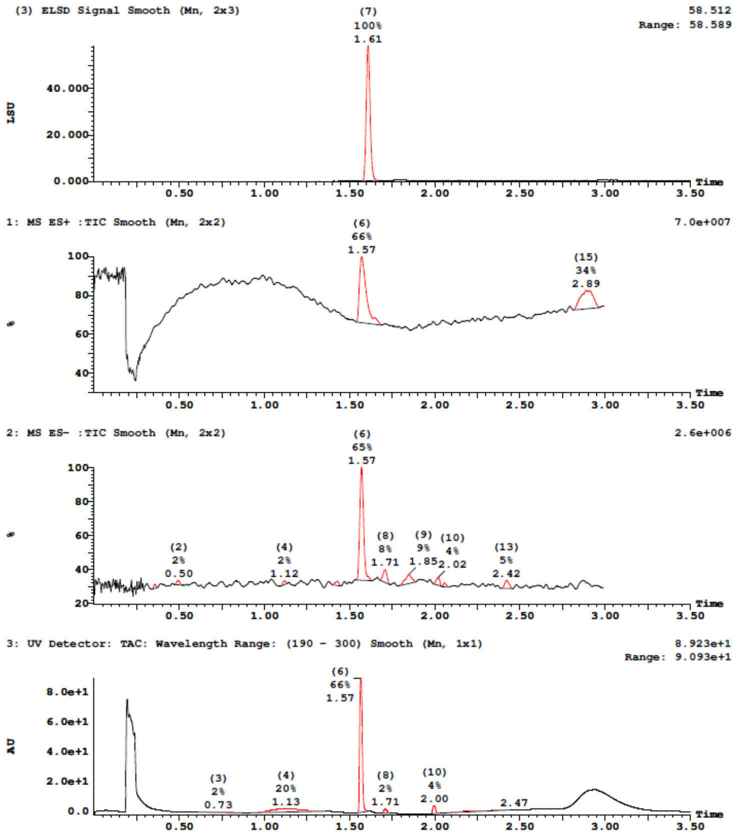

Peak ID	Compound	Time	Mass Found
6		1.57	Not Found

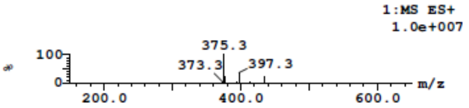

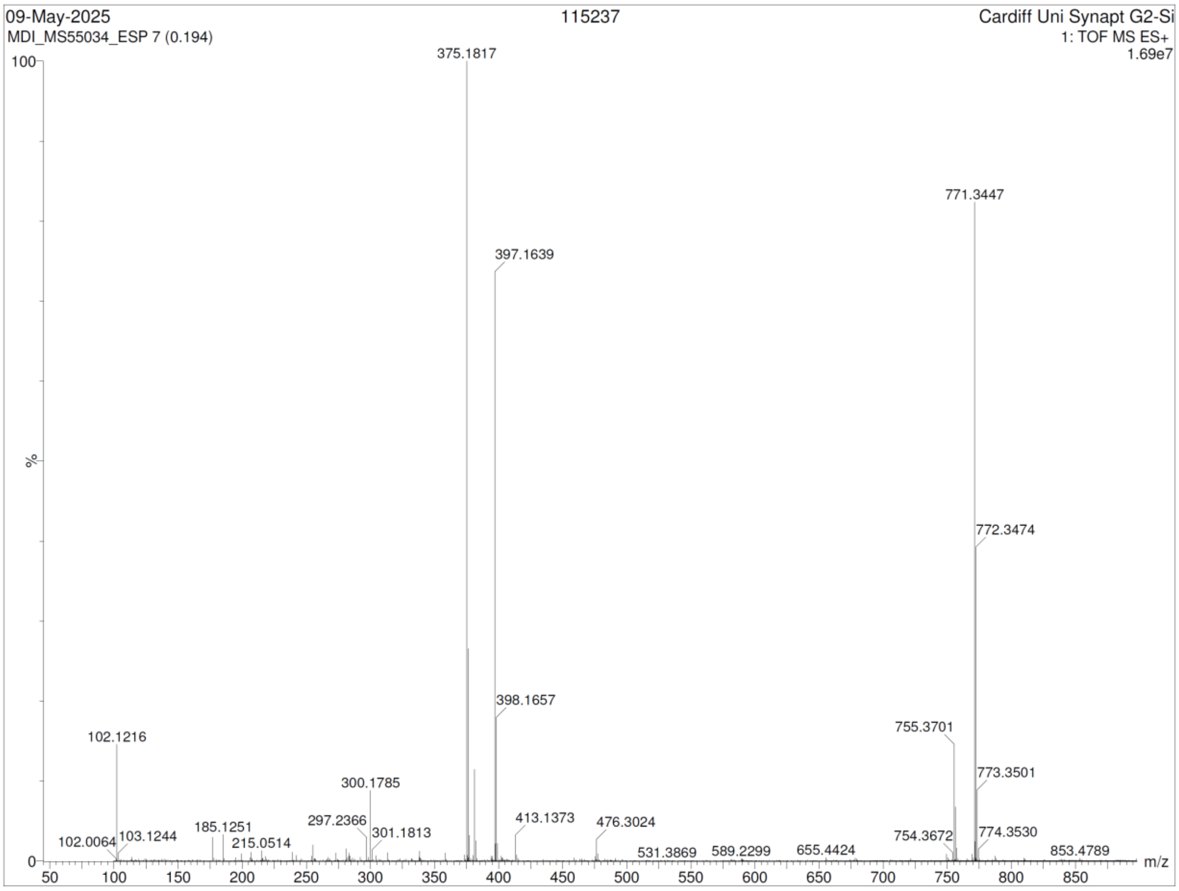

Single Mass Analysis

Tolerance = 10.0 PPM / DBE: min = -1.5, max = 50.0
Element prediction: Off
Number of isotope peaks used for i-FIT = 3

Monoisotopic Mass, Odd and Even Electron Ions
14 formula(e) evaluated with 1 results within limits (up to 50 closest results for each mass)
Elements Used:
C: 0-22 H: 0-23 N: 0-4 O: 0-3

09-May-2025 115237 Cardiff Uni Synapt G2-Si
MDI_MS55034_ESP 7 (0.194) 1: TOF MS ES+
1.69e+007

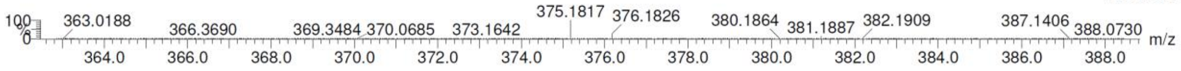

Minimum:				-1.5				
Maximum:		5.0	10.0	50.0				
Mass	Calc. Mass	mDa	PPM	DBE	i-FIT	Norm	Conf(%)	Formula
375.1817	375.1821	-0.4	-1.1	13.5	1176.0	n/a	n/a	C22 H23 N4 O2

2-Benzyl-7-oxo-6-(2-phenoxyethyl)-4,5,6,7-tetrahydro-2*H*-pyrazolo[3,4-*c*]pyridine-3-carboxamide (**59**)

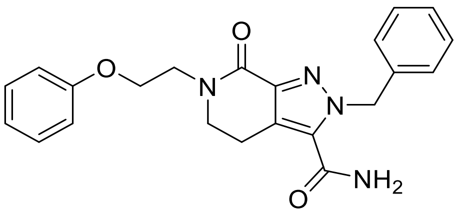

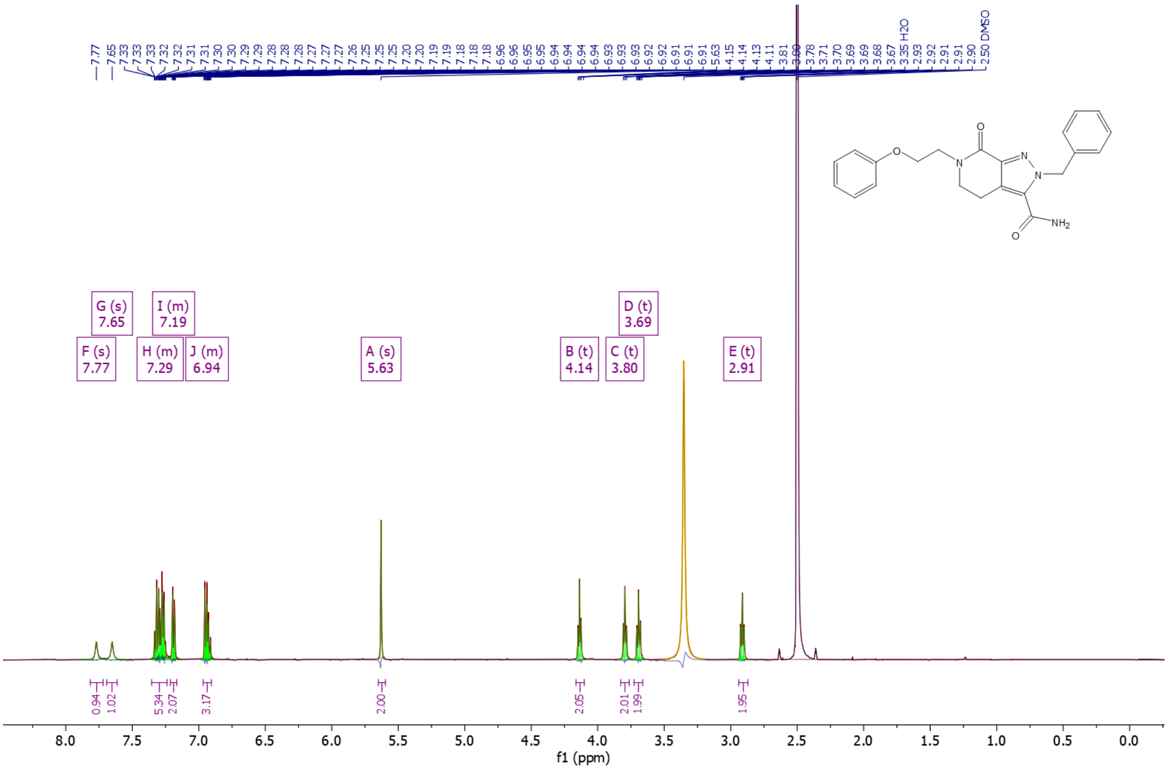

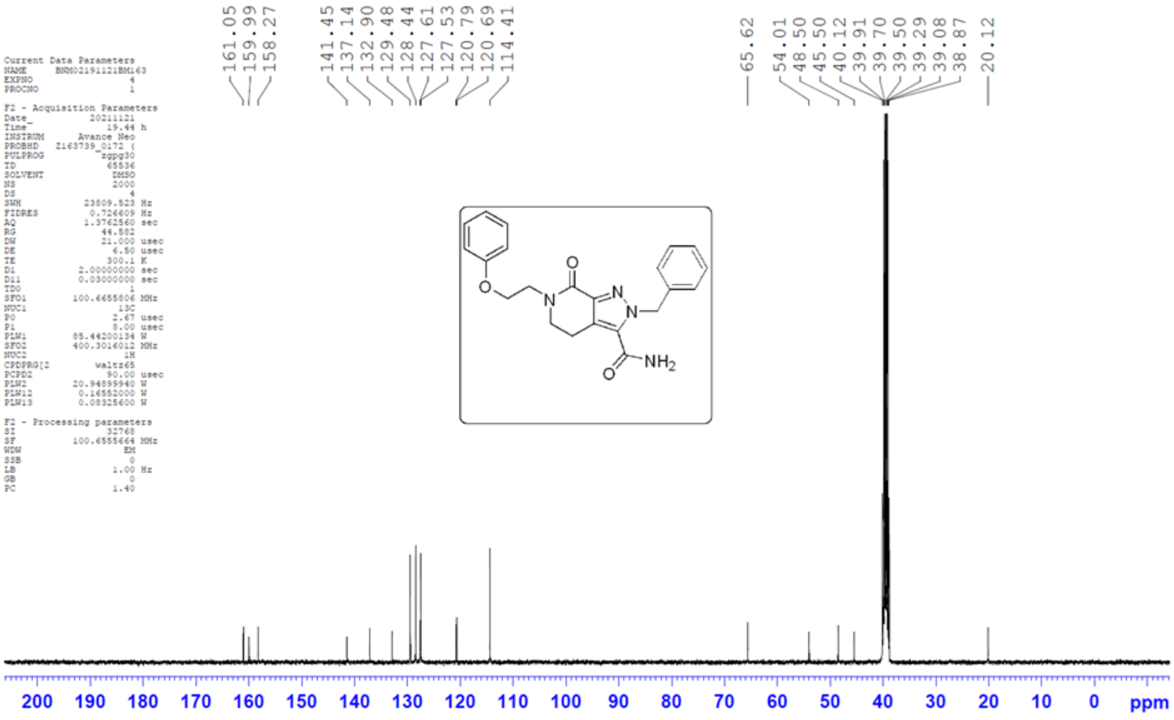

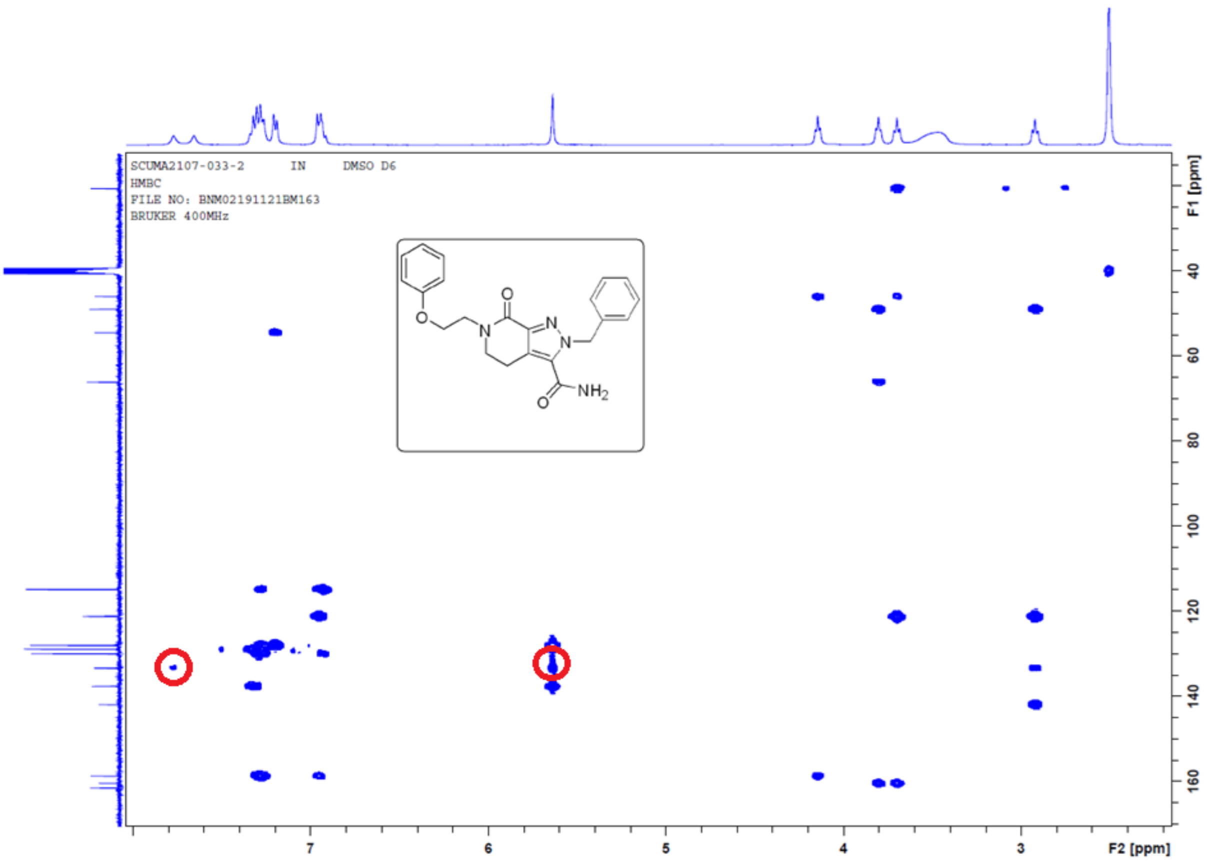

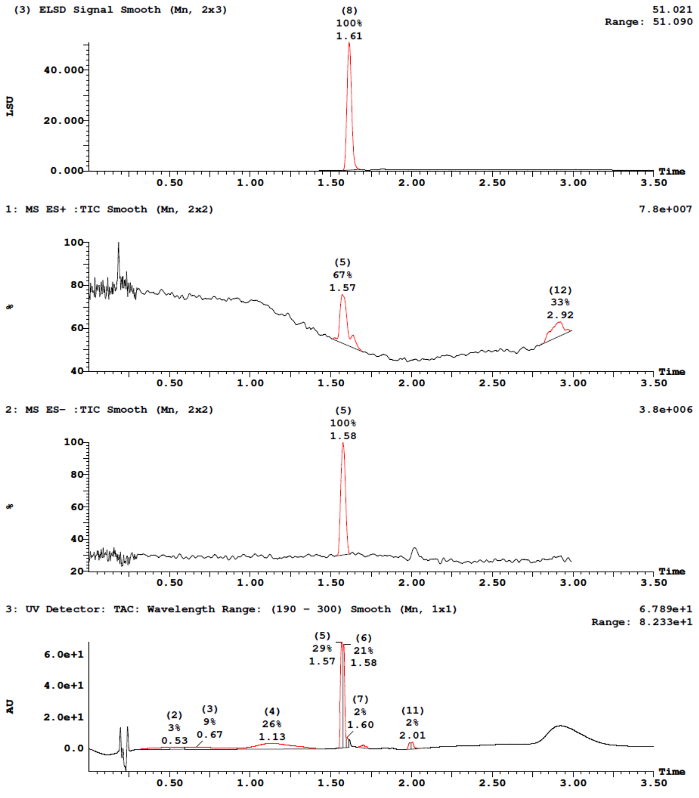

Peak ID	Compound	Time	Mass Found
5		1.57	Not Found

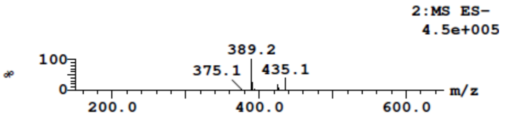

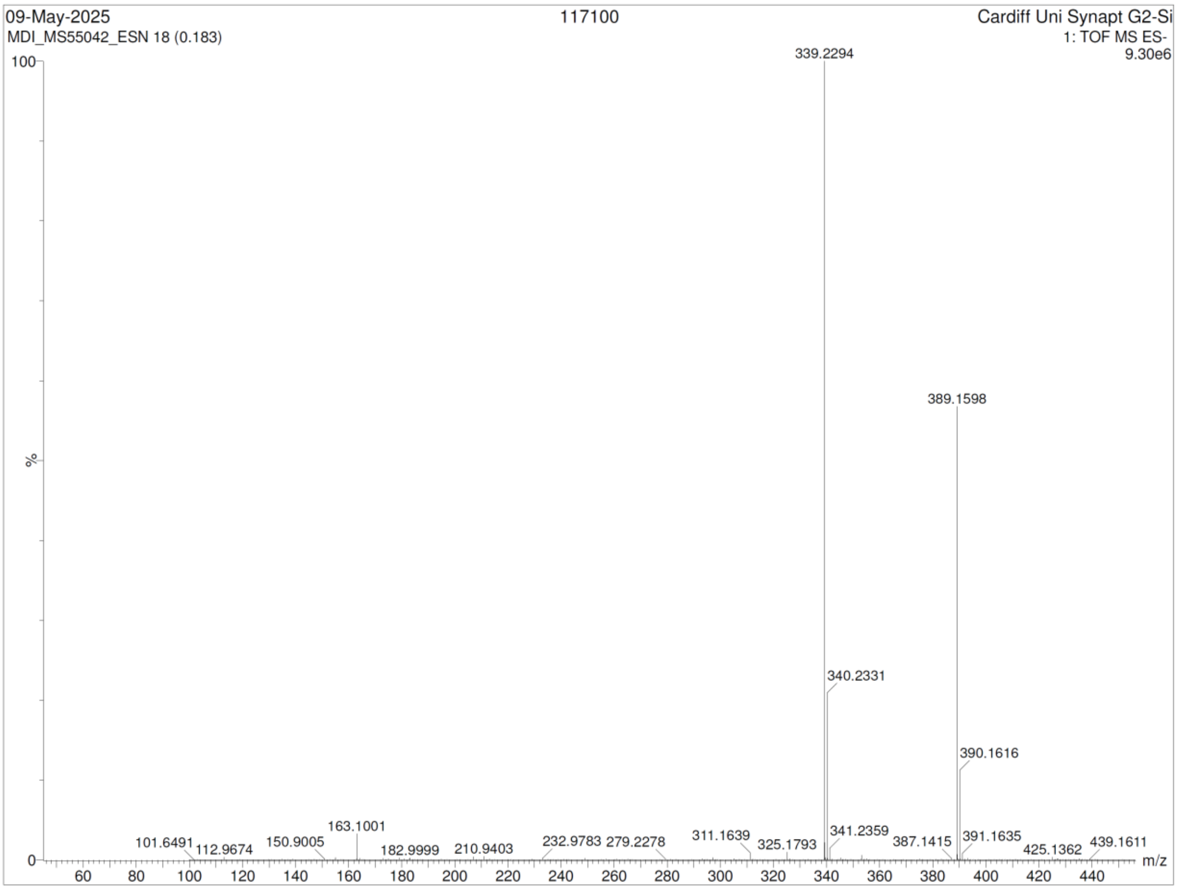

Elemental Composition Report

Page 1

Single Mass Analysis

Tolerance = 10.0 PPM / DBE: min = -1.5, max = 50.0
Element prediction: Off
Number of isotope peaks used for i-FIT = 3

Monoisotopic Mass, Odd and Even Electron Ions
15 formula(e) evaluated with 1 results within limits (up to 50 closest results for each mass)
Elements Used:
C: 0-22 H: 0-21 N: 0-4 O: 0-3

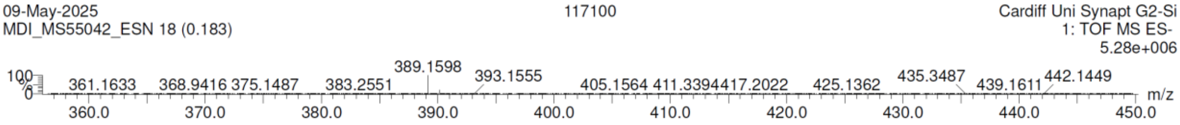

Minimum:				-1.5				
Maximum:		5.0	10.0	50.0				
Mass	Calc. Mass	mDa	PPM	DBE	i-FIT	Norm	Conf(%)	Formula
389.1598	389.1614	-1.6	-4.1	14.5	884.5	n/a	n/a	C22 H21 N4 O3

2-Benzyl-6-(4-hydroxybenzyl)-7-oxo-4,5,6,7-tetrahydro-2*H*-pyrazolo[3,4-*c*]pyridine-3-carboxamide (**60**)

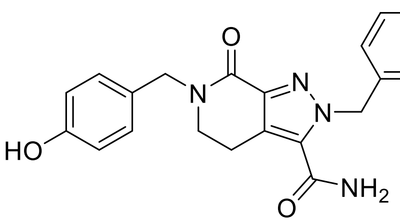

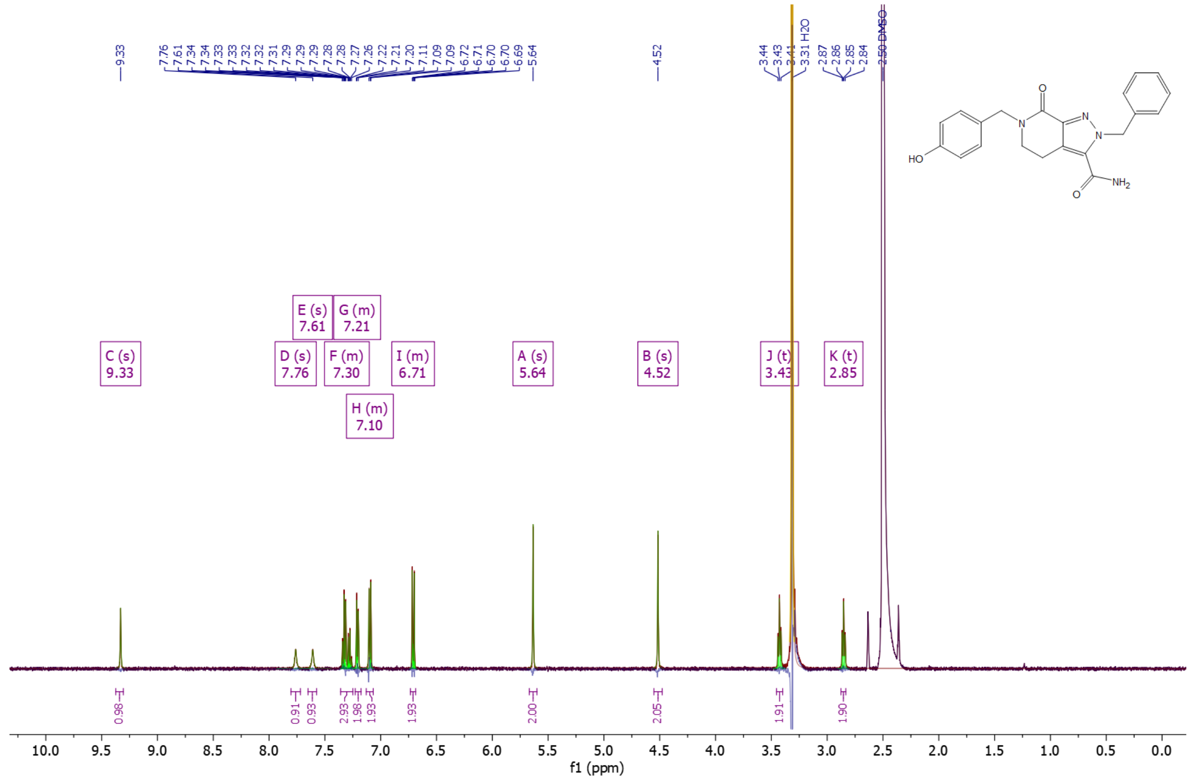

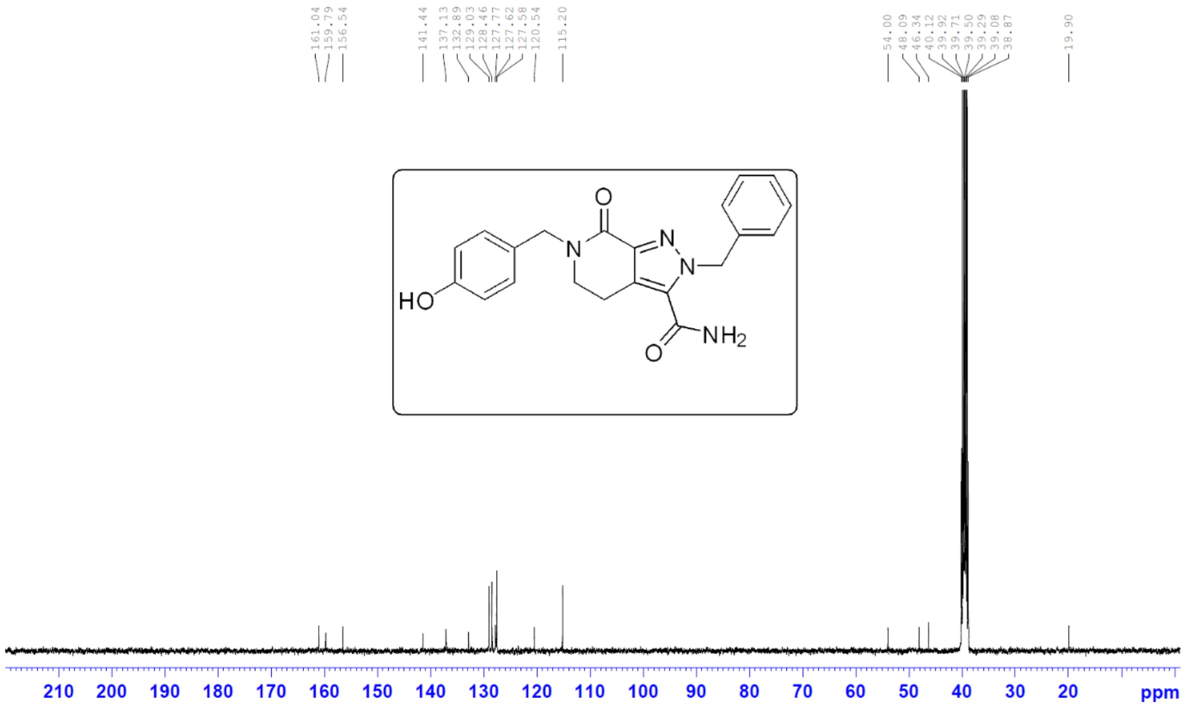

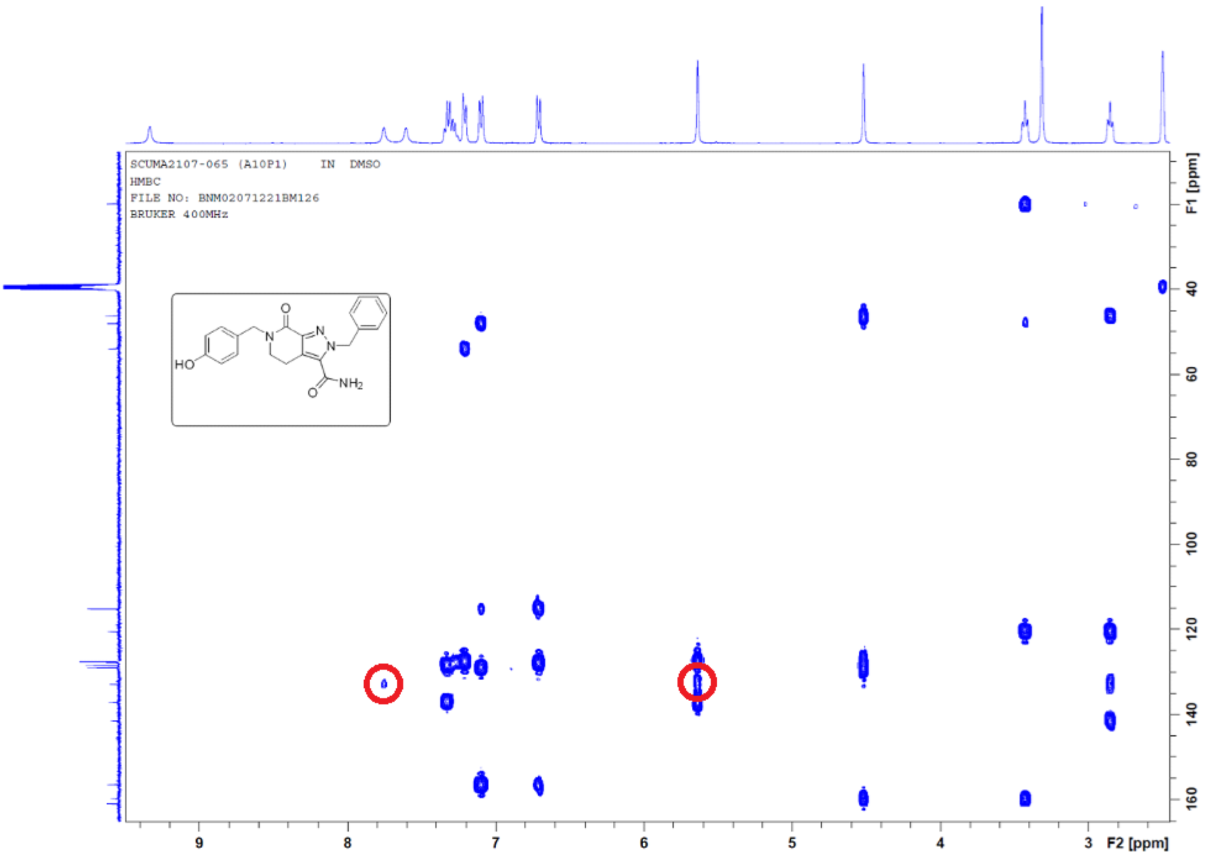

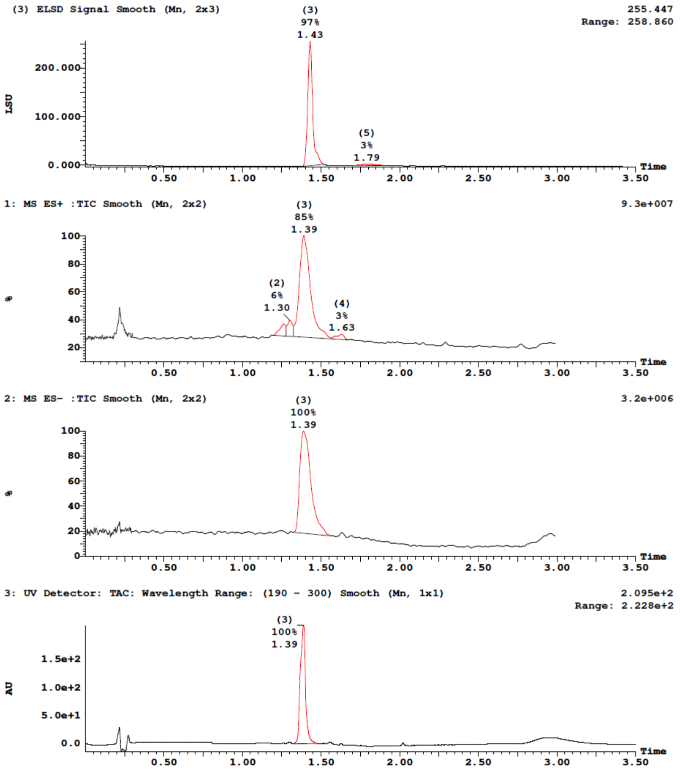

Peak ID	Compound	Time	Mass Found
3		1.39	Not Found

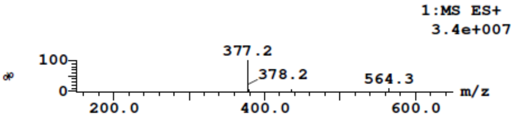

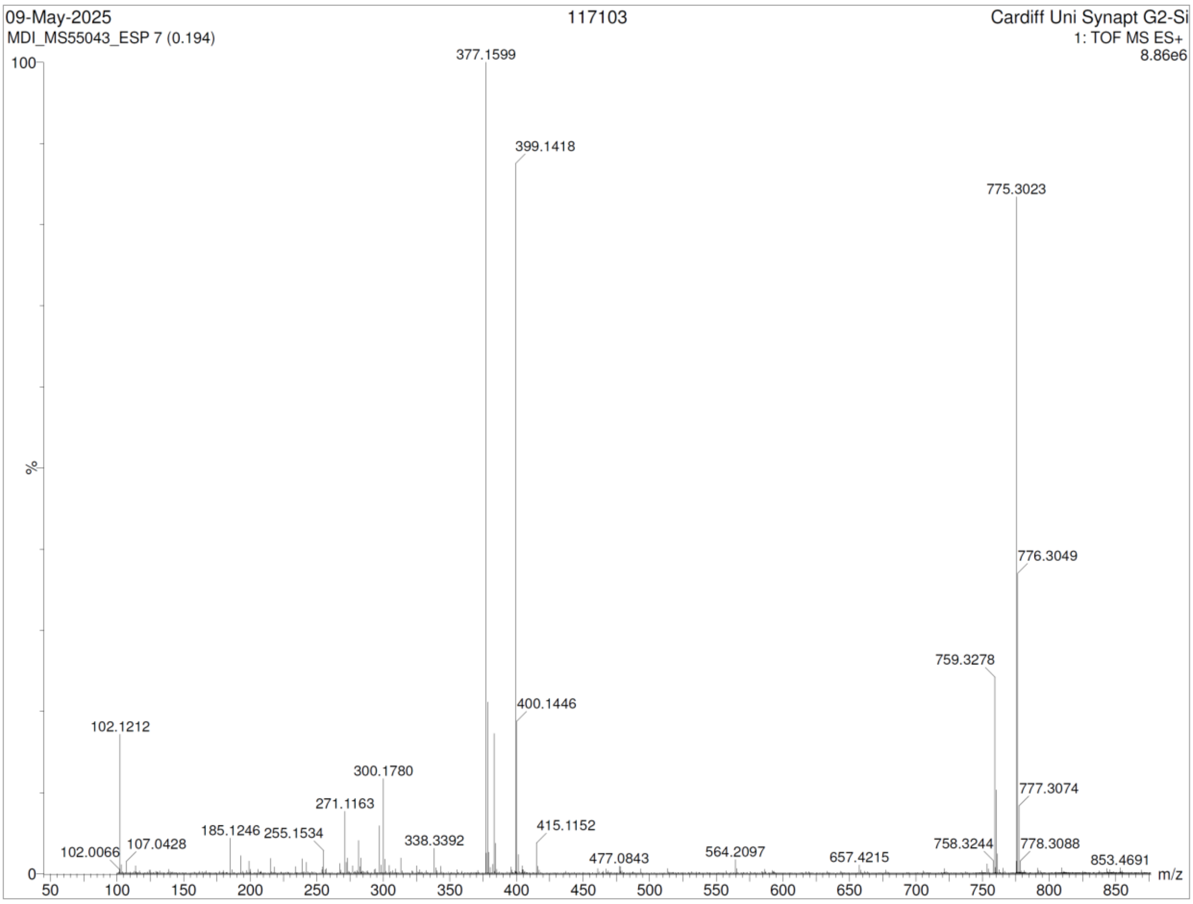

Elemental Composition Report

Page 1

Single Mass Analysis

Tolerance = 10.0 PPM / DBE: min = -1.5, max = 50.0
Element prediction: Off
Number of isotope peaks used for i-FIT = 3

Monoisotopic Mass, Odd and Even Electron Ions
15 formula(e) evaluated with 1 results within limits (up to 50 closest results for each mass)
Elements Used:
C: 0-21 H: 0-21 N: 0-4 O: 0-3

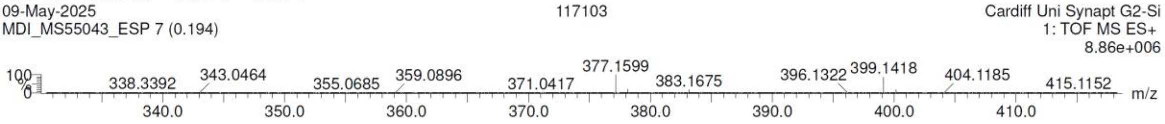

Minimum:				-1.5				
Maximum:		5.0	10.0	50.0				
Mass	Calc. Mass	mDa	PPM	DBE	i-FIT	Norm	Conf(%)	Formula
377.1599	377.1614	-1.5	-4.0	13.5	1171.4	n/a	n/a	C21 H21 N4 O3

2-Benzyl-6-(4-methoxybenzyl)-7-oxo-4,5,6,7-tetrahydro-2*H*-pyrazolo[3,4-*c*]pyridine-3-carboxamide (**61**)

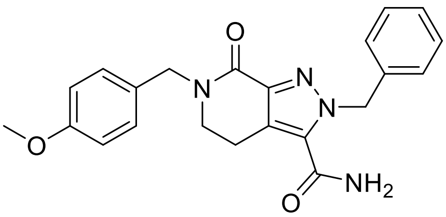

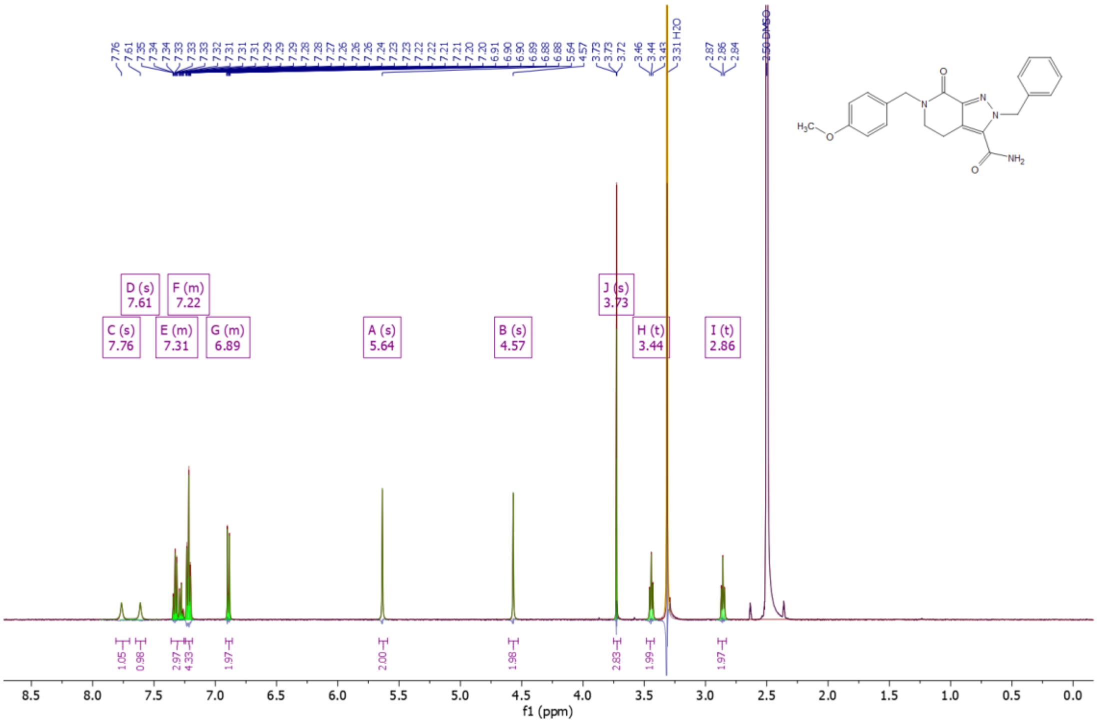

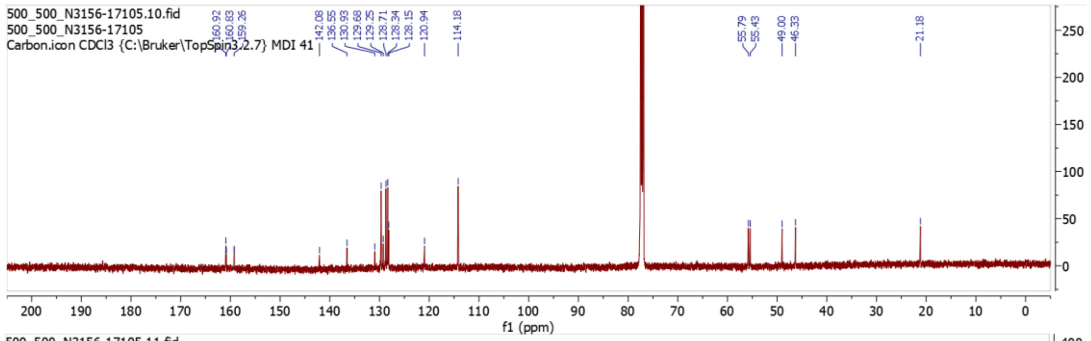

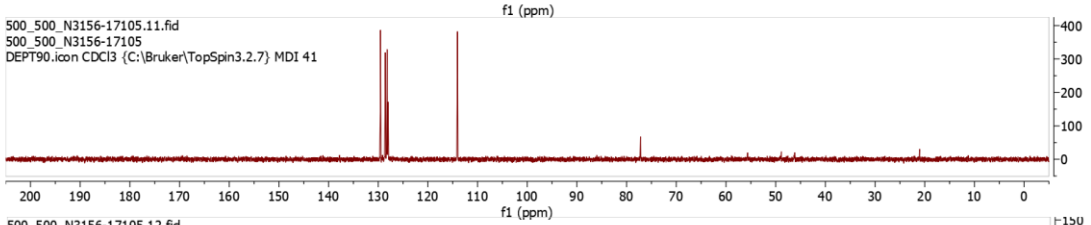

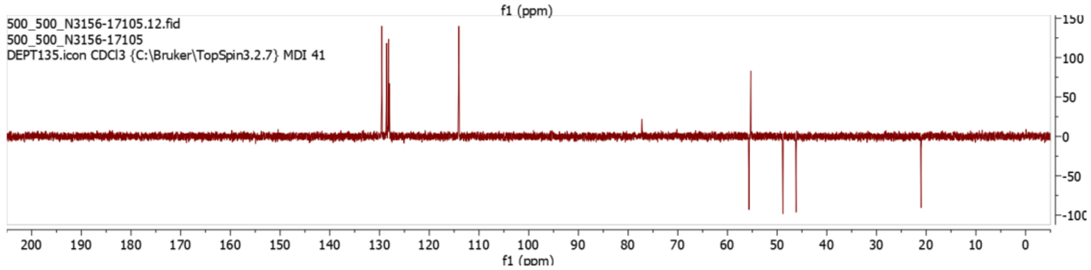

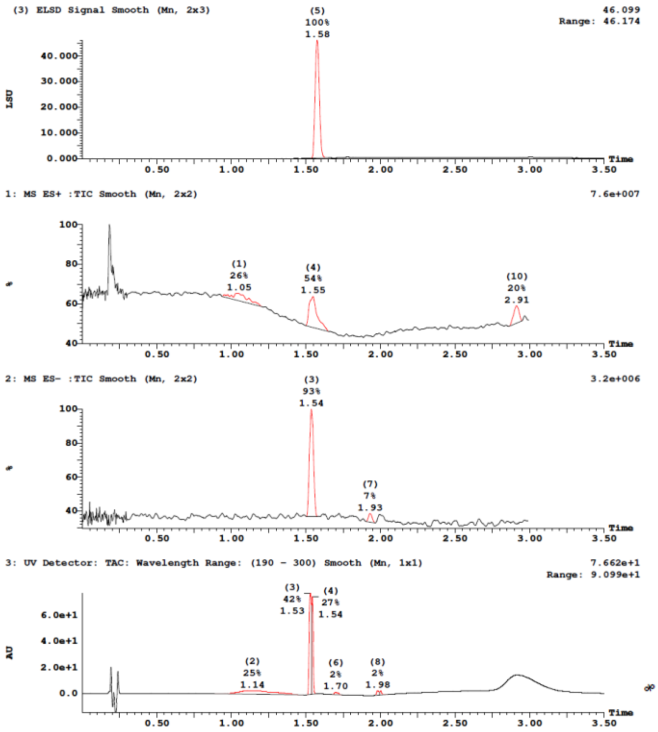

Peak ID	Compound	Time	Mass Found
3		1.54	Not Found

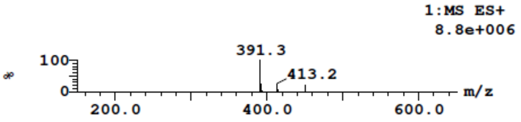

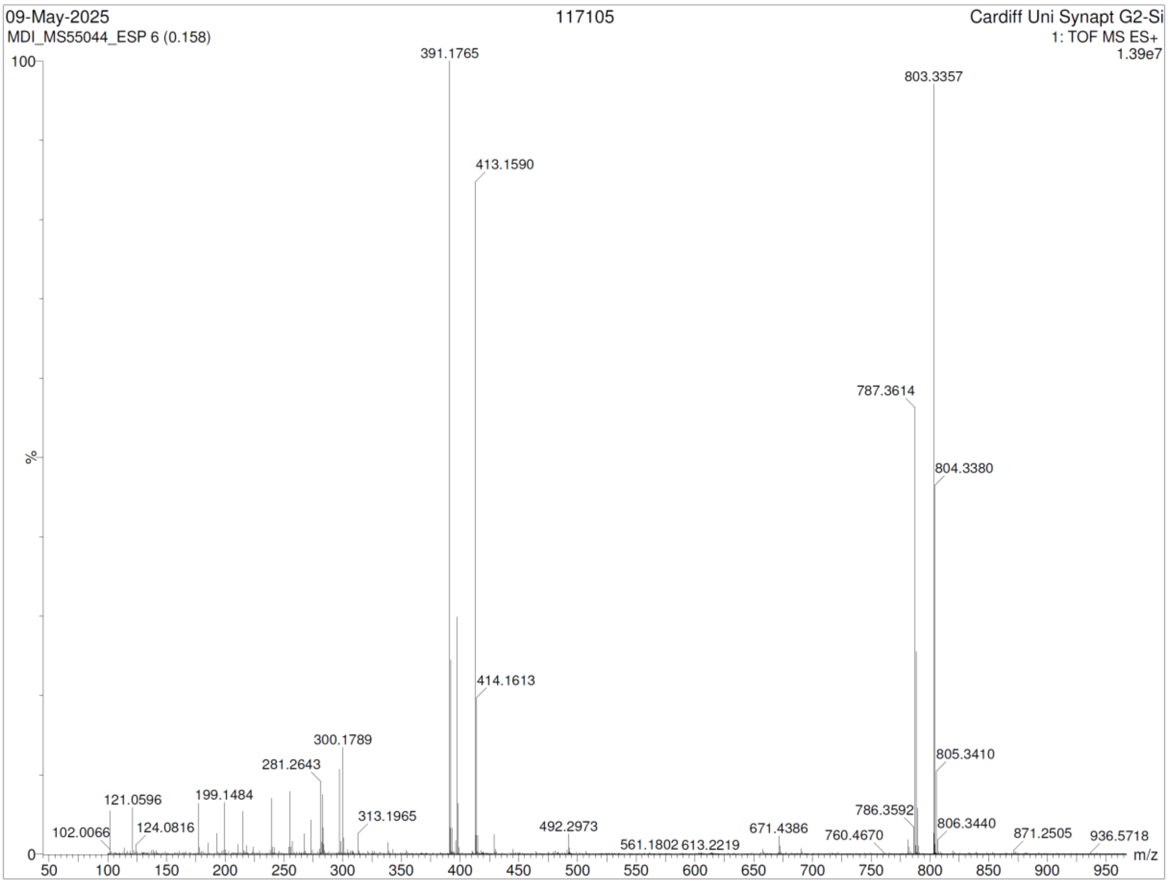

Elemental Composition Report

Single Mass Analysis

Tolerance = 10.0 PPM / DBE: min = -1.5, max = 50.0
Element prediction: Off
Number of isotope peaks used for i-FIT = 3

Monoisotopic Mass, Odd and Even Electron Ions
15 formula(e) evaluated with 1 results within limits (up to 50 closest results for each mass)
Elements Used:
C: 0-22 H: 0-23 N: 0-4 O: 0-3

09-May-2025 117105 Cardiff Uni Synapt G2-Si
MDI_MS55044_ESP 6 (0.158) 1: TOF MS ES+
1.39e+007

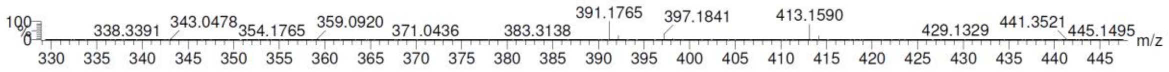

Minimum:				-1.5				
Maximum:		5.0	10.0	50.0				
Mass	Calc. Mass	mDa	PPM	DBE	i-FIT	Norm	Conf(%)	Formula
391.1765	391.1770	-0.5	-1.3	13.5	1093.2	n/a	n/a	C22 H23 N4 O3

2-Benzyl-6-(3-hydroxyphenethyl)-7-oxo-4,5,6,7-tetrahydro-2*H*-pyrazolo[3,4-*c*]pyridine-3-carboxamide (**62**)

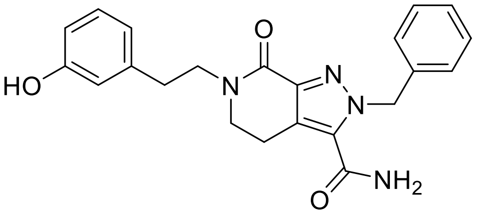

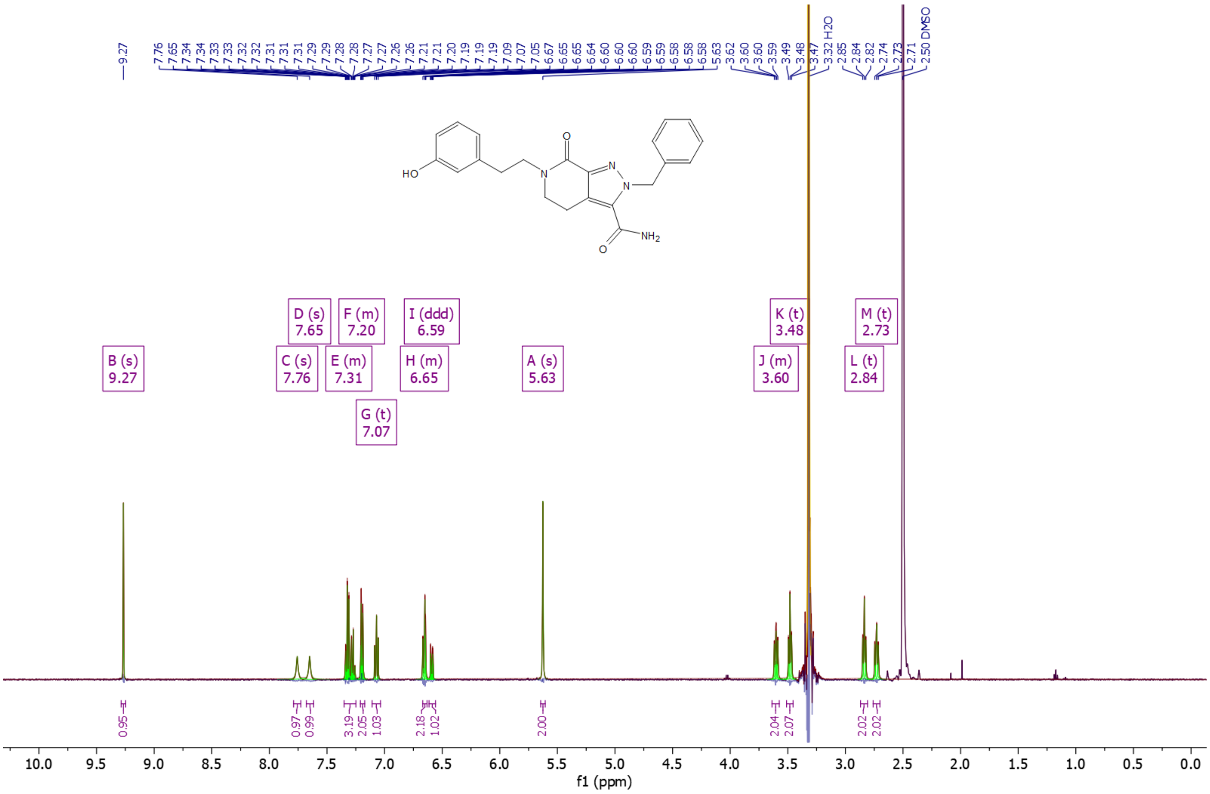

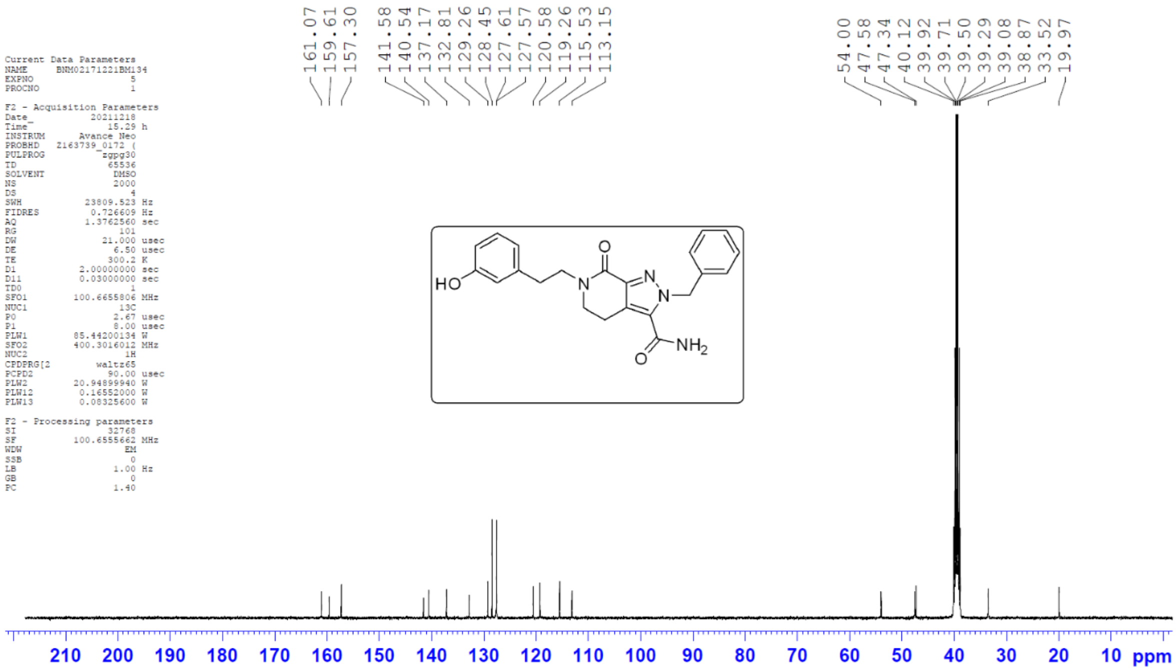

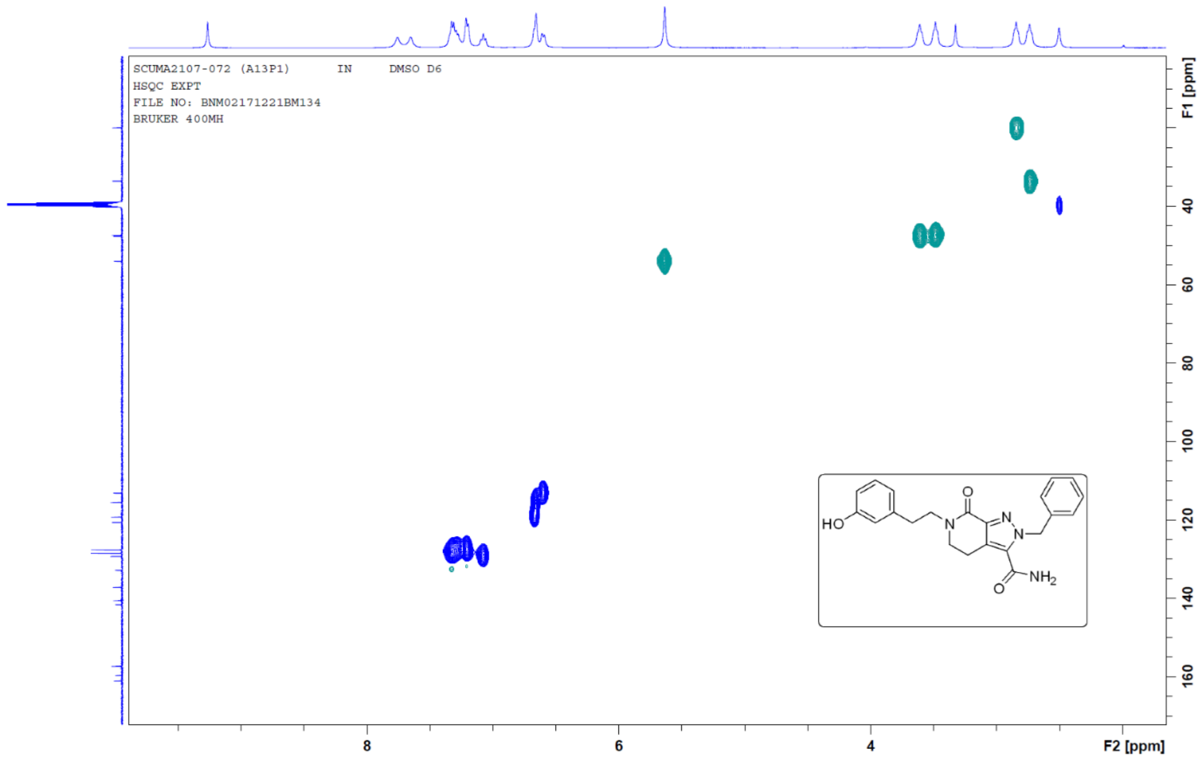
SCUMA2107-072 (A13P1) IN DMSO D6
HSQC EXPT
FILE NO: BNM02171221BM134
BRUKER 400MH
HO
O
N
N
N
NH2
O
F1 [ppm]
F2 [ppm]

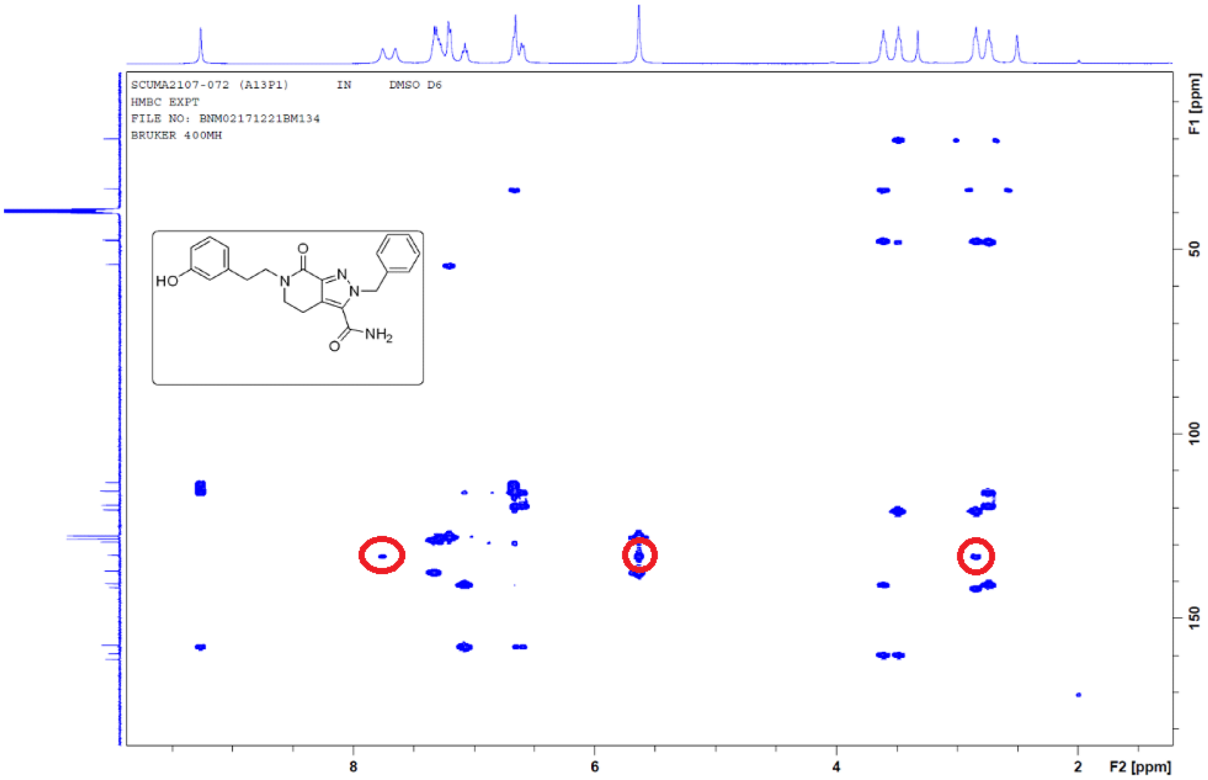
SCUMA2107-072 (A13P1) IN DMSO D6
HMBC EXPT
FILE NO: BNM02171221BM134
BRUKER 400MH
HO
O
N
N
N
NH2
O
F1 [ppm]
F2 [ppm]

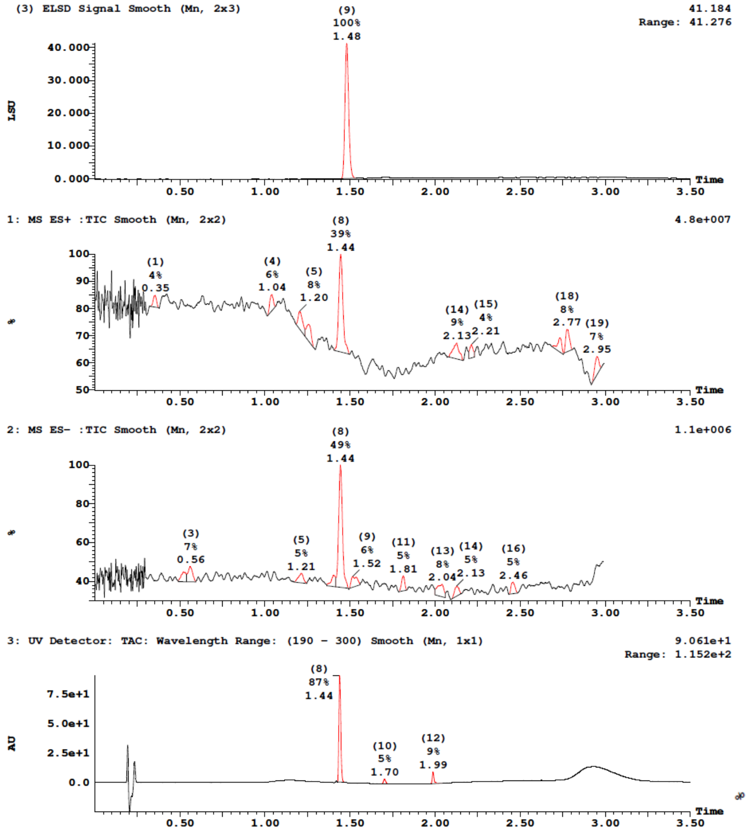

Peak ID	Compound	Time	Mass Found
8		1.44	Not Found

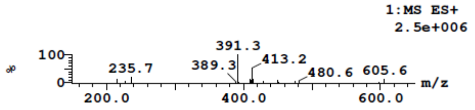

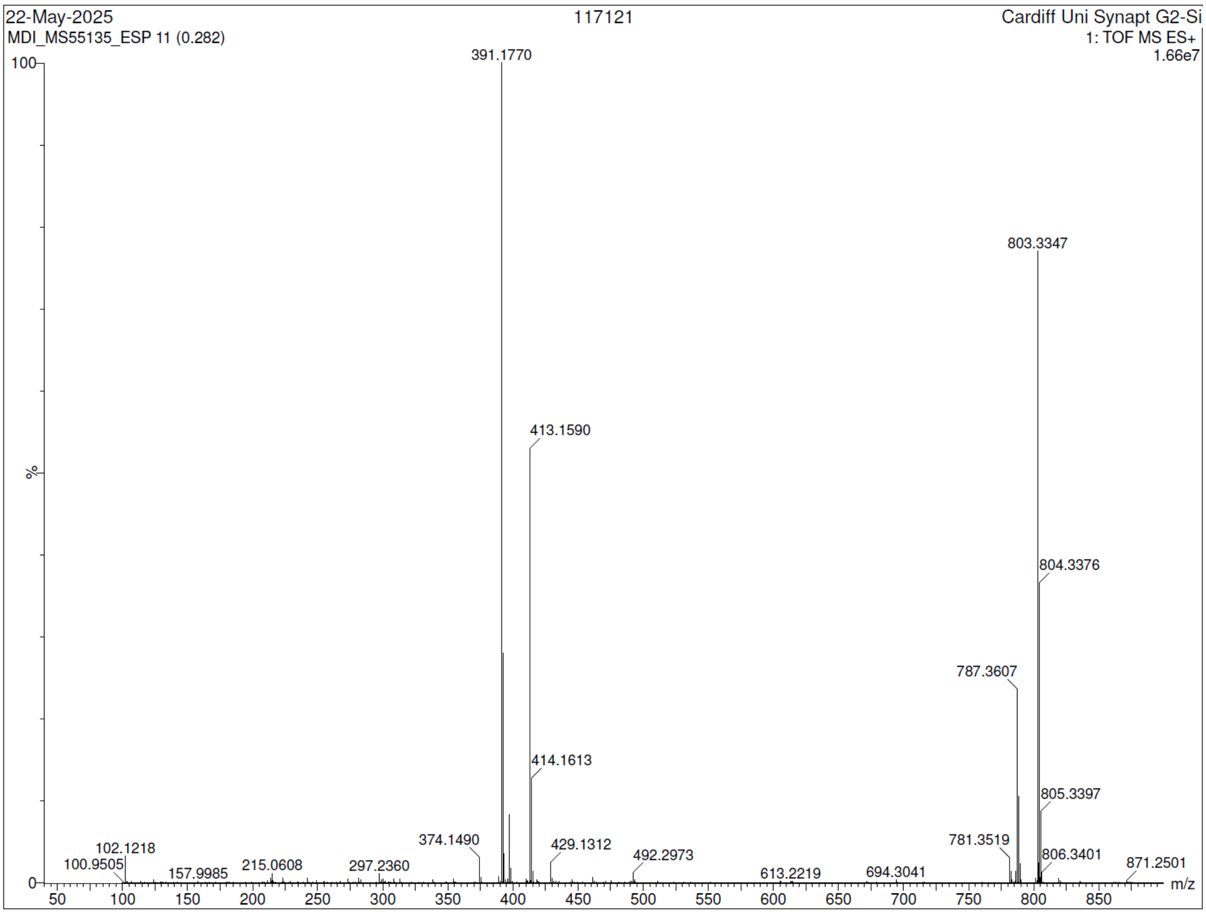

Elemental Composition Report

Single Mass Analysis
Tolerance = 50.0 PPM / DBE: min = -1.5, max = 50.0
Element prediction: Off
Number of isotope peaks used for i-FIT = 3

Monoisotopic Mass, Odd and Even Electron Ions
15 formula(e) evaluated with 1 results within limits (up to 50 closest results for each mass)
Elements Used:
C: 0-22 H: 0-23 N: 0-4 O: 0-3

22-May-2025 117121 Cardiff Uni Synapt G2-Si
MDI_MS55135_ESP 11 (0.282) 1: TOF MS ES+
1.66e+007

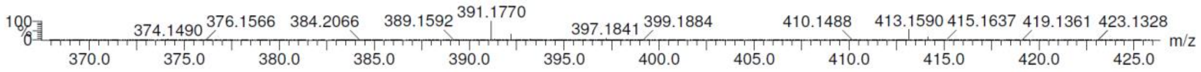

Mass	Calc. Mass	mDa	PPM	DBE	i-FIT	Norm	Conf(%)	Formula
Minimum:				-1.5				
Maximum:		5.0	50.0	50.0				
391.1770	391.1770	0.0	0.0	13.5	1153.2	n/a	n/a	C22 H23 N4 O3

2-Benzyl-6-(3-methoxyphenethyl)-7-oxo-4,5,6,7-tetrahydro-2*H*-pyrazolo[3,4-*c*]pyridine-3-carboxamide (**63**)

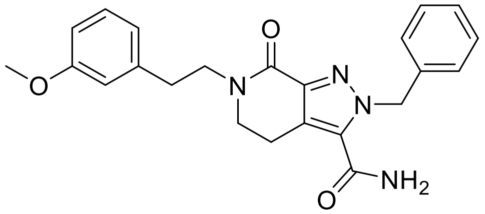

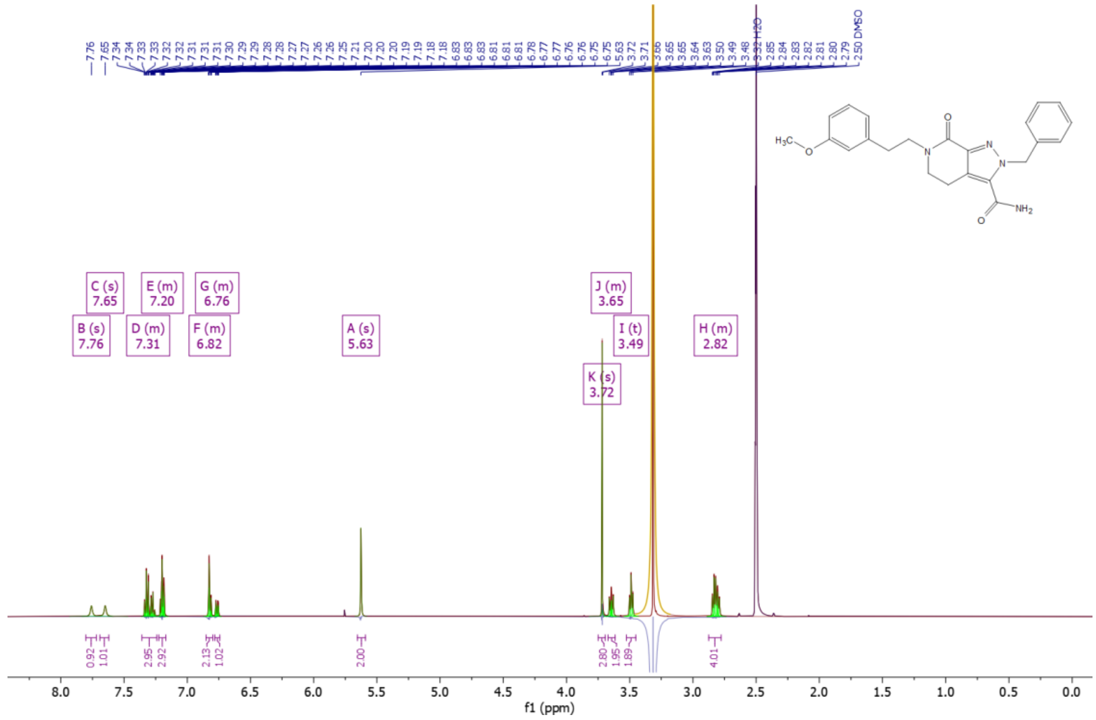

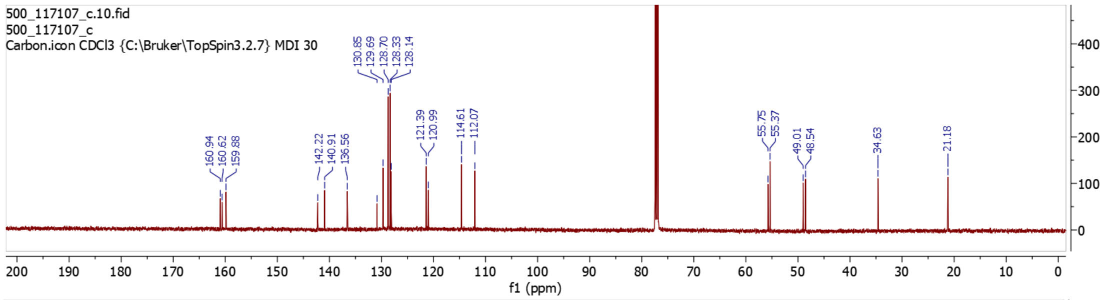

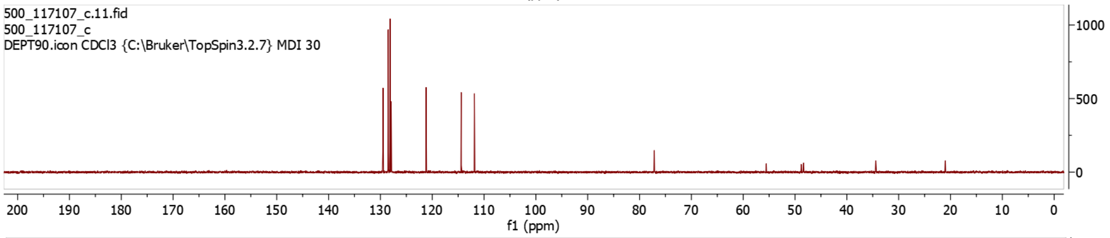

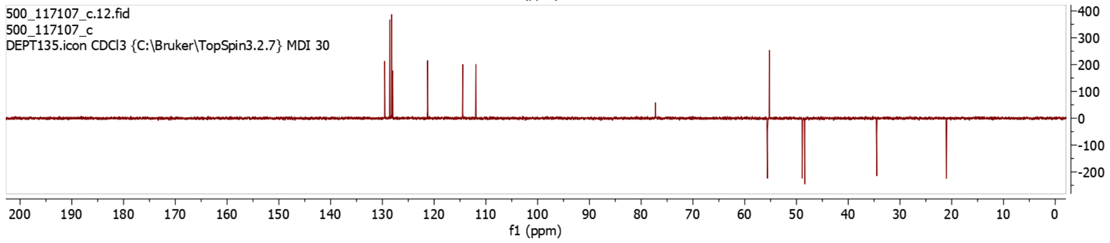

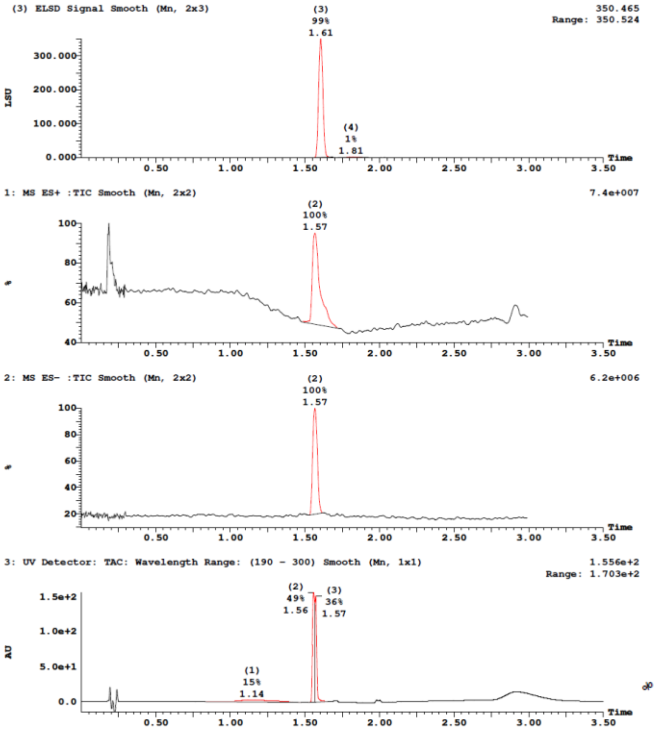

Peak ID	Compound	Time	Mass Found
2		1.57	Not Found

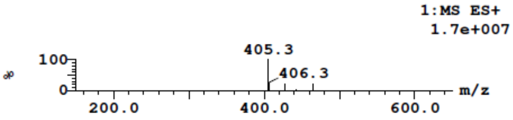

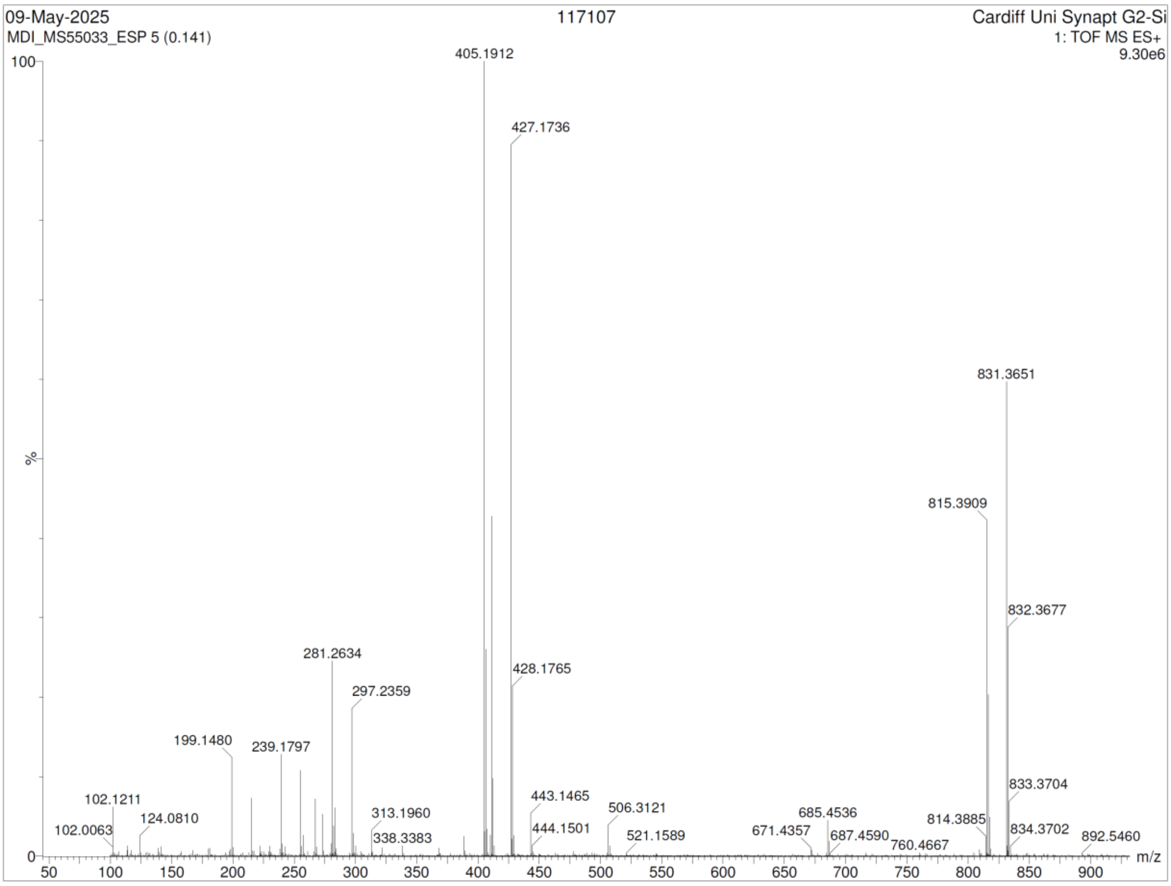

Elemental Composition Report **Page 1**

Single Mass Analysis

Tolerance = 10.0 PPM / DBE: min = -1.5, max = 50.0
Element prediction: Off
Number of isotope peaks used for i-FIT = 3

Monoisotopic Mass, Odd and Even Electron Ions
15 formula(e) evaluated with 1 results within limits (up to 50 closest results for each mass)
Elements Used:
C: 0-23 H: 0-25 N: 0-4 O: 0-3

09-May-2025 117107 Cardiff Uni Synapt G2-Si
MDI_MS55033_ESP 5 (0.141) 1: TOF MS ES+
9.30e+006

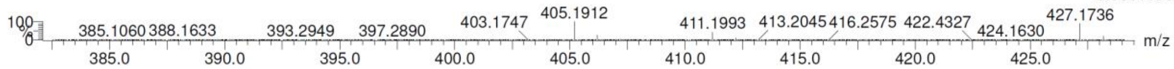

Minimum:				-1.5				
Maximum:		5.0	10.0	50.0				
Mass	Calc. Mass	mDa	PPM	DBE	i-FIT	Norm	Conf(%)	Formula
405.1912	405.1927	-1.5	-3.7	13.5	1111.0	n/a	n/a	C23 H25 N4 O3

2-Benzyl-6-(2-methoxyethyl)-7-oxo-4,5,6,7-tetrahydro-2*H*-pyrazolo[3,4-*c*]pyridine-3-carboxamide (**64**)

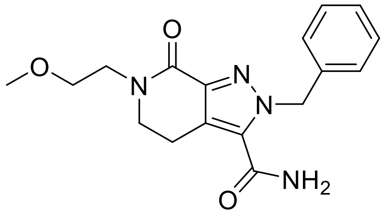

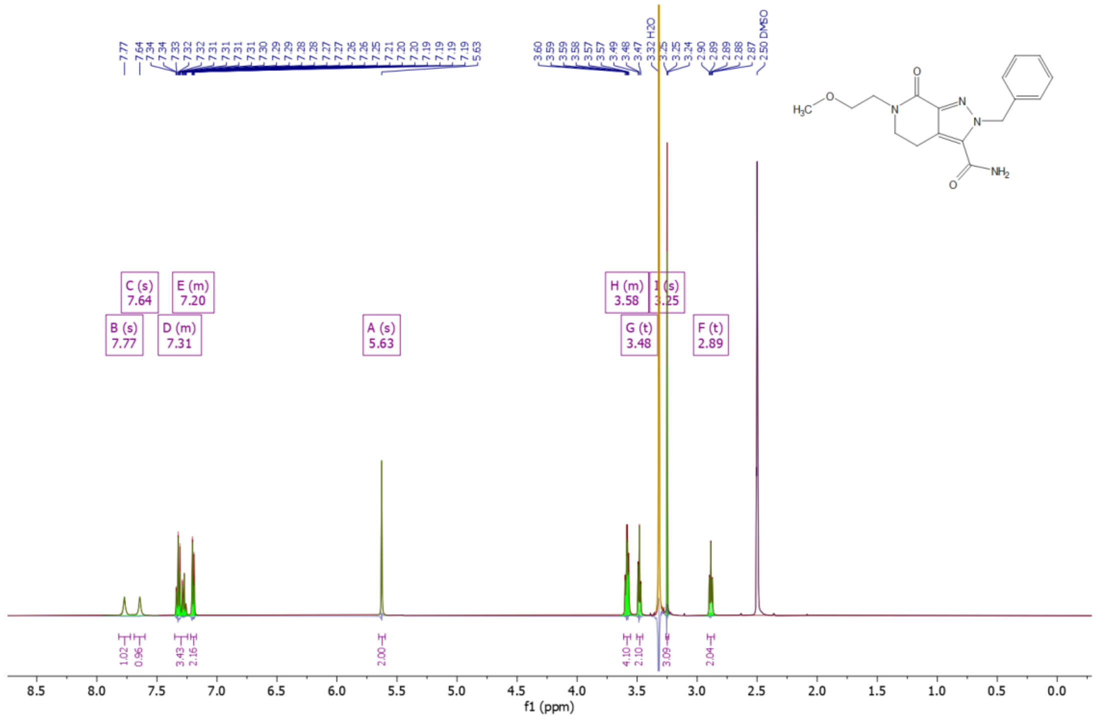

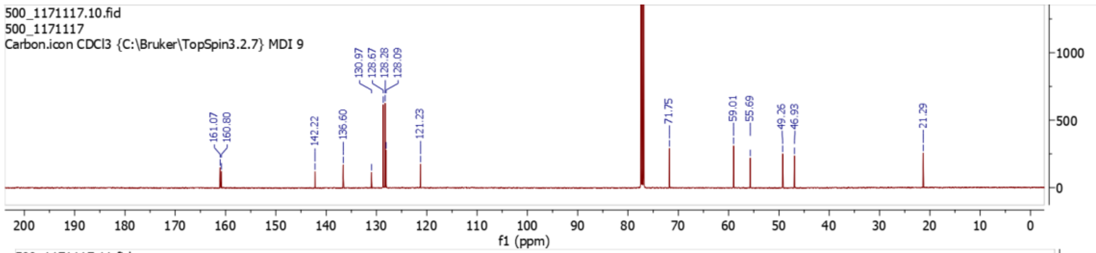

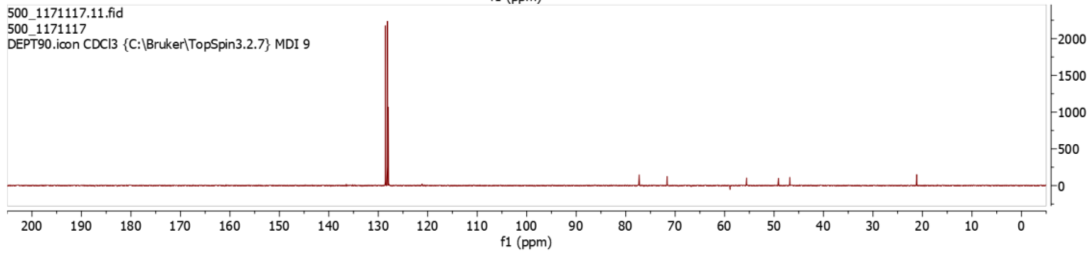

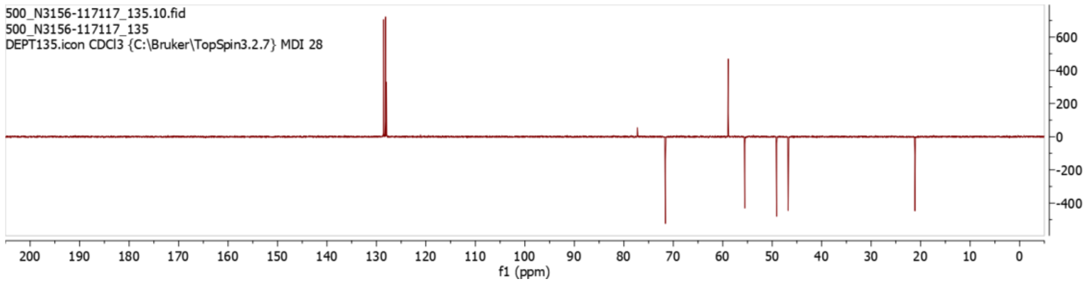

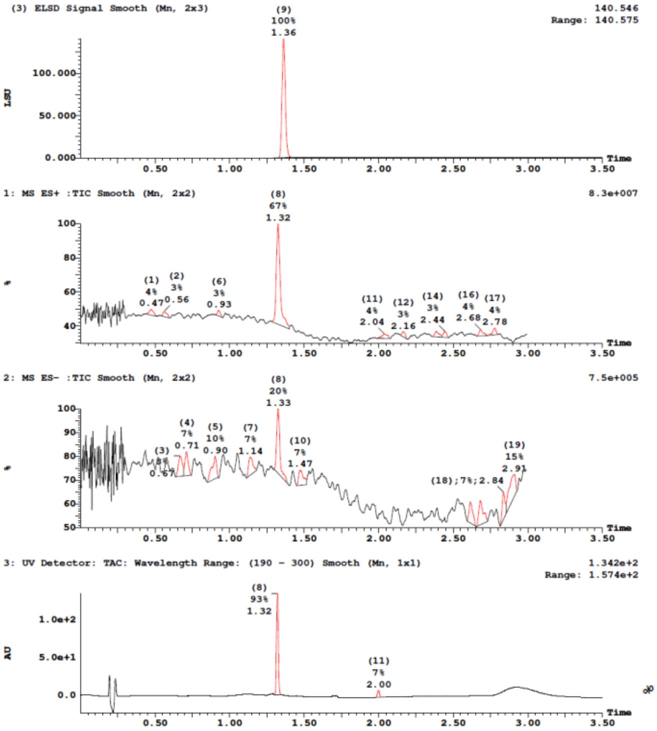

Peak ID	Compound	Time	Mass Found
8		1.32	Not Found

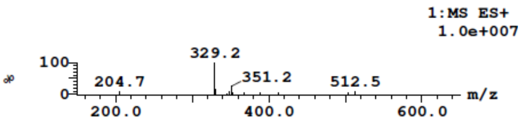

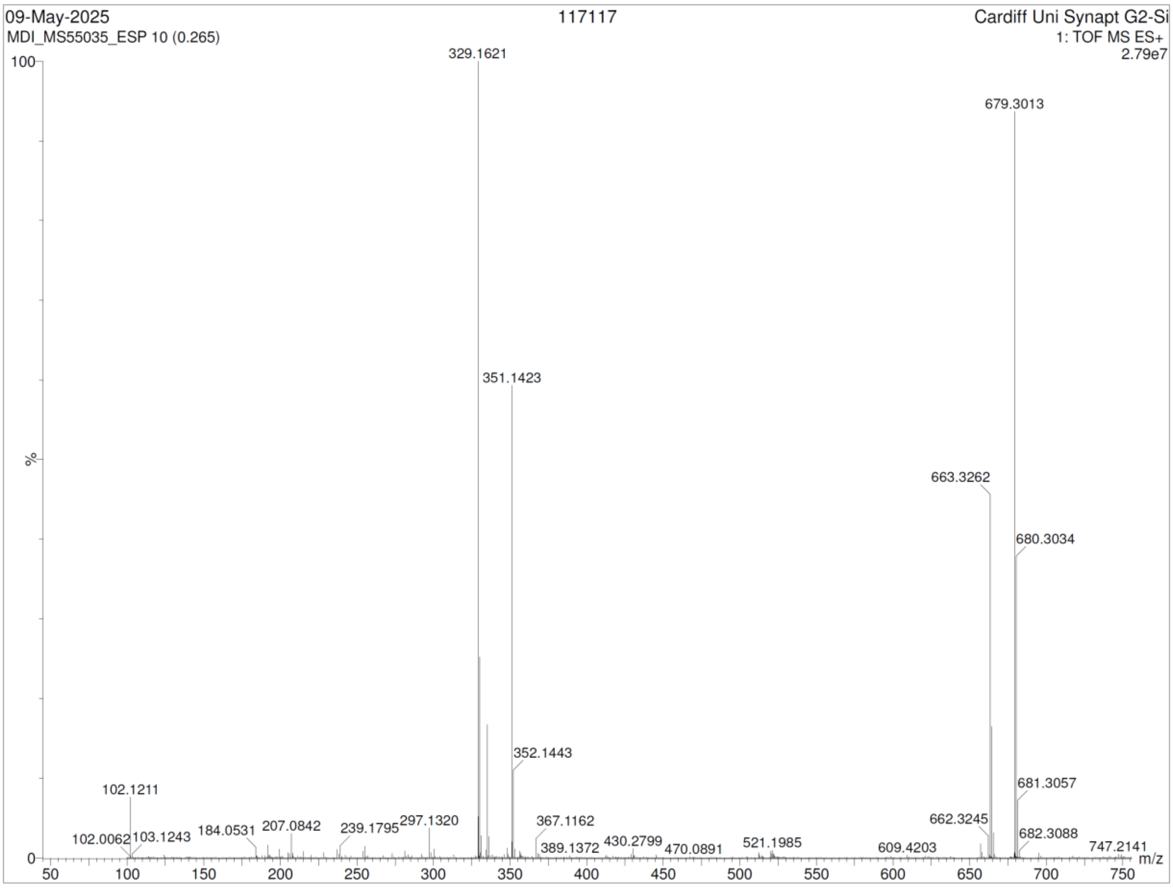

Single Mass Analysis

Tolerance = 10.0 PPM / DBE: min = -1.5, max = 50.0
Element prediction: Off
Number of isotope peaks used for i-FIT = 3

Monoisotopic Mass, Odd and Even Electron Ions
18 formula(e) evaluated with 1 results within limits (up to 50 closest results for each mass)
Elements Used:
C: 0-17 H: 0-21 N: 0-4 O: 0-3

09-May-2025 117117 Cardiff Uni Synapt G2-Si
MDI_MS55035_ESP 10 (0.265) 1: TOF MS ES+
2.79e+007

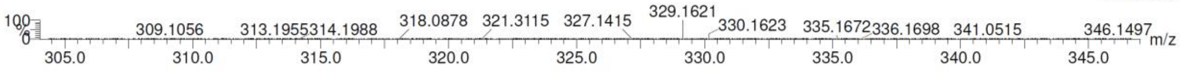

Minimum:				-1.5				
Maximum:		5.0	10.0	50.0				
Mass	Calc. Mass	mDa	PPM	DBE	i-FIT	Norm	Conf(%)	Formula
329.1621	329.1614	0.7	2.1	9.5	1252.7	n/a	n/a	C17 H21 N4 O3

2-Benzyl-6-(2-(dimethylamino)-2-oxoethyl)-7-oxo-4,5,6,7-tetrahydro-2*H*-pyrazolo[3,4-*c*]pyridine-3-carboxamide (**65**)

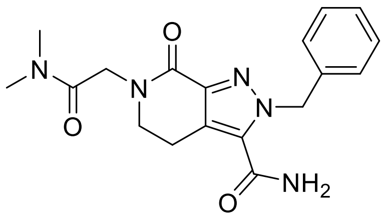

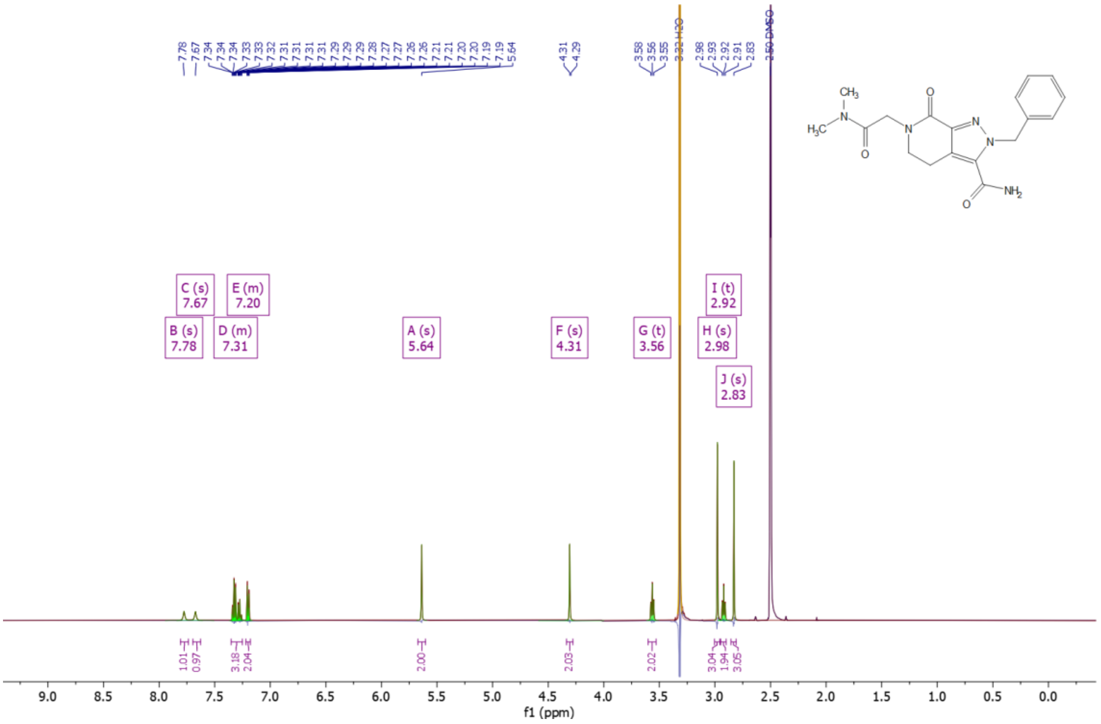

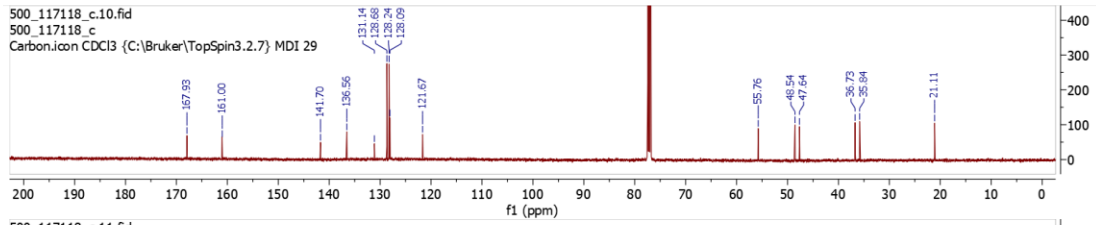

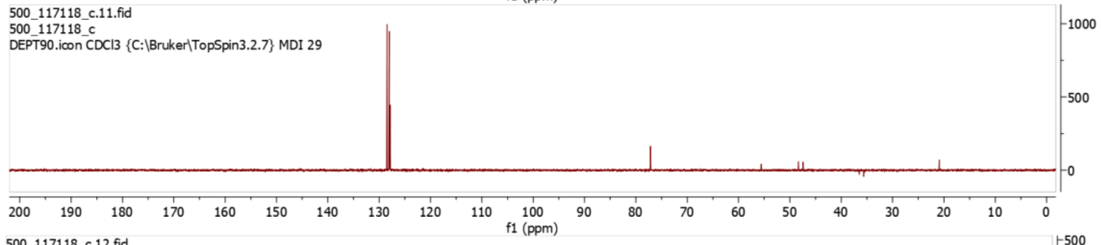

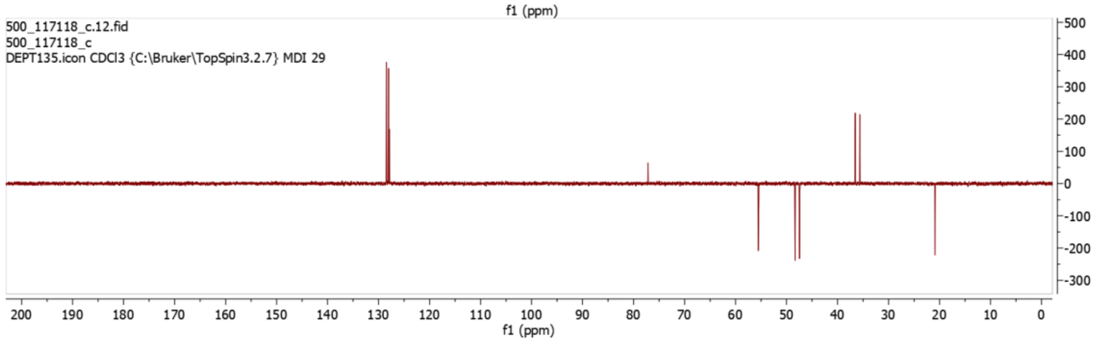

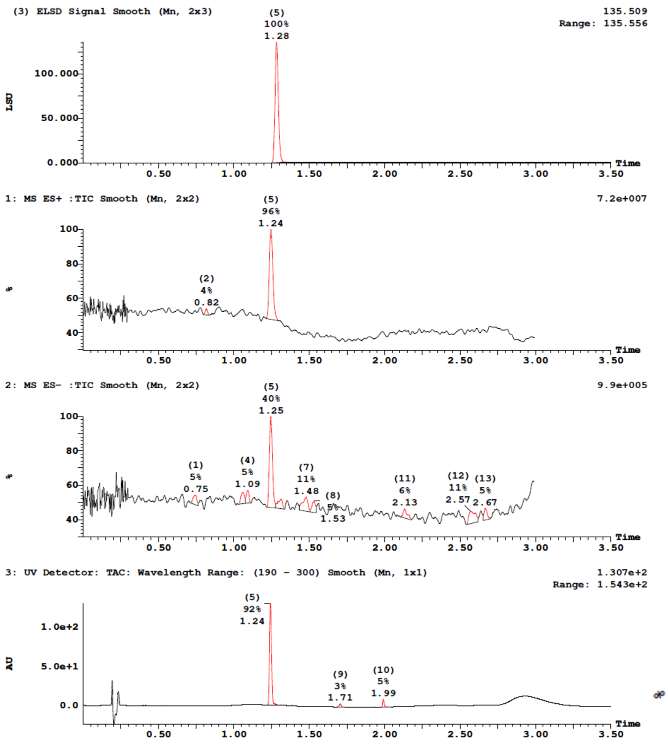

Peak ID	Compound	Time	Mass Found
5		1.24	Not Found

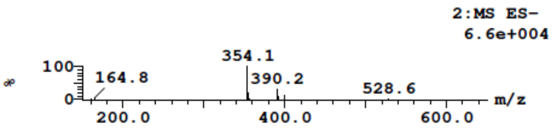

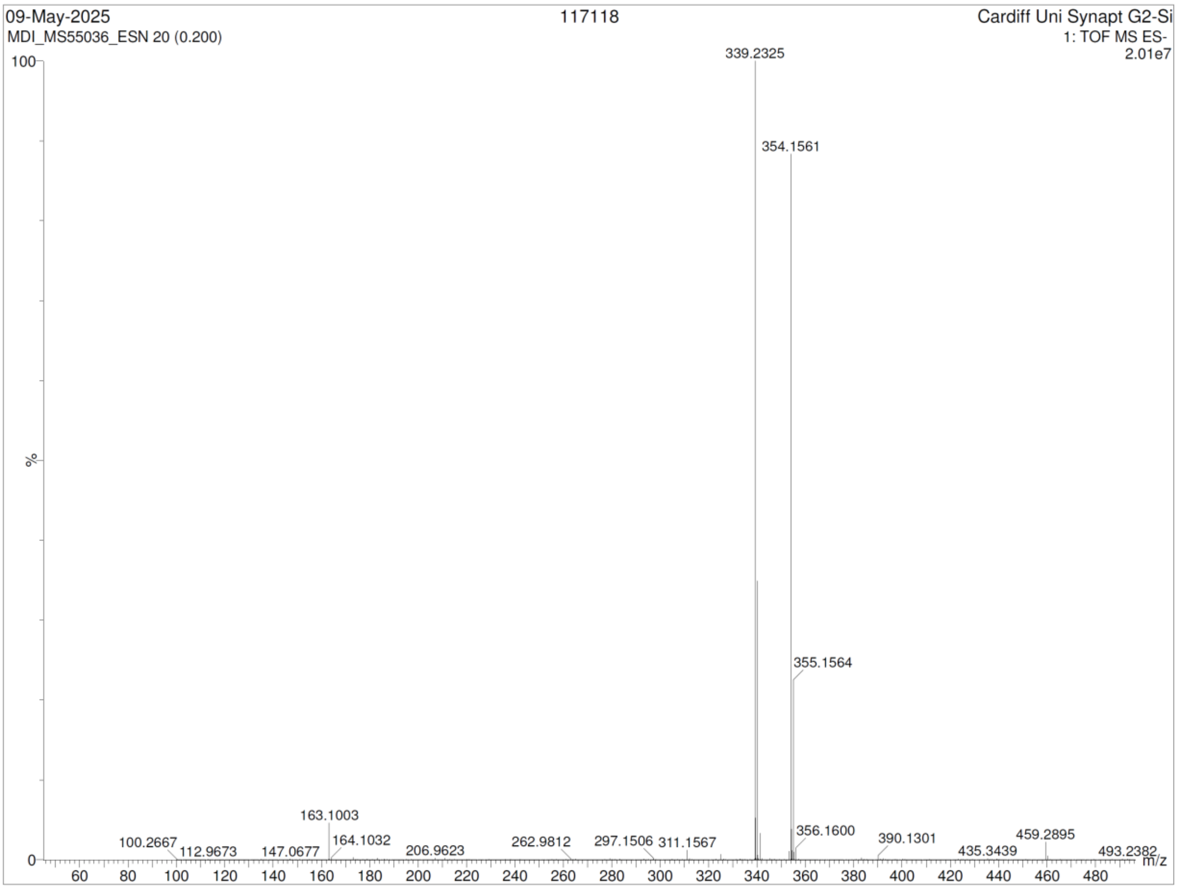

Elemental Composition Report

Single Mass Analysis

Tolerance = 10.0 PPM / DBE: min = -1.5, max = 50.0
Element prediction: Off
Number of isotope peaks used for i-FIT = 3

Monoisotopic Mass, Odd and Even Electron Ions
19 formula(e) evaluated with 1 results within limits (up to 50 closest results for each mass)
Elements Used:
C: 0-18 H: 0-20 N: 0-5 O: 0-3

09-May-2025 117118 Cardiff Uni Synapt G2-Si
MDI_MS55036_ESN 20 (0.200) 1: TOF MS ES-
2.01e+007

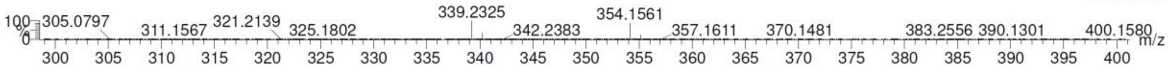

Minimum:				-1.5				
Maximum:		5.0	10.0	50.0				
Mass	Calc. Mass	mDa	PPM	DBE	i-FIT	Norm	Conf(%)	Formula
354.1561	354.1566	-0.5	-1.4	11.5	980.3	n/a	n/a	C18 H20 N5 O3

2-Benzyl-6-(3-hydroxy-1-phenylpropyl)-7-oxo-4,5,6,7-tetrahydro-2*H*-pyrazolo[3,4-*c*]pyridine-3-carboxamide (**66**)

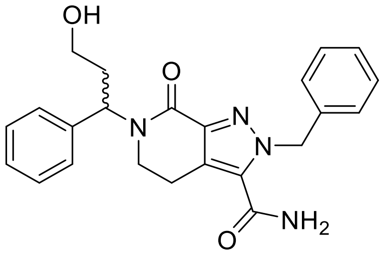

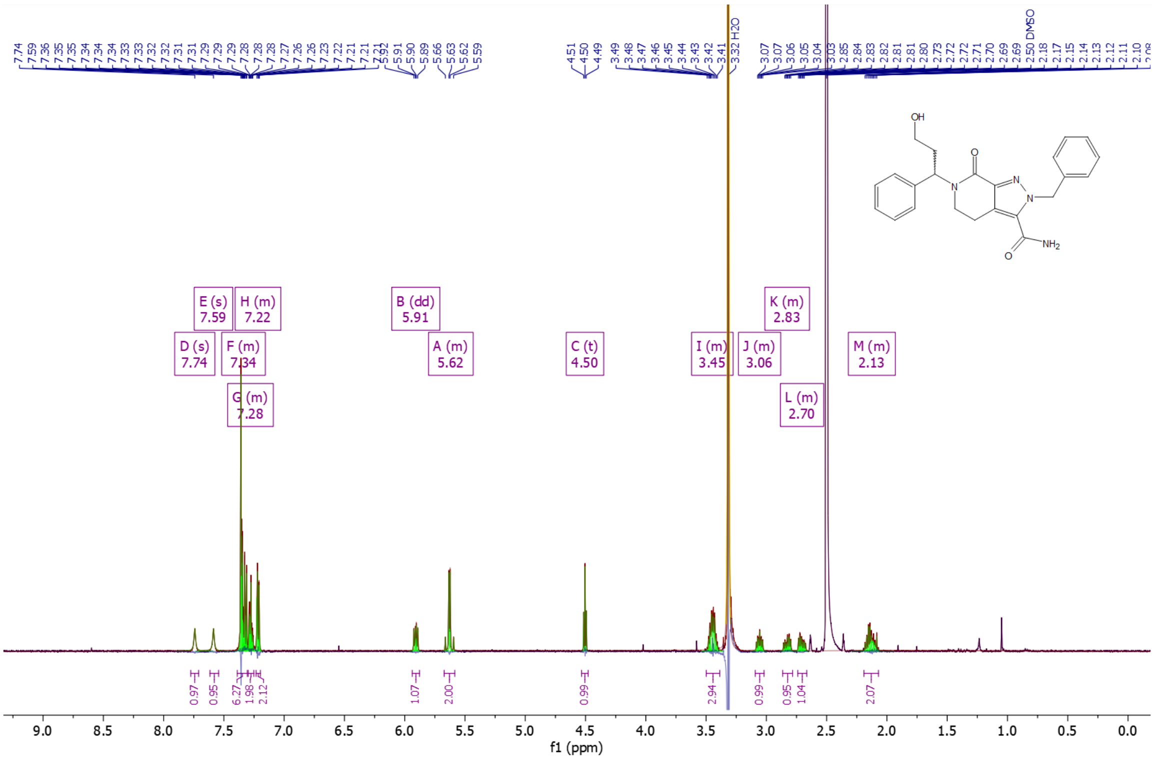

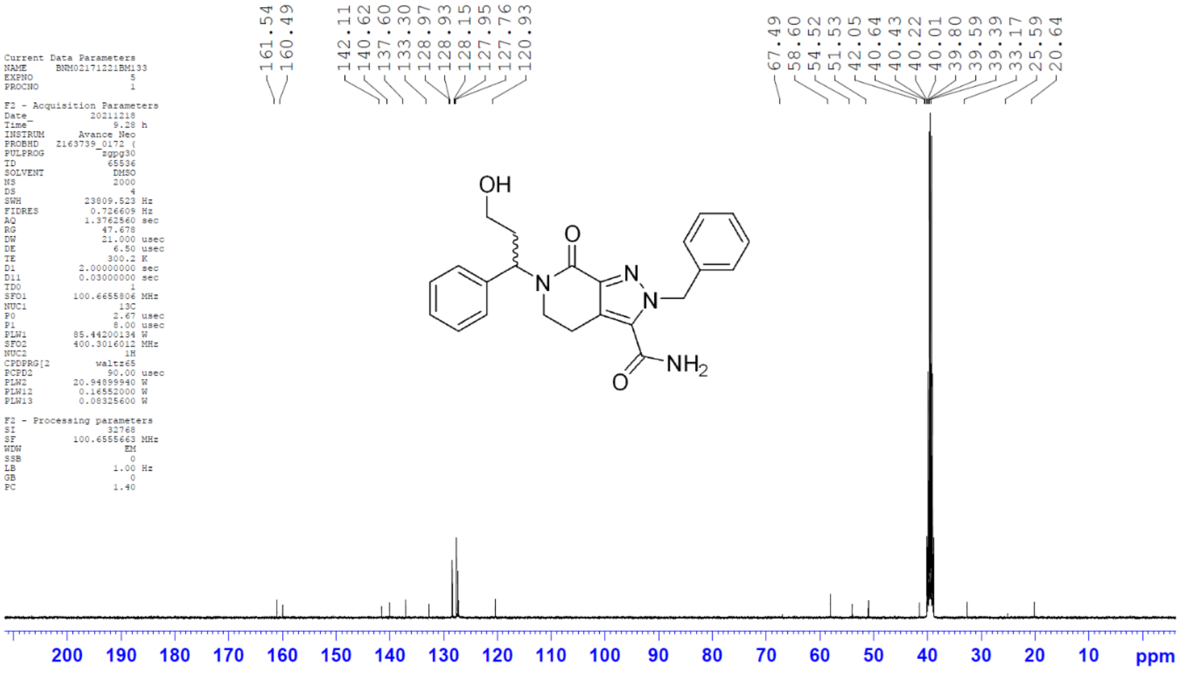

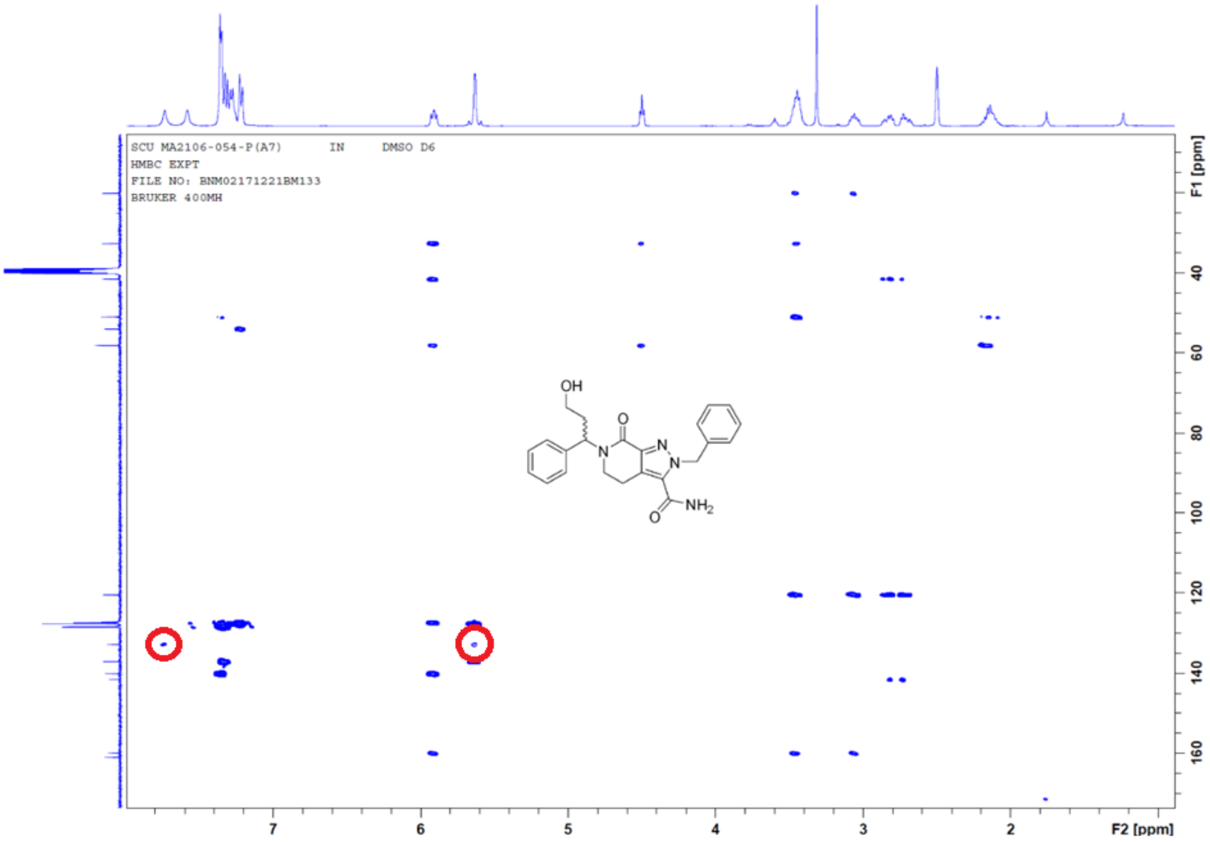
SCU MA2106-054-P(A7) IN DMSO D6
HMBC EXPT
FILE NO: BNM02171221BM133
BRUKER 400MH
OH
O
N
N
N
NH2
O
F1 [ppm]
F2 [ppm]

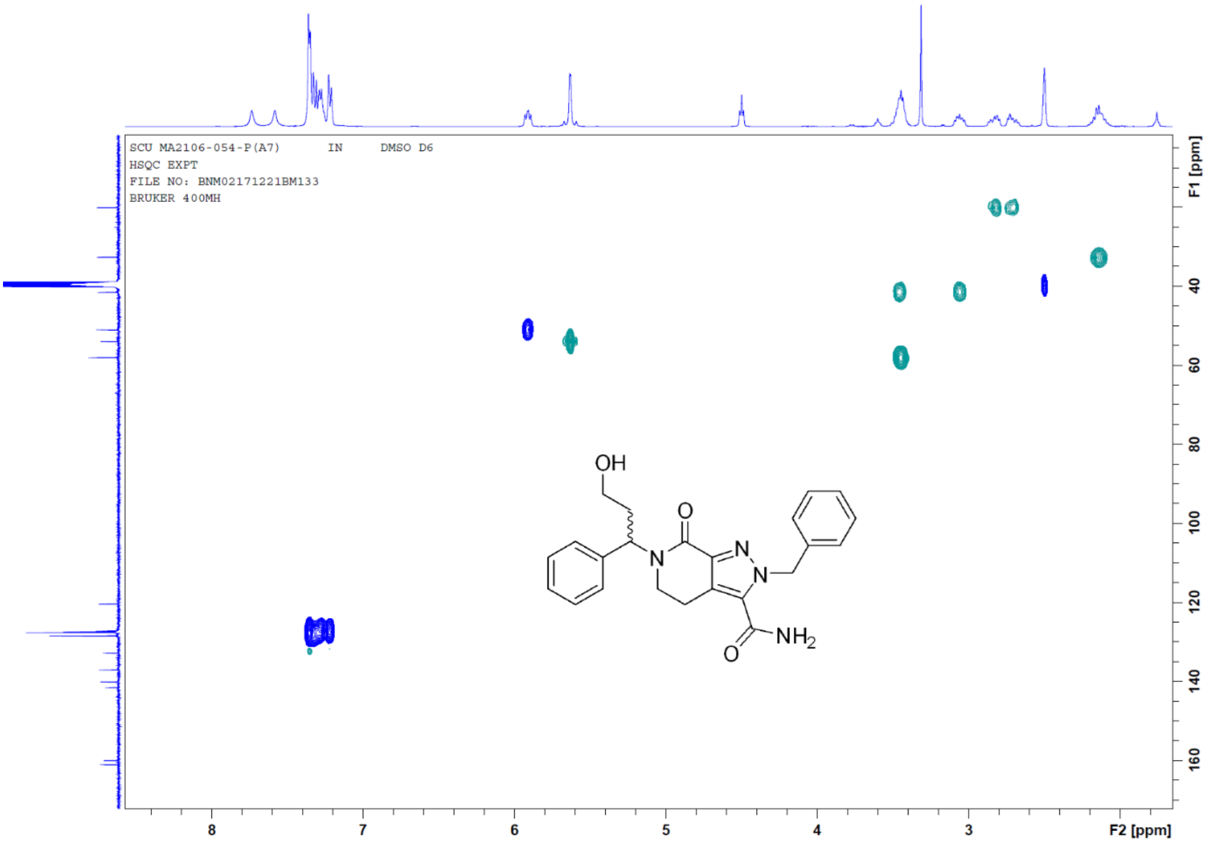
SCU MA2106-054-P(A7) IN DMSO D6
HSQC EXPT
FILE NO: BNM02171221BM133
BRUKER 400MH
OH
O
N
N
N
NH2
O
F1 [ppm]
F2 [ppm]

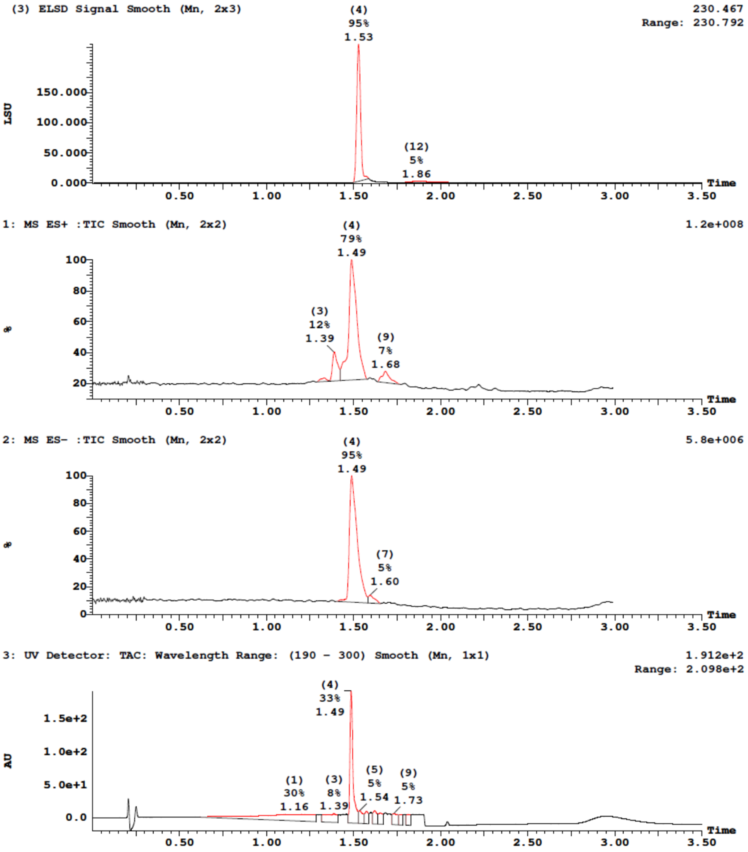

Peak ID	Compound	Time	Mass Found
4		1.49	Not Found

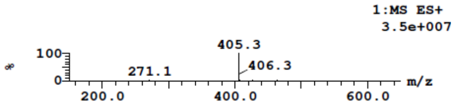

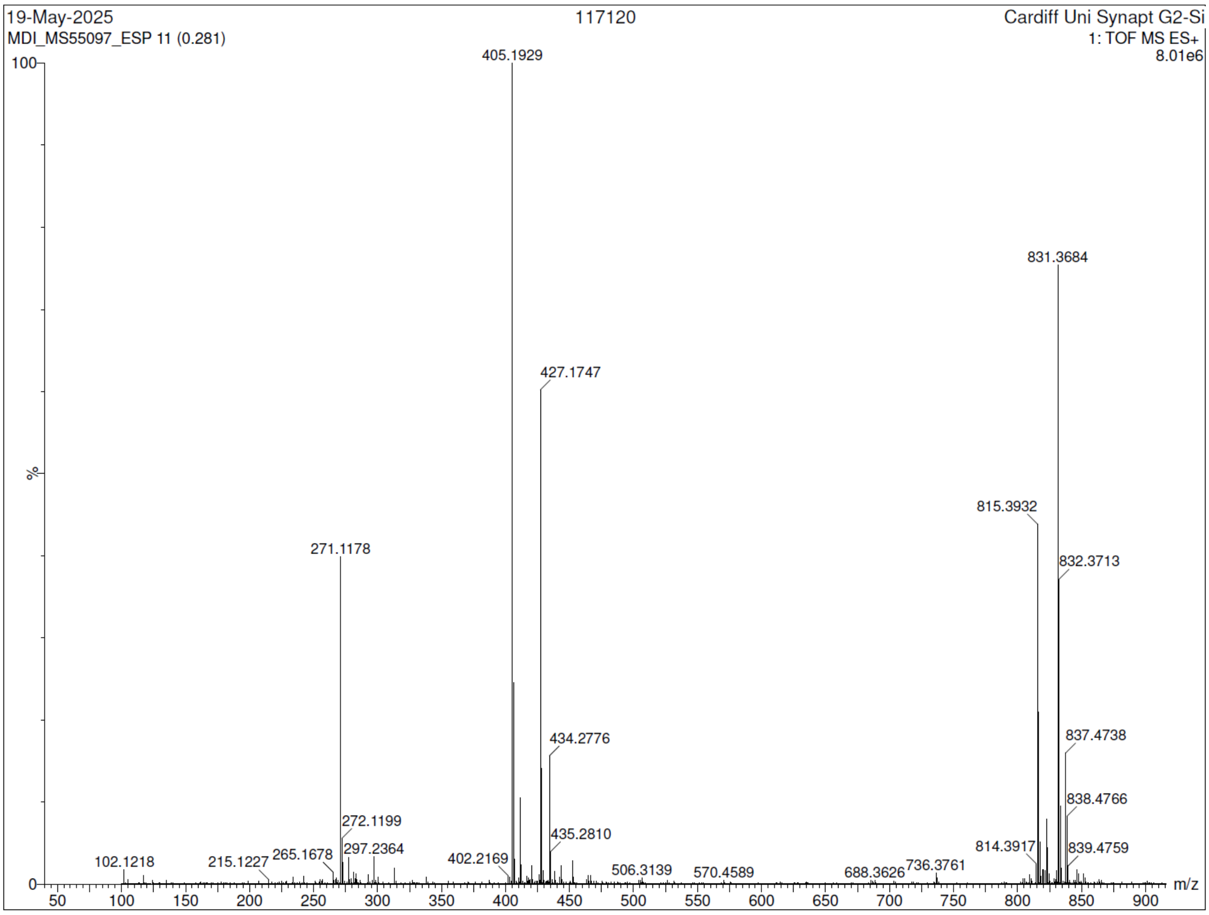

Elemental Composition Report

Single Mass Analysis

Tolerance = 50.0 PPM / DBE: min = -1.5, max = 50.0
Element prediction: Off
Number of isotope peaks used for i-FIT = 3

Monoisotopic Mass, Odd and Even Electron Ions
15 formula(e) evaluated with 1 results within limits (up to 50 closest results for each mass)
Elements Used:
C: 0-23 H: 0-25 N: 0-4 O: 0-3

19-May-2025 117120 Cardiff Uni Synapt G2-Si
MDI_MS55097_ESP 11 (0.281) 1: TOF MS ES+
8.01e+006

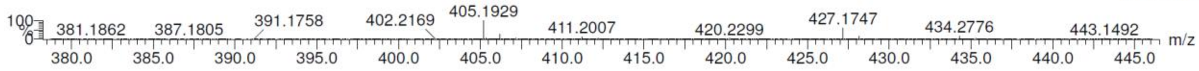

Minimum:				-1.5				
Maximum:		5.0	50.0	50.0				
Mass	Calc. Mass	mDa	PPM	DBE	i-FIT	Norm	Conf(%)	Formula
405.1929	405.1927	0.2	0.5	13.5	1055.5	n/a	n/a	C23 H25 N4 O3

2-Benzyl-7-oxo-6-phenethyl-4,5,6,7-tetrahydro-2*H*-pyrazolo[3,4-*c*]pyridine-3-carboxylic acid (**67**)

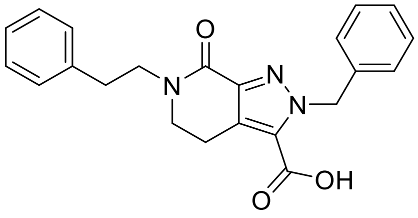

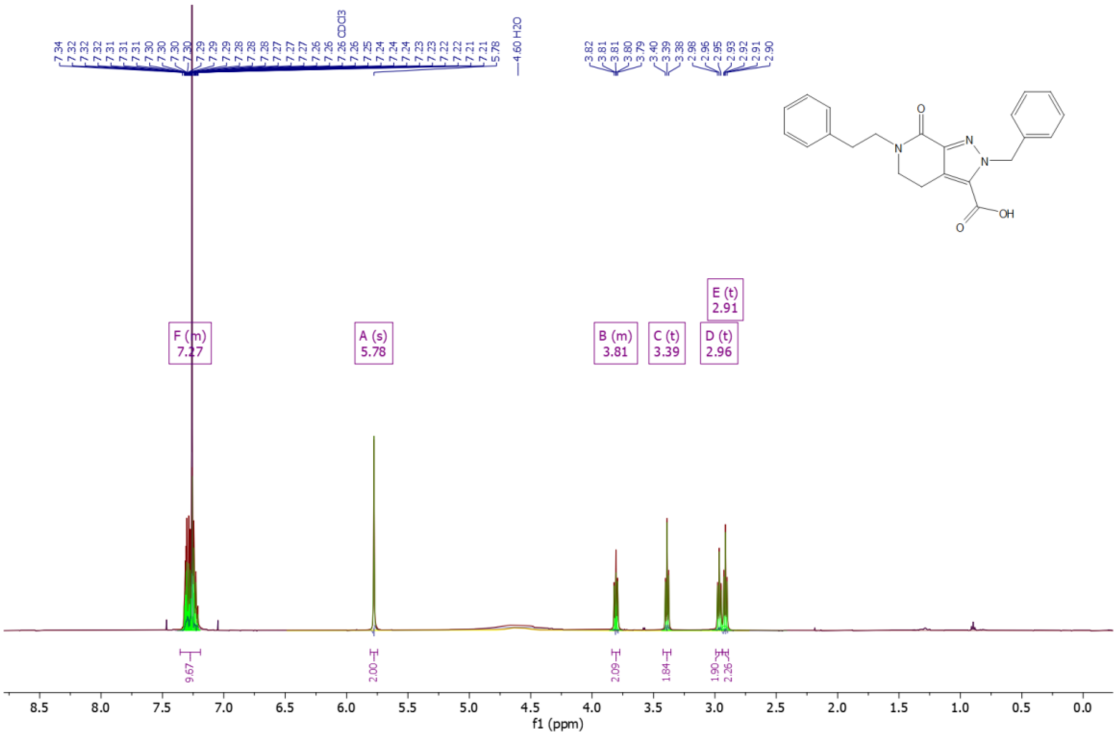

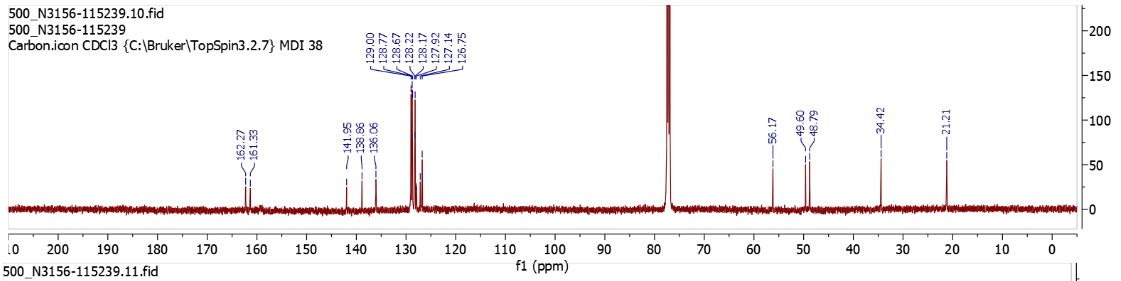

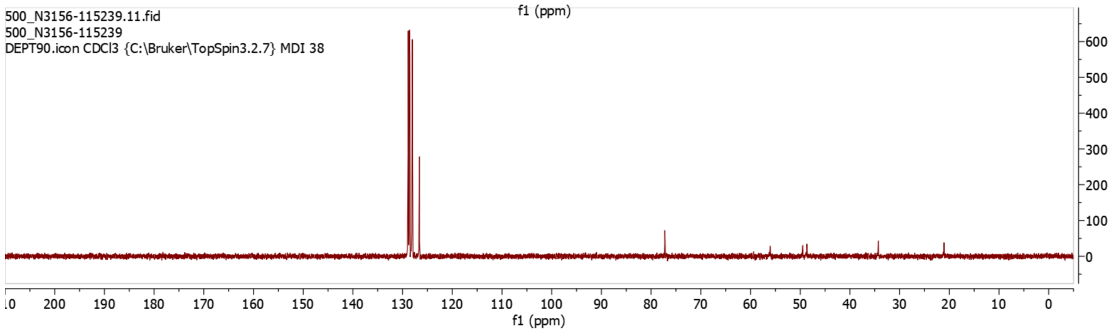

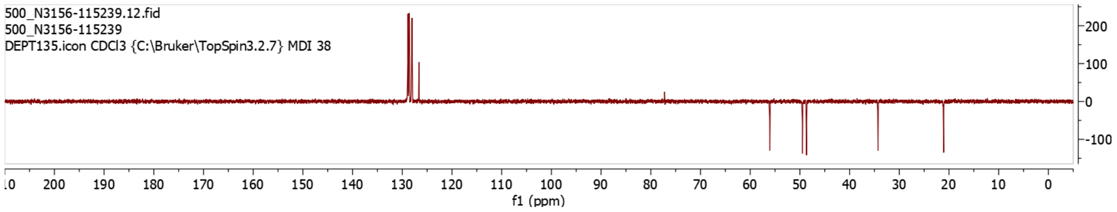

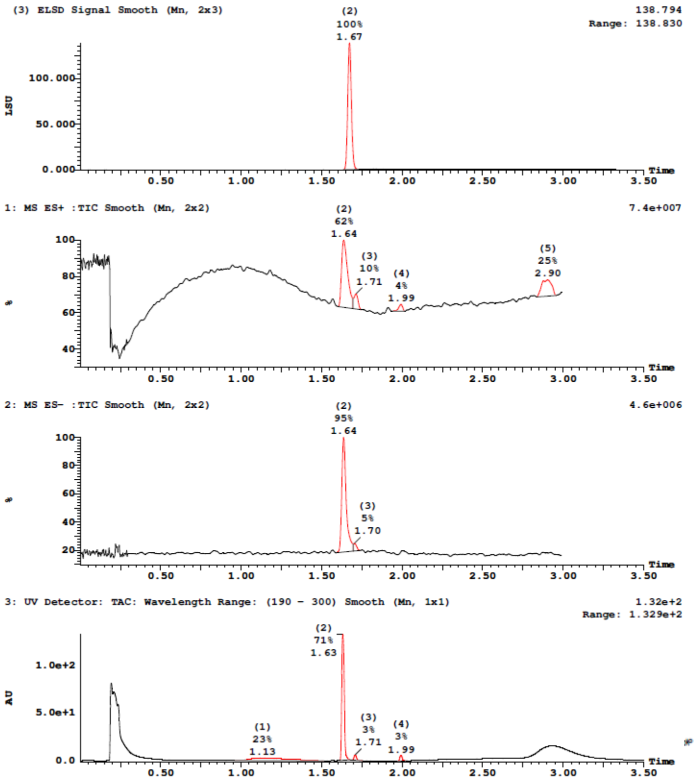

Peak ID	Compound	Time	Mass Found
2		1.64	Not Found

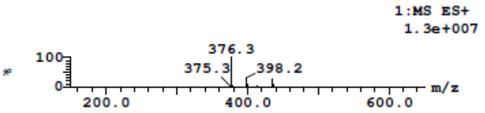

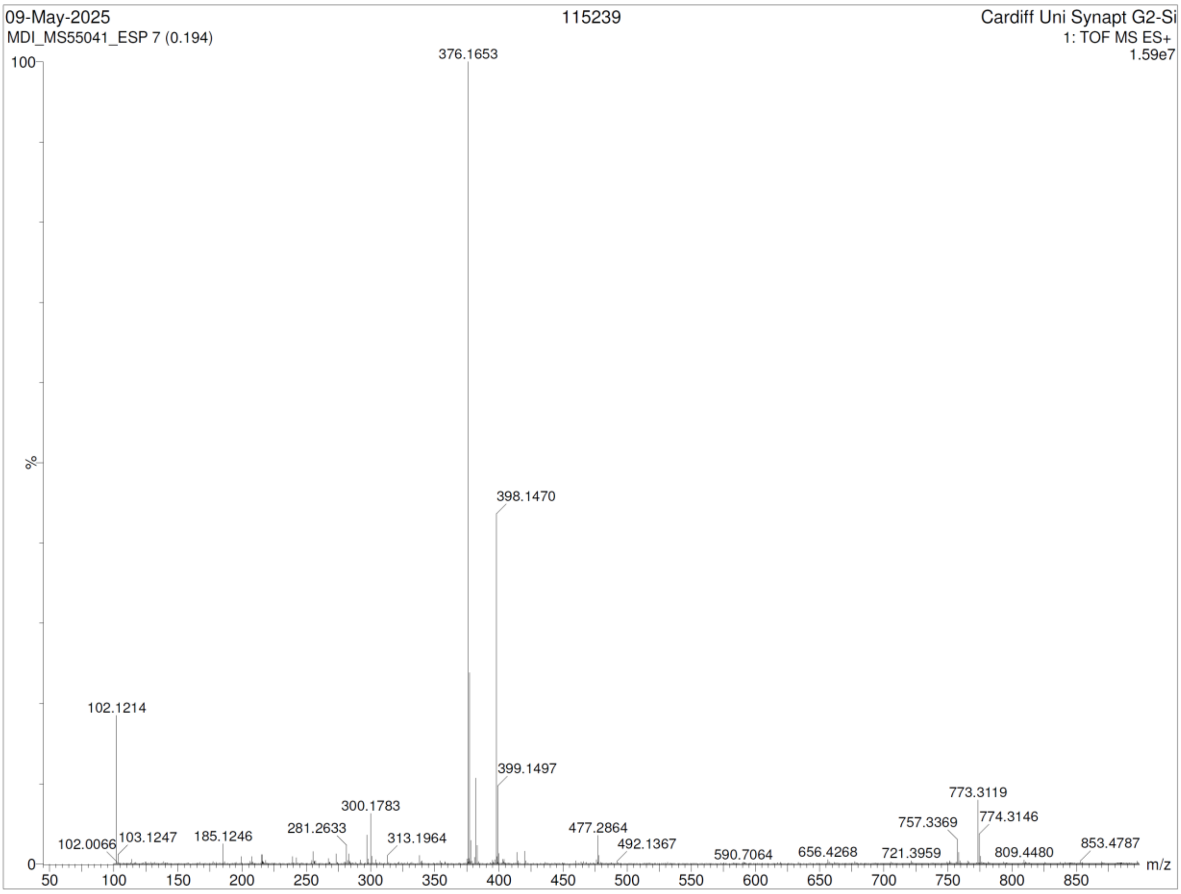

Elemental Composition Report

Single Mass Analysis

Tolerance = 10.0 PPM / DBE: min = -1.5, max = 50.0
Element prediction: Off
Number of isotope peaks used for i-FIT = 3

Monoisotopic Mass, Odd and Even Electron Ions
11 formula(e) evaluated with 1 results within limits (up to 50 closest results for each mass)
Elements Used:
C: 0-22 H: 0-22 N: 0-3 O: 0-3

09-May-2025 115239 Cardiff Uni Synapt G2-Si
MDI_MS55041_ESP 7 (0.194) 1: TOF MS ES+
1.59e+007

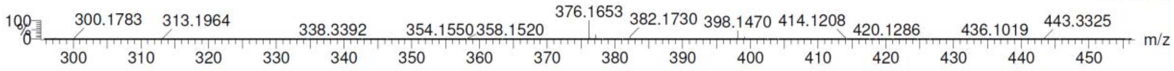

Minimum:				-1.5				
Maximum:		5.0	10.0	50.0				
Mass	Calc. Mass	mDa	PPM	DBE	i-FIT	Norm	Conf(%)	Formula
376.1653	376.1661	-0.8	-2.1	13.5	1094.1	n/a	n/a	C22 H22 N3 O3

2-Benzyl-6-(cyclopropyl(phenyl)methyl)-7-oxo-4,5,6,7-tetrahydro-2*H*-pyrazolo[3,4-*c*]pyridine-3-carboxamide (**68**)

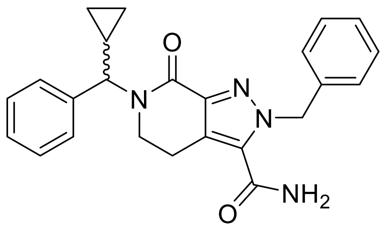

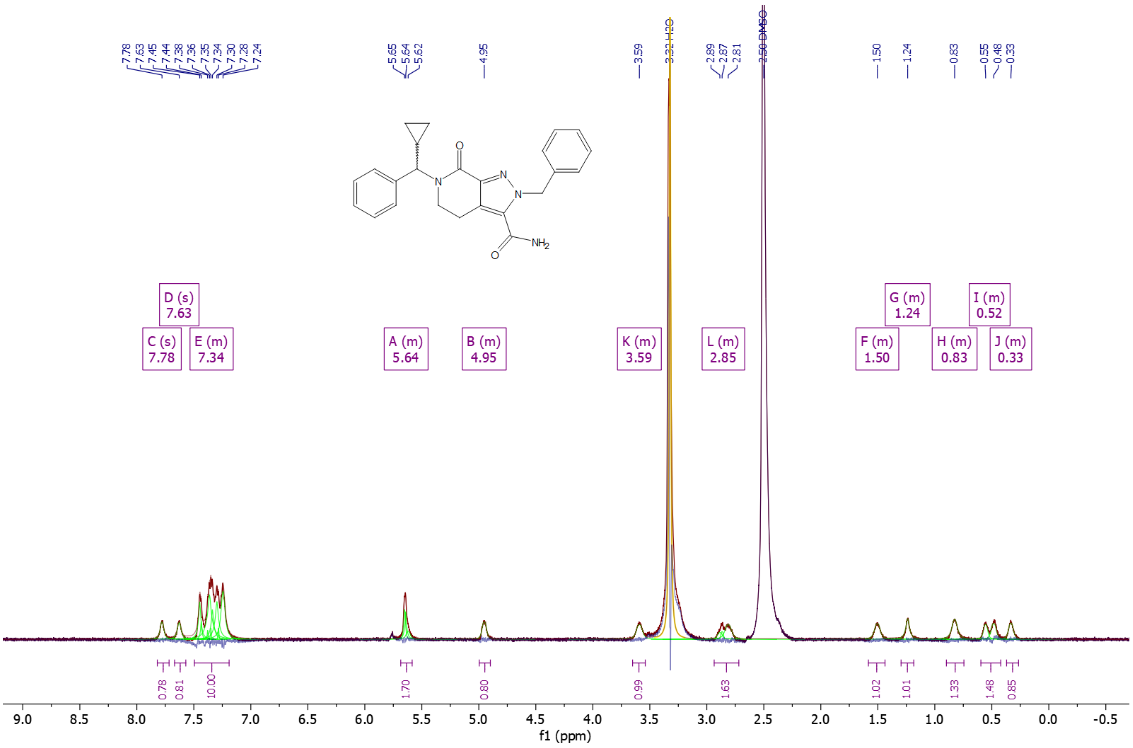

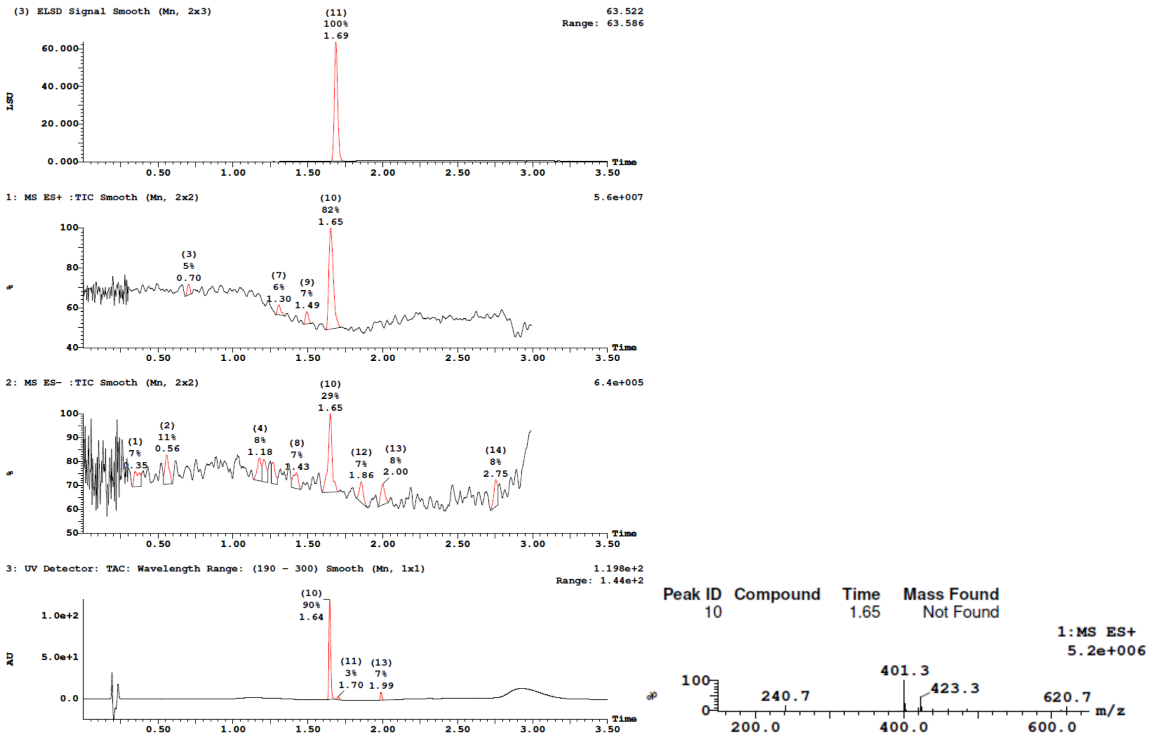

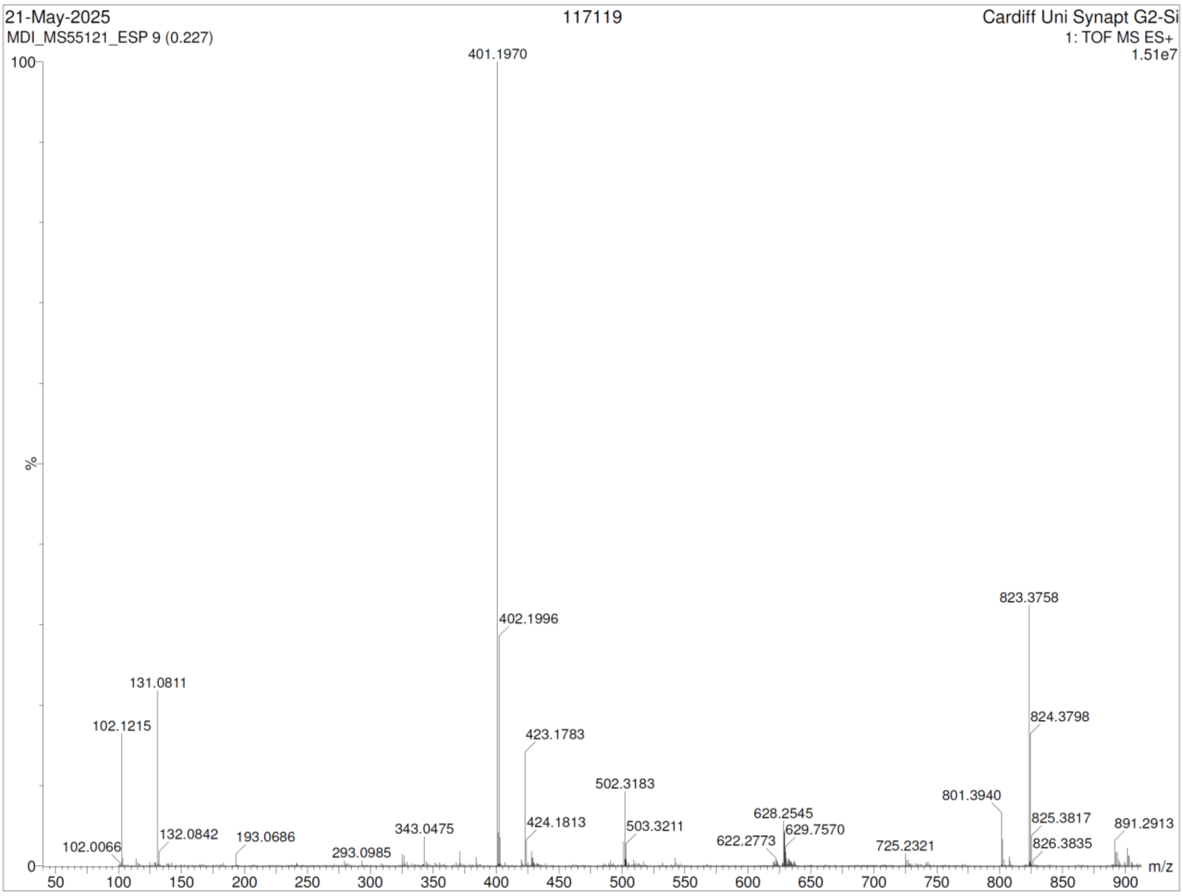

Elemental Composition Report

Page 1

Single Mass Analysis

Tolerance = 50.0 PPM / DBE: min = -1.5, max = 50.0
Element prediction: Off
Number of isotope peaks used for i-FIT = 3

Monoisotopic Mass, Odd and Even Electron Ions
10 formula(e) evaluated with 1 results within limits (up to 50 closest results for each mass)
Elements Used:
C: 0-24 H: 0-25 N: 0-4 O: 0-2

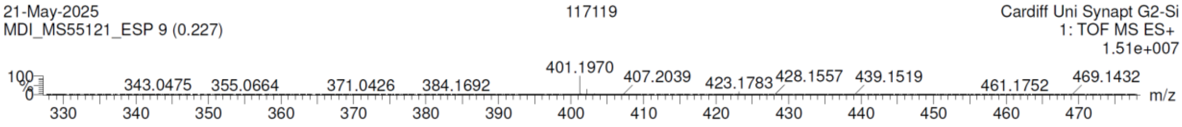

Minimum:				-1.5				
Maximum:		5.0	50.0	50.0				
Mass	Calc. Mass	mDa	PPM	DBE	i-FIT	Norm	Conf(%)	Formula
401.1970	401.1978	-0.8	-2.0	14.5	1115.7	n/a	n/a	C24 H25 N4 O2

(*R*)-2-Benzyl-6-(cyclopropyl(phenyl)methyl)-7-oxo-4,5,6,7-tetrahydro-2*H*-pyrazolo[3,4-*c*]pyridine-3-carboxamide (**69**, MDI-117740)

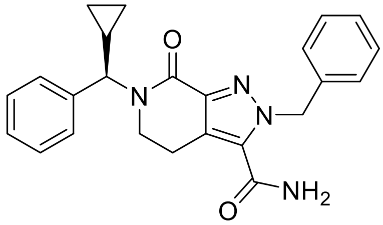

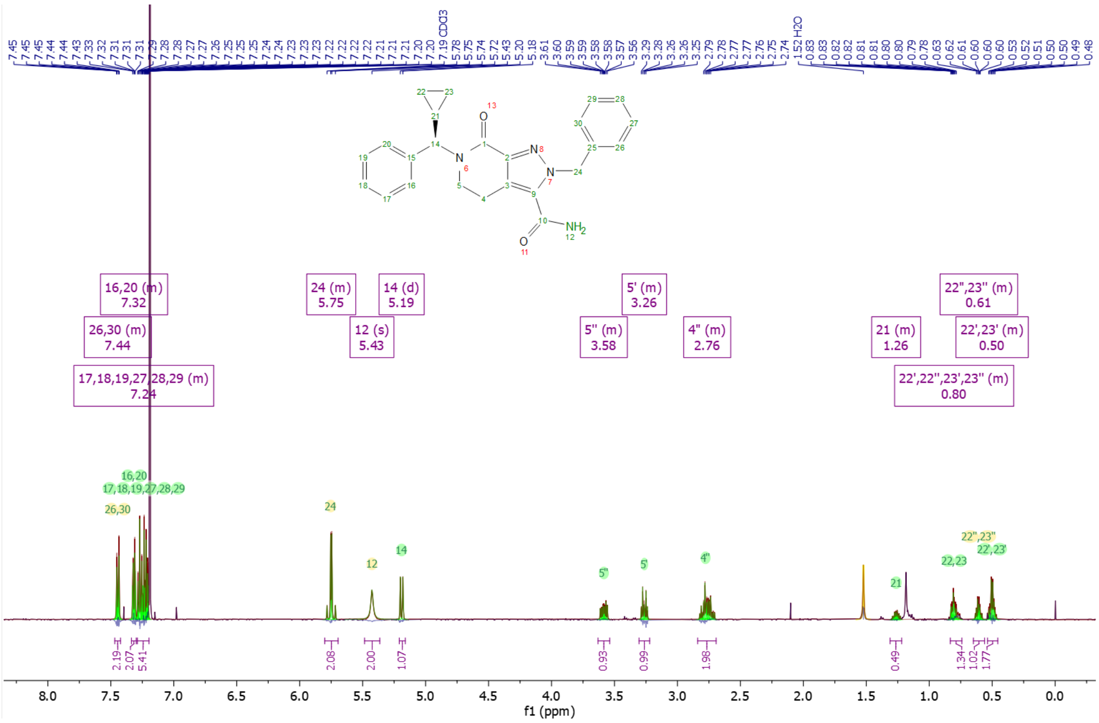

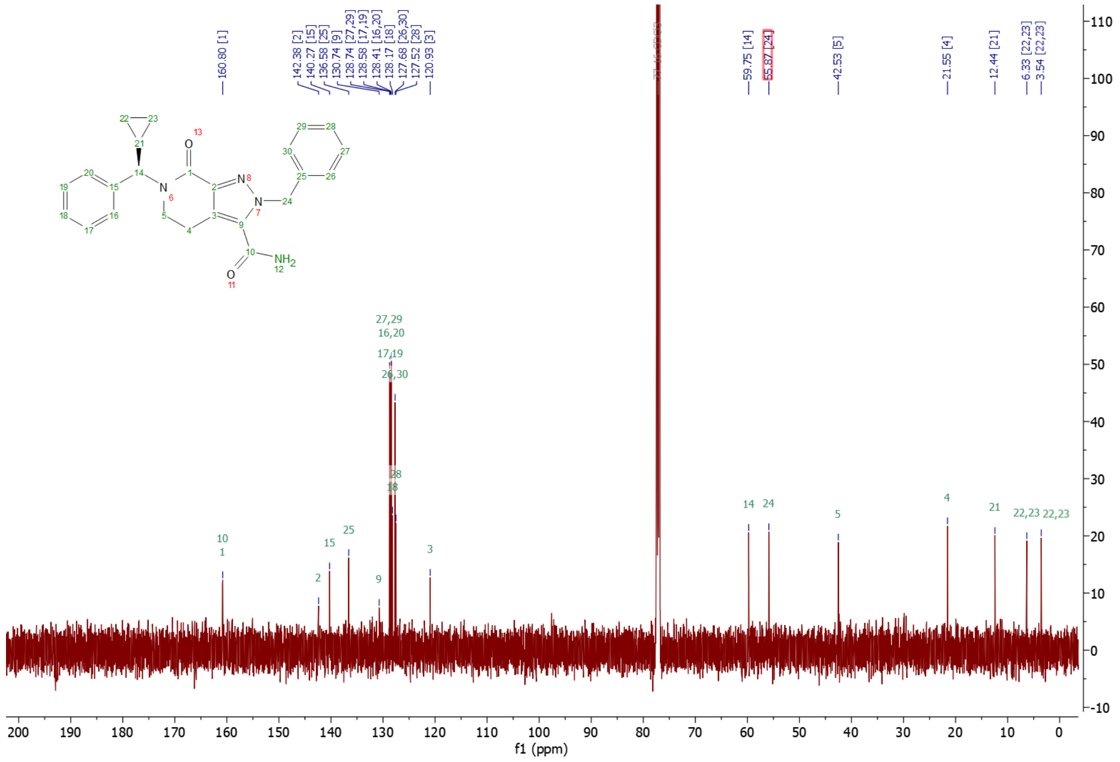

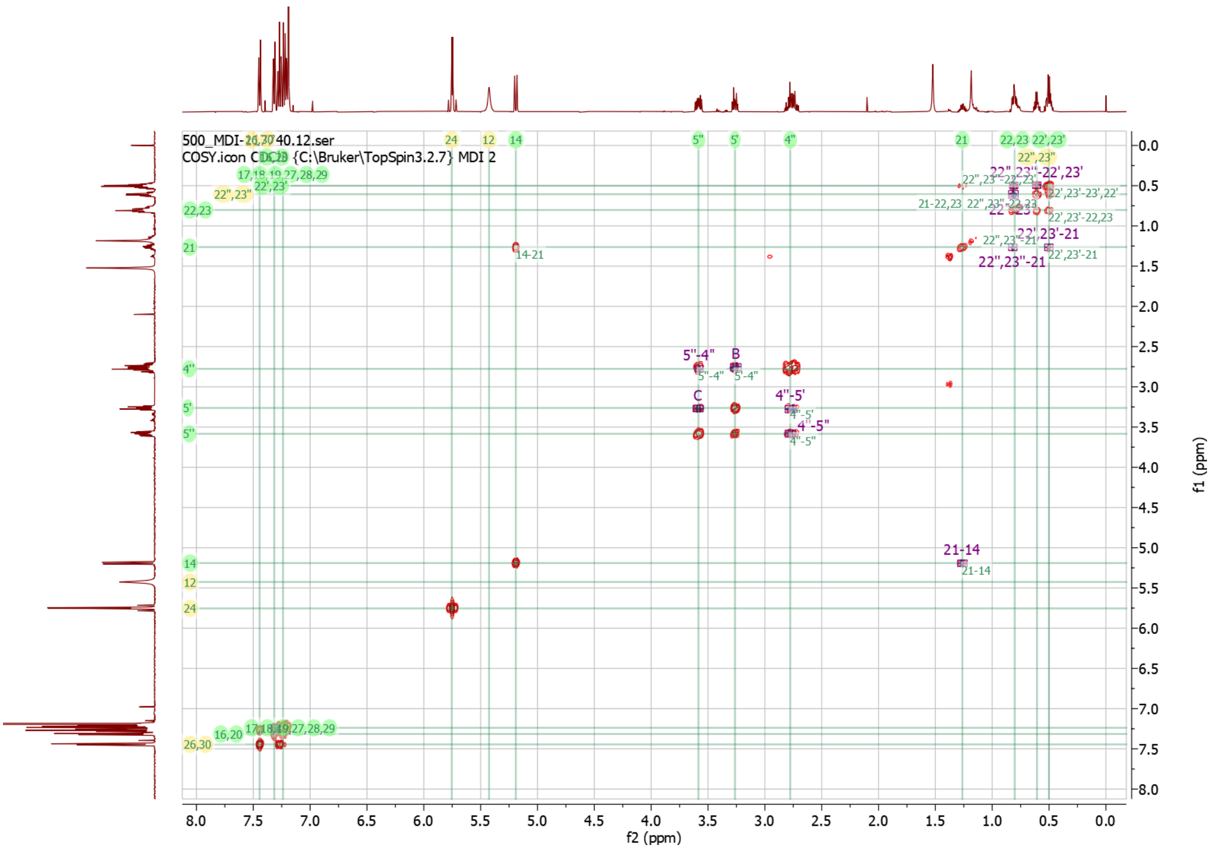
500_MDI-
40.12.ser
COSY.icon
{C:\Bruker\TopSpin3.2.7} MDI 2
f2 (ppm)
f1 (ppm)

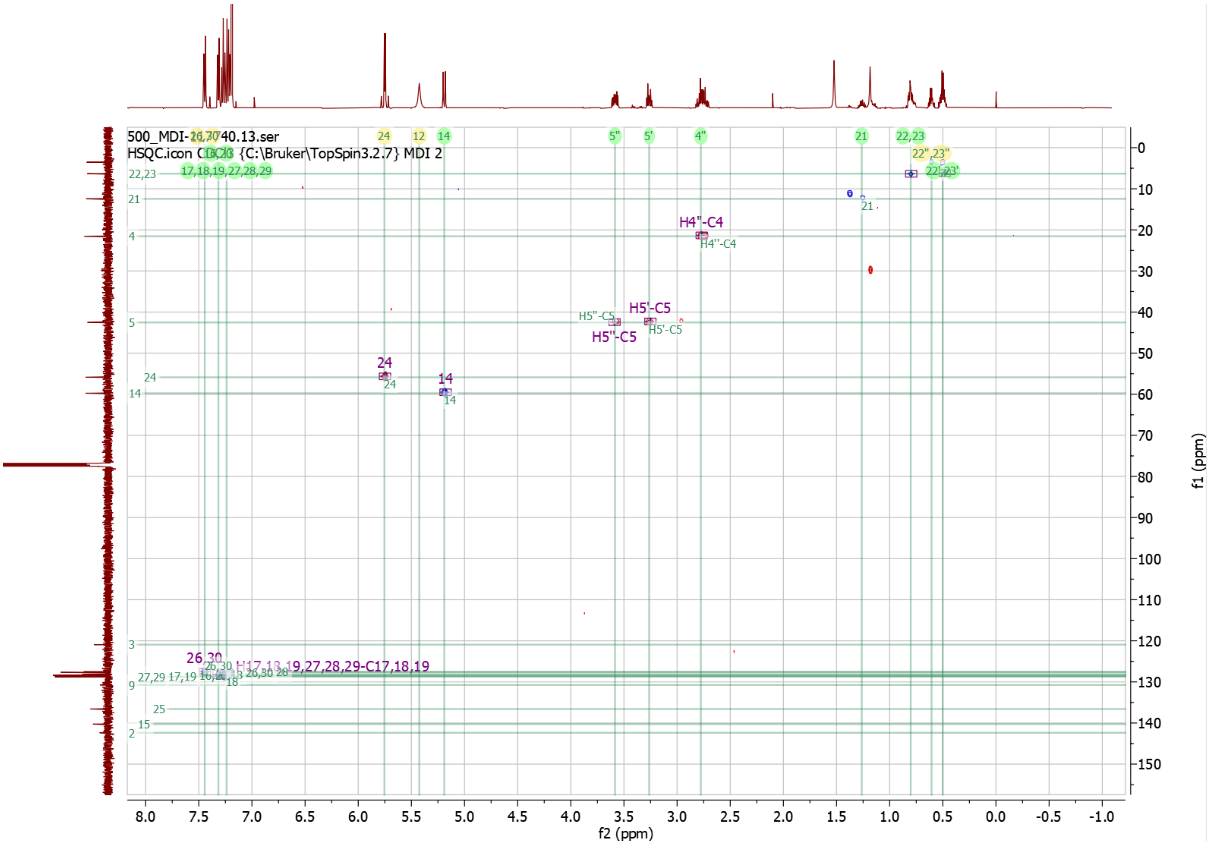
500_MDI-
40.13.ser
HSQC.icon
{C:\Bruker\TopSpin3.2.7} MDI 2
H4"-C4
H5'-C5
H5"-C5
f2 (ppm)
f1 (ppm)

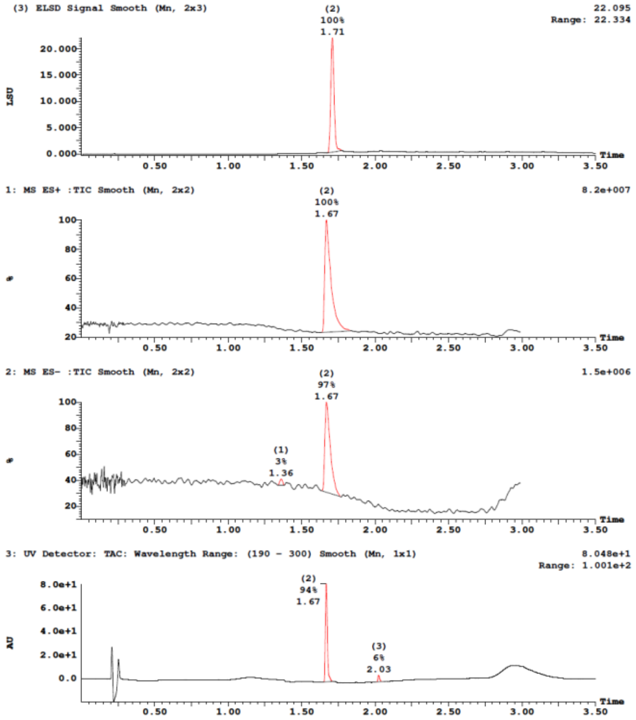

Peak ID	Compound	Time	Mass Found
2		1.67	Not Found

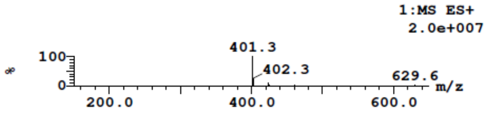

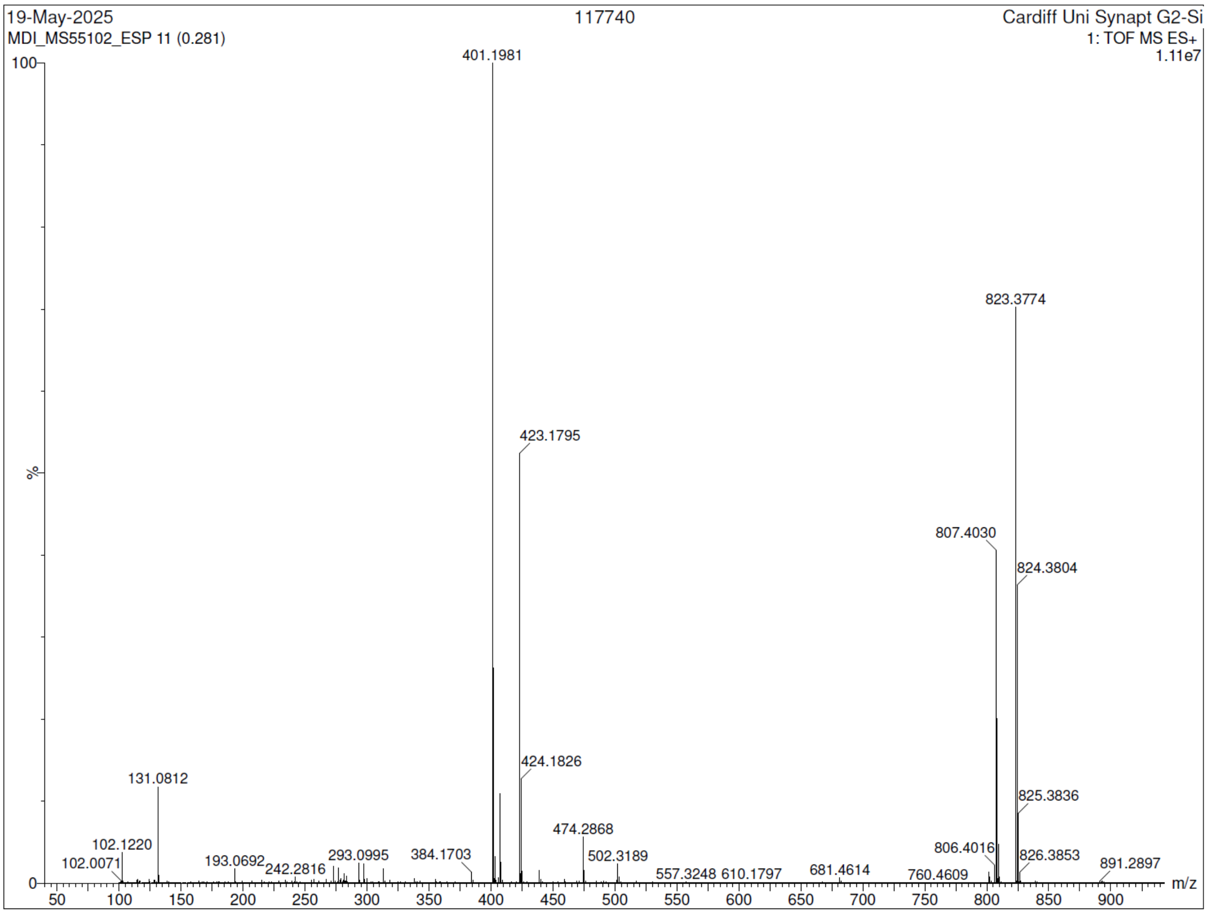

Single Mass Analysis

Tolerance = 50.0 PPM / DBE: min = -1.5, max = 50.0
Element prediction: Off
Number of isotope peaks used for i-FIT = 3

Monoisotopic Mass, Odd and Even Electron Ions
10 formula(e) evaluated with 1 results within limits (up to 50 closest results for each mass)
Elements Used:
C: 0-24 H: 0-25 N: 0-4 O: 0-2

19-May-2025 117740 Cardiff Uni Synapt G2-Si
MDI_MS55102_ESP 11 (0.281) 1: TOF MS ES+
1.11e+007

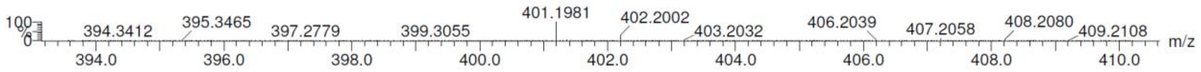

Minimum:				-1.5				
Maximum:		5.0	50.0	50.0				
Mass	Calc. Mass	mDa	PPM	DBE	i-FIT	Norm	Conf(%)	Formula
401.1981	401.1978	0.3	0.7	14.5	1159.2	n/a	n/a	C24 H25 N4 O2

(*S*)-2-Benzyl-6-(cyclopropyl(phenyl)methyl)-7-oxo-4,5,6,7-tetrahydro-2*H*-pyrazolo[3,4-*c*]pyridine-3-carboxamide (**70**)

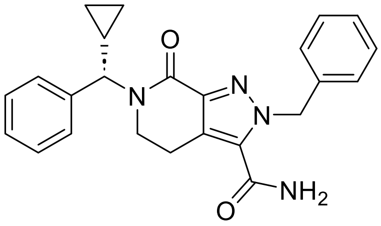

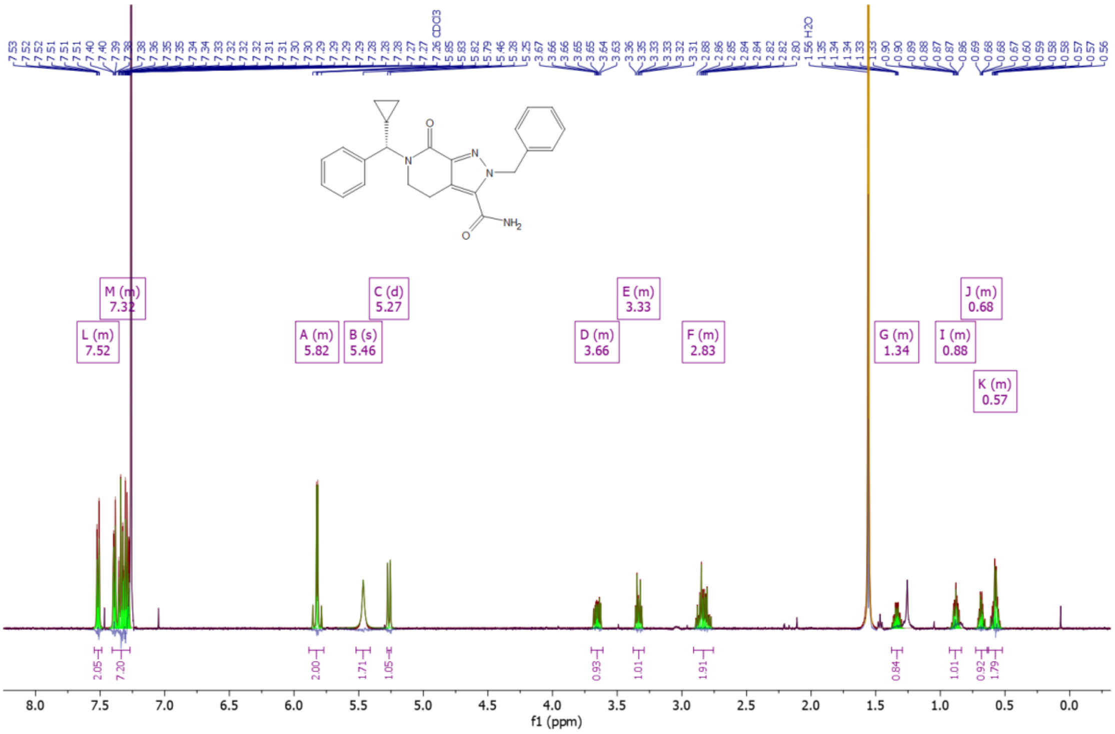

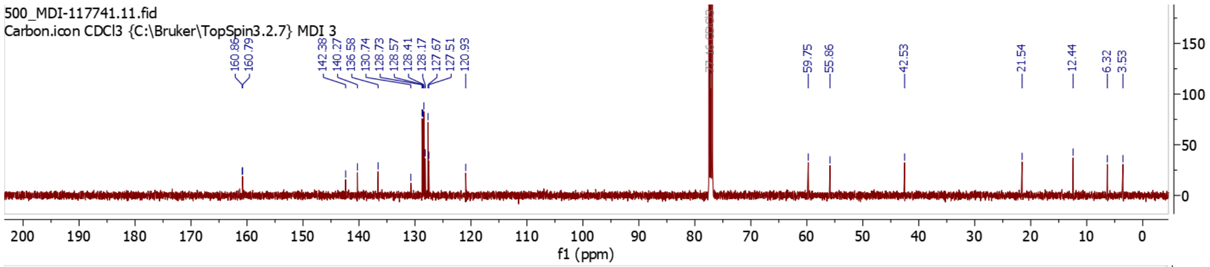

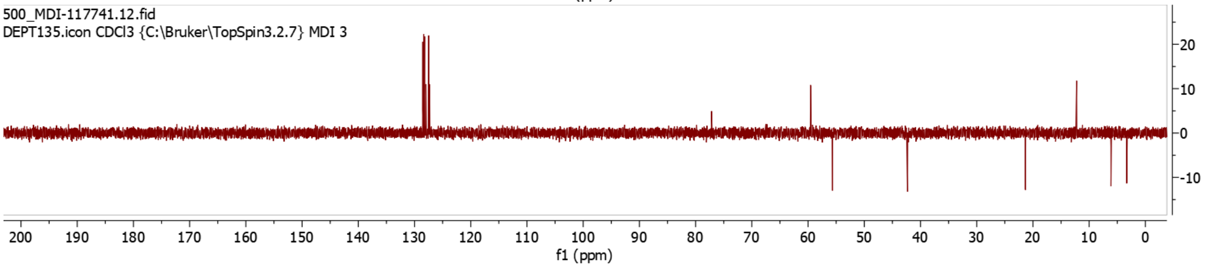

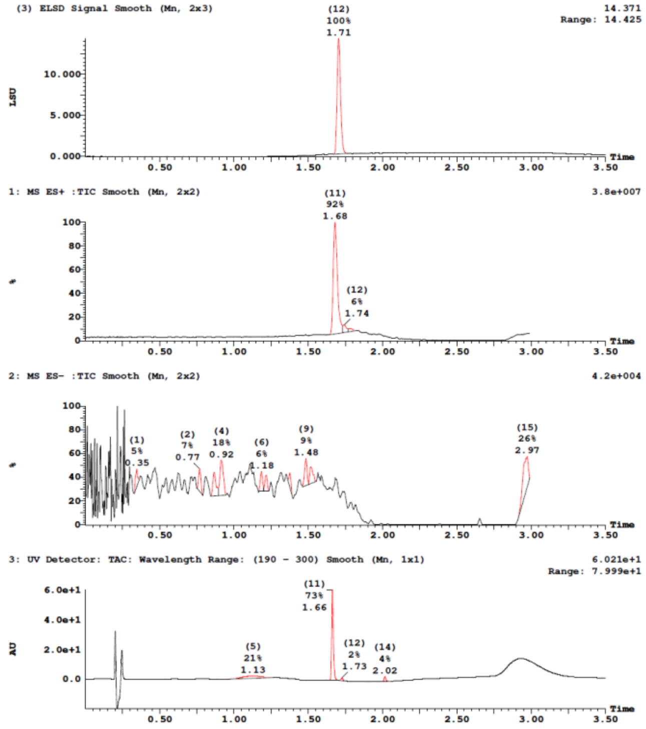

Peak ID	Compound	Time	Mass Found
11		1.68	Not Found

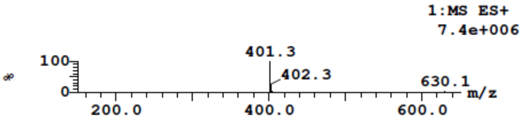

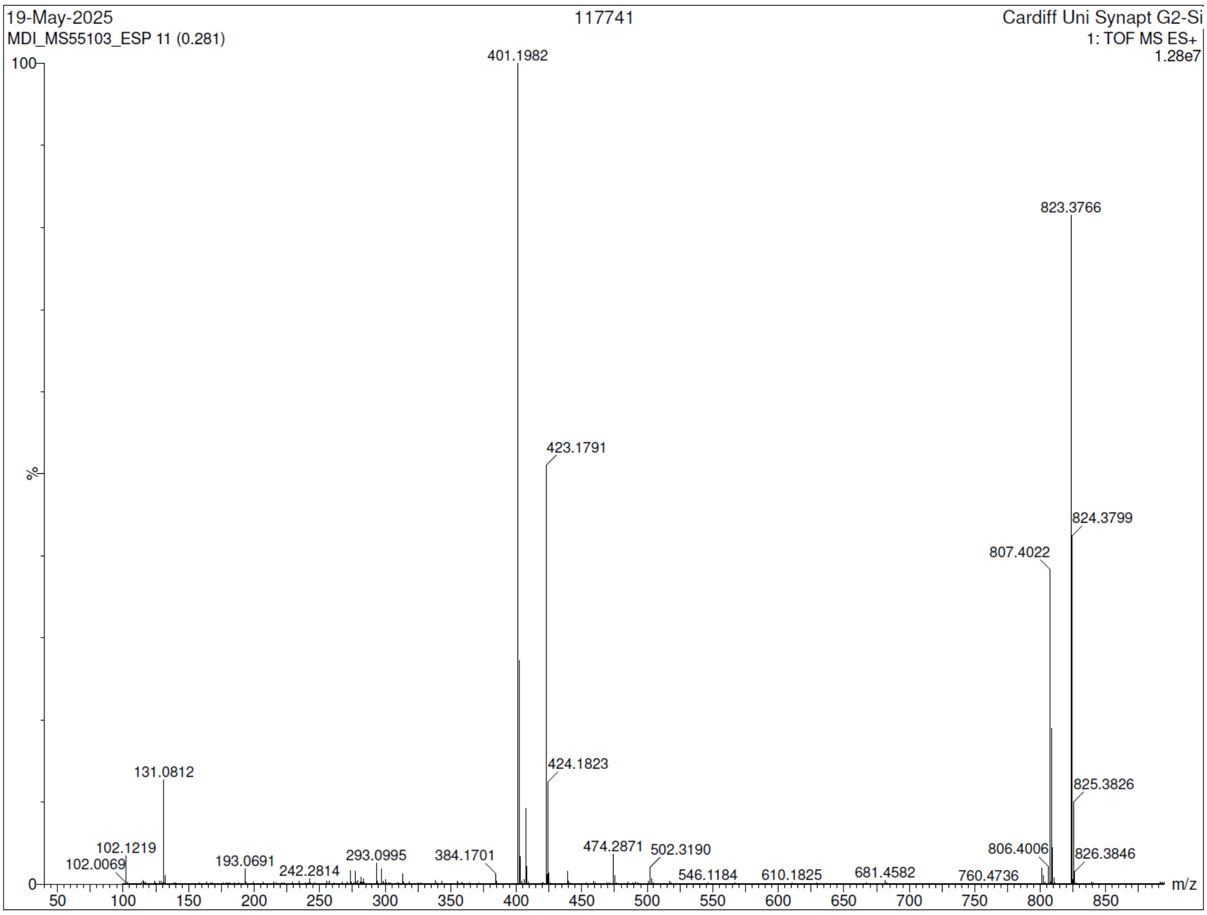

Elemental Composition Report

Single Mass Analysis
Tolerance = 50.0 PPM / DBE: min = -1.5, max = 50.0
Element prediction: Off
Number of isotope peaks used for i-FIT = 3

Monoisotopic Mass, Odd and Even Electron Ions
10 formula(e) evaluated with 1 results within limits (up to 50 closest results for each mass)
Elements Used:
C: 0-24 H: 0-25 N: 0-4 O: 0-2

19-May-2025 117741 Cardiff Uni Synapt G2-Si
MDI_MS55103_ESP 11 (0.281) 1: TOF MS ES+
1.28e+007

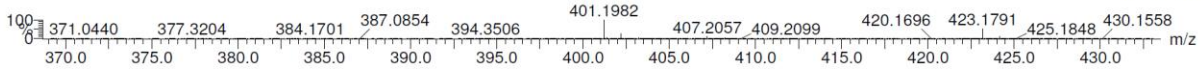

Mass	Calc. Mass	mDa	PPM	DBE	i-FIT	Norm	Conf(%)	Formula
Minimum:				-1.5				
Maximum:		5.0	50.0	50.0				
401.1982	401.1978	0.4	1.0	14.5	1102.5	n/a	n/a	C24 H25 N4 O2

2-Benzyl-6-(cyclopropyl(2-((2-methoxyethyl)amino)phenyl)methyl)-7-oxo-4,5,6,7-tetrahydro-2*H*-pyrazolo[3,4-*c*]pyridine-3-carboxamide (**71**)

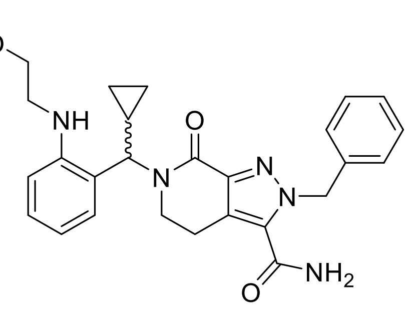

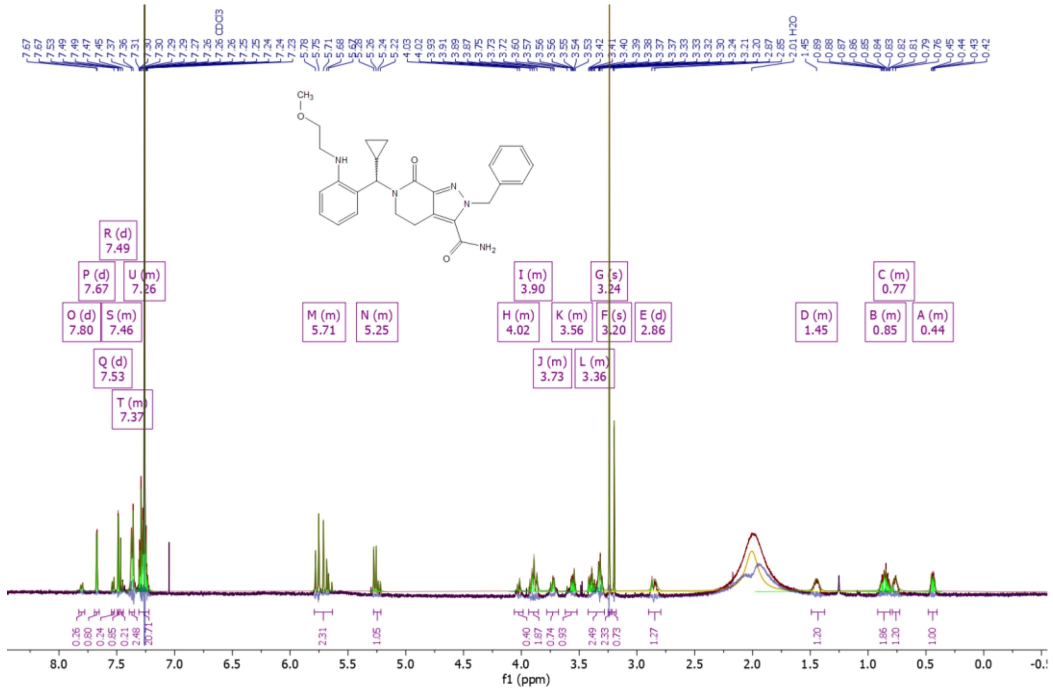

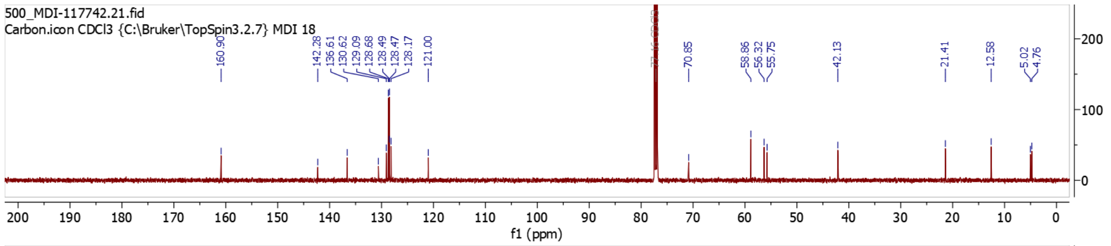

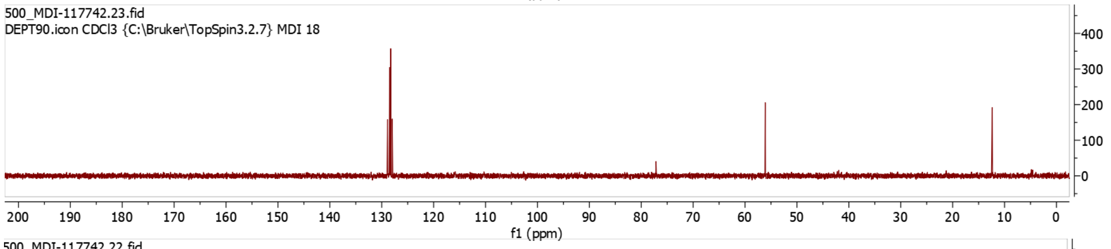

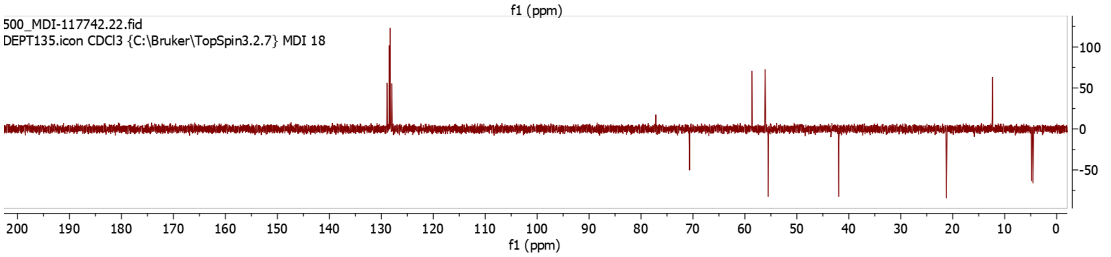

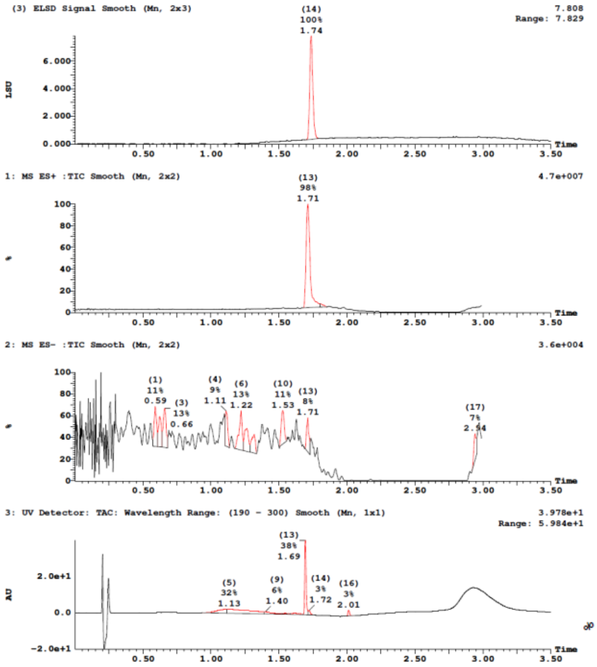

Peak ID	Compound	Time	Mass Found
13		1.71	Not Found

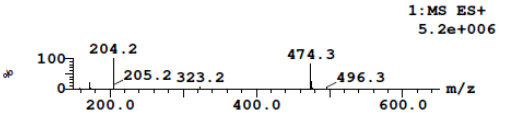

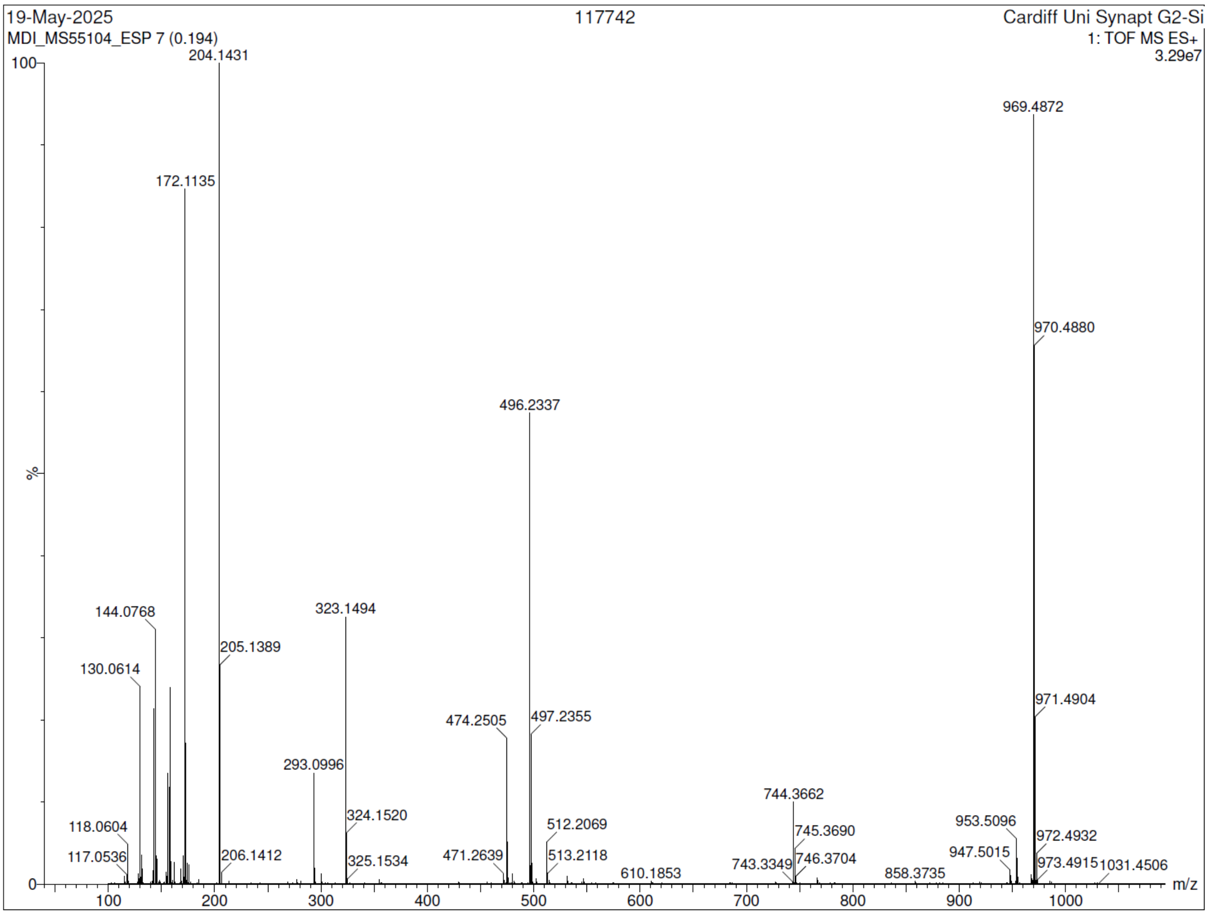

Single Mass Analysis

Tolerance = 50.0 PPM / DBE: min = -1.5, max = 50.0
Element prediction: Off
Number of isotope peaks used for i-FIT = 3

Monoisotopic Mass, Odd and Even Electron Ions
19 formula(e) evaluated with 1 results within limits (up to 50 closest results for each mass)
Elements Used:
C: 0-27 H: 0-32 N: 0-5 O: 0-3

19-May-2025 117742 Cardiff Uni Synapt G2-Si
MDI_MS55104_ESP 7 (0.194) 1: TOF MS ES+
5.85e+006

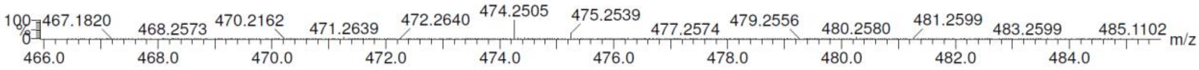

Minimum:				-1.5				
Maximum:		5.0	50.0	50.0				
Mass	Calc. Mass	mDa	PPM	DBE	i-FIT	Norm	Conf(%)	Formula
474.2505	474.2505	0.0	0.0	14.5	977.9	n/a	n/a	C27 H32 N5 O3

N-(2-(2-Benzyl-3-carbamoyl-7-oxo-2,4,5,7-tetrahydro-6*H*-pyrazolo[3,4-*c*]pyridin-6-yl)-2-phenylethyl)thiazole-5-carboxamide (**72**)

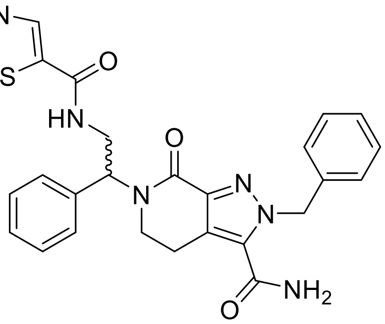

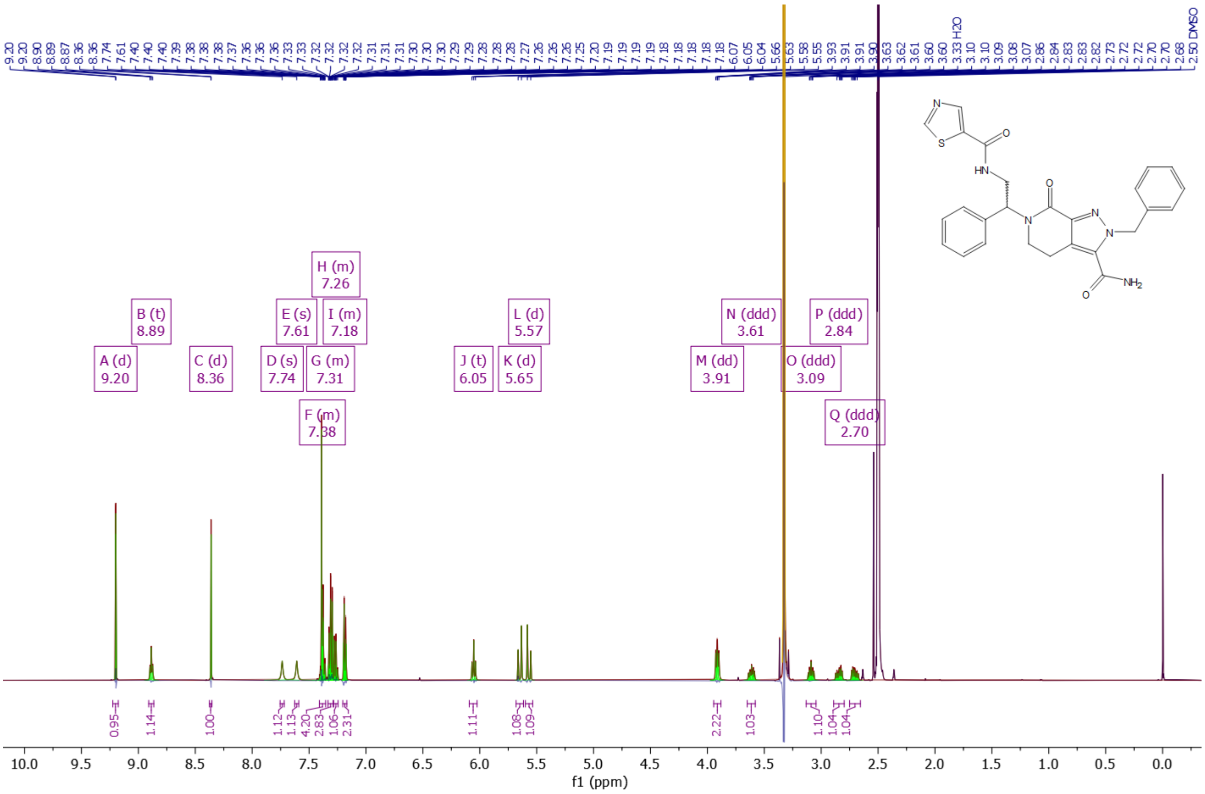

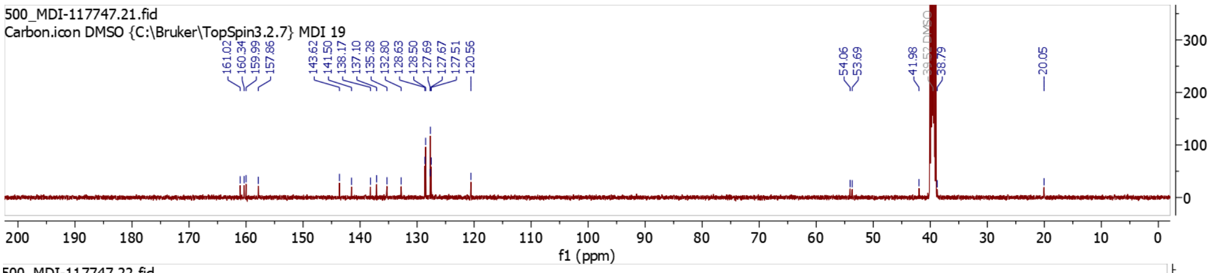

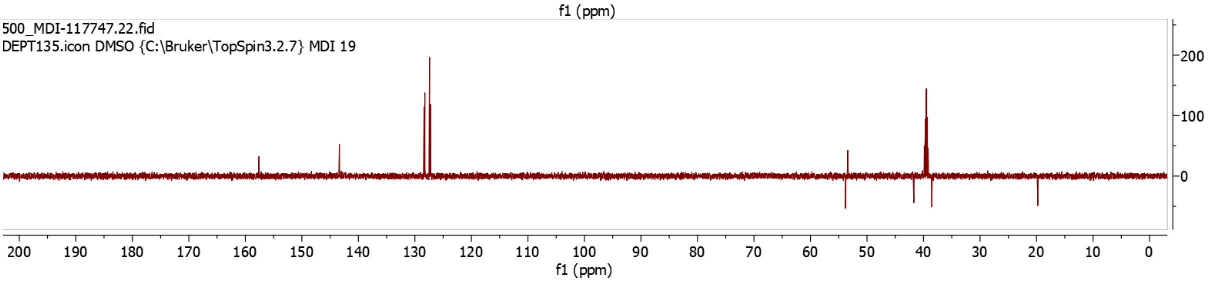

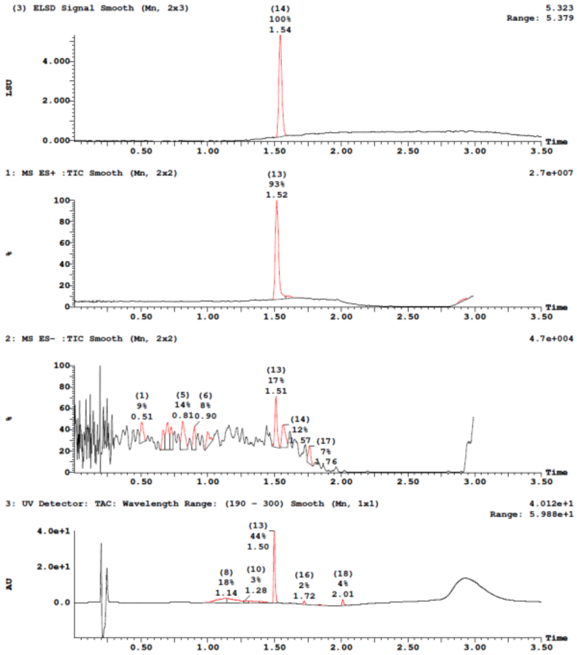

Peak ID	Compound	Time	Mass Found
13		1.52	Not Found

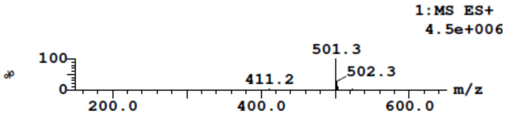

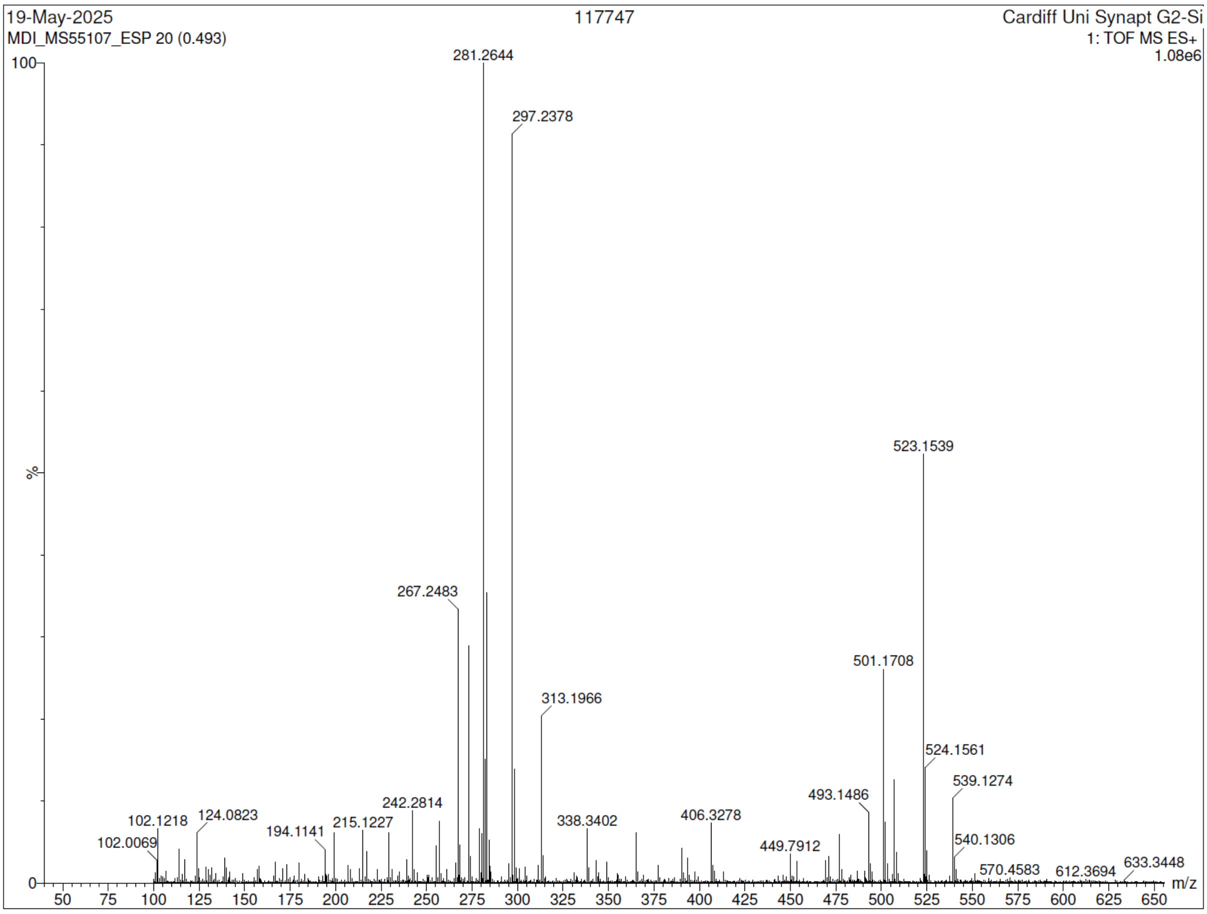

Elemental Composition Report

Single Mass Analysis

Tolerance = 50.0 PPM / DBE: min = -1.5, max = 50.0
Element prediction: Off
Number of isotope peaks used for i-FIT = 3

Monoisotopic Mass, Odd and Even Electron Ions
27 formula(e) evaluated with 1 results within limits (up to 50 closest results for each mass)
Elements Used:
C: 0-26 H: 0-25 N: 0-6 O: 3-3 S: 0-3

19-May-2025 117747 Cardiff Uni Synapt G2-Si
MDI_MS55107_ESP 20 (0.493) 1: TOF MS ES+
2.81e+005

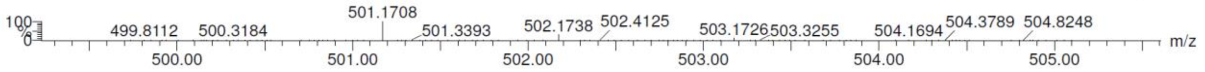

		mDa	PPM	DBE				
Minimum:				-1.5				
Maximum:		5.0	50.0	50.0				

Mass	Calc. Mass	mDa	PPM	DBE	i-FIT	Norm	Conf(%)	Formula
501.1708	501.1709	-0.1	-0.2	17.5	702.2	n/a	n/a	C26 H25 N6 O3 S

2,6-Dibenzyl-7-oxo-4,5,6,7-tetrahydro-2*H*-pyrazolo[3,4-*c*]pyridine-3-carboxylic acid (**81**)

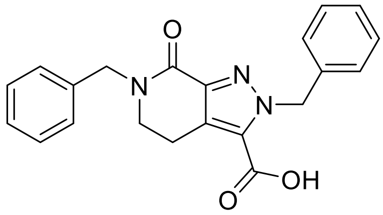

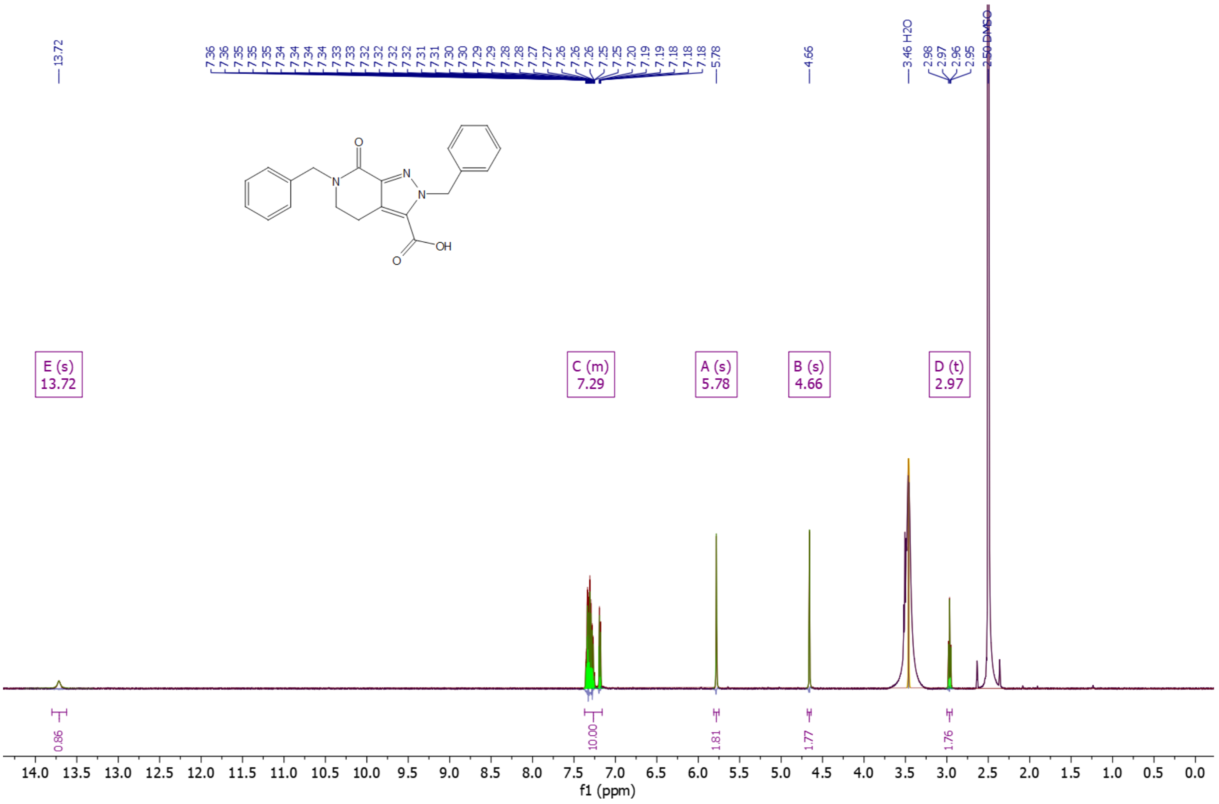

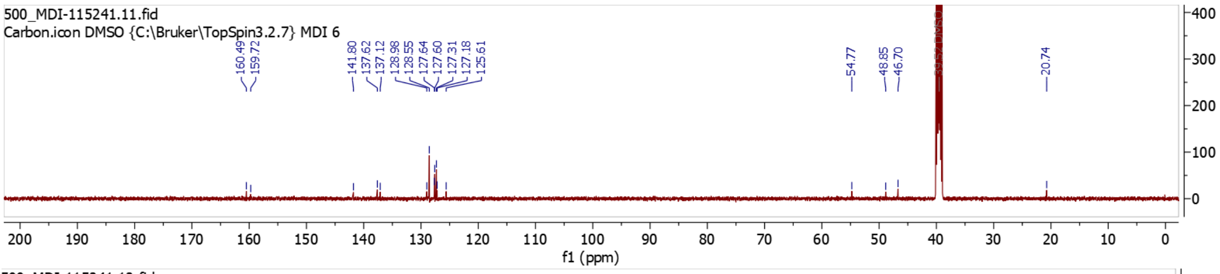

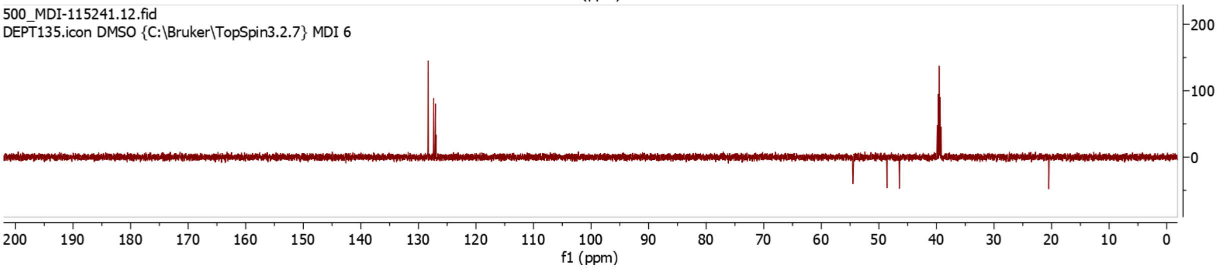

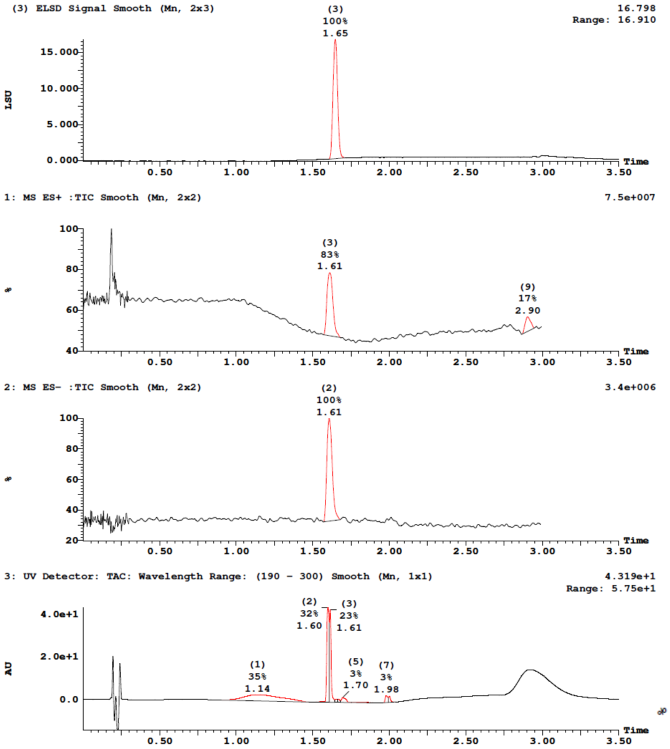

Peak ID	Compound	Time	Mass Found
2		1.61	Not Found

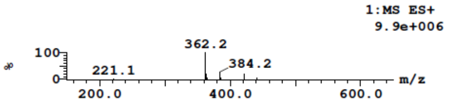

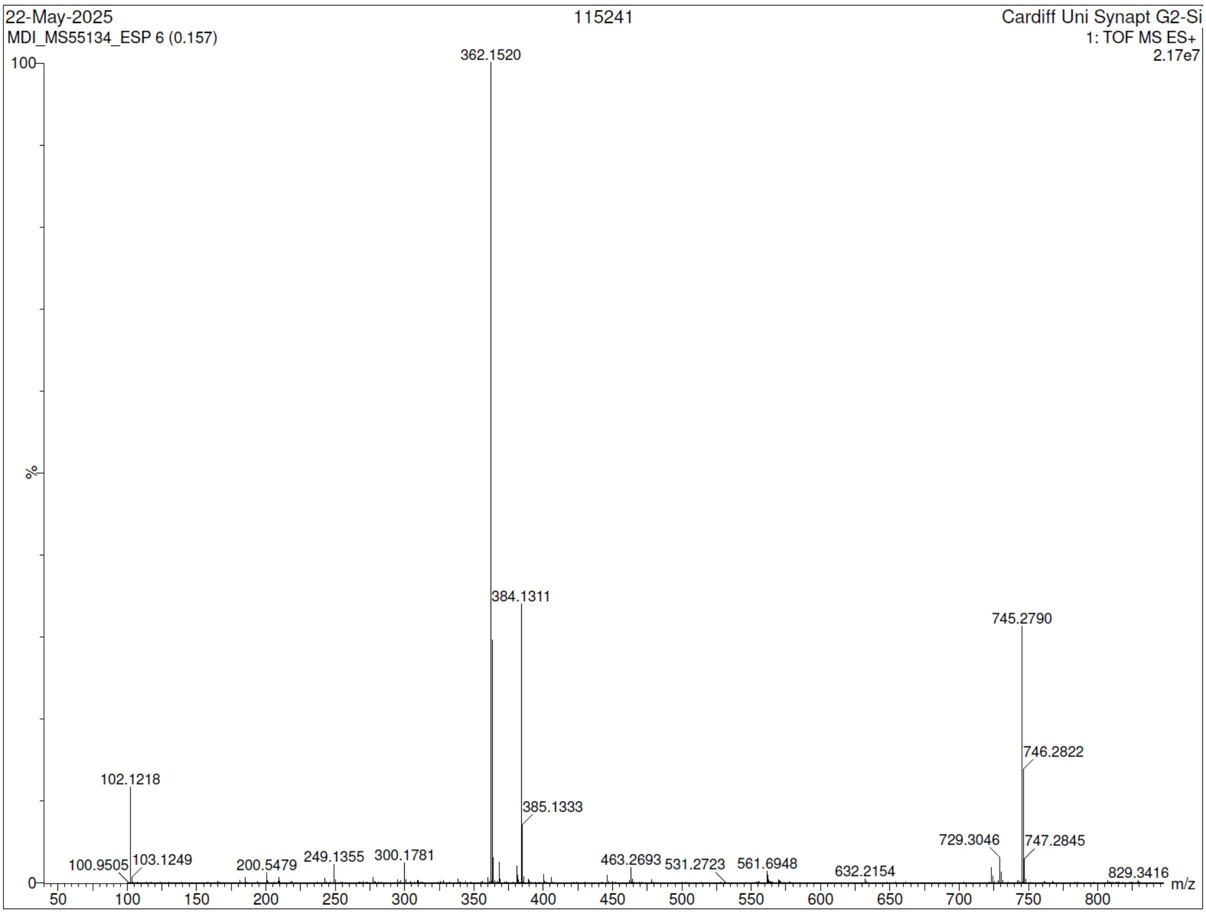

Elemental Composition Report

Single Mass Analysis

Tolerance = 50.0 PPM / DBE: min = -1.5, max = 50.0
Element prediction: Off
Number of isotope peaks used for i-FIT = 3

Monoisotopic Mass, Even Electron Ions
11 formula(e) evaluated with 1 results within limits (up to 50 closest results for each mass)
Elements Used:
C: 0-21 H: 0-20 N: 0-3 O: 0-3

22-May-2025 115241 Cardiff Uni Synapt G2-Si
MDI_MS55134_ESP 6 (0.157) 1: TOF MS ES+
2.17e+007

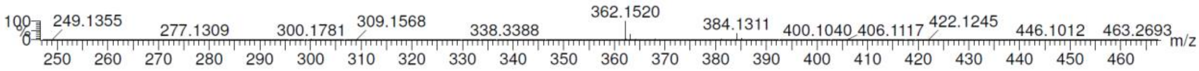

Minimum:				-1.5				
Maximum:		5.0	50.0	50.0				
Mass	Calc. Mass	mDa	PPM	DBE	i-FIT	Norm	Conf(%)	Formula
362.1520	362.1505	1.5	4.1	13.5	1177.4	n/a	n/a	C21 H20 N3 O3

2,6-Dibenzyl-*N*-methyl-7-oxo-4,5,6,7-tetrahydro-2*H*-pyrazolo[3,4-*c*]pyridine-3-carboxamide (**82**)

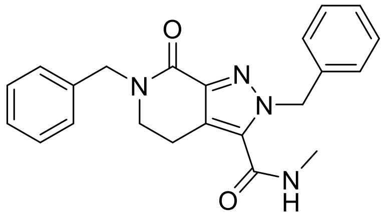

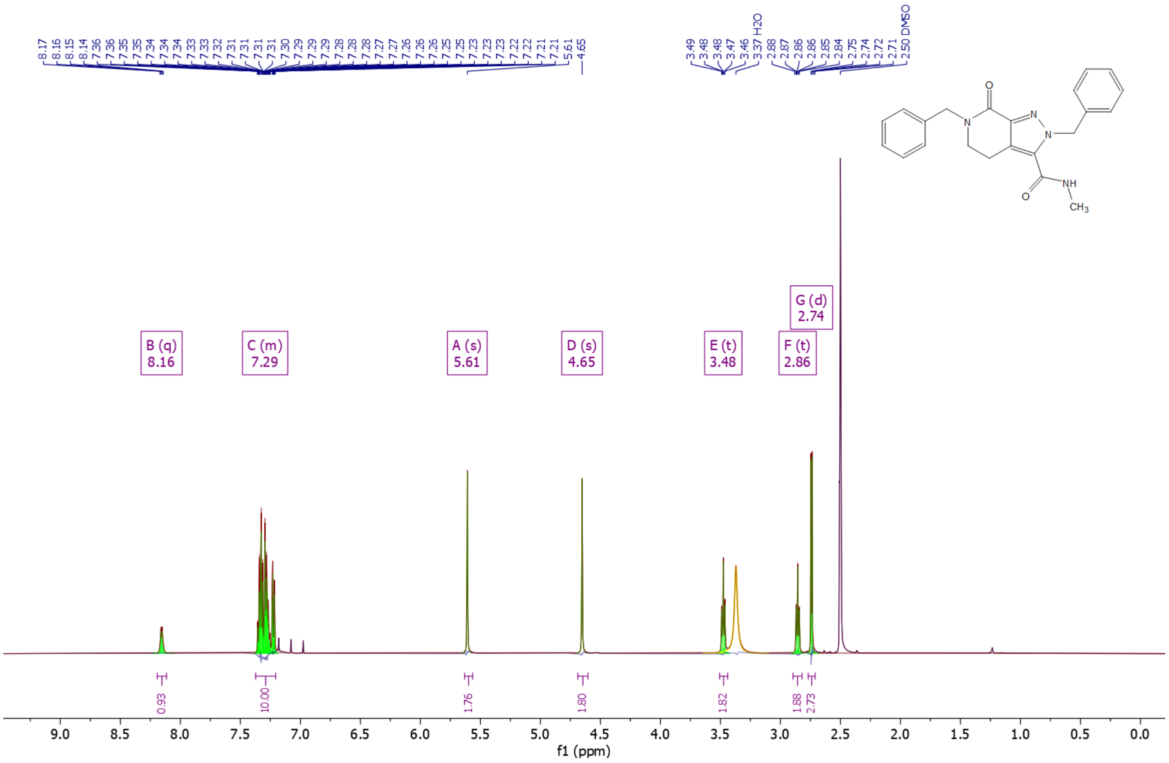

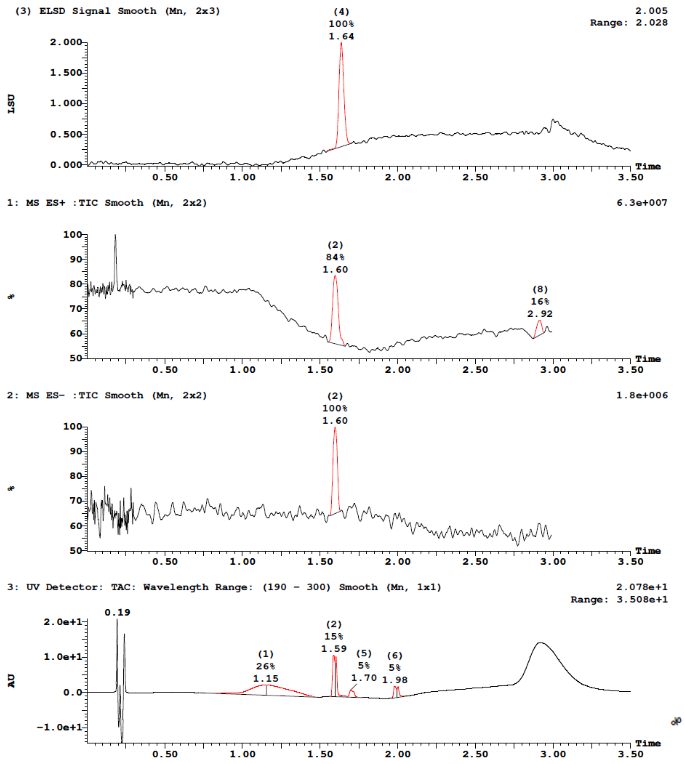

Peak ID	Compound	Time	Mass Found
2		1.60	Not Found

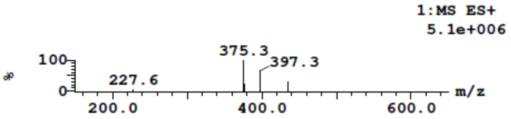

2,6-Dibenzyl-*N*-cyclopropyl-7-oxo-4,5,6,7-tetrahydro-2*H*-pyrazolo[3,4-*c*]pyridine-3-carboxamide (**83**)

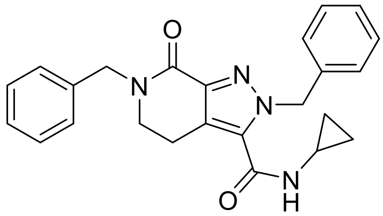

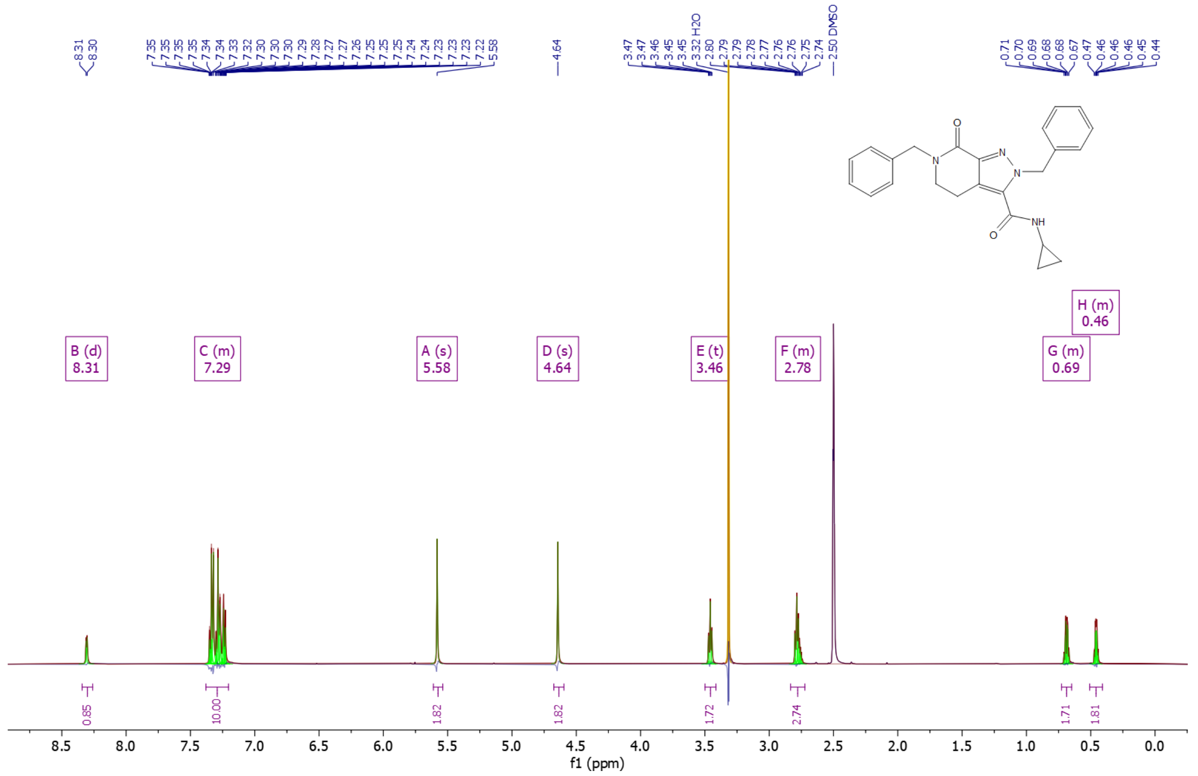

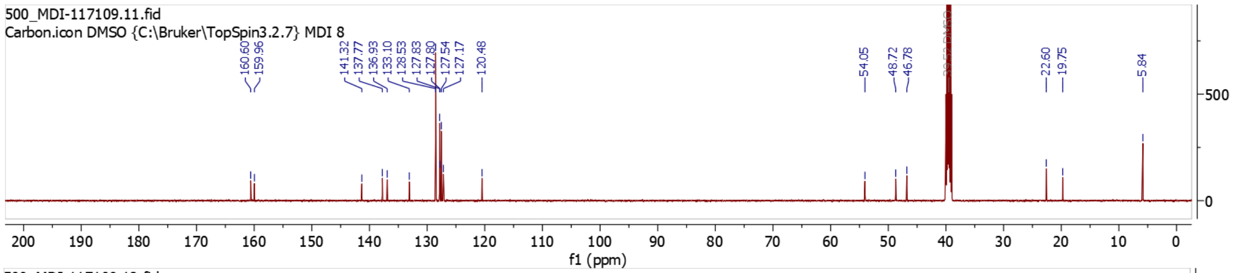

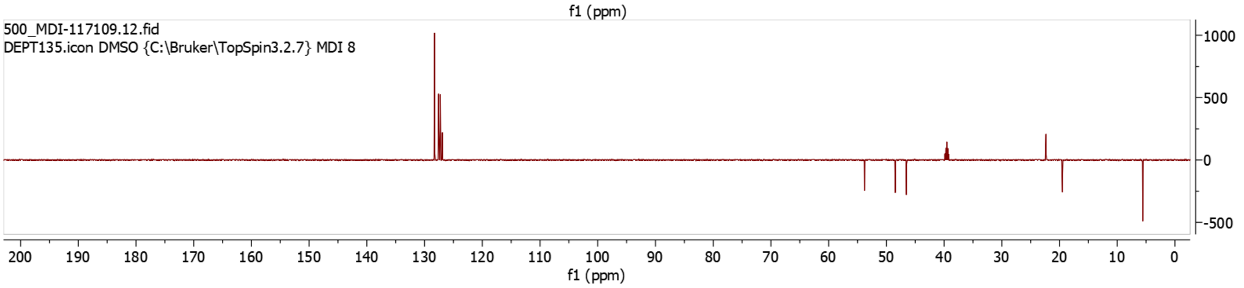

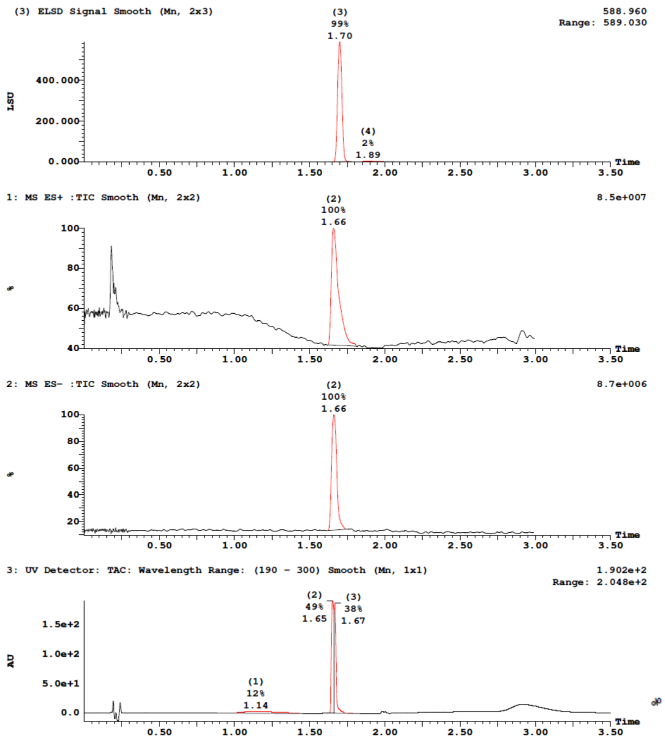

Peak ID	Compound	Time	Mass Found
2		1.66	Not Found

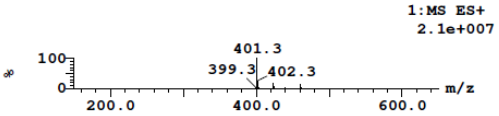

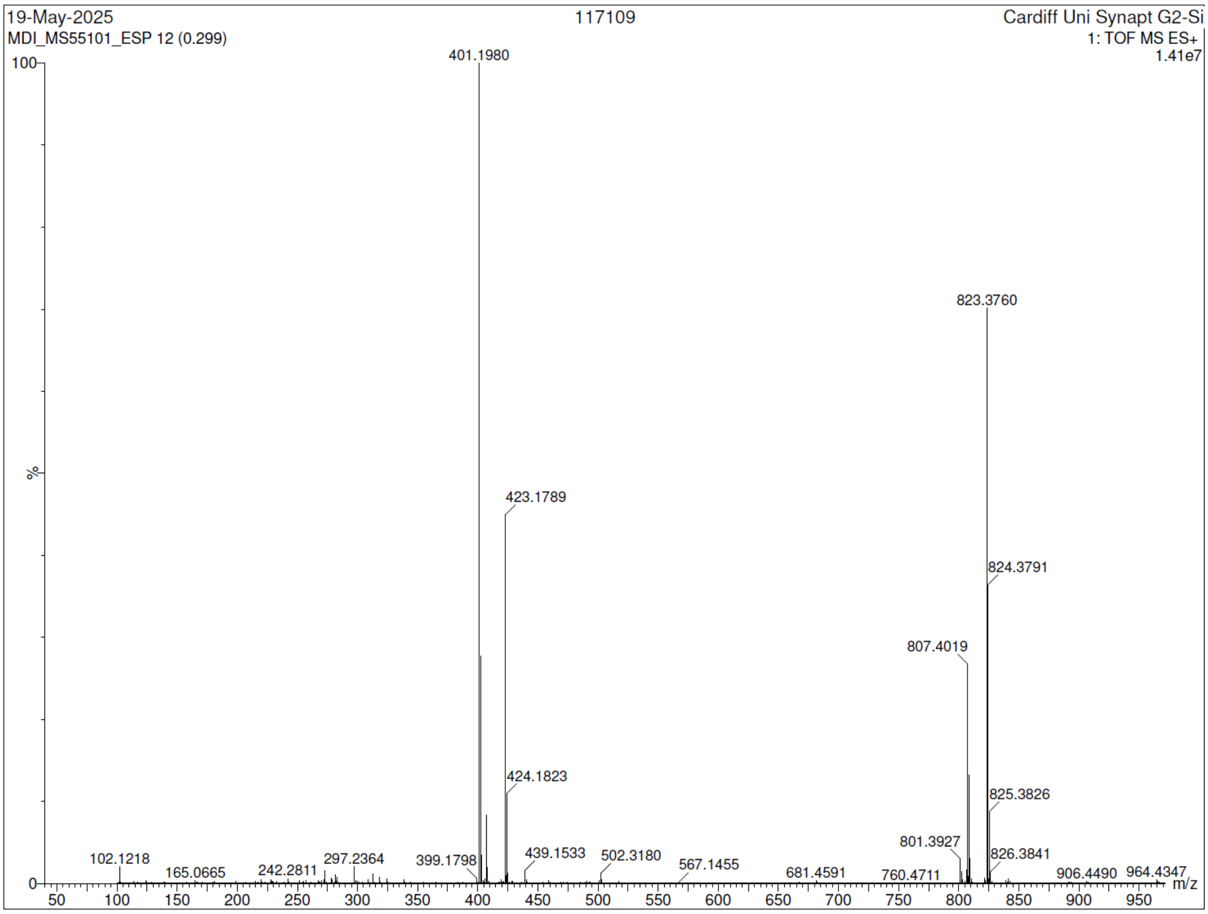

Elemental Composition Report

Single Mass Analysis

Tolerance = 50.0 PPM / DBE: min = -1.5, max = 50.0
Element prediction: Off
Number of isotope peaks used for i-FIT = 3

Monoisotopic Mass, Odd and Even Electron Ions
10 formula(e) evaluated with 1 results within limits (up to 50 closest results for each mass)
Elements Used:
C: 0-24 H: 0-25 N: 0-4 O: 0-2

19-May-2025 | 117109 | Cardiff Uni Synapt G2-Si
MDI_MS55101_ESP 12 (0.299) | 1: TOF MS ES+
1.41e+007

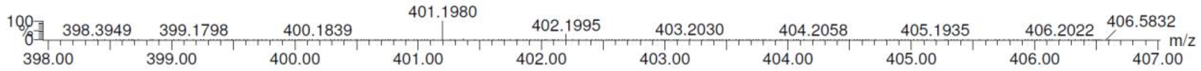

Mass	Calc. Mass	mDa	PPM	DBE	i-FIT	Norm	Conf(%)	Formula
Minimum:				-1.5				
Maximum:		5.0	50.0	50.0				
401.1980	401.1978	0.2	0.5	14.5	1082.7	n/a	n/a	C24 H25 N4 O2

2,6-Dibenzyl-7-oxo-*N*-phenyl-4,5,6,7-tetrahydro-2*H*-pyrazolo[3,4-*c*]pyridine-3-carboxamide (**84**)

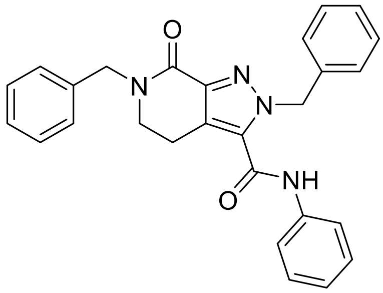

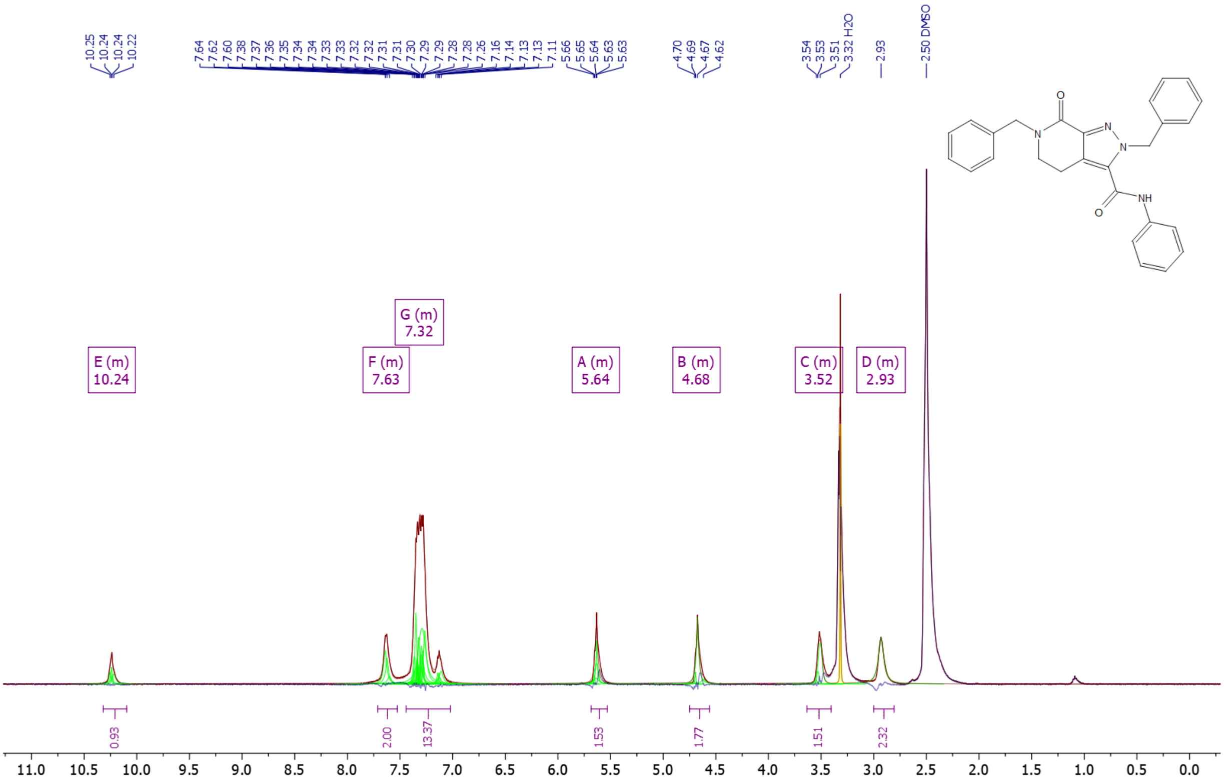

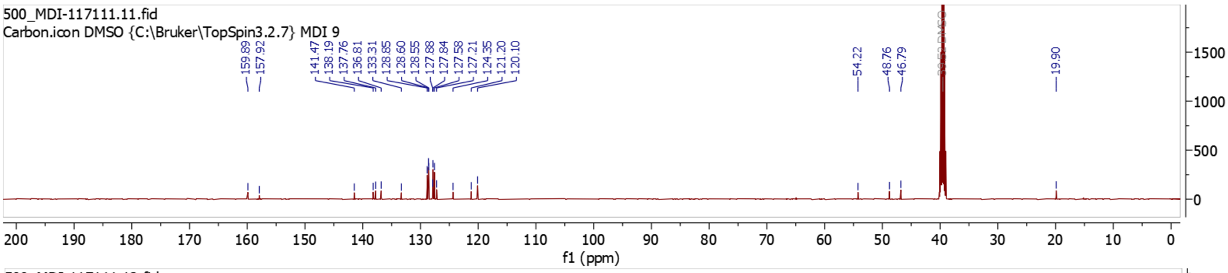

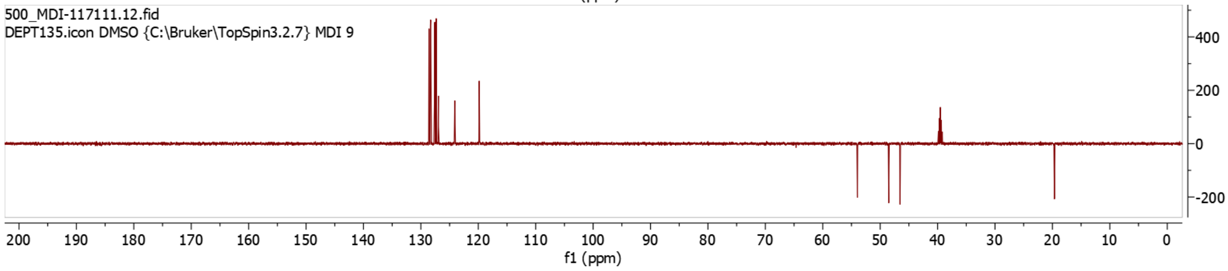

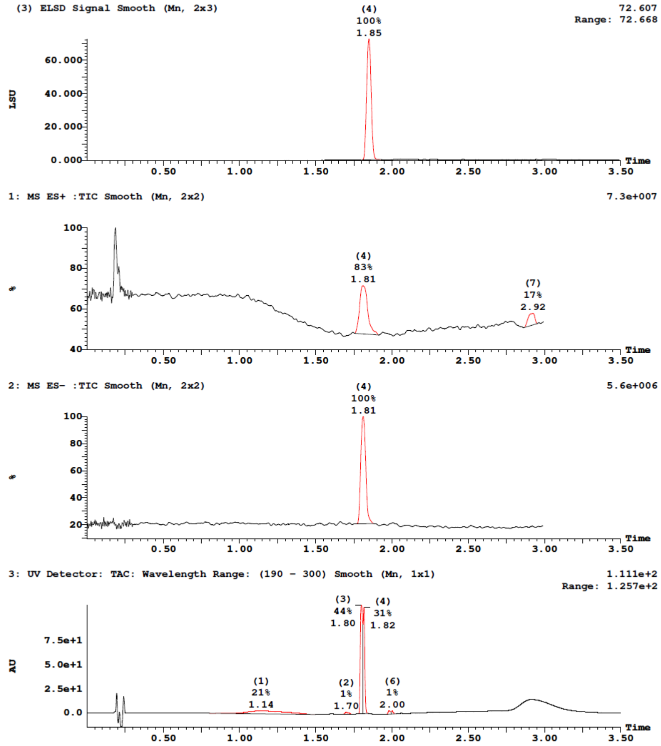

Peak ID	Compound	Time	Mass Found
3		1.80	Not Found

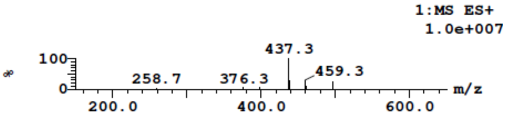

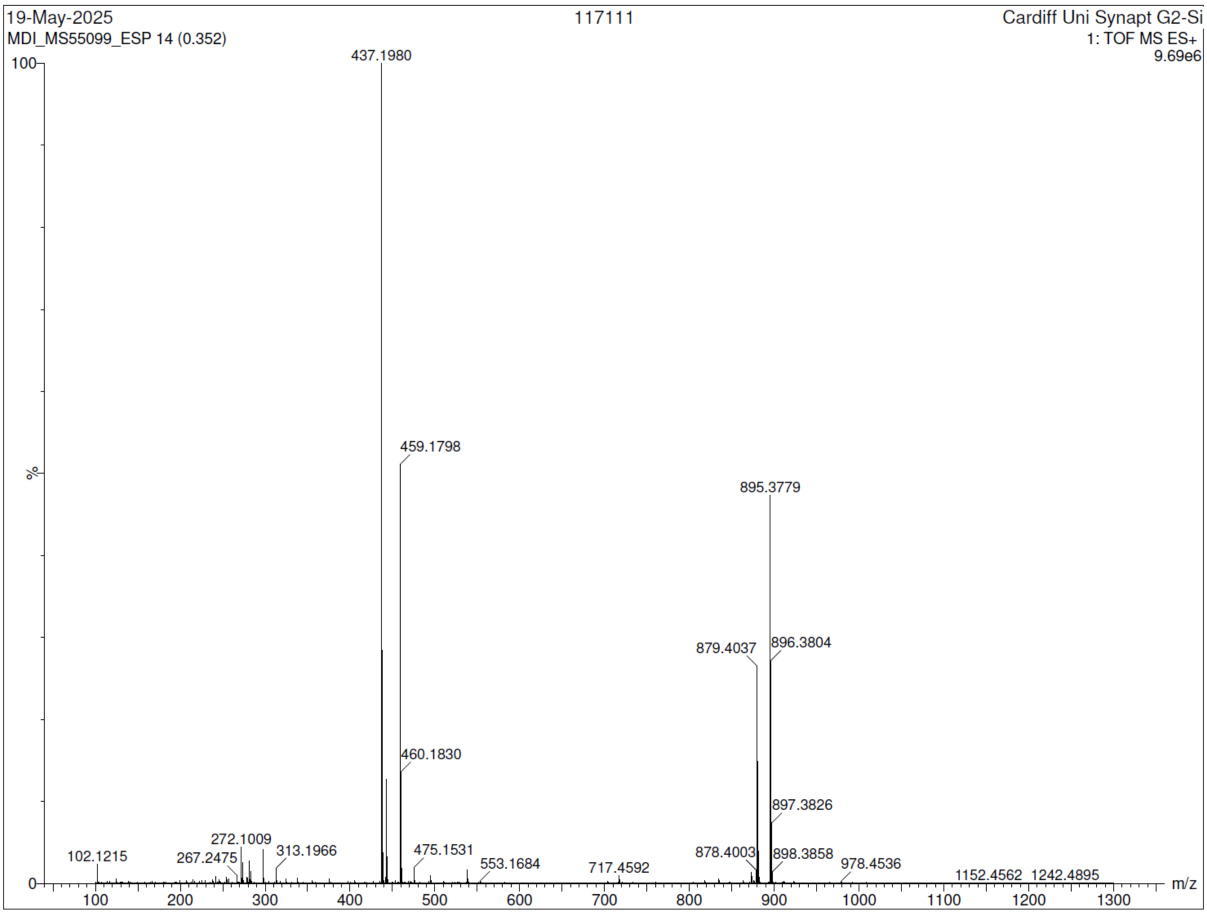

Elemental Composition Report

Single Mass Analysis
Tolerance = 50.0 PPM / DBE: min = -1.5, max = 50.0
Element prediction: Off
Number of isotope peaks used for i-FIT = 3

Monoisotopic Mass, Odd and Even Electron Ions
9 formula(e) evaluated with 1 results within limits (up to 50 closest results for each mass)
Elements Used:
C: 0-27 H: 0-25 N: 0-4 O: 0-2

19-May-2025 117111 Cardiff Uni Synapt G2-Si
MDI_MS55099_ESP 14 (0.352) 1: TOF MS ES+
9.69e+006

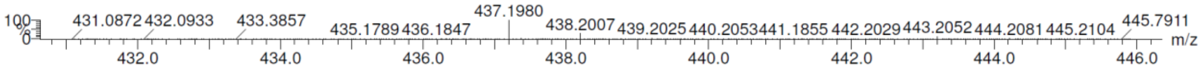

Minimum:				-1.5				
Maximum:		5.0	50.0	50.0				
Mass	Calc. Mass	mDa	PPM	DBE	i-FIT	Norm	Conf(%)	Formula
437.1980	437.1978	0.2	0.5	17.5	1054.2	n/a	n/a	C27 H25 N4 O2

2,6-Dibenzyl-*N*-(2-methoxyethyl)-7-oxo-4,5,6,7-tetrahydro-2*H*-pyrazolo[3,4-*c*]pyridine-3-carboxamide (**85**)

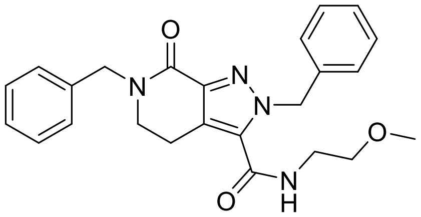

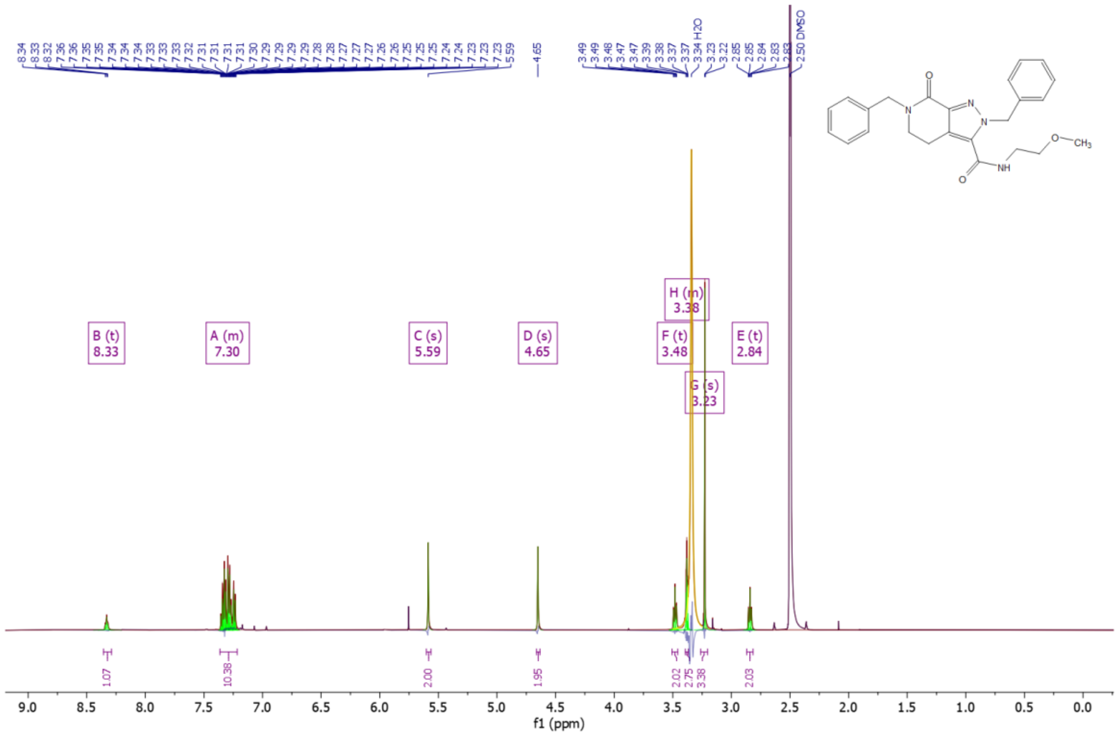

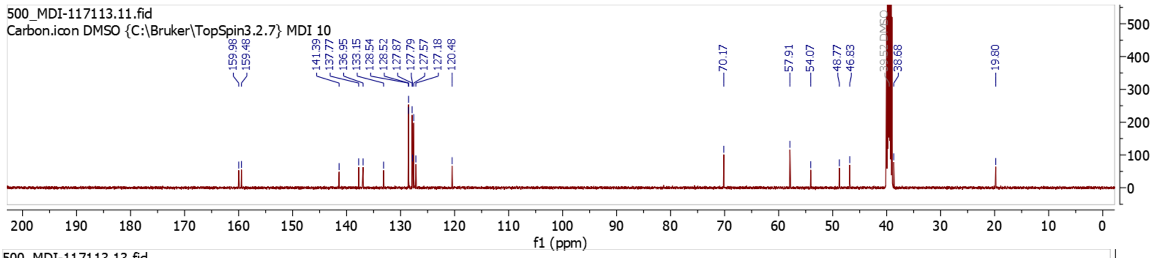

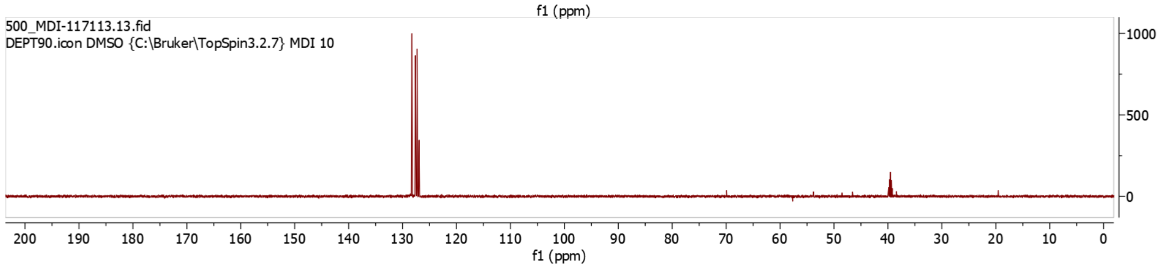

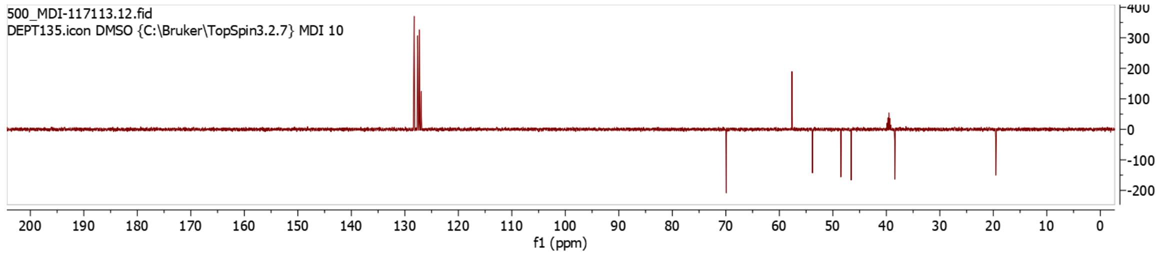

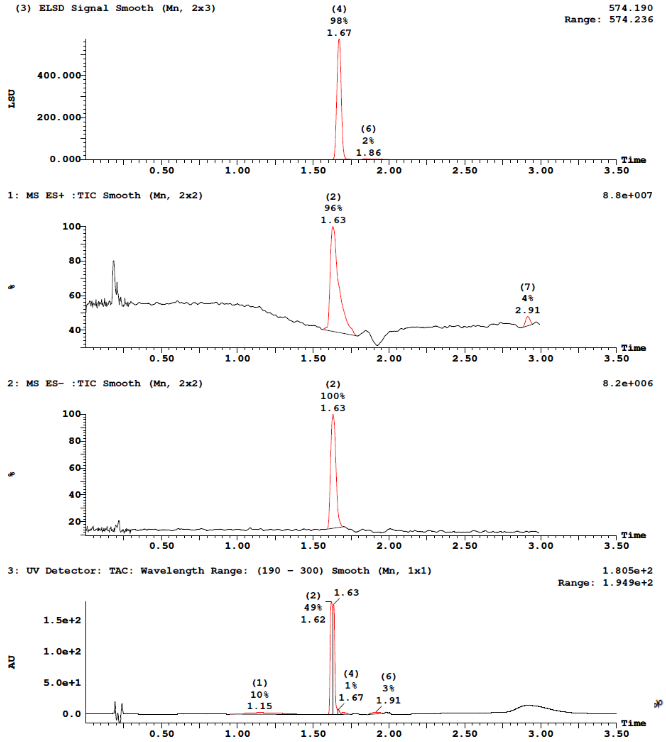

Peak ID	Compound	Time	Mass Found
2		1.63	Not Found

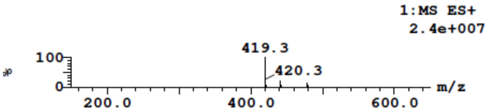

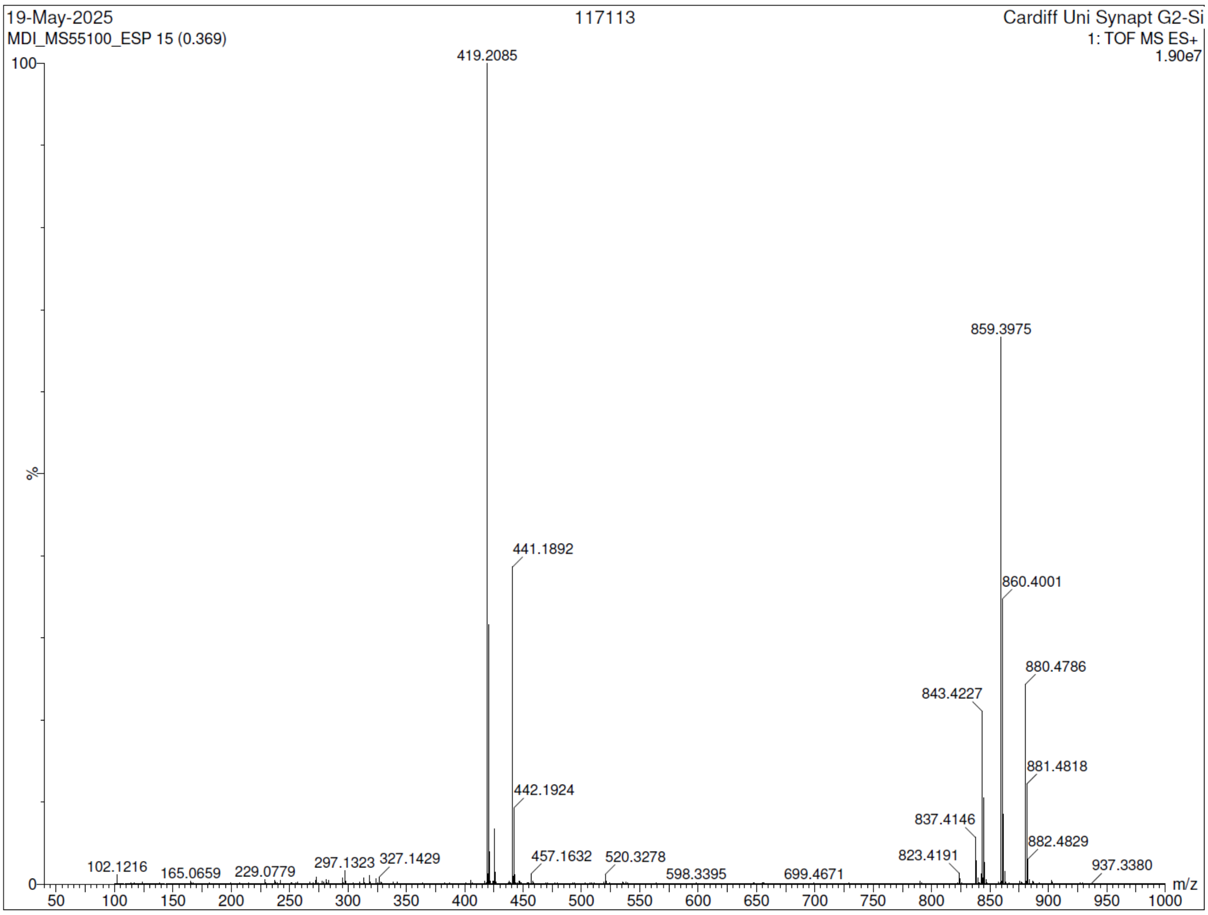

Elemental Composition Report

Single Mass Analysis

Tolerance = 50.0 PPM / DBE: min = -1.5, max = 50.0
Element prediction: Off
Number of isotope peaks used for i-FIT = 3

Monoisotopic Mass, Odd and Even Electron Ions
15 formula(e) evaluated with 1 results within limits (up to 50 closest results for each mass)
Elements Used:
C: 0-24 H: 0-27 N: 0-4 O: 0-3

19-May-2025 117113 Cardiff Uni Synapt G2-Si
MDI_MS55100_ESP 15 (0.369) 1: TOF MS ES+
1.90e+007

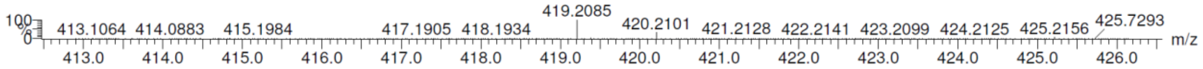

Minimum:				-1.5				
Maximum:		5.0	50.0	50.0				
Mass	Calc. Mass	mDa	PPM	DBE	i-FIT	Norm	Conf(%)	Formula
419.2085	419.2083	0.2	0.5	13.5	1111.6	n/a	n/a	C24 H27 N4 O3

2,6-Dibenzyl-*N*-(2-(dimethylamino)ethyl)-7-oxo-4,5,6,7-tetrahydro-2*H*-pyrazolo[3,4-*c*]pyridine-3-carboxamide (**86**)

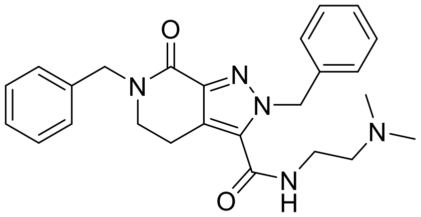

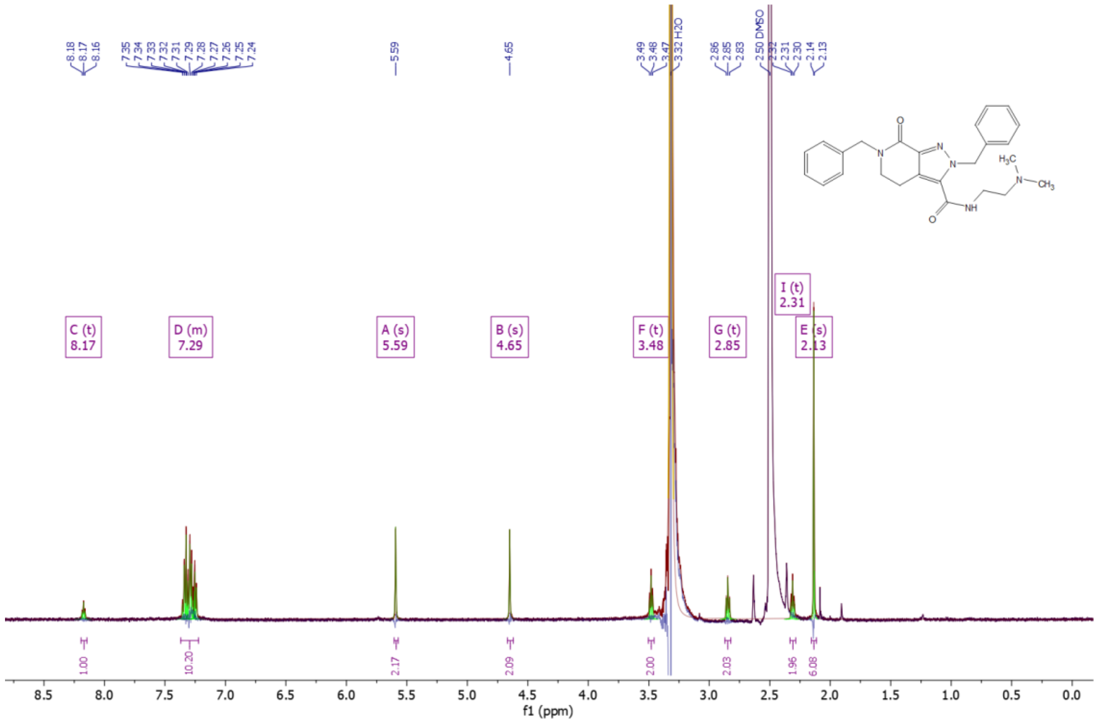

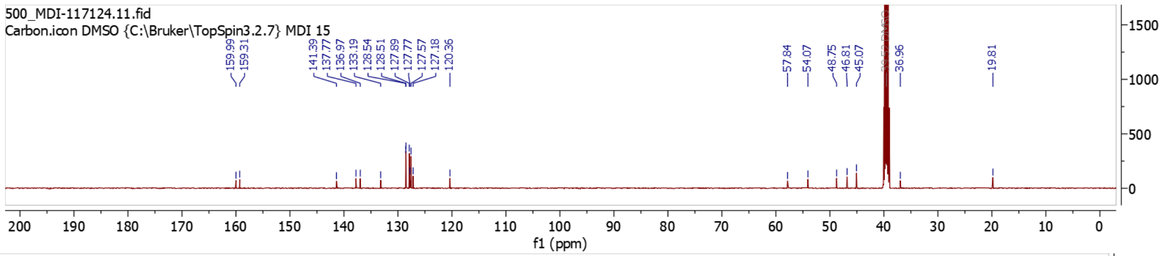

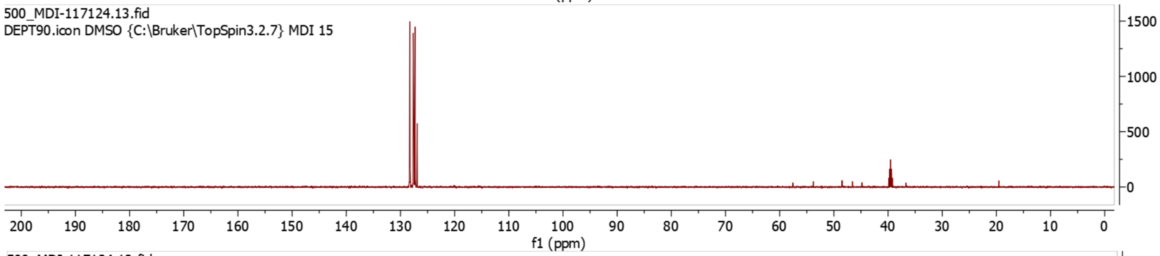

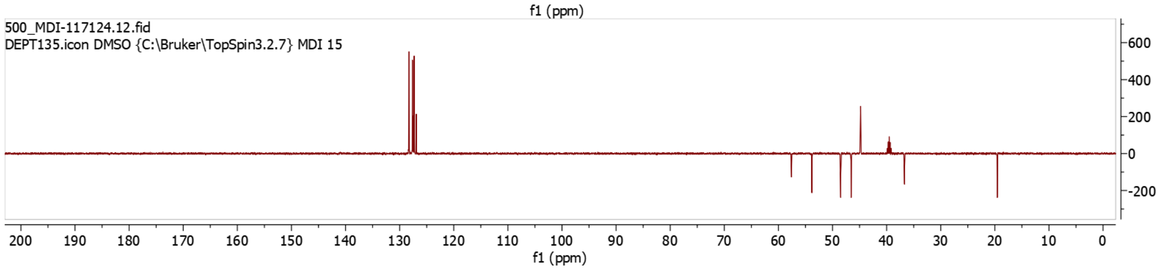

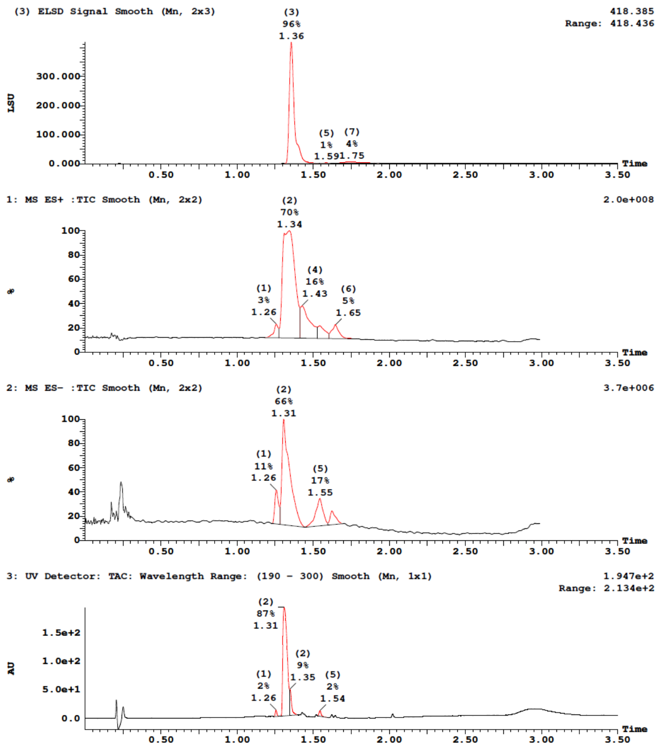

Peak ID	Compound	Time	Mass Found
2		1.34	Not Found

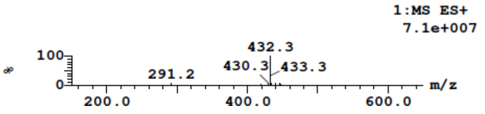

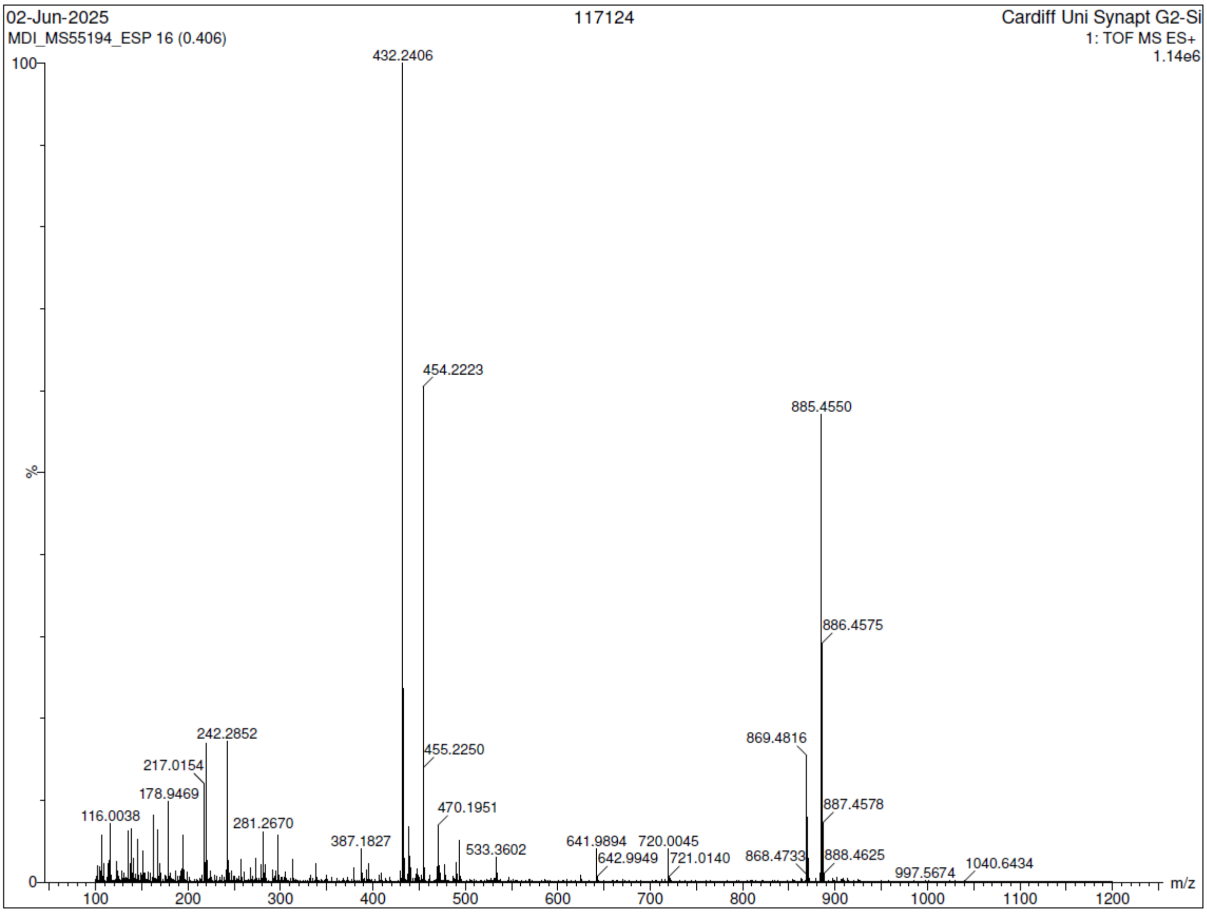

Single Mass Analysis

Tolerance = 10.0 PPM / DBE: min = -1.5, max = 50.0
Element prediction: Off
Number of isotope peaks used for i-FIT = 3

Monoisotopic Mass, Odd and Even Electron Ions
13 formula(e) evaluated with 1 results within limits (up to 50 closest results for each mass)
Elements Used:
C: 0-25 H: 0-30 N: 0-5 O: 0-2

02-Jun-2025 117124 Cardiff Uni Synapt G2-Si
MDI_MS55194_ESP 16 (0.406) 1: TOF MS ES+
1.14e+006

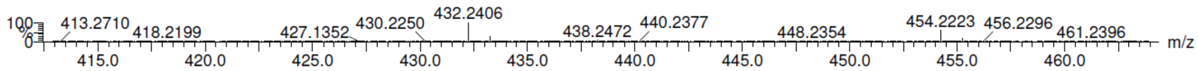

Minimum:				-1.5				
Maximum:		5.0	10.0	50.0				
Mass	Calc. Mass	mDa	PPM	DBE	i-FIT	Norm	Conf(%)	Formula
432.2406	432.2400	0.6	1.4	13.5	938.8	n/a	n/a	C25 H30 N5 O2

2,6-Dibenzyl-*N*-(2-hydroxyethyl)-7-oxo-4,5,6,7-tetrahydro-2*H*-pyrazolo[3,4-*c*]pyridine-3-carboxamide (**88**)

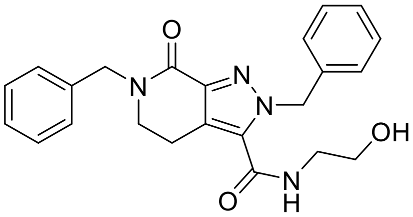

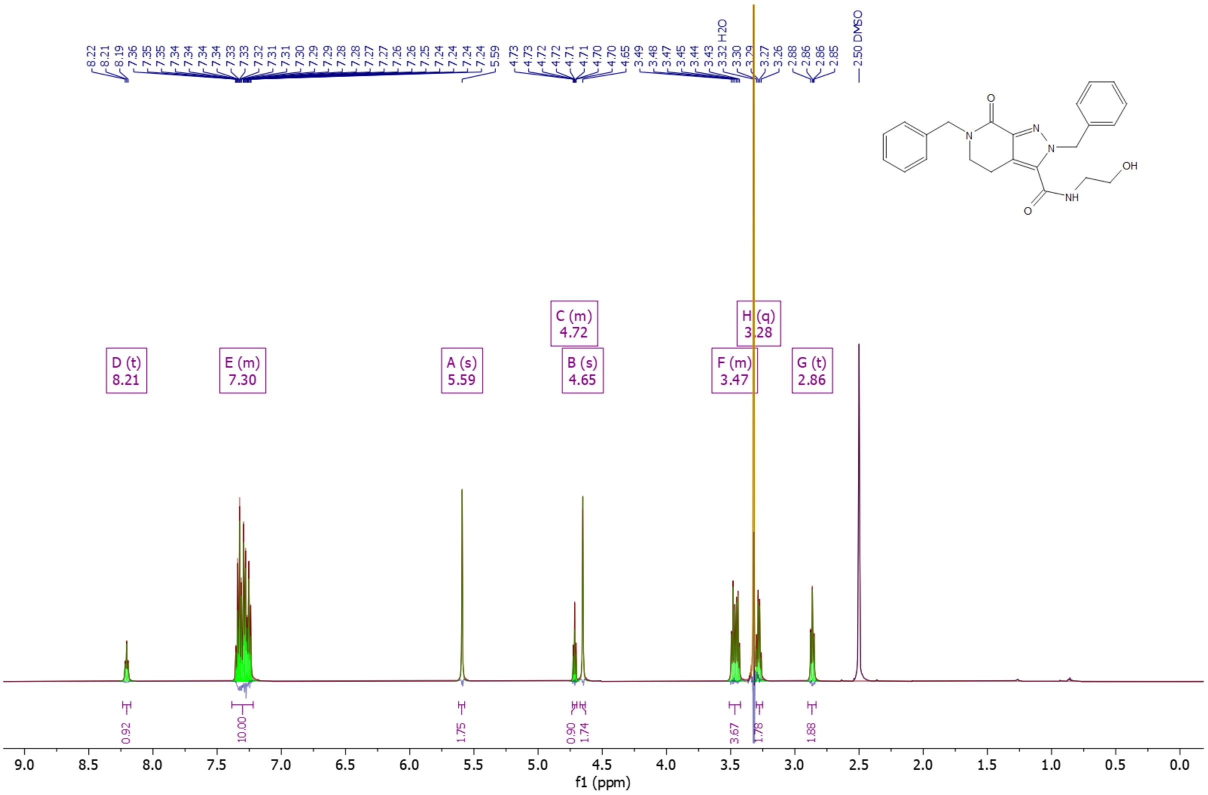

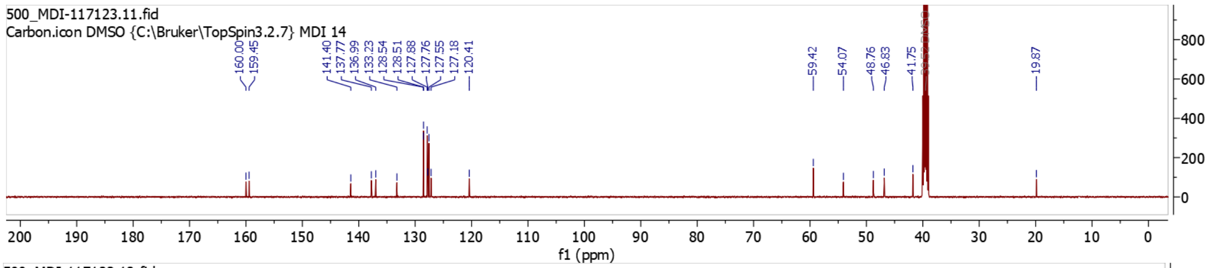

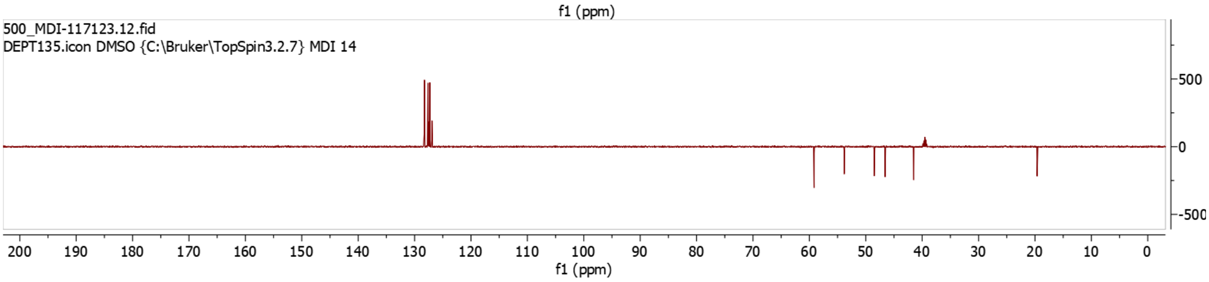

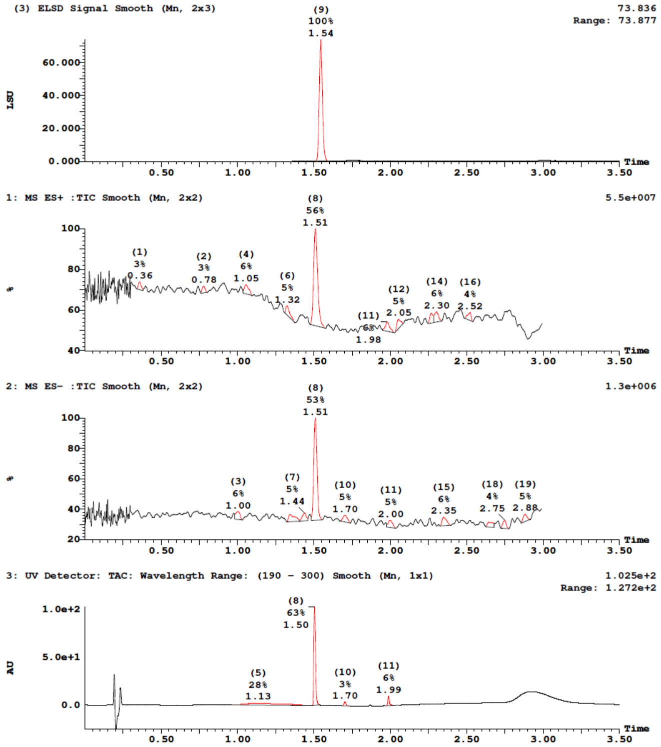

Peak ID	Compound	Time	Mass Found
9		1.54	Not Found

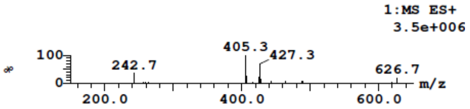

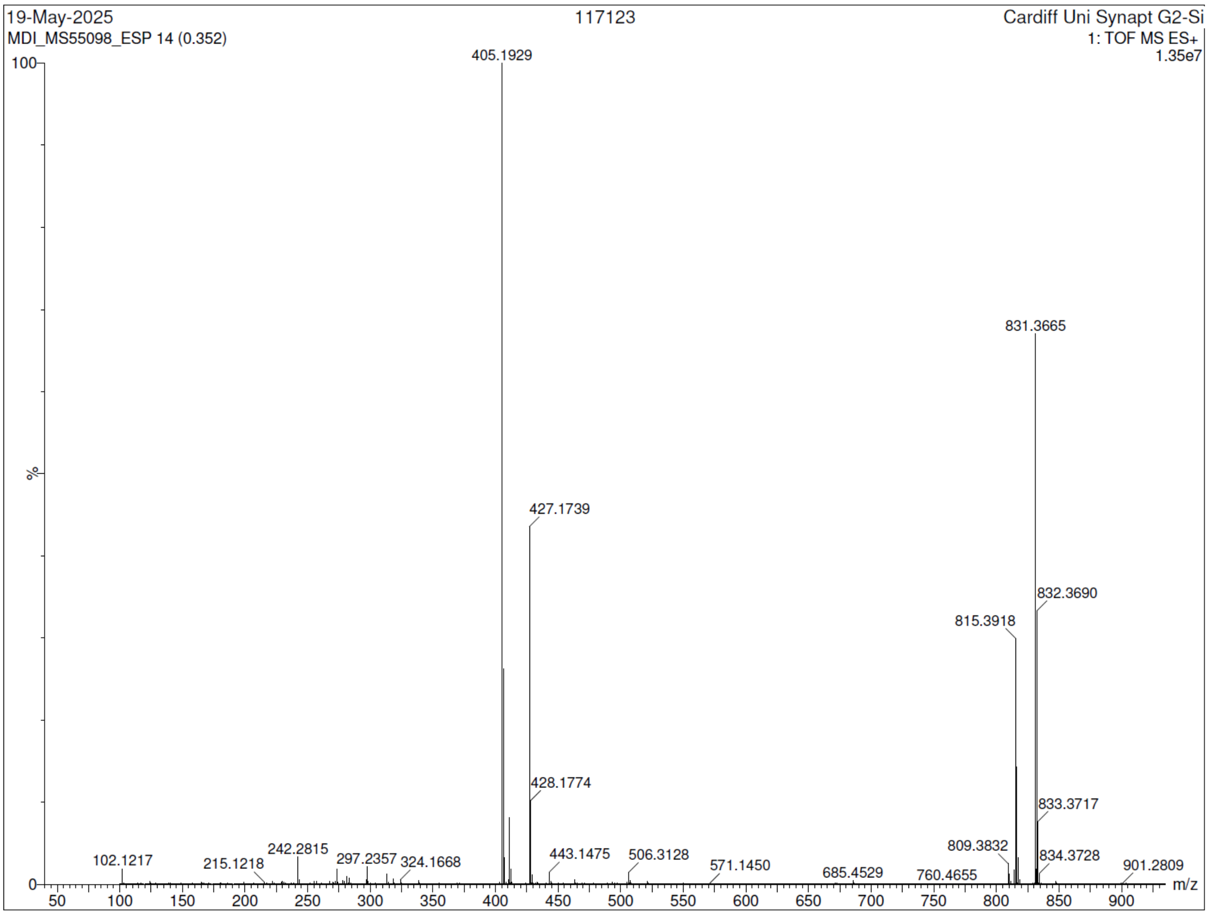

Elemental Composition Report

Single Mass Analysis

Tolerance = 50.0 PPM / DBE: min = -1.5, max = 50.0
Element prediction: Off
Number of isotope peaks used for i-FIT = 3

Monoisotopic Mass, Odd and Even Electron Ions
15 formula(e) evaluated with 1 results within limits (up to 50 closest results for each mass)
Elements Used:
C: 0-23 H: 0-25 N: 0-4 O: 0-3

19-May-2025 117123 Cardiff Uni Synapt G2-Si
MDI_MS55098_ESP 14 (0.352) 1: TOF MS ES+
1.35e+007

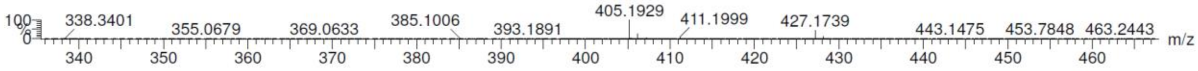

Minimum:				-1.5				
Maximum:		5.0	50.0	50.0				
Mass	Calc. Mass	mDa	PPM	DBE	i-FIT	Norm	Conf(%)	Formula
405.1929	405.1927	0.2	0.5	13.5	1075.6	n/a	n/a	C23 H25 N4 O3

2,6-Dibenzyl-3-(1,3,4-oxadiazol-2-yl)-2,4,5,6-tetrahydro-7*H*-pyrazolo[3,4-c]pyridin-7-one (**89**)

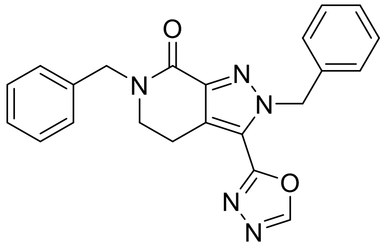

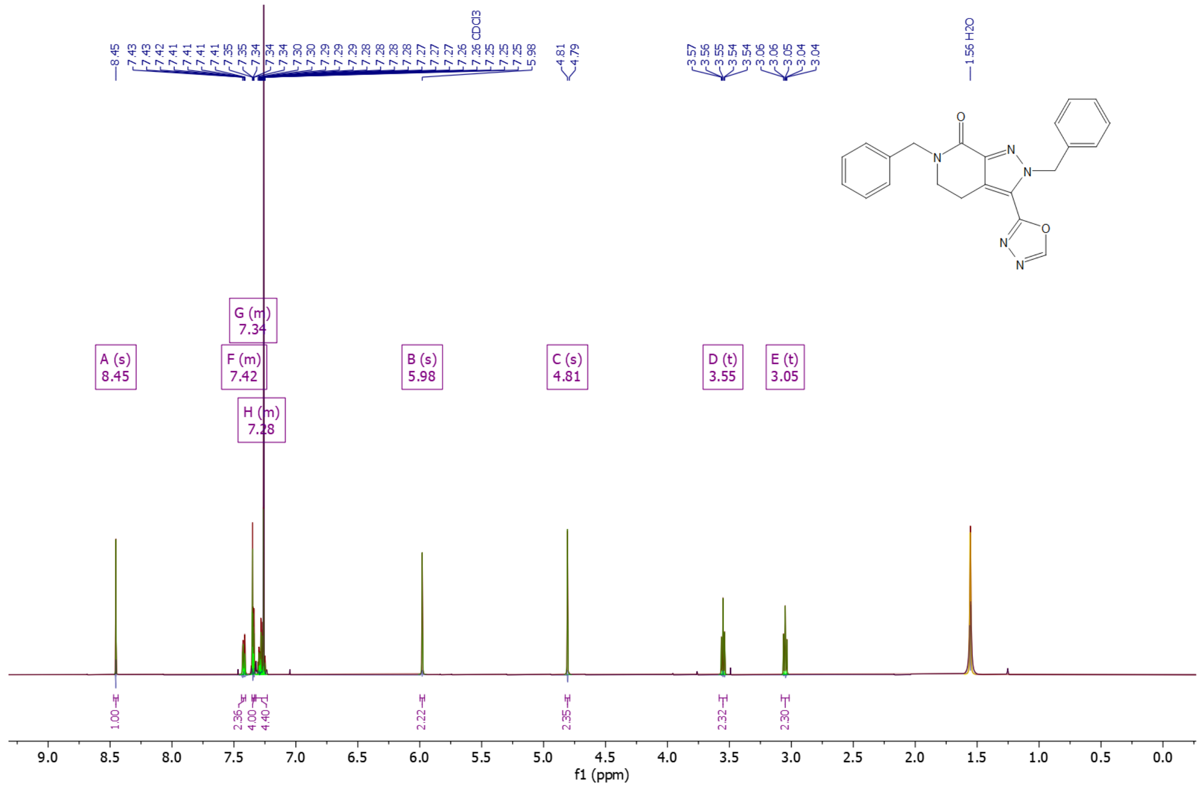

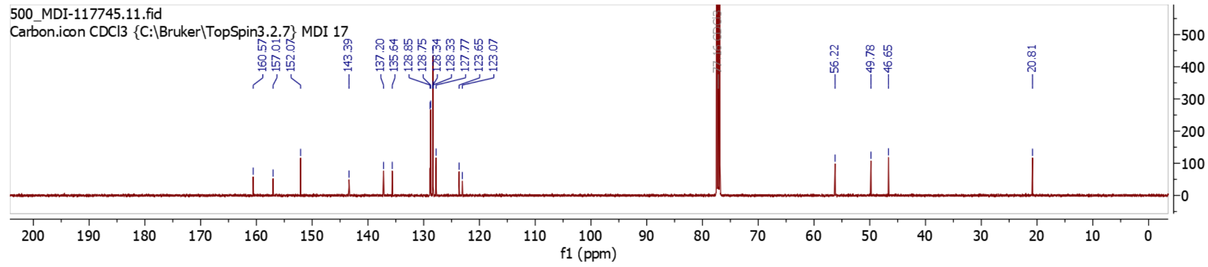

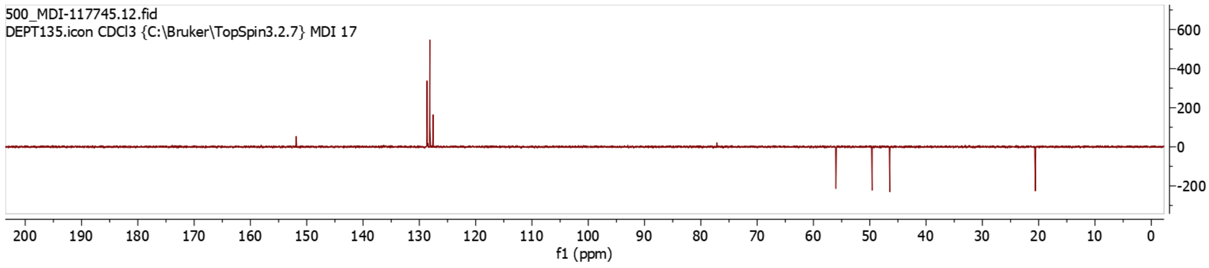

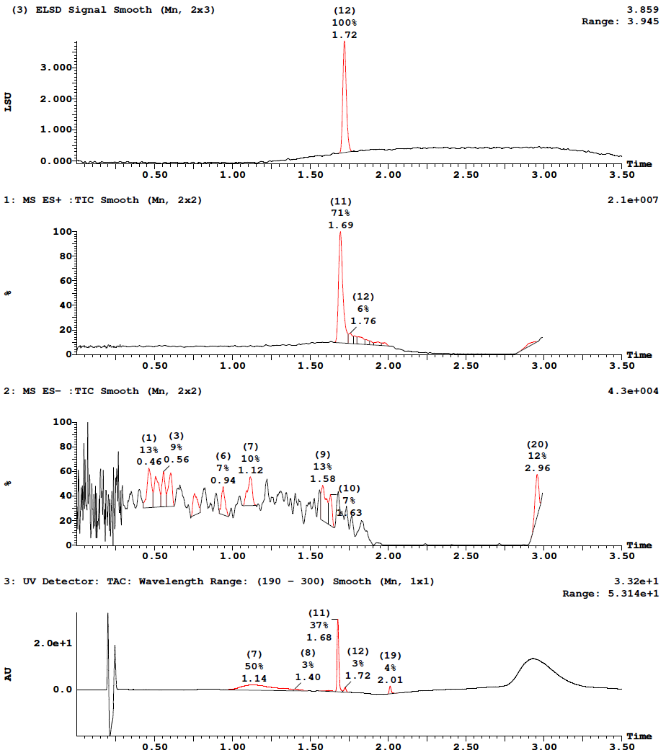

Peak ID	Compound	Time	Mass Found
11		1.69	Not Found

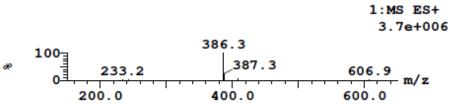

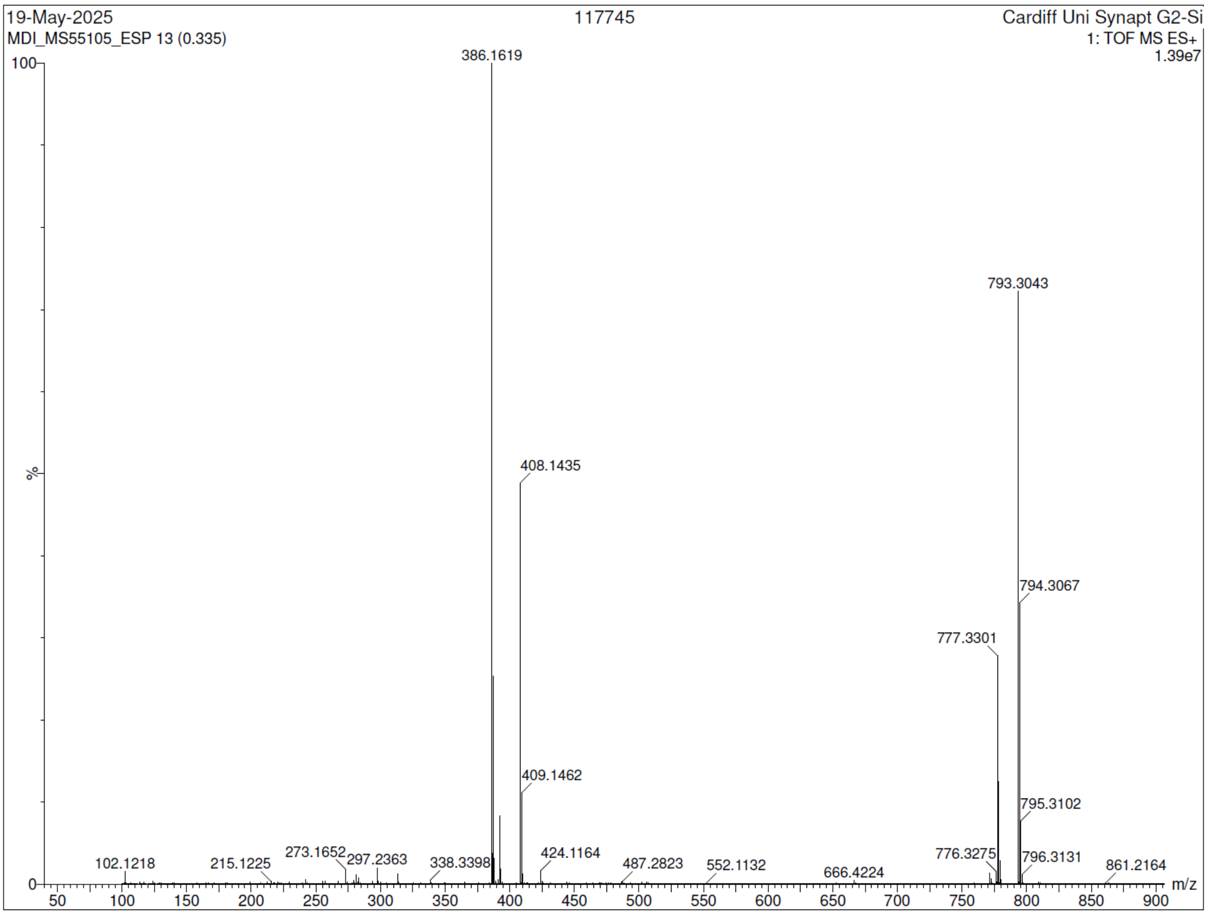

Elemental Composition Report

Single Mass Analysis

Tolerance = 50.0 PPM / DBE: min = -1.5, max = 50.0
Element prediction: Off
Number of isotope peaks used for i-FIT = 3

Monoisotopic Mass, Odd and Even Electron Ions
13 formula(e) evaluated with 1 results within limits (up to 50 closest results for each mass)
Elements Used:
C: 0-22 H: 0-20 N: 0-5 O: 0-2

19-May-2025 117745 Cardiff Uni Synapt G2-Si
MDI_MS55105_ESP 13 (0.335) 1: TOF MS ES+
1.39e+007

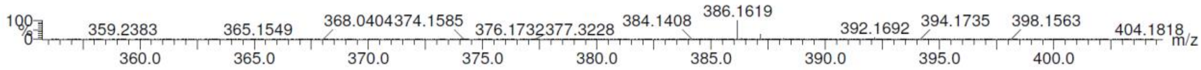

Minimum:				-1.5				
Maximum:		5.0	50.0	50.0				
Mass	Calc. Mass	mDa	PPM	DBE	i-FIT	Norm	Conf(%)	Formula
386.1619	386.1617	0.2	0.5	15.5	1147.6	n/a	n/a	C22 H20 N5 O2

2,6-Dibenzyl-3-(4,5-dihydrooxazol-2-yl)-2,4,5,6-tetrahydro-7*H*-pyrazolo[3,4-*c*]pyridin-7-one (**90**)

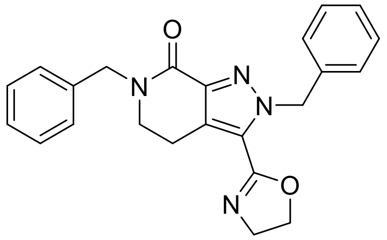

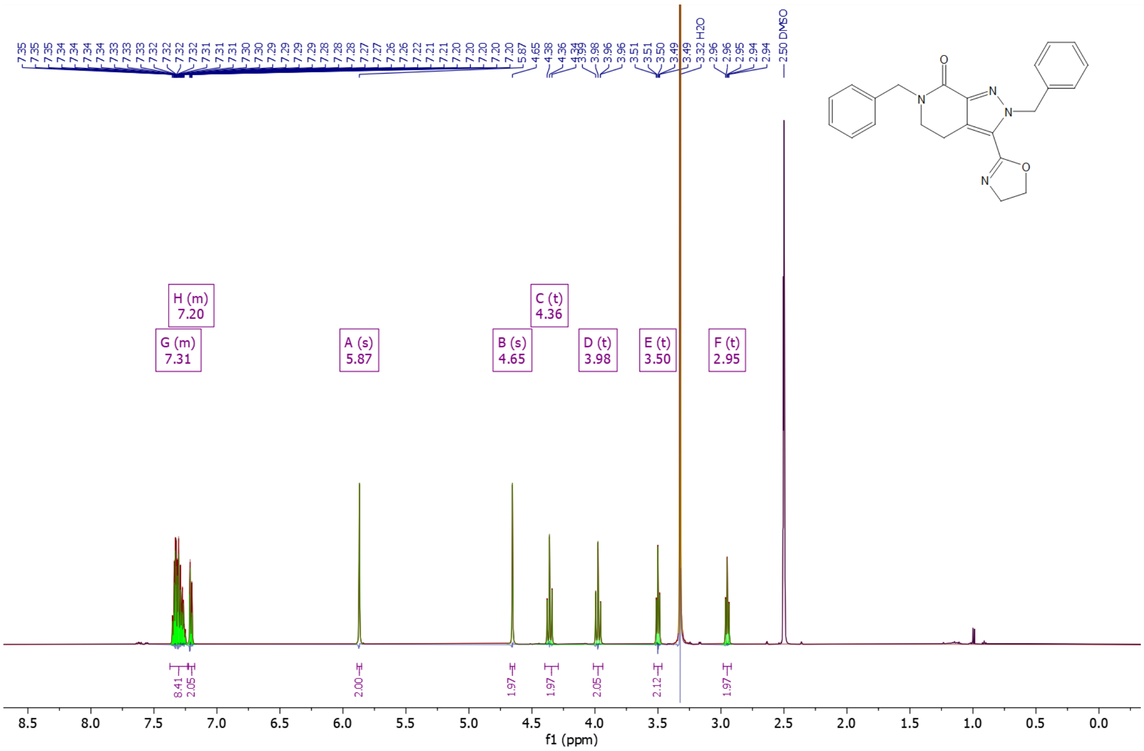

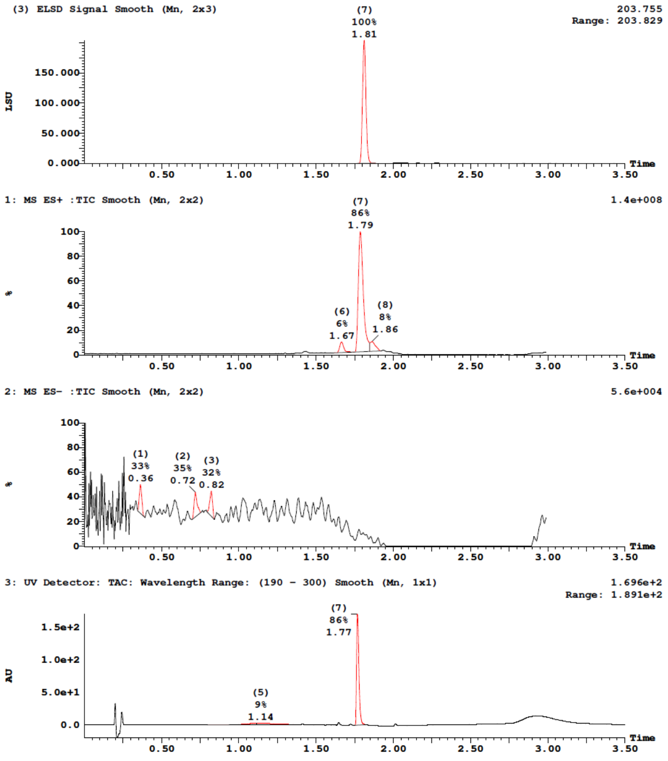

Peak ID	Compound	Time	Mass Found
7		1.79	Not Found

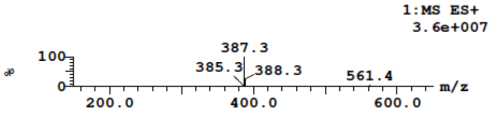

2,6-Dibenzyl-7-oxo-4,5,6,7-tetrahydro-2*H*-pyrazolo[3,4-*c*]pyridine-3-carbonitrile (**91**)

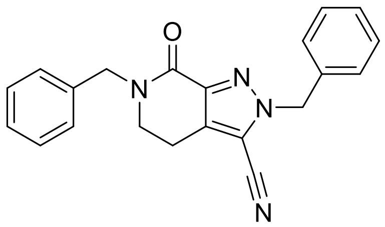

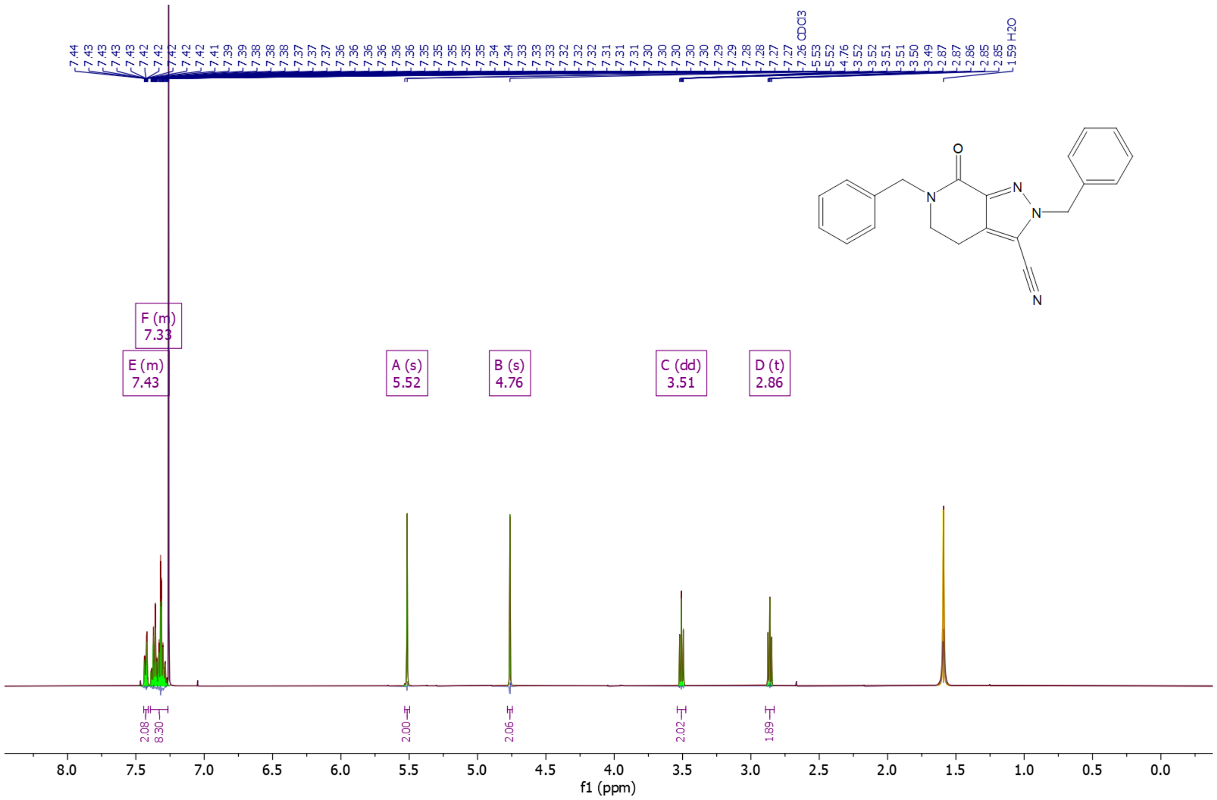

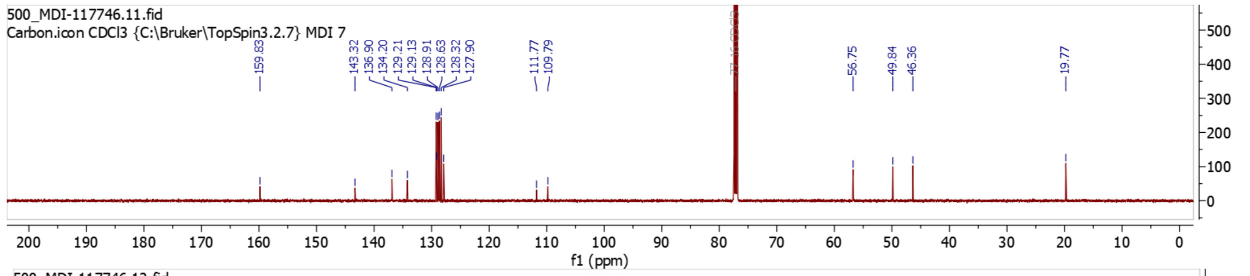

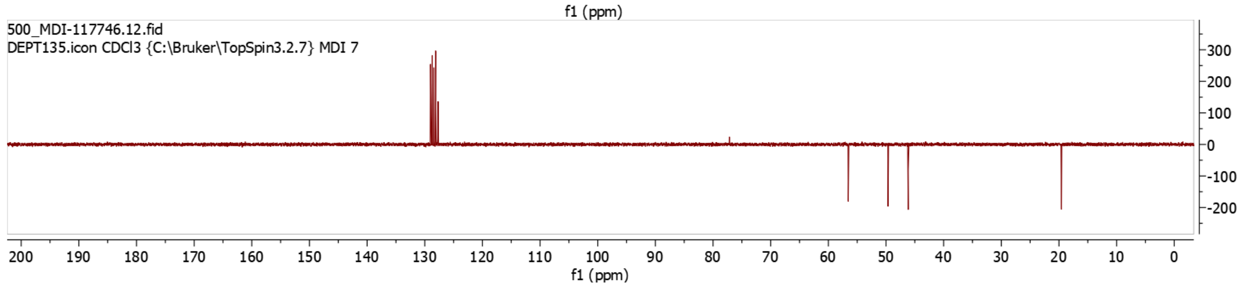

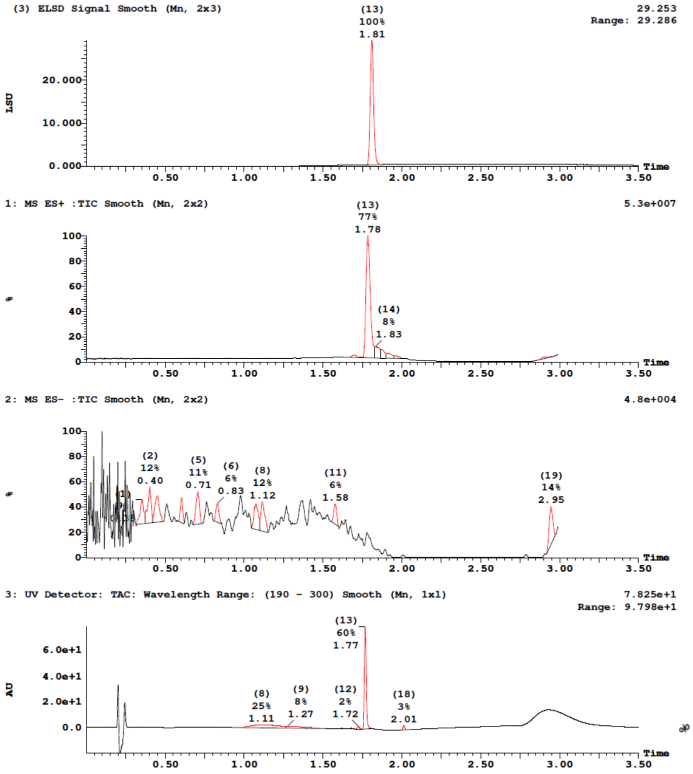

Peak ID	Compound	Time	Mass Found
13		1.78	Not Found

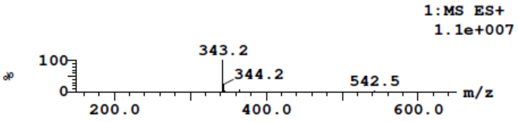

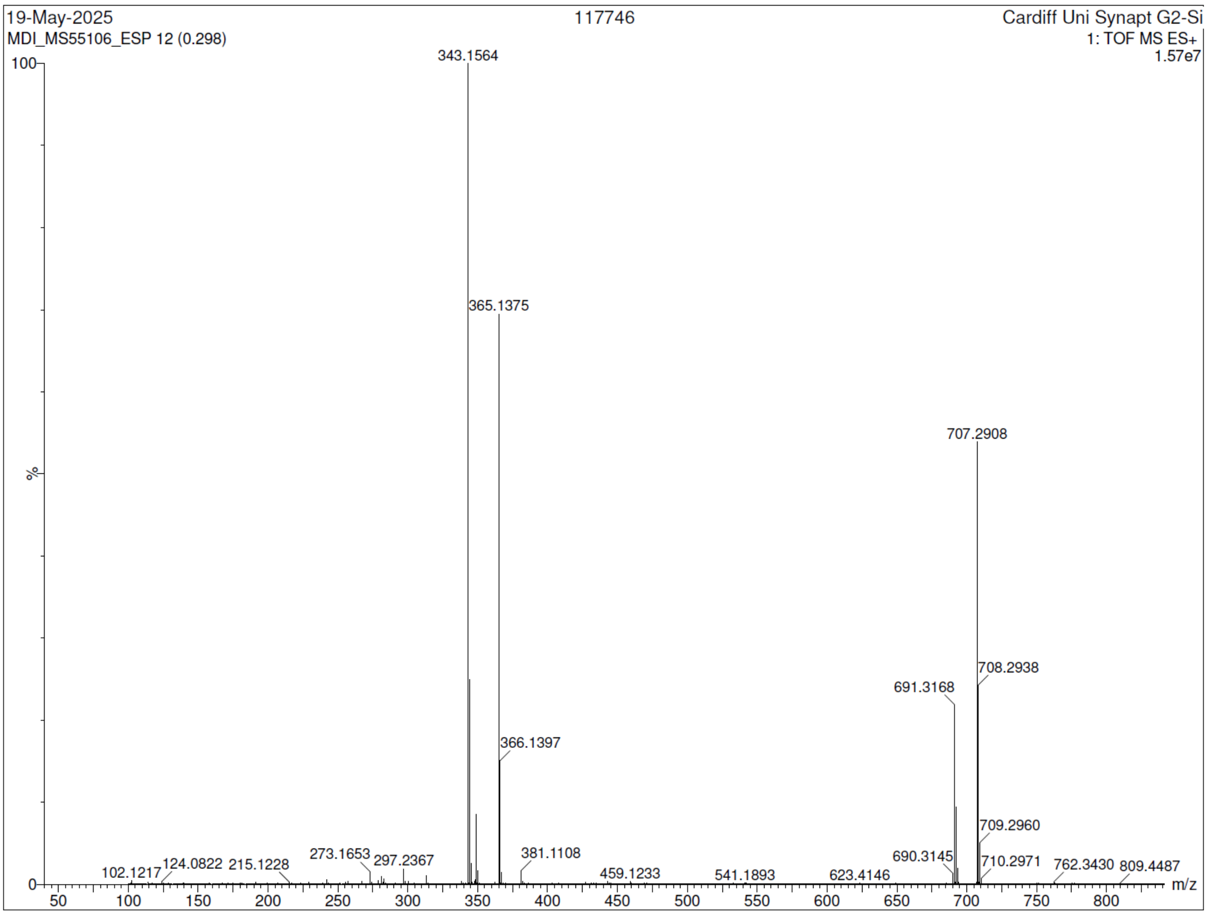

Elemental Composition Report

Single Mass Analysis

Tolerance = 50.0 PPM / DBE: min = -1.5, max = 50.0
Element prediction: Off
Number of isotope peaks used for i-FIT = 3

Monoisotopic Mass, Odd and Even Electron Ions
6 formula(e) evaluated with 1 results within limits (up to 50 closest results for each mass)
Elements Used:
C: 0-21 H: 0-19 N: 0-4 O: 0-1

19-May-2025 117746 Cardiff Uni Synapt G2-Si
MDI_MS55106_ESP 12 (0.298) 1: TOF MS ES+
1.57e+007

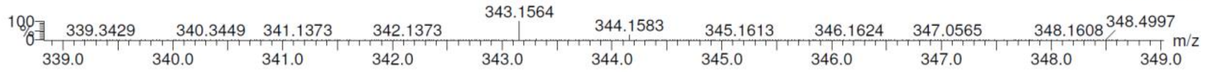

Minimum:				-1.5				
Maximum:		5.0	50.0	50.0				
Mass	Calc. Mass	mDa	PPM	DBE	i-FIT	Norm	Conf(%)	Formula
343.1564	343.1559	0.5	1.5	14.5	1174.7	n/a	n/a	C21 H19 N4 O
